# Changes in genital Human Papillomavirus (HPV) prevalence among urban females a decade after the Malaysian HPV vaccination program

**DOI:** 10.1371/journal.pone.0278477

**Published:** 2022-12-20

**Authors:** Su Pei Khoo, Nor Adriana Muhammad Ridzuan Tan, Reena Rajasuriar, Nazrila Hairizan Nasir, Patti Gravitt, Chiu Wan Ng, Yin Ling Woo

**Affiliations:** 1 Department of Obstetrics and Gynaecology, Faculty of Medicine, University of Malaya, Kuala Lumpur, Malaysia; 2 Department of Medicine, Faculty of Medicine, University of Malaya, Kuala Lumpur, Malaysia; 3 Division of Family Health Development, Ministry of Health, Putrajaya, Malaysia; 4 Center for Global Health, National Institutes of Health, Bethesda, Maryland, United States of America; 5 Department of Social and Preventive Medicine, Faculty of Medicine, University of Malaya, Kuala Lumpur, Malaysia; International Medical University, MALAYSIA

## Abstract

To increase the coverage of HPV vaccination, Malaysia implemented a national school-based vaccination program for all 13-year-old girls in 2010. Two years later, a clinic-based catch-up program was started for 16 to 21-year-old girls. We assessed the prevalence of a range of HPV genotypes, among a sample of urban women within the age groups of 18–24 and 35–45 years in 2019–2020, a decade into the national vaccination program. The HPV prevalence was then compared to that reported in an unvaccinated population in 2013–2015. We sampled a total of 1134 participants, comprising of 277 women aged 18–24 years and 857 women aged 35–45 years, from several urban clinics in the state of Selangor. Participants provided a self-acquired vaginal sample for HPV genotyping. Comprehensive sociodemographic and vaccination history were collected. The HPV vaccination coverage among women in the younger age group increased from 9.3% in 2013–2015 to 75.5% in 2019–2020. The prevalence of vaccine-targeted HPV16/18 decreased 91% (CI: 14.5%–99.0%) among the younger women, from 4.0% in 2013–2015 to 0.4% in 2019–2020. There was also an 87% (CI: 27.5%–97.5%) reduction in HPV6/11/16/18. There was no difference in the prevalence of non-vaccine targeted HPV genotypes among younger women. The HPV prevalence among older women, for both vaccine targeted and non-vaccine targeted genotypes in 2019–2020, did not differ from 2013–2015. The observed decline in prevalence of vaccine-targeted HPV genotype among younger women a decade after the national HPV vaccination program is an early indication of its effectiveness in reducing the burden of cervical cancer.

## Introduction

Cervical cancer is one of the most preventable types of cancer, yet it is the fourth most common cancer affecting women globally. In 2020, over 600,000 women were diagnosed with cervical cancer, and more than half of them succumbed to this disease [1]. Almost all cervical cancers are due to persistent infection of high-risk Human Papillomavirus (HRHPV) [2]. Of the 14 known HRHPV genotypes, HPV16 and HPV18 collectively account for more than 70% of all cervical cancer cases, while HPV31/33/45/52/58 account for another 20% of total cases [3]. The low-risk HPV genotypes, HPV6 and HPV11, are responsible for 90% of ano-genital warts [4]. Currently available HPV vaccines primarily target the HPV16/18 genotypes—solely in a bivalent formulation, in combination with HPV 6/11 in a quadrivalent formulation and with the addition of the other 5 HRHPV in a nonavalent formulation.

The goal of national HPV vaccination program is to reduce the incidence of cervical cancer, but this reduction will not be observed immediately due to the long natural history of HPV infection leading to the development of cancer [5–7]. Data from clinical trials and studies from real-world settings have shown the efficacy and effectiveness of HPV vaccines against cervical HPV infection, ano-genital warts and high-grade precancerous lesions [8–10]. Thus, observations of decreasing HPV prevalence among women after the implementation of vaccinations programs have been used as an early indication of program effectiveness. Many high-income countries with long established national vaccination programs, such as Australia and United States, have reported a reduction in the prevalence of vaccine-targeted HPV after many years of vaccination which augurs well for the women in these countries. Nevertheless, no significant change in the prevalence of non-vaccine targeted HPV has been observed in these countries [11, 12].

Malaysia is an upper-middle-income Southeast Asian nation of 32 million population [13]. Cervical cancer is the second most common cancer among the country’s women aged between 15 and 44 years old, with an age-standardized incidence rate of 10.2 per 100000 women in 2020 [14]. In 2010, Malaysia became the first country in Southeast Asia to implement a national HPV vaccination program, where free vaccinations were provided to all school-going-13-year-old girls annually. This was supplemented with a clinic-based catch-up program for unvaccinated 16 to 21 year old girls during the period 2012 to 2019 [15]. Women who were not eligible for inclusion into the national program had the option to buy vaccination services from private health care providers. There has been changes to the vaccination program over the years; the original 3-dose schedule was changed to a 2-dose schedule in 2015 [16], with quadrivalent and bivalent HPV vaccines being used alternately [17].

The national HPV vaccination program has reported high vaccine uptake of more than 80% of targeted school girls annually [18]. However, little else is known concerning the effectiveness of the program. Therefore, we conducted a cross-sectional study to determine the HPV prevalence among a sample of adult women attending public clinics in the state of Selangor, Malaysia a decade after the start of the vaccination program. We then compared our results to an earlier study of HPV prevalence among women sampled from the same study sites over a 3-year period from 2013 to 2015. Since most participants in the earlier study had not been eligible for the national vaccination program, that study provided information of HPV prevalence in the pre-vaccination era in Malaysia [19].

## Materials and methods

### Study design and setting

This was a cross-sectional study carried out between June 2019 and December 2020 (hereinafter referred to as 2019 study) in several public clinics in Selangor, the most populous state in Malaysia. The participants were recruited, via convenience sampling, from women who visited public clinics for routine health checks or who were accompanying caregivers to patients. Two specific age groups were selected: 18-24-year-old women who were eligible to receive vaccination under the national HPV vaccination program (either the school-based or catch-up program) and 35-45-year-old women who were not. Women in these age groups who agreed to self-collect a vaginal sample for HPV testing and answered a questionnaire were recruited for the study. Self-sampling is the recommended sampling approach for HPV testing by both local and international guidelines [21, 22]. The exclusion criteria were pregnancy, partial/complete hysterectomy, currently menstruating, acute illness and never been sexually active. This study obtained ethical approval from the Ministry of Health, Medical Research Ethics Committee (NMRR-13-3229-44851) and the University of Malaya Medical Ethics Committee (MREC ID 20181219–6935). Written informed consent was obtained from all participants.

### Sample collection

All participants self-collected a vaginal sample using a dry flocked swab (Copan Diagnostic Inc, Italy, #552C/80mm) enclosed in a plastic packaging tube. Verbal and diagrammatic instructions were provided by trained research staff before the self-sampling procedure. Participants were instructed to gently insert the swab into the lower vagina and rotate it ten times before placing back into its packaging tube. All swabs were kept at room temperature for a maximum of two weeks before sample preparation. The swabs were swirled vigorously for 20 seconds in a tube containing 5ml of ThinPrep PreservCyt Solution (Hologic, USA) for the preservation of cells. A 1 ml-aliquot cell suspension was then stored at 2–8°C up to 6 months before DNA extraction was performed in batches.

### HPV genotyping

HPV genotyping was performed using the BGISEQ-100 (Beijing Genome Institute (BGI)-assembled Ion Proton Sequencer from Life Technologies, South San Francisco, California, USA) as previously described [20]. Briefly, DNA amplification were carried out using a multiplex polymerase chain reaction (PCR) targeting the β-globin (HBB) gene and L1 gene of 16 HPV DNA; 14 high risk genotypes: 16, 18, 31, 33, 35, 39, 45, 51, 52, 56, 58, 59, 66, 68; and 2 low-risk genotypes: 6 and 11. The detected HPV sequence was aligned with a BGI-curated standard references HPV database from the National Centre for Biotechnology Information (NCBI) by using the HPV typing software (China Food and Drug Administration (CFDA) registration number: 022702129, registered under Beijing Genome Institute). The sample was defined as HPV-positive for a corresponding type if the HPV genotype reads and HBB reads were over a threshold set by the manufacturer, otherwise HPV-negative. Samples with insufficient HBB DNA was defined as invalid regardless of HPV genotype reads.

### Data collection

Immediately after sample collection, the women were asked to fill in a questionnaire, providing socio-demographic data and vaccination history [19].

### Statistical analysis

To assess the change in HPV prevalence, a subset of age-stratified dataset (18–24 and 35–45 years old) was extracted from the prevalence study conducted in 2013–2015 (hereinafter referred to as 2013 study) [19]. The distribution of participants across two study periods was described according to age groups. The differences in characteristics between study periods were analysed using Pearson Chi Square test. HPV prevalence estimates (with 95% confidence interval) were computed by age groups for the following categories: Any HPV (HRHPV+HPV6/11), high-risk HPV (HRHPV), vaccine-targeted HPV genotypes (HPV16/18 and HPV6/11/16/18), HPV31/33/45/52/58 and non-HPV16/18 genotypes. We estimated prevalence changes between study periods using formula of 1-prevalence ratio (PR). The changes in prevalence were presented as percentage, with corresponding 95% confidence interval. Prevalence ratio was used as it allowed a quantification of the trend in prevalence changes between the two study periods [21]. The shift in the HPV genotype distribution between study periods was also observed using prevalence estimates (with 95% confidence interval). HPV prevalence estimates (with 95% confidence interval) and changes were also computed by subgroups of vaccination (unvaccinated and vaccinated groups). Study participants were classified as ‘vaccinated’ by self-reporting of receiving at least 1 dose of the HPV vaccine.

Sample size was calculated using OpenEpi software [22]. The prevalence of HPV16/18 among 18-24-year-old women in the 2013 study was reported to be 4% [19]. With an estimated reduction of 90% in the prevalence of HPV16/18 among women aged 18–24 in 2019, the sample size needed for this study was 368, at the desired level of confidence interval at 95% [22]. We assumed that there would be no difference in the HPV prevalence among women aged 35–45 between 2013 and 2019. Therefore, the estimated HRHPV prevalence would remain at 7% [19]. Based on the desired level of confidence interval at 95% and a margin of error at 2%, the calculated sample size for women aged 35–45 was 625 [22]. All analyses were performed using STATA version 16 (StataCorp LLC, USA). A two-tailed *p*-value of <0.05 was considered statistically significant.

## Results

### Study participants

We recruited a total of 1149 participants between June 2019 to December 2020. However, 15 (1.3%) of them were excluded due to insufficient DNA for HPV genotyping and the final analysis was carried out on 1134 participants with valid HPV genotyping results. There were 277 women in the 18–24 years age group and 857 women in the 35–45 years age group. We compared our results with that obtained from the analysis of 428 participants from the same age groups in the 2013 study. There were 75 women in the 18–24 years age group and 353 in the 35–45 years age groups in the 2013 study [20].

The demographic characteristics of participants from the 2013 and 2019 studies are shown in Table 1. Among participants in the 18–24 years age group, the vaccine coverage was 75.5% in the 2019 study which was significantly higher than the 2013 study (9.3%). There were more participants in the 2019 study who were from the Chinese ethnic background and who had tertiary education. However, there were less participants from the low-income group in the 2019 study.

**Table 1:** Study participants characteristics across two study periods.

Variable	Total	Study periods		*p*-value*
		2013–2015	2019–2020	
	N (%)	n (%)	n (%)	
**18–24 years old**				
Overall	352 (100.0)	75 (100.0)	277 (100.0)	
**Ethnicity**				<0.001
Malay	213 (60.5)	45 (60.0)	168 (60.7)	
Chinese	75 (21.3)	8 (10.7)	67 (24.2)	
Indian	37 (10.5)	19 (25.3)	18 (6.5)	
Other	27 (7.7)	3 (4.0)	24 (8.7)	
**Marital status**				0.405
Currently married	230 (65.3)	53 (70.7)	177 (63.9)	
Single	111 (31.5)	19 (25.3)	92 (33.2)	
Separated/divorced/widowed	11 (3.1)	3 (4.0)	8 (2.9)	
**Education level**				0.035
None/Primary	25 (7.1)	8 (10.7)	17 (6.1)	
Secondary	190 (54.0)	47 (62.7)	143 (51.6)	
Tertiary and above	137 (38.9)	20 (26.7)	117 (42.2)	
^§^ **Household income (USD)**				<0.001
< 1195	294 (83.5)	74 (98.7)	220 (79.4)	
> 1195	58 (16.5)	1 (1.3)	57 (20.6)	
**Received ≥ 1 dose of HPV vaccine**				<0.001
No	136 (38.6)	68 (90.7)	68 (24.6)	
Yes	216 (61.4)	7 (9.3)	209 (75.5)	
**35–45 years old**				
Overall	1210 (100.0)	353 (100.0)	857 (100.0)	
**Ethnicity**				<0.001
Malay	704 (58.2)	164 (46.5)	540 (63.0)	
Chinese	271 (22.4)	86 (24.4)	185 (21.6)	
Indian	203 (16.8)	98 (27.8)	105 (12.3)	
Other	32 (2.6)	5 (1.4)	27 (3.2)	
**Marital status**				0.634
Currently married	1124 (92.9)	325 (92.1)	799 (93.2)	
Single	22 (1.8)	6 (1.7)	16 (1.9)	
Separated/divorced/widowed	64 (5.3)	22 (6.2)	42 (4.9)	
**Education level**				<0.001
None/Primary	53 (4.4)	36 (10.2)	17 (2.0)	
Secondary and below	498 (41.2)	219 (62.0)	279 (32.6)	
Tertiary and above	659 (54.5)	98 (27.8)	561 (65.5)	
^§^ **Household income (USD)**				<0.001
< 1195	650 (53.7)	293 (83.0)	357 (41.7)	
≥ 1195	560 (46.3)	60 (17.0)	500 (58.3)	
**Received ≥ 1 dose of HPV vaccine**				0.001
No	1135 (93.8)	344 (97.5)	791 (92.3)	
Yes	75 (6.2)	9 (2.5)	66 (7.7)	

*P-value was generated using Pearson Chi-Square test.

^§^The household income was calculated based on a conversion rate of 0.24 (Malaysian Ringgit to US dollar). The cut-off value of USD1195 denotes the poorest 40% in the income distribution of the Malaysian population.

Missing data was excluded from the analysis.

Abbreviations: N/A; Not available

The uptake of vaccine among participants aged 35–45 years old in the 2019 study was 7.7% which was significantly higher than the 2013 study (2.5%). In this age group of 35–45, more Indians (27.8%) was recruited in the 2013 study compared to the 2019 study (12.3%). Generally, more participants attended tertiary education (65.5% vs 27.8%) and had a higher household income (58.3% vs 17.0%) in the 2019 study.

### Changes of HPV prevalence across two study periods

Among women aged 18–24, there was a statistically significant 91% (CI: 14.5%–99.0%) decline in the prevalence of vaccine targeted HPV genotype (HPV16/18) from 4% in 2013 to 0.4% in 2019. An 87% (CI: 27.5%–97.5%) statistically significant reduction was also observed in the prevalence of HPV genotypes targeted by quadrivalent vaccine (HPV6/11/16/18) in this age group. There were no statistically significant changes in the prevalence of non-vaccine targeted HPV genotypes between these study periods. Among women aged 35–45, there was no difference in the prevalence for all HPV categories between 2013 and 2019. The detailed HPV prevalence for both age groups across two study periods is shown in Table 2.

**Table 2:** HPV prevalence among women aged 18–24 and 35–45 by study periods.

HPV categories	2013–2015		2019–2020		*Prevalence changes		
	HPV positive		HPV positive		%	Lower bound (%)	Upper bound (%)
	n	% (95% CI)	n	% (95% CI)			
**18–24 years old**							
HRHPV+HPV6/11	9	12.0 (6.4–21.5)	23	8.3 (5.6–12.2)	– 30.8	– 66.6	+ 43.2
HRHPV	8	10.7 (5.4–20.0)	23	8.3 (5.6–12.2)	– 22.2	– 63.7	+ 66.9
HPV16/18	3	4.0 (1.3–11.7)	1	0.4 (0.1–2.5)	**– 91.0**	**– 99.0**	**– 14.5**
HPV6/11/16/18	4	5.3 (2.0–13.4)	2	0.7 (0.2–2.9)	**– 86.5**	**– 97.5**	**– 27.5**
HPV31/33/45/52/58	2	2.7 (0.7–10.1)	9	3.3 (1.7–6.1)	+ 21.8	– 73.1	+ 452.0
Non-HPV16/18	5	6.7 (2.8–15.1)	22	7.9 (5.3–11.8)	+ 19.1	– 53.3	+ 204.0
**35–45 years old**							
HRHPV+HPV6/11	26	7.4 (5.1–10.6)	49	5.7 (4.4–7.5)	– 22.4	– 51.0	+ 22.9
HRHPV	24	6.8 (4.6–10.0)	46	5.4 (4.0–7.1)	– 21.1	– 51.0	+ 27.3
HPV16/18	6	1.7 (0.8–3.7)	9	1.1 (0.6–2.0)	– 38.2	– 77.8	+ 72.3
HPV6/11/16/18	9	2.5 (1.3–4.8)	13	1.5 (0.9–2.6)	– 40.5	– 74.3	+ 37.9
HPV31/33/45/52/58	12	3.4 (1.9–5.9)	18	2.1 (1.3–3.3)	– 38.2	– 69.9	+ 26.9
Non-HPV16/18	18	5.1 (3.2–5.9)	37	4.3 (3.1–5.9)	– 15.3	– 51.1	+ 46.7

* Prevalence changes were calculated using HPV prevalence reported in 2013–2015 as the reference. Prevalence changes that were statistically significant between 2013–2015 and 2019–2020 were marked bold.

### HPV genotype distribution by age group across two study periods

**Fig 1:**
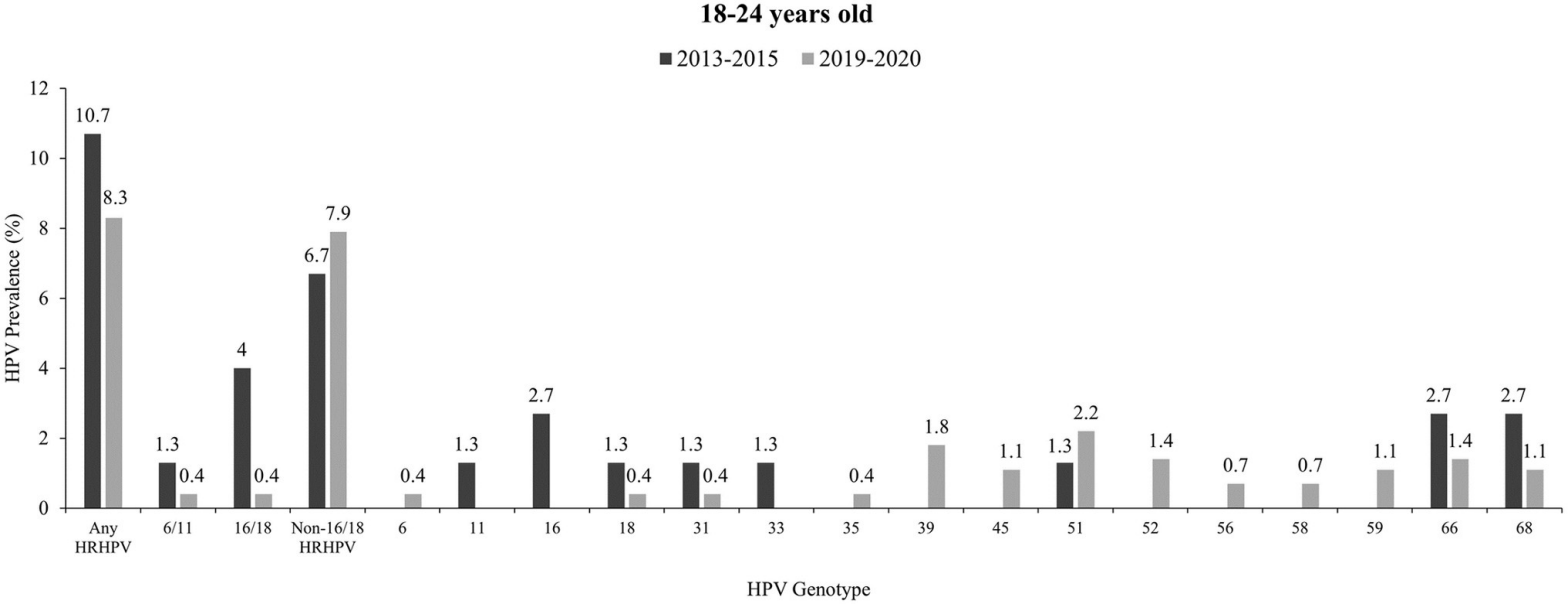
Distribution of HPV genotypes among women aged 18–24 across two study periods. Non-16/18 HRHPV infections contributed to 95% (7.9% of 8.3%) of the overall HRHPV prevalence in 2019–2020 whereas in 2013–2015, 62.6% (6.7% of 10.7%) of HRHPV prevalence was made up of non-16/18 HRHPV infections. Abbreviations: CI, confidence interval; HPV, Human papillomavirus.

**Fig 2:**
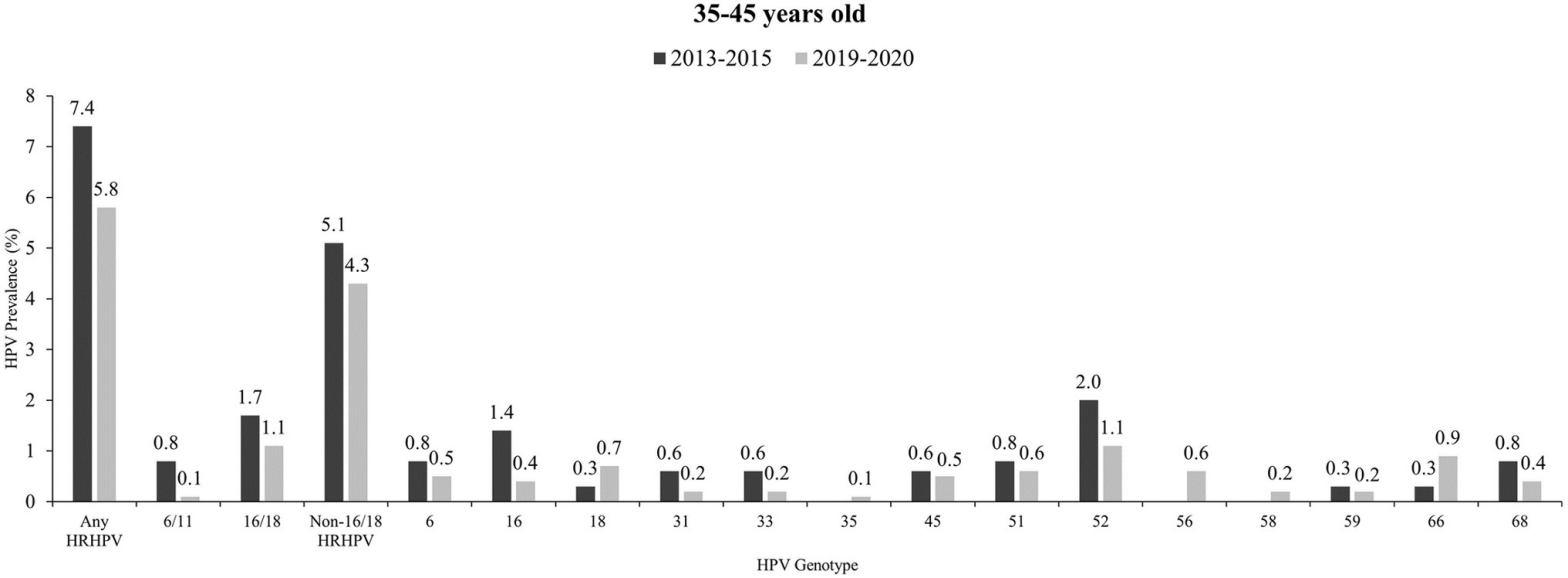
Distribution of HPV genotypes among women aged 35–45 across two study periods. Other than HPV52, vaccine-targeted HPV genotype such as HPV18 remained on of the most frequently detected HPV genotypes across both study periods. Abbreviations: CI, confidence interval; HPV, Human papillomavirus. HPV11 and HPV39 infections were not reported among women aged 35–45 across both study periods.

The distribution of HPV genotype according to age groups across two study periods is demonstrated in Figs 1 and 2. In 2019, non-16/18 HRHPV infections made up 95% (7.9% of 8.3%) of the overall HRHPV prevalence among 18-24-year-old women compared to 62.6% (6.7% of 10.7%) in 2013. HPV16 was the most common HPV genotype detected in 2013 whereas in 2019, HPV51 had the highest frequency irrespective of vaccination status (please refer to Fig 1).

Among women aged 35–45, HPV52 appeared to be the most common HPV genotype detected in both study periods. Other than that, vaccine-targeted HPV genotypes such as HPV18 remained one of the top 3 most common HPV genotypes in 2019 (please refer to Fig 2).

### HPV prevalence among women aged 18–24 by vaccination status across two study periods

The prevalence of HPV among women aged 18–24 by vaccination status between study periods are shown in Table 3. The prevalence of all HPV categories among unvaccinated 18-24-year-old participants in the 2019 study were not different from the prevalence reported in the 2013 study. Of note, none of the vaccinated women in both 2013 and 2019 studies was positive for HPV16/18 infection. Within the 2019 study, the prevalence of all HPV categories did not differ by vaccination status.

**Table 3:** HPV prevalence among women aged 18–24 by vaccination status and study periods.

HPV categories	2013–2015		2019–2020		*Prevalence changes (95% CI) compared to overall prevalence in 2013–2015 by vaccination status in 2019–2020			Prevalence changes (95% CI) by vaccination status in 2019–2020		
	HPV positive		HPV positive							
	n	% (95% CI)	n	% (95% CI)	%	Lower bound (%)	Upper bound (%)	%	Lower bound (%)	Upper bound (%)
**18–24 years old**										
**HRHPV+HPV6/11**										
Overall	9	12.0 (6.4–21.5)	23	8.3 (5.6–12.2)	– 30.8	– 66.6	+ 43.2			
Unvaccinated	9	13.2 (6.2–23.6)	5	7.4 (2.4–16.3)	– 38.7	– 78.4	+ 73.8	Reference		
Vaccinated	0	0	18	8.6 (5.2–13.3)	– 28.2	– 66.3	+ 52.8	+ 17.1	– 54.8	+ 203.6
**HRHPV**										
Overall	8	10.7 (5.4–20.0)	23	8.3 (5.6–12.2)	– 22.2	– 63.7	+ 66.9			
Unvaccinated	8	11.8 (5.2–21.9)	5	7.4 (2.4–16.3)	– 31.1	– 76.3	+ 100.6	Reference		
Vaccinated	0	0	18	8.6 (5.2–13.3)	– 19.3	– 64.0	+ 77.9	+ 17.1	– 54.8	+ 203.6
**HPV16/18**										
Overall	3	4.0 (1.3–11.7)	1	0.4 (0.1–2.5)	**– 91.0**	**– 99.0**	**– 14.5**			
Unvaccinated	3	4.4 (0.9–12.4)	1	1.5 (0.0–7.9)	– 63.2	– 96.1	+ 245.1	N/A		
Vaccinated	0	0	0	0	N/A			N/A		
**HPV6/11/16/18**										
Overall	4	5.3 (2.0–13.4)	2	0.7 (0.2–2.9)	**– 86.5**	**– 97.5**	**– 27.5**			
Unvaccinated	4	5.9 (1.6–14.4)	1	1.5 (0.0–7.9)	– 72.4	– 96.9	+ 140.7	Reference		
Vaccinated	0	0	1	0.5 (0.0–2.6)	**– 91.0**	**– 99.0**	**– 21.0**	– 67.5	– 97.9	+ 413.2
**HPV31/33/45/52/58**										
Overall	2	2.7 (0.7–10.1)	9	3.3 (1.7–6.1)	+ 21.8	– 73.1	+ 452.0			
Unvaccinated	2	2.9 (0.0–10.2)	2	2.9 (0.0–10.2)	+ 10.3	– 84.0	+ 661.6	Reference		
Vaccinated	0	0	7	3.3 (1.4–6.8)	+ 25.6	– 73.3	+ 491.3	+ 13.9	– 75.8	+ 435.2
**Non-HPV16/18**										
Overall	5	6.7 (2.8–15.1)	22	7.9 (5.3–11.8)	+ 19.1	– 53.3	+ 204.0			
Unvaccinated	5	7.4 (2.4–16.3)	4	5.9 (1.6–14.4)	– 11.8	– 76.0	+ 215.2	Reference		
Vaccinated	0	0	18	8.6 (5.2–13.3)	+ 29.2	– 50.0	+ 235.7	+ 46.4	– 48.7	+ 317.7

*Prevalence changes were calculated using overall HPV prevalence (regardless of vaccination status) reported in 2013–2015 as the reference.

Abbreviations: CI, confidence interval; HPV, Human papillomavirus

## Discussion

This is the first Malaysian study that reports the HPV prevalence among women a decade after the national HPV vaccination program was implemented. We demonstrated a 91% decline in the vaccine-targeted HPV prevalence (HPV16/18) among young women aged 18–24. There was no significant change in non-vaccine targeted HPV prevalence in this age group. Among older women who were not eligible for the vaccination program, no significant change in HPV prevalence was observed between 2013 and 2019.

Our finding, of large reductions in HPV16/18 in the post-vaccination era among young women is consistent with findings from other countries with a national vaccination program [11, 12, 23]. As the bivalent and quadrivalent vaccines were used alternately throughout the 10-year vaccination program, our study also observed a decline of 87% (CI: 27.5%–97.5%) in the quadrivalent vaccine-targeted HPV prevalence (HPV6/11/16/18) across both study periods. This finding can be generalizable to the Malaysian population as the vaccine coverage in our study (75.5%) was comparable to the national statistics (>80%) [18]. This is the first indication of the potential effectiveness of the national HPV vaccination program in reducing vaccine-targeted HPV prevalence in Malaysia.

Despite not statistically significant, our study observed an increasing trend in non-vaccine targeted HPV prevalence among women aged 18–24. The increasing trend of non-vaccine targeted HPV prevalence in the post-vaccination era is similar to those reported in many other countries with consistently high vaccine coverage [11, 12, 24–26]. This suggests that the effectiveness of HPV vaccines is type-specific [27]. This emergence of non-vaccine targeted such as HPV52 and 58 might be clinically important as they were among the common HPV genotypes associated with high-grade precancerous lesions and invasive cervical cancer after HPV16 and 18 in the Asian population [28–31]. There were concerns raised on possible diminished impact of vaccination program in the Asian population compared to the western populations thus warrants further investigations to inform vaccination policy recommendations [32, 33].

Of note, the prevalence of HPV6/11 alone declined from 1.3% in 2013 to 0.4% in 2019, even though only a portion of the vaccinated cohorts would have received quadrivalent vaccines. The impact of HPV vaccination program on reducing genital warts following a decline in HPV6/11 has previously been shown in countries utilizing quadrivalent vaccines [9]. However, the sample size in this study was small and information on the type of vaccines received by each participant were not available, thus necessitates further evaluation. This suggests that consideration can be given to the choice of vaccine depending on the local burden of genital warts.

Several studies have demonstrated a significant decline in the prevalence of overall HRHPV among vaccine-eligible individual years after implementation of the vaccination program [11, 12, 24]. On the contrary, our study did not show statistically significant changes in the overall HRHPV prevalence among women aged 18–24 between 2013 and 2019. This is because the HPV infections in the Malaysian setting were dominated with non-vaccine targeted HPV infections thus the reduction of vaccine-targeted HPV prevalence had minimal impact on the overall HRHPV prevalence. Additionally, the sample size of our study might not be sufficient to detect the difference in HRHPV prevalence as it was designed to assess the differences in vaccine-targeted HPV prevalence between 2013 and 2019.

Real-world evidence previously demonstrated herd effects among the unvaccinated women where there is high vaccine coverage [34]. Despite high vaccine coverage among the 18-24-year-old women in our study, herd effect was not observed among the unvaccinated individuals in our study. Compared to the 2013 study, there were no statistically significant differences in overall HRHPV, vaccine-targeted HPV genotype (HPV16/18 and HPV6/11/16/18), HPV31/33/45/52/58 and non-HPV16/18 HRHPV prevalence between vaccinated and unvaccinated individuals in the 2019 study. This is likely due to the small number of HPV-positive subjects and unvaccinated individuals in our study.

Even though not routinely indicated, a significant higher proportion of women aged 35–45 reported to have received HPV vaccines in 2019 compared to 2013 (7.7% vs 2.5%). This may be an effect of convenient sampling approach which have oversampled women with higher education level and household income in the 2019 study. The prevalence of overall HRHPV and vaccine-targeted HPV (HPV16/18) and non-vaccine targeted HPV prevalence in 2019 were not statistically significantly different from 2013 among the 35-45-year-old women. This finding is consistent with other published studies where no significant changes were shown in terms of HPV type distribution over the years among the older age group who did not benefit from the vaccination program [35, 36]. This also explains why HPV18 remains one of the most common HRHPV types detected in this age group, similar to the HPV genotype distribution observed earlier in 2013.

A strength of this study is the prevalence reporting of a range of HPV genotypes, including the vaccine-targeted and non-vaccine targeted HPV genotypes in the post-vaccination era among women aged 18–24, who were targets of the program. We also included an older group of women aged 35–45, who were not eligible in the program. This allowed an assessment of HPV prevalence changes between the pre-vaccination era (using HPV prevalence reported in 2013–2015) and post-vaccination era as an indicator of the program effectiveness. Additionally, our study population comprised of multi-ethnic women from urban public clinics, which was a representation of the highly urbanized, multi-ethnic Malaysian setting [37, 38]. To the best of our knowledge, this is the first study in Malaysia and Southeast Asia which has investigated the potential impact of the national HPV vaccination program after a decade of implementation. However, this study has several limitations. The number of subjects recruited for the age group 18–24 in the 2013 study was small (N = 75) therefore further analysis to adjust for other risk factors could not be performed due to a small number of HPV16/18-positive subject. Additionally, the recruitment for this study was prematurely closed due to the COVID-19 pandemic, therefore the number of women recruited for the age group 18–24 was 277 instead of the target sample size of 368 subjects. A post hoc power analysis was conducted and the calculated power of the study based on the existing sample size was 73.2% [22]. Other than that, the convenient sampling approach might have led to potential sampling bias. Vaccination history and measures of sociodemographic were self-reported and could be subjected to response desirability and recall bias.

We described the changes in overall HPV (HRHPV+/-HPV6/11), vaccine-targeted HPV (HPV16/18 and HPV6/11/16/18) and non-vaccine targeted HRHPV (non-HPV16/18) prevalence among women aged 18–24, the target group for the national HPV vaccination program in Malaysia. A 91% and 87% decline in the prevalence of HPV16/18 and HPV6/11/16/18, respectively, was observed after 10 years of the national school-based HPV vaccination program. There were no significant changes in the overall HPV prevalence among the older unvaccinated women aged 35–45. The low detection of HPV16/18 in the young women targeted in the national HPV vaccination program suggests the early effectiveness of the program.

## Supporting information

S1 Data Dataset for the study. (PDF)
