# Supplementary material for: Changes in genital Human Papillomavirus (HPV) prevalence among urban females a decade after the Malaysian HPV vaccination program
Source: PLoS One. 2022 Dec 20;17(12):e0278477. doi: 10.1371/journal.pone.0278477 (PMC9767374; doi:10.1371/journal.pone.0278477)
Supplement: S1 Data — (PDF) [file pone.0278477.s001.pdf]

no	era	agegroup	Vaxprogram	ethnic	marital_regp	educat	income	vaccine	sexdebut_gp	lifetime_part
1	2013-2015	18-24 yo	Not elligible	Chinese	currently married	Secondary	<USD1195	no	18 yo and below	3 and above
2	2013-2015	35-45 yo	Not elligible	Malay	currently married	None-primary	<USD1195	no	25-29 yo	1
3	2013-2015	35-45 yo	Not elligible	Chinese	currently married	Secondary	<USD1195	no	19-24 yo	1
4	2013-2015	35-45 yo	Not elligible	Malay	currently married	Secondary	<USD1195	no	19-24 yo	1
5	2013-2015	35-45 yo	Not elligible	Malay	currently married	Secondary	<USD1195	no	19-24 yo	
6	2013-2015	35-45 yo	Not elligible	Chinese	currently married	Secondary	<USD1195	no	19-24 yo	1
7	2013-2015	35-45 yo	Not elligible	Malay	currently married	Secondary	<USD1195	no	19-24 yo	1
8	2013-2015	35-45 yo	Not elligible	Indian	currently married	Tertiary and above	<USD1195	no	25-29 yo	1
9	2013-2015	18-24 yo	Not elligible	Malay	currently married	Secondary	<USD1195	no	18 yo and below	1
10	2013-2015	35-45 yo	Not elligible	Chinese	currently married	Secondary	≥USD1195	no	19-24 yo	1
11	2013-2015	18-24 yo	Not elligible	Malay	currently married	Secondary	<USD1195	no	18 yo and below	1
12	2013-2015	35-45 yo	Not elligible	Chinese	currently married	Secondary	<USD1195	no	19-24 yo	1
13	2013-2015	35-45 yo	Not elligible	Malay	currently married	Secondary	<USD1195	no	30 and above	1
14	2013-2015	35-45 yo	Not elligible	Chinese	currently married	Secondary	<USD1195	no	25-29 yo	1
15	2013-2015	18-24 yo	Not elligible	Malay	currently married	Secondary	<USD1195	no	18 yo and below	
16	2013-2015	35-45 yo	Not elligible	Malay	currently married	Secondary	<USD1195	no	25-29 yo	
17	2013-2015	35-45 yo	Not elligible	Chinese	currently married	Secondary	<USD1195	no	19-24 yo	1
18	2013-2015	35-45 yo	Not elligible	Indian	currently married	Secondary	<USD1195	no	19-24 yo	1
19	2013-2015	35-45 yo	Not elligible	Other	currently married	Secondary	<USD1195	no	30 and above	1
20	2013-2015	35-45 yo	Not elligible	Malay	currently married	Tertiary and above	<USD1195	no	19-24 yo	
21	2013-2015	35-45 yo	Not elligible	Malay	currently married	Tertiary and above	<USD1195	no	25-29 yo	1
22	2013-2015	35-45 yo	Not elligible	Indian	currently married	Secondary	<USD1195	no	18 yo and below	1
23	2013-2015	35-45 yo	Not elligible	Chinese	currently married	Secondary	<USD1195	no	19-24 yo	1
24	2013-2015	18-24 yo	Not elligible	Malay	currently married	Tertiary and above	<USD1195	no	19-24 yo	1
25	2013-2015	35-45 yo	Not elligible	Malay	currently married	Secondary	<USD1195	no	18 yo and below	3 and above
26	2013-2015	35-45 yo	Not elligible	Malay	currently married	Secondary	<USD1195	no	19-24 yo	1
27	2013-2015	35-45 yo	Not elligible	Malay	currently married	Secondary	<USD1195	no	19-24 yo	1
28	2013-2015	35-45 yo	Not elligible	Chinese	currently married	None-primary	<USD1195	no	25-29 yo	1
29	2013-2015	35-45 yo	Not elligible	Indian	currently married	Secondary	<USD1195	no	25-29 yo	1
30	2013-2015	35-45 yo	Not elligible	Chinese	currently married	Secondary	<USD1195	no	30 and above	1
31	2013-2015	18-24 yo	Not elligible	Malay	single	Secondary	<USD1195	no	18 yo and below	3 and above
32	2013-2015	18-24 yo	Not elligible	Malay	single	Tertiary and above	<USD1195	no	19-24 yo	1
33	2013-2015	18-24 yo	Not elligible	Malay	currently married	Secondary	<USD1195	no	19-24 yo	1
34	2013-2015	18-24 yo	Not elligible	Malay	currently married	Tertiary and above	<USD1195	yes	19-24 yo	1
35	2013-2015	18-24 yo	Not elligible	Malay	currently married	Tertiary and above	<USD1195	no	19-24 yo	1
36	2013-2015	35-45 yo	Not elligible	Indian	currently married	None-primary	<USD1195	no	18 yo and below	1
37	2013-2015	35-45 yo	Not elligible	Malay	currently married	Tertiary and above	<USD1195	no	25-29 yo	1
38	2013-2015	35-45 yo	Not elligible	Chinese	currently married	Tertiary and above	<USD1195	no	25-29 yo	1
39	2013-2015	35-45 yo	Not elligible	Malay	currently married	Tertiary and above	≥USD1195	no	25-29 yo	1

40	2013-2015	35-45 yo	Not elligible	Malay	currently married	Secondary	<USD1195	no	19-24 yo	1
41	2013-2015	35-45 yo	Not elligible	Malay	currently married	None-primary	<USD1195	no	19-24 yo	1
42	2013-2015	18-24 yo	Not elligible	Malay	currently married	Secondary	<USD1195	no	18 yo and below	1
43	2013-2015	35-45 yo	Not elligible	Indian	currently married	Secondary	<USD1195	no	18 yo and below	2
44	2013-2015	35-45 yo	Not elligible	Indian	currently married	Secondary	<USD1195	no	25-29 yo	1
45	2013-2015	35-45 yo	Not elligible	Indian	currently married	Secondary	<USD1195	no	25-29 yo	1
46	2013-2015	35-45 yo	Not elligible	Chinese	single	Secondary	<USD1195	yes	19-24 yo	1
47	2013-2015	18-24 yo	Not elligible	Indian	currently married	Secondary	<USD1195	no	18 yo and below	1
48	2013-2015	35-45 yo	Not elligible	Indian	currently married	Secondary	<USD1195	no	19-24 yo	1
49	2013-2015	35-45 yo	Not elligible	Malay	currently married	Tertiary and above	<USD1195	no	25-29 yo	1
50	2013-2015	35-45 yo	Not elligible	Chinese	currently married	Tertiary and above	<USD1195	no	19-24 yo	2
51	2013-2015	35-45 yo	Not elligible	Chinese	currently married	Secondary	≥USD1195	no	18 yo and below	3 and above
52	2013-2015	35-45 yo	Not elligible	Chinese	currently married	Tertiary and above	<USD1195	no	25-29 yo	1
53	2013-2015	35-45 yo	Not elligible	Indian	currently married	Secondary	<USD1195	no	19-24 yo	1
54	2013-2015	35-45 yo	Not elligible	Malay	currently married	Secondary	<USD1195	no	19-24 yo	1
55	2013-2015	18-24 yo	Not elligible	Malay	currently married	Tertiary and above	<USD1195	no	18 yo and below	1
56	2013-2015	35-45 yo	Not elligible	Malay	currently married	Tertiary and above	≥USD1195	no	30 and above	1
57	2013-2015	35-45 yo	Not elligible	Chinese	currently married	Secondary	<USD1195	no	25-29 yo	2
58	2013-2015	35-45 yo	Not elligible	Chinese	currently married	Secondary	<USD1195	no	18 yo and below	3 and above
59	2013-2015	35-45 yo	Not elligible	Chinese	currently married	Secondary	<USD1195	no	19-24 yo	1
60	2013-2015	18-24 yo	Not elligible	Malay	currently married	Secondary	<USD1195	no	18 yo and below	1
61	2013-2015	35-45 yo	Not elligible	Chinese	currently married	Secondary	≥USD1195	no	18 yo and below	1
62	2013-2015	35-45 yo	Not elligible	Chinese	currently married	Secondary	<USD1195	no	19-24 yo	3 and above
63	2013-2015	35-45 yo	Not elligible	Malay	currently married	Secondary	<USD1195	no	19-24 yo	1
64	2013-2015	35-45 yo	Not elligible	Chinese	currently married	Tertiary and above	<USD1195	yes	25-29 yo	1
65	2013-2015	35-45 yo	Not elligible	Chinese	currently married	Secondary	<USD1195	no	25-29 yo	1
66	2013-2015	18-24 yo	Not elligible	Malay	currently married	Secondary	<USD1195	no	19-24 yo	1
67	2013-2015	18-24 yo	Not elligible	Malay	currently married	Secondary	<USD1195	no	19-24 yo	1
68	2013-2015	35-45 yo	Not elligible	Indian	currently married	Secondary	<USD1195	no	25-29 yo	1
69	2013-2015	35-45 yo	Not elligible	Chinese	currently married	Tertiary and above	≥USD1195	no	19-24 yo	2
70	2013-2015	35-45 yo	Not elligible	Malay	currently married	Secondary	<USD1195	no	19-24 yo	1
71	2013-2015	18-24 yo	Not elligible	Indian	currently married	Secondary	<USD1195	no	19-24 yo	1
72	2013-2015	18-24 yo	Not elligible	Malay	currently married	Tertiary and above	<USD1195	no	18 yo and below	1
73	2013-2015	35-45 yo	Not elligible	Chinese	currently married	Tertiary and above	≥USD1195	no	19-24 yo	2
74	2013-2015	18-24 yo	Not elligible	Malay	currently married	Secondary	<USD1195	no	18 yo and below	1
75	2013-2015	35-45 yo	Not elligible	Chinese	currently married	Secondary	<USD1195	no	19-24 yo	1
76	2013-2015	35-45 yo	Not elligible	Chinese	currently married	Secondary	≥USD1195	no	19-24 yo	2
77	2013-2015	18-24 yo	Not elligible	Other	currently married	Secondary	<USD1195	no	19-24 yo	1
78	2013-2015	35-45 yo	Not elligible	Indian	currently married	Secondary	<USD1195	no	19-24 yo	1
79	2013-2015	35-45 yo	Not elligible	Indian	currently married	Secondary	<USD1195	no	25-29 yo	1

80	2013-2015	35-45 yo	Not elligible	Malay	currently married	Secondary	<USD1195	no	19-24 yo	2
81	2013-2015	35-45 yo	Not elligible	Malay	currently married	Tertiary and above	<USD1195	no	18 yo and below	
82	2013-2015	35-45 yo	Not elligible	Malay	currently married	Tertiary and above	<USD1195	no	25-29 yo	1
83	2013-2015	35-45 yo	Not elligible	Malay	currently married	Secondary	<USD1195	no	19-24 yo	1
84	2013-2015	18-24 yo	Not elligible	Malay	currently married	Tertiary and above	<USD1195	no	19-24 yo	1
86	2013-2015	35-45 yo	Not elligible	Malay	currently married	Secondary	<USD1195	no	19-24 yo	1
87	2013-2015	35-45 yo	Not elligible	Malay	currently married	None-primary	<USD1195	no	18 yo and below	2
88	2013-2015	35-45 yo	Not elligible	Malay	currently married	Tertiary and above	≥USD1195	no	19-24 yo	1
89	2013-2015	35-45 yo	Not elligible	Chinese	currently married	Secondary	<USD1195	no	19-24 yo	3 and above
90	2013-2015	35-45 yo	Not elligible	Malay	currently married	Secondary	<USD1195	no	18 yo and below	1
91	2013-2015	35-45 yo	Not elligible	Chinese	currently married	Secondary	≥USD1195	no	19-24 yo	2
92	2013-2015	35-45 yo	Not elligible	Chinese	currently married	Secondary	<USD1195	no	19-24 yo	1
93	2013-2015	35-45 yo	Not elligible	Malay	currently married	Secondary	<USD1195	no	19-24 yo	1
94	2013-2015	35-45 yo	Not elligible	Malay	currently married	Secondary	<USD1195	no	19-24 yo	1
95	2013-2015	35-45 yo	Not elligible	Indian	currently married	Tertiary and above	≥USD1195	no	18 yo and below	
96	2013-2015	35-45 yo	Not elligible	Malay	currently married	Tertiary and above	≥USD1195	no	25-29 yo	1
97	2013-2015	35-45 yo	Not elligible	Malay	currently married	Tertiary and above	≥USD1195	no	25-29 yo	1
98	2013-2015	35-45 yo	Not elligible	Indian	currently married	Secondary	<USD1195	no	25-29 yo	1
99	2013-2015	35-45 yo	Not elligible	Malay	currently married	Secondary	<USD1195	no	19-24 yo	1
100	2013-2015	35-45 yo	Not elligible	Malay	currently married	Secondary	<USD1195	no	19-24 yo	1
101	2013-2015	35-45 yo	Not elligible	Other	currently married	Tertiary and above	≥USD1195	no	25-29 yo	1
102	2013-2015	35-45 yo	Not elligible	Malay	currently married	Tertiary and above	≥USD1195	no	19-24 yo	1
103	2013-2015	35-45 yo	Not elligible	Malay	currently married	Tertiary and above	≥USD1195	no	25-29 yo	1
104	2013-2015	35-45 yo	Not elligible	Malay	currently married	Tertiary and above	≥USD1195	no	30 and above	1
105	2013-2015	35-45 yo	Not elligible	Malay	currently married	None-primary	<USD1195	no	25-29 yo	1
106	2013-2015	18-24 yo	Not elligible	Malay	currently married	Tertiary and above	<USD1195	no	19-24 yo	1
107	2013-2015	35-45 yo	Not elligible	Malay	currently married	None-primary	<USD1195	no	18 yo and below	1
108	2013-2015	35-45 yo	Not elligible	Malay	currently married	Secondary	<USD1195	no	19-24 yo	1
109	2013-2015	35-45 yo	Not elligible	Malay	currently married	Secondary	<USD1195	no	19-24 yo	1
110	2013-2015	35-45 yo	Not elligible	Malay	currently married	Tertiary and above	<USD1195	no	25-29 yo	1
111	2013-2015	35-45 yo	Not elligible	Malay	currently married	Secondary	<USD1195	no	19-24 yo	1
112	2013-2015	35-45 yo	Not elligible	Chinese	currently married	Secondary	<USD1195	no	18 yo and below	1
113	2013-2015	35-45 yo	Not elligible	Malay	currently married	Tertiary and above	<USD1195	no	25-29 yo	1
114	2013-2015	35-45 yo	Not elligible	Indian	currently married	Secondary	<USD1195	no	19-24 yo	1
115	2013-2015	35-45 yo	Not elligible	Chinese	currently married	Tertiary and above	≥USD1195	no	19-24 yo	1
116	2013-2015	35-45 yo	Not elligible	Malay	currently married	Tertiary and above	≥USD1195	no	25-29 yo	1
117	2013-2015	35-45 yo	Not elligible	Chinese	currently married	Secondary	≥USD1195	no	18 yo and below	3 and above
118	2013-2015	18-24 yo	Not elligible	Malay	currently married	Secondary	<USD1195	no	18 yo and below	2
119	2013-2015	35-45 yo	Not elligible	Indian	currently married	Secondary	<USD1195	no	19-24 yo	1
120	2013-2015	35-45 yo	Not elligible	Indian	currently married	Secondary	<USD1195	no	19-24 yo	1

121	2013-2015	35-45 yo	Not elligible	Chinese	currently married	Secondary	<USD1195	no	19-24 yo	1
122	2013-2015	18-24 yo	Not elligible	Malay	currently married	Secondary	<USD1195	no	18 yo and below	1
123	2013-2015	35-45 yo	Not elligible	Chinese	currently married	Secondary	<USD1195	no	18 yo and below	3 and above
124	2013-2015	18-24 yo	Not elligible	Malay	currently married	Secondary	<USD1195	no	18 yo and below	1
125	2013-2015	35-45 yo	Not elligible	Chinese	currently married	Secondary	<USD1195	no	19-24 yo	1
126	2013-2015	35-45 yo	Not elligible	Malay	currently married	Tertiary and above	<USD1195	no	25-29 yo	1
127	2013-2015	35-45 yo	Not elligible	Malay	currently married	Secondary	<USD1195	no	25-29 yo	1
128	2013-2015	18-24 yo	Not elligible	Malay	currently married	Secondary	<USD1195	no	19-24 yo	1
129	2013-2015	35-45 yo	Not elligible	Malay	currently married	Secondary	<USD1195	no	30 and above	1
130	2013-2015	35-45 yo	Not elligible	Chinese	currently married	Secondary	<USD1195	no	30 and above	1
131	2013-2015	35-45 yo	Not elligible	Chinese	currently married	Secondary	<USD1195	no	19-24 yo	
132	2013-2015	35-45 yo	Not elligible	Chinese	currently married	Secondary	≥USD1195	no	19-24 yo	1
133	2013-2015	35-45 yo	Not elligible	Malay	currently married	Secondary	<USD1195	no	19-24 yo	1
134	2013-2015	35-45 yo	Not elligible	Malay	currently married	Secondary	<USD1195	no	25-29 yo	1
135	2013-2015	35-45 yo	Not elligible	Indian	currently married	Secondary	<USD1195	no	19-24 yo	1
136	2013-2015	18-24 yo	Not elligible	Chinese	currently married	Secondary	<USD1195	no	18 yo and below	
137	2013-2015	35-45 yo	Not elligible	Malay	currently married	Tertiary and above	<USD1195	no	25-29 yo	1
138	2013-2015	35-45 yo	Not elligible	Malay	currently married	Secondary	<USD1195	yes	25-29 yo	1
139	2013-2015	35-45 yo	Not elligible	Malay	currently married	Tertiary and above	<USD1195	no	25-29 yo	1
140	2013-2015	35-45 yo	Not elligible	Malay	currently married	Tertiary and above	<USD1195	no	25-29 yo	1
141	2013-2015	35-45 yo	Not elligible	Malay	currently married	Tertiary and above	<USD1195	yes	25-29 yo	1
142	2013-2015	35-45 yo	Not elligible	Indian	currently married	Secondary	<USD1195	no	19-24 yo	1
143	2013-2015	18-24 yo	Not elligible	Chinese	single	Tertiary and above	<USD1195	yes	19-24 yo	1
144	2013-2015	35-45 yo	Not elligible	Chinese	currently married	Secondary	<USD1195	no	25-29 yo	1
145	2013-2015	18-24 yo	Not elligible	Chinese	currently married	Secondary	<USD1195	no	18 yo and below	2
146	2013-2015	35-45 yo	Not elligible	Chinese	currently married	Secondary	<USD1195	yes	19-24 yo	1
147	2013-2015	35-45 yo	Not elligible	Chinese	currently married	Secondary	<USD1195	no	19-24 yo	
148	2013-2015	18-24 yo	school-based	Indian	single	Tertiary and above	≥USD1195	yes	18 yo and below	1
149	2013-2015	35-45 yo	Not elligible	Chinese	currently married	Secondary	<USD1195	no	19-24 yo	1
150	2013-2015	35-45 yo	Not elligible	Chinese	currently married	Secondary	<USD1195	yes	18 yo and below	
151	2013-2015	35-45 yo	Not elligible	Malay	currently married	Tertiary and above	<USD1195	no	25-29 yo	1
152	2013-2015	35-45 yo	Not elligible	Indian	currently married	Tertiary and above	≥USD1195	no	25-29 yo	1
153	2013-2015	35-45 yo	Not elligible	Chinese	currently married	Tertiary and above	<USD1195	no	25-29 yo	1
154	2013-2015	35-45 yo	Not elligible	Malay	currently married	Tertiary and above	<USD1195	no	25-29 yo	1
155	2013-2015	35-45 yo	Not elligible	Indian	currently married	Tertiary and above	≥USD1195	no	30 and above	1
156	2013-2015	35-45 yo	Not elligible	Malay	currently married	Tertiary and above	<USD1195	no	25-29 yo	1
157	2013-2015	35-45 yo	Not elligible	Chinese	currently married	Secondary	<USD1195	no	18 yo and below	1
158	2013-2015	35-45 yo	Not elligible	Chinese	currently married	Tertiary and above	≥USD1195	no	25-29 yo	1
159	2013-2015	35-45 yo	Not elligible	Malay	currently married	Secondary	<USD1195	no	19-24 yo	1
160	2013-2015	35-45 yo	Not elligible	Malay	currently married	Secondary	<USD1195	no	19-24 yo	1

161	2013-2015	35-45 yo	Not elligible	Malay	currently married	Secondary	<USD1195	no	19-24 yo	1
162	2013-2015	35-45 yo	Not elligible	Indian	currently married	None-primary	<USD1195	no	19-24 yo	1
163	2013-2015	35-45 yo	Not elligible	Malay	currently married	Secondary	<USD1195	no	19-24 yo	2
164	2013-2015	35-45 yo	Not elligible	Malay	currently married	Tertiary and above	<USD1195	no	19-24 yo	1
165	2013-2015	35-45 yo	Not elligible	Malay	currently married	Secondary	<USD1195	no	19-24 yo	1
166	2013-2015	35-45 yo	Not elligible	Malay	currently married	Secondary	<USD1195	no	30 and above	1
167	2013-2015	35-45 yo	Not elligible	Malay	currently married	Secondary	<USD1195	no	19-24 yo	1
168	2013-2015	35-45 yo	Not elligible	Indian	currently married	Secondary	<USD1195	no	19-24 yo	1
169	2013-2015	35-45 yo	Not elligible	Malay	currently married	Secondary	<USD1195	no	19-24 yo	1
170	2013-2015	35-45 yo	Not elligible	Indian	currently married	None-primary	<USD1195	no	18 yo and below	1
171	2013-2015	35-45 yo	Not elligible	Malay	currently married	Tertiary and above	≥USD1195	no	25-29 yo	1
172	2013-2015	35-45 yo	Not elligible	Indian	currently married	Secondary	<USD1195	no	19-24 yo	1
173	2013-2015	35-45 yo	Not elligible	Malay	currently married	Secondary	<USD1195	no	19-24 yo	2
174	2013-2015	35-45 yo	Not elligible	Indian	currently married	Secondary	<USD1195	no	18 yo and below	1
175	2013-2015	35-45 yo	Not elligible	Indian	currently married	Secondary	<USD1195	no	30 and above	2
176	2013-2015	18-24 yo	Not elligible	Indian	currently married	Secondary	<USD1195	no	19-24 yo	1
178	2013-2015	35-45 yo	Not elligible	Malay	currently married	Secondary	<USD1195	no	19-24 yo	1
179	2013-2015	35-45 yo	Not elligible	Chinese	currently married	None-primary	<USD1195	no	18 yo and below	
180	2013-2015	35-45 yo	Not elligible	Malay	currently married	Secondary	<USD1195	no	19-24 yo	1
181	2013-2015	35-45 yo	Not elligible	Malay	currently married	Secondary	<USD1195	no	19-24 yo	1
182	2013-2015	35-45 yo	Not elligible	Malay	currently married	Secondary	<USD1195	no	18 yo and below	1
183	2013-2015	35-45 yo	Not elligible	Other	currently married	Secondary	<USD1195	no	19-24 yo	2
184	2013-2015	18-24 yo	Not elligible	Indian	currently married	None-primary	<USD1195	no	19-24 yo	1
185	2013-2015	18-24 yo	Not elligible	Indian	currently married	Secondary	<USD1195	no	18 yo and below	1
186	2013-2015	35-45 yo	Not elligible	Indian	currently married	Tertiary and above	<USD1195	no	25-29 yo	1
187	2013-2015	35-45 yo	Not elligible	Malay	currently married	Secondary	<USD1195	no	19-24 yo	1
188	2013-2015	35-45 yo	Not elligible	Malay	currently married	Tertiary and above	<USD1195	no	25-29 yo	1
189	2013-2015	35-45 yo	Not elligible	Indian	currently married	Secondary	<USD1195	no	25-29 yo	1
190	2013-2015	35-45 yo	Not elligible	Indian	currently married	Secondary	<USD1195	yes	25-29 yo	1
191	2013-2015	35-45 yo	Not elligible	Indian	currently married	Secondary	<USD1195	no	19-24 yo	1
192	2013-2015	35-45 yo	Not elligible	Indian	currently married	Secondary	<USD1195	no	19-24 yo	1
193	2013-2015	35-45 yo	Not elligible	Indian	currently married	Secondary	<USD1195	no	25-29 yo	1
194	2013-2015	18-24 yo	Not elligible	Other	currently married	None-primary	<USD1195	no	19-24 yo	1
195	2013-2015	35-45 yo	Not elligible	Malay	currently married	None-primary	<USD1195	no	25-29 yo	1
196	2013-2015	35-45 yo	Not elligible	Malay	currently married	Secondary	<USD1195	no	19-24 yo	1
197	2013-2015	35-45 yo	Not elligible	Malay	currently married	None-primary	<USD1195	no	18 yo and below	1
198	2013-2015	35-45 yo	Not elligible	Indian	currently married	Secondary	<USD1195	no	25-29 yo	1
200	2013-2015	18-24 yo	Not elligible	Malay	currently married	Secondary	<USD1195	no	18 yo and below	2
201	2013-2015	35-45 yo	Not elligible	Malay	currently married	Secondary	<USD1195	no	19-24 yo	1
203	2013-2015	35-45 yo	Not elligible	Chinese	currently married	None-primary	<USD1195	no	19-24 yo	1

204	2013-2015	18-24 yo	Not elligible	Indian	single	Secondary	<USD1195	no	18 yo and below	1
205	2013-2015	35-45 yo	Not elligible	Indian	currently married	Secondary	<USD1195	no	30 and above	1
206	2013-2015	35-45 yo	Not elligible	Malay	currently married	Secondary	<USD1195	no	30 and above	1
207	2013-2015	35-45 yo	Not elligible	Indian	currently married	Secondary	≥USD1195	no	19-24 yo	1
208	2013-2015	35-45 yo	Not elligible	Malay	currently married	None-primary	<USD1195	no	19-24 yo	1
209	2013-2015	35-45 yo	Not elligible	Chinese	currently married	Tertiary and above	≥USD1195	no	25-29 yo	1
210	2013-2015	35-45 yo	Not elligible	Indian	currently married	None-primary	<USD1195	no	19-24 yo	1
211	2013-2015	18-24 yo	Not elligible	Malay	currently married	Secondary	<USD1195	no	19-24 yo	1
212	2013-2015	18-24 yo	Not elligible	Malay	currently married	Secondary	<USD1195	no	18 yo and below	1
213	2013-2015	18-24 yo	Not elligible	Malay	currently married	Secondary	<USD1195	no	18 yo and below	1
214	2013-2015	18-24 yo	Not elligible	Malay	currently married	Secondary	<USD1195	yes	19-24 yo	1
215	2013-2015	35-45 yo	Not elligible	Chinese	currently married	Secondary	<USD1195	no	25-29 yo	1
216	2013-2015	35-45 yo	Not elligible	Indian	currently married	Secondary	<USD1195	no	30 and above	1
217	2013-2015	18-24 yo	Not elligible	Malay	single	Secondary	<USD1195	no	18 yo and below	1
218	2013-2015	18-24 yo	Not elligible	Malay	currently married	Secondary	<USD1195	no	19-24 yo	1
219	2013-2015	18-24 yo	Not elligible	Indian	single	Tertiary and above	<USD1195	no	19-24 yo	2
220	2013-2015	18-24 yo	Not elligible	Indian	single	Secondary	<USD1195	no	18 yo and below	1
221	2013-2015	35-45 yo	Not elligible	Indian	currently married	Secondary	<USD1195	no	25-29 yo	1
222	2013-2015	35-45 yo	Not elligible	Indian	currently married	None-primary	<USD1195	no	19-24 yo	1
223	2013-2015	35-45 yo	Not elligible	Malay	currently married	Secondary	<USD1195	no	25-29 yo	1
224	2013-2015	35-45 yo	Not elligible	Malay	currently married	Secondary	<USD1195	no	25-29 yo	1
225	2013-2015	18-24 yo	Not elligible	Indian	currently married	None-primary	<USD1195	no	18 yo and below	1
226	2013-2015	35-45 yo	Not elligible	Indian	currently married	Secondary	<USD1195	no	19-24 yo	1
227	2013-2015	18-24 yo	Not elligible	Indian	currently married	Secondary	<USD1195	no	19-24 yo	1
228	2013-2015	35-45 yo	Not elligible	Malay	currently married	Secondary	<USD1195	no	25-29 yo	1
229	2013-2015	35-45 yo	Not elligible	Malay	currently married	Secondary	<USD1195	no	19-24 yo	1
230	2013-2015	35-45 yo	Not elligible	Chinese	currently married	Secondary	<USD1195	no	19-24 yo	1
231	2013-2015	18-24 yo	Not elligible	Malay	currently married	Secondary	<USD1195	no	18 yo and below	1
232	2013-2015	35-45 yo	Not elligible	Indian	currently married	Secondary	<USD1195	no	25-29 yo	1
233	2013-2015	35-45 yo	Not elligible	Indian	currently married	Secondary	<USD1195	no	25-29 yo	1
234	2013-2015	35-45 yo	Not elligible	Indian	currently married	Secondary	<USD1195	no	19-24 yo	1
235	2013-2015	35-45 yo	Not elligible	Indian	currently married	Tertiary and above	≥USD1195	no	25-29 yo	1
236	2013-2015	35-45 yo	Not elligible	Malay	currently married	Secondary	<USD1195	no	19-24 yo	1
237	2013-2015	35-45 yo	Not elligible	Malay	currently married	Secondary	<USD1195	no	19-24 yo	2
238	2013-2015	35-45 yo	Not elligible	Indian	currently married	Secondary	<USD1195	no	19-24 yo	1
239	2013-2015	18-24 yo	Not elligible	Indian	currently married	None-primary	<USD1195	no	18 yo and below	1
240	2013-2015	35-45 yo	Not elligible	Malay	currently married	Secondary	<USD1195	no	19-24 yo	1
241	2013-2015	35-45 yo	Not elligible	Malay	currently married	Secondary	<USD1195	no	19-24 yo	1
242	2013-2015	35-45 yo	Not elligible	Other	currently married	Secondary	<USD1195	no	18 yo and below	
243	2013-2015	35-45 yo	Not elligible	Chinese	currently married	Secondary	<USD1195	no	25-29 yo	1

244	2013-2015	35-45 yo	Not elligible	Chinese	currently married	Secondary	<USD1195	no	19-24 yo	1
245	2013-2015	35-45 yo	Not elligible	Indian	currently married	None-primary	<USD1195	no	25-29 yo	1
246	2013-2015	35-45 yo	Not elligible	Malay	currently married	Tertiary and above	<USD1195	no	19-24 yo	1
247	2013-2015	35-45 yo	Not elligible	Malay	currently married	Secondary	<USD1195	no	19-24 yo	1
248	2013-2015	35-45 yo	Not elligible	Malay	currently married	Tertiary and above	<USD1195	no	25-29 yo	1
249	2013-2015	35-45 yo	Not elligible	Chinese	currently married	Tertiary and above	≥USD1195	no	19-24 yo	2
250	2013-2015	35-45 yo	Not elligible	Malay	currently married	Tertiary and above	<USD1195	no	25-29 yo	1
251	2013-2015	35-45 yo	Not elligible	Malay	currently married	Secondary	<USD1195	no	30 and above	1
252	2013-2015	35-45 yo	Not elligible	Indian	currently married	Secondary	<USD1195	no	19-24 yo	1
253	2013-2015	35-45 yo	Not elligible	Indian	currently married	Secondary	<USD1195	no	19-24 yo	1
254	2013-2015	35-45 yo	Not elligible	Indian	currently married	Secondary	<USD1195	no	19-24 yo	1
255	2013-2015	35-45 yo	Not elligible	Indian	currently married	Secondary	<USD1195	no	19-24 yo	1
256	2013-2015	35-45 yo	Not elligible	Malay	currently married	Tertiary and above	≥USD1195	yes	19-24 yo	1
257	2013-2015	35-45 yo	Not elligible	Indian	currently married	Tertiary and above	≥USD1195	no	25-29 yo	1
258	2013-2015	35-45 yo	Not elligible	Indian	currently married	None-primary	<USD1195	no	18 yo and below	1
259	2013-2015	18-24 yo	Not elligible	Chinese	separated/divorced/w	Secondary	<USD1195	no	18 yo and below	3 and above
260	2013-2015	35-45 yo	Not elligible	Chinese	currently married	Secondary	<USD1195	no	25-29 yo	1
261	2013-2015	18-24 yo	Not elligible	Indian	single	Secondary	<USD1195	no	19-24 yo	1
262	2013-2015	35-45 yo	Not elligible	Malay	currently married	Tertiary and above	<USD1195	no	25-29 yo	1
263	2013-2015	35-45 yo	Not elligible	Malay	currently married	Tertiary and above	≥USD1195	no	19-24 yo	2
264	2013-2015	35-45 yo	Not elligible	Indian	currently married	None-primary	<USD1195	no	19-24 yo	1
265	2013-2015	35-45 yo	Not elligible	Indian	currently married	Secondary	<USD1195	no	19-24 yo	1
266	2013-2015	18-24 yo	Not elligible	Malay	currently married	Tertiary and above	<USD1195	no	19-24 yo	1
267	2013-2015	35-45 yo	Not elligible	Chinese	currently married	Tertiary and above	≥USD1195	no	25-29 yo	2
268	2013-2015	35-45 yo	Not elligible	Malay	currently married	Secondary	<USD1195	no	19-24 yo	1
269	2013-2015	35-45 yo	Not elligible	Malay	currently married	Secondary	≥USD1195	no	25-29 yo	1
270	2013-2015	35-45 yo	Not elligible	Malay	separated/divorced/w	Secondary	<USD1195	no	19-24 yo	1
271	2013-2015	35-45 yo	Not elligible	Indian	currently married	Secondary	<USD1195	no	19-24 yo	1
272	2013-2015	35-45 yo	Not elligible	Malay	currently married	Tertiary and above	<USD1195	no	19-24 yo	1
273	2013-2015	35-45 yo	Not elligible	Malay	currently married	None-primary	<USD1195	no	19-24 yo	1
274	2013-2015	35-45 yo	Not elligible	Chinese	currently married	Secondary	<USD1195	no	18 yo and below	3 and above
275	2013-2015	35-45 yo	Not elligible	Malay	currently married	Tertiary and above	<USD1195	no	25-29 yo	1
276	2013-2015	35-45 yo	Not elligible	Chinese	currently married	Tertiary and above	≥USD1195	no	30 and above	1
277	2013-2015	35-45 yo	Not elligible	Malay	currently married	Tertiary and above	≥USD1195	no	25-29 yo	1
278	2013-2015	35-45 yo	Not elligible	Malay	currently married	Tertiary and above	≥USD1195	no	25-29 yo	1
279	2013-2015	35-45 yo	Not elligible	Malay	currently married	Tertiary and above	<USD1195	no	25-29 yo	1
280	2013-2015	35-45 yo	Not elligible	Malay	currently married	Secondary	<USD1195	no	25-29 yo	1
281	2013-2015	35-45 yo	Not elligible	Malay	currently married	Tertiary and above	<USD1195	no	25-29 yo	1
282	2013-2015	35-45 yo	Not elligible	Malay	currently married	Tertiary and above	<USD1195	no	19-24 yo	1
283	2013-2015	35-45 yo	Not elligible	Malay	currently married	Tertiary and above	<USD1195	no	19-24 yo	1

284	2013-2015	35-45 yo	Not elligible	Malay	currently married	Tertiary and above	<USD1195	no	18 yo and below	3 and above
285	2013-2015	35-45 yo	Not elligible	Chinese	separated/divorced/w	None-primary	<USD1195	no	19-24 yo	3 and above
286	2013-2015	35-45 yo	Not elligible	Indian	currently married	Secondary	<USD1195	no	30 and above	1
287	2013-2015	35-45 yo	Not elligible	Malay	currently married	Tertiary and above	≥USD1195	no	25-29 yo	1
288	2013-2015	35-45 yo	Not elligible	Chinese	single	Tertiary and above	<USD1195	yes	19-24 yo	3 and above
289	2013-2015	18-24 yo	Not elligible	Malay	single	Tertiary and above	<USD1195	no	19-24 yo	3 and above
290	2013-2015	35-45 yo	Not elligible	Indian	currently married	None-primary	<USD1195	no	18 yo and below	1
291	2013-2015	35-45 yo	Not elligible	Indian	currently married	Secondary	<USD1195	no	19-24 yo	1
292	2013-2015	35-45 yo	Not elligible	Malay	currently married	Secondary	<USD1195	no	25-29 yo	1
293	2013-2015	18-24 yo	Not elligible	Indian	currently married	None-primary	<USD1195	no		
294	2013-2015	35-45 yo	Not elligible	Malay	currently married	Secondary	<USD1195	no	19-24 yo	1
295	2013-2015	18-24 yo	Not elligible	Malay	currently married	Secondary	<USD1195	no	18 yo and below	1
296	2013-2015	18-24 yo	Not elligible	Malay	currently married	None-primary	<USD1195	no	18 yo and below	2
297	2013-2015	18-24 yo	Not elligible	Malay	currently married	Secondary	<USD1195	no	19-24 yo	1
298	2013-2015	35-45 yo	Not elligible	Indian	currently married	Secondary	<USD1195	no	19-24 yo	2
299	2013-2015	35-45 yo	Not elligible	Chinese	currently married	Secondary	<USD1195	no	19-24 yo	1
300	2013-2015	35-45 yo	Not elligible	Chinese	currently married	Tertiary and above	≥USD1195	no	25-29 yo	2
301	2013-2015	35-45 yo	Not elligible	Indian	currently married	None-primary	<USD1195	no	19-24 yo	1
302	2013-2015	18-24 yo	Not elligible	Malay	currently married	None-primary	<USD1195	no	18 yo and below	3 and above
303	2013-2015	35-45 yo	Not elligible	Malay	currently married	Secondary	<USD1195	no	19-24 yo	1
304	2013-2015	35-45 yo	Not elligible	Malay	currently married	Secondary	<USD1195	no	19-24 yo	1
305	2013-2015	35-45 yo	Not elligible	Other	currently married	Tertiary and above	≥USD1195	no	19-24 yo	1
306	2013-2015	35-45 yo	Not elligible	Indian	currently married	None-primary	<USD1195	no	18 yo and below	1
307	2013-2015	35-45 yo	Not elligible	Chinese	currently married	None-primary	<USD1195	no	18 yo and below	
308	2013-2015	35-45 yo	Not elligible	Chinese	currently married	Secondary	<USD1195	no	18 yo and below	1
309	2013-2015	18-24 yo	Not elligible	Malay	currently married	Secondary	<USD1195	no	19-24 yo	1
310	2013-2015	35-45 yo	Not elligible	Malay	currently married	Tertiary and above	≥USD1195	no	25-29 yo	1
311	2013-2015	35-45 yo	Not elligible	Indian	currently married	Tertiary and above	≥USD1195	no	25-29 yo	1
312	2013-2015	35-45 yo	Not elligible	Malay	currently married	Secondary	<USD1195	no	25-29 yo	1
313	2013-2015	35-45 yo	Not elligible	Malay	currently married	Secondary	<USD1195	no	25-29 yo	1
314	2013-2015	35-45 yo	Not elligible	Malay	currently married	Tertiary and above	≥USD1195	no	25-29 yo	1
315	2013-2015	35-45 yo	Not elligible	Chinese	currently married	Secondary	≥USD1195	no	19-24 yo	1
316	2013-2015	35-45 yo	Not elligible	Malay	currently married	Tertiary and above	<USD1195	no	25-29 yo	1
317	2013-2015	18-24 yo	Not elligible	Indian	currently married	Secondary	<USD1195	no	19-24 yo	1
318	2013-2015	35-45 yo	Not elligible	Malay	currently married	Secondary	<USD1195	no	18 yo and below	3 and above
319	2013-2015	35-45 yo	Not elligible	Malay	currently married	Secondary	<USD1195	no	19-24 yo	1
320	2013-2015	18-24 yo	Not elligible	Other	currently married	None-primary	<USD1195	no	19-24 yo	1
321	2013-2015	35-45 yo	Not elligible	Indian	currently married	None-primary	<USD1195	no	18 yo and below	1
322	2013-2015	35-45 yo	Not elligible	Malay	currently married	Tertiary and above	≥USD1195	no	19-24 yo	1
323	2013-2015	35-45 yo	Not elligible	Indian	currently married	None-primary	<USD1195	no	18 yo and below	1

324	2013-2015	35-45 yo	Not elligible	Indian	currently married	Secondary	<USD1195	no	19-24 yo	1
325	2013-2015	35-45 yo	Not elligible	Malay	currently married	Secondary	<USD1195	no	19-24 yo	1
326	2013-2015	35-45 yo	Not elligible	Malay	currently married	Tertiary and above	≥USD1195	no	19-24 yo	1
327	2013-2015	35-45 yo	Not elligible	Chinese	currently married	Secondary	<USD1195	no	18 yo and below	1
328	2013-2015	35-45 yo	Not elligible	Malay	currently married	Secondary	<USD1195	no	19-24 yo	3 and above
329	2013-2015	35-45 yo	Not elligible	Indian	currently married	None-primary	<USD1195	no	25-29 yo	1
330	2013-2015	35-45 yo	Not elligible	Chinese	currently married	Secondary	≥USD1195	no	25-29 yo	1
331	2013-2015	35-45 yo	Not elligible	Chinese	currently married	Secondary	<USD1195	no	19-24 yo	3 and above
332	2013-2015	35-45 yo	Not elligible	Chinese	currently married	Tertiary and above	<USD1195	no	18 yo and below	3 and above
333	2013-2015	35-45 yo	Not elligible	Malay	currently married	Secondary	≥USD1195	no	19-24 yo	1
334	2013-2015	35-45 yo	Not elligible	Chinese	currently married	Secondary	<USD1195	no	19-24 yo	2
335	2013-2015	18-24 yo	Not elligible	Malay	currently married	Tertiary and above	<USD1195	no	19-24 yo	1
336	2013-2015	35-45 yo	Not elligible	Malay	currently married	Tertiary and above	≥USD1195	no	25-29 yo	1
337	2013-2015	35-45 yo	Not elligible	Indian	currently married	Secondary	<USD1195	no	19-24 yo	1
338	2013-2015	35-45 yo	Not elligible	Malay	currently married	Secondary	<USD1195	no	25-29 yo	1
339	2013-2015	35-45 yo	Not elligible	Chinese	currently married	Secondary	<USD1195	no	19-24 yo	2
340	2013-2015	18-24 yo	Not elligible	Malay	single	Tertiary and above	<USD1195	yes	18 yo and below	3 and above
341	2013-2015	35-45 yo	Not elligible	Chinese	currently married	Secondary	<USD1195	no	25-29 yo	1
342	2013-2015	35-45 yo	Not elligible	Malay	currently married	Secondary	≥USD1195	no	19-24 yo	1
343	2013-2015	35-45 yo	Not elligible	Malay	currently married	Secondary	<USD1195	no	18 yo and below	1
344	2013-2015	35-45 yo	Not elligible	Malay	currently married	Tertiary and above	<USD1195	no	25-29 yo	1
345	2013-2015	35-45 yo	Not elligible	Indian	currently married	None-primary	<USD1195	no	18 yo and below	2
346	2013-2015	35-45 yo	Not elligible	Malay	currently married	Secondary	<USD1195	no	19-24 yo	2
347	2013-2015	35-45 yo	Not elligible	Malay	currently married	Tertiary and above	≥USD1195	no	25-29 yo	1
348	2013-2015	35-45 yo	Not elligible	Malay	currently married	Tertiary and above	≥USD1195	no	25-29 yo	1
349	2013-2015	18-24 yo	Not elligible	Chinese	currently married	Tertiary and above	<USD1195	no		
350	2013-2015	35-45 yo	Not elligible	Malay	currently married	Secondary	<USD1195	no	18 yo and below	1
351	2013-2015	35-45 yo	Not elligible	Malay	currently married	Tertiary and above	≥USD1195	no	19-24 yo	1
352	2013-2015	35-45 yo	Not elligible	Indian	currently married	Tertiary and above	<USD1195	no	30 and above	1
353	2013-2015	35-45 yo	Not elligible	Malay	currently married	Tertiary and above	≥USD1195	no	25-29 yo	1
354	2013-2015	35-45 yo	Not elligible	Chinese	currently married	Secondary	<USD1195	no	18 yo and below	2
355	2013-2015	35-45 yo	Not elligible	Indian	currently married	Secondary	<USD1195	no	19-24 yo	2
356	2013-2015	35-45 yo	Not elligible	Indian	currently married	Secondary	<USD1195	no	25-29 yo	1
357	2013-2015	35-45 yo	Not elligible	Indian	currently married	None-primary	<USD1195	no	19-24 yo	1
358	2013-2015	35-45 yo	Not elligible	Indian	currently married	Secondary	<USD1195	no	19-24 yo	1
359	2013-2015	35-45 yo	Not elligible	Malay	currently married	Secondary	<USD1195	no	19-24 yo	2
360	2013-2015	35-45 yo	Not elligible	Indian	currently married	Tertiary and above	<USD1195	no	30 and above	1
361	2013-2015	35-45 yo	Not elligible	Indian	currently married	None-primary	<USD1195	no	25-29 yo	1
362	2013-2015	35-45 yo	Not elligible	Malay	currently married	Secondary	<USD1195	no	19-24 yo	1
363	2013-2015	18-24 yo	Not elligible	Indian	currently married	Tertiary and above	<USD1195	yes	19-24 yo	1

364	2013-2015	35-45 yo	Not elligible	Chinese	single	Secondary	<USD1195	no	19-24 yo	3 and above
365	2013-2015	35-45 yo	Not elligible	Indian	currently married	Secondary	<USD1195	no	25-29 yo	1
366	2013-2015	35-45 yo	Not elligible	Chinese	single	Secondary	<USD1195	no	19-24 yo	3 and above
367	2013-2015	35-45 yo	Not elligible	Indian	currently married	Secondary	<USD1195	no	25-29 yo	2
368	2013-2015	35-45 yo	Not elligible	Indian	currently married	Secondary	<USD1195	no	25-29 yo	1
369	2013-2015	18-24 yo	Not elligible	Chinese	single	Tertiary and above	<USD1195	no	19-24 yo	1
370	2013-2015	35-45 yo	Not elligible	Malay	currently married	Secondary	<USD1195	no	19-24 yo	1
371	2013-2015	35-45 yo	Not elligible	Malay	currently married	Secondary	<USD1195	no	19-24 yo	1
372	2013-2015	35-45 yo	Not elligible	Malay	currently married	Tertiary and above	<USD1195	no	19-24 yo	1
373	2013-2015	35-45 yo	Not elligible	Chinese	currently married	Tertiary and above	≥USD1195	no	19-24 yo	1
374	2013-2015	35-45 yo	Not elligible	Malay	currently married	Secondary	<USD1195	no	25-29 yo	1
375	2013-2015	35-45 yo	Not elligible	Chinese	currently married	None-primary	<USD1195	no	19-24 yo	1
376	2013-2015	35-45 yo	Not elligible	Malay	currently married	Tertiary and above	≥USD1195	no	25-29 yo	1
377	2013-2015	35-45 yo	Not elligible	Malay	currently married	Secondary	<USD1195	no	19-24 yo	1
378	2013-2015	35-45 yo	Not elligible	Malay	currently married	Secondary	<USD1195	no	19-24 yo	1
379	2013-2015	35-45 yo	Not elligible	Chinese	currently married	Tertiary and above	≥USD1195	no	25-29 yo	1
380	2013-2015	35-45 yo	Not elligible	Indian	currently married	Tertiary and above	<USD1195	no	30 and above	1
381	2013-2015	35-45 yo	Not elligible	Malay	currently married	None-primary	<USD1195	no	30 and above	1
382	2013-2015	35-45 yo	Not elligible	Malay	currently married	Secondary	<USD1195	no	25-29 yo	1
383	2013-2015	35-45 yo	Not elligible	Indian	currently married	None-primary	<USD1195	no	19-24 yo	1
384	2013-2015	35-45 yo	Not elligible	Chinese	single	Tertiary and above	≥USD1195	no	19-24 yo	3 and above
385	2013-2015	35-45 yo	Not elligible	Malay	separated/divorced/w	Secondary	<USD1195	no	19-24 yo	2
386	2013-2015	18-24 yo	Not elligible	Indian	separated/divorced/w	Secondary	<USD1195	no	18 yo and below	1
387	2013-2015	35-45 yo	Not elligible	Indian	currently married	Secondary	≥USD1195	no	19-24 yo	1
388	2013-2015	18-24 yo	Not elligible	Malay	single	Secondary	<USD1195	no	18 yo and below	3 and above
389	2013-2015	35-45 yo	Not elligible	Malay	separated/divorced/w	Secondary	<USD1195	no	19-24 yo	1
390	2013-2015	35-45 yo	Not elligible	Malay	currently married	Tertiary and above	<USD1195	no	25-29 yo	1
391	2013-2015	35-45 yo	Not elligible	Indian	separated/divorced/w	Tertiary and above	<USD1195	no	25-29 yo	1
392	2013-2015	35-45 yo	Not elligible	Indian	currently married	Secondary	<USD1195	no	18 yo and below	1
393	2013-2015	35-45 yo	Not elligible	Indian	separated/divorced/w	None-primary	<USD1195	no	18 yo and below	1
394	2013-2015	35-45 yo	Not elligible	Malay	separated/divorced/w	Secondary	<USD1195	no	25-29 yo	1
395	2013-2015	18-24 yo	Not elligible	Malay	currently married	Tertiary and above	<USD1195	no	19-24 yo	1
396	2013-2015	35-45 yo	Not elligible	Chinese	separated/divorced/w	Secondary	<USD1195	no	19-24 yo	1
397	2013-2015	35-45 yo	Not elligible	Malay	separated/divorced/w	Tertiary and above	<USD1195	no	19-24 yo	1
398	2013-2015	35-45 yo	Not elligible	Chinese	separated/divorced/w	Secondary	<USD1195	no	18 yo and below	2
399	2013-2015	35-45 yo	Not elligible	Malay	separated/divorced/w	Secondary	<USD1195	no	19-24 yo	2
400	2013-2015	35-45 yo	Not elligible	Malay	separated/divorced/w	Tertiary and above	<USD1195	no	19-24 yo	1
401	2013-2015	35-45 yo	Not elligible	Malay	currently married	Tertiary and above	<USD1195	no	25-29 yo	1
402	2013-2015	35-45 yo	Not elligible	Chinese	separated/divorced/w	Secondary	<USD1195	no	25-29 yo	1
403	2013-2015	35-45 yo	Not elligible	Malay	separated/divorced/w	Secondary	<USD1195	no	19-24 yo	1

404	2013-2015	35-45 yo	Not elligible	Indian	currently married	Secondary	<USD1195	no	25-29 yo	1
405	2013-2015	35-45 yo	Not elligible	Indian	currently married	Secondary	<USD1195	no	25-29 yo	1
406	2013-2015	35-45 yo	Not elligible	Indian	currently married	Tertiary and above	<USD1195	no	30 and above	1
407	2013-2015	18-24 yo	Not elligible	Indian	single	Secondary	<USD1195	no	18 yo and below	1
408	2013-2015	35-45 yo	Not elligible	Indian	separated/divorced/w	None-primary	<USD1195	no	19-24 yo	1
409	2013-2015	18-24 yo	Not elligible	Chinese	single	Secondary	<USD1195	yes	19-24 yo	1
411	2013-2015	35-45 yo	Not elligible	Indian	currently married	Tertiary and above	<USD1195	no	19-24 yo	1
412	2013-2015	35-45 yo	Not elligible	Malay	separated/divorced/w	Secondary	<USD1195	no	25-29 yo	1
413	2013-2015	35-45 yo	Not elligible	Indian	separated/divorced/w	Secondary	<USD1195	no	19-24 yo	1
414	2013-2015	35-45 yo	Not elligible	Indian	currently married	Secondary	<USD1195	no	25-29 yo	1
416	2013-2015	18-24 yo	Not elligible	Indian	single	Secondary	<USD1195	no	19-24 yo	1
417	2013-2015	35-45 yo	Not elligible	Indian	separated/divorced/w	Secondary	<USD1195	no	19-24 yo	
418	2013-2015	18-24 yo	Not elligible	Malay	separated/divorced/w	Secondary	<USD1195	no	18 yo and below	1
420	2013-2015	35-45 yo	Not elligible	Indian	currently married	Secondary	<USD1195	no	30 and above	1
421	2013-2015	18-24 yo	Not elligible	Malay	single	Tertiary and above	<USD1195	no	19-24 yo	1
422	2013-2015	35-45 yo	Not elligible	Malay	currently married	Secondary	<USD1195	no	30 and above	1
423	2013-2015	18-24 yo	Not elligible	Malay	single	Secondary	<USD1195	no	18 yo and below	2
424	2013-2015	35-45 yo	Not elligible	Chinese	single	Tertiary and above	≥USD1195	no	19-24 yo	1
425	2013-2015	35-45 yo	Not elligible	Malay	separated/divorced/w	Secondary	<USD1195	no	19-24 yo	1
426	2013-2015	35-45 yo	Not elligible	Indian	currently married	Secondary	<USD1195	no	19-24 yo	1
427	2013-2015	35-45 yo	Not elligible	Chinese	currently married	Secondary	<USD1195	no	30 and above	
428	2013-2015	35-45 yo	Not elligible	Malay	separated/divorced/w	Secondary	<USD1195	no	30 and above	
429	2013-2015	35-45 yo	Not elligible	Chinese	currently married	Secondary	<USD1195	no	30 and above	
430	2013-2015	35-45 yo	Not elligible	Chinese	currently married	Secondary	<USD1195	no	30 and above	
431	2013-2015	35-45 yo	Not elligible	Indian	currently married	Secondary	<USD1195	no	30 and above	
432	2013-2015	35-45 yo	Not elligible	Indian	currently married	Secondary	<USD1195	no	30 and above	
433	2013-2015	35-45 yo	Not elligible	Malay	separated/divorced/w	Tertiary and above	<USD1195	no	25-29 yo	1
434	2013-2015	18-24 yo	Not elligible	Malay	single	Secondary	<USD1195	no	18 yo and below	1
435	2013-2015	35-45 yo	Not elligible	Chinese	separated/divorced/w	Secondary	<USD1195	no	19-24 yo	2
436	2019-2021	35-45 yo	Not elligible	Chinese	currently married	Secondary	≥USD1195	no	18 yo and below	3 and above
437	2019-2021	35-45 yo	Not elligible	Indian	currently married	Secondary	<USD1195	no	25-29 yo	1
438	2019-2021	35-45 yo	Not elligible	Malay	currently married	Tertiary and above	≥USD1195	no	30 and above	1
439	2019-2021	35-45 yo	Not elligible	Malay	separated/divorced/w	Secondary	<USD1195	no	19-24 yo	2
440	2019-2021	35-45 yo	Not elligible	Malay	currently married	Tertiary and above	≥USD1195	yes	18 yo and below	2
441	2019-2021	35-45 yo	Not elligible	Chinese	separated/divorced/w	Tertiary and above	≥USD1195	no	30 and above	1
442	2019-2021	35-45 yo	Not elligible	Malay	currently married	Secondary	<USD1195	no	30 and above	1
443	2019-2021	35-45 yo	Not elligible	Malay	currently married	Secondary	≥USD1195	no	19-24 yo	1
444	2019-2021	35-45 yo	Not elligible	Malay	currently married	Tertiary and above	≥USD1195	no	19-24 yo	1
445	2019-2021	18-24 yo	catchup	Malay	currently married	Secondary	<USD1195	yes	19-24 yo	1
446	2019-2021	35-45 yo	Not elligible	Malay	currently married	Tertiary and above	≥USD1195	no	25-29 yo	1

447	2019-2021	35-45 yo	Not elligible	Malay	currently married	Tertiary and above	≥USD1195	yes	19-24 yo	1
448	2019-2021	35-45 yo	Not elligible	Chinese	single	Tertiary and above	<USD1195	yes	30 and above	3 and above
449	2019-2021	35-45 yo	Not elligible	Malay	currently married	Tertiary and above	<USD1195	no	25-29 yo	1
450	2019-2021	35-45 yo	Not elligible	Malay	currently married	Tertiary and above	≥USD1195	no	25-29 yo	1
451	2019-2021	35-45 yo	Not elligible	Malay	currently married	Tertiary and above	≥USD1195	no	25-29 yo	1
452	2019-2021	35-45 yo	Not elligible	Malay	currently married	Tertiary and above	≥USD1195	no	19-24 yo	1
453	2019-2021	35-45 yo	Not elligible	Malay	currently married	Tertiary and above	≥USD1195	no	19-24 yo	1
454	2019-2021	35-45 yo	Not elligible	Malay	currently married	Secondary	≥USD1195	no	19-24 yo	1
455	2019-2021	35-45 yo	Not elligible	Malay	single	Tertiary and above	≥USD1195	no	30 and above	1
456	2019-2021	35-45 yo	Not elligible	Malay	currently married	Tertiary and above	≥USD1195	yes	25-29 yo	1
457	2019-2021	35-45 yo	Not elligible	Malay	currently married	Secondary	≥USD1195	no	19-24 yo	1
458	2019-2021	35-45 yo	Not elligible	Chinese	currently married	Tertiary and above	≥USD1195	no	25-29 yo	1
459	2019-2021	35-45 yo	Not elligible	Chinese	currently married	Tertiary and above	<USD1195	no	25-29 yo	3 and above
460	2019-2021	35-45 yo	Not elligible	Malay	currently married	Secondary	≥USD1195	no	30 and above	2
461	2019-2021	35-45 yo	Not elligible	Indian	currently married	Secondary	<USD1195	no	25-29 yo	1
462	2019-2021	35-45 yo	Not elligible	Indian	currently married	Secondary	<USD1195	no	25-29 yo	1
463	2019-2021	35-45 yo	Not elligible	Malay	currently married	Tertiary and above	≥USD1195	no	25-29 yo	1
464	2019-2021	35-45 yo	Not elligible	Malay	currently married	Secondary	≥USD1195	no	25-29 yo	1
465	2019-2021	35-45 yo	Not elligible	Malay	currently married	Secondary	<USD1195	no	19-24 yo	2
466	2019-2021	35-45 yo	Not elligible	Malay	currently married	Tertiary and above	<USD1195	no	25-29 yo	1
467	2019-2021	35-45 yo	Not elligible	Malay	currently married	Secondary	<USD1195	no	19-24 yo	1
468	2019-2021	35-45 yo	Not elligible	Malay	separated/divorced/w	Tertiary and above	≥USD1195	no	25-29 yo	1
469	2019-2021	35-45 yo	Not elligible	Malay	currently married	Tertiary and above	≥USD1195	no	30 and above	1
470	2019-2021	35-45 yo	Not elligible	Malay	currently married	Tertiary and above	≥USD1195	no	25-29 yo	1
471	2019-2021	35-45 yo	Not elligible	Malay	currently married	Secondary	<USD1195	no	25-29 yo	1
472	2019-2021	35-45 yo	Not elligible	Malay	currently married	Tertiary and above	≥USD1195	no	19-24 yo	1
473	2019-2021	35-45 yo	Not elligible	Malay	single	Secondary	<USD1195	no	25-29 yo	1
474	2019-2021	35-45 yo	Not elligible	Malay	separated/divorced/w	Secondary	<USD1195	no	19-24 yo	1
475	2019-2021	35-45 yo	Not elligible	Malay	currently married	Tertiary and above	<USD1195	no	19-24 yo	1
476	2019-2021	35-45 yo	Not elligible	Malay	currently married	Secondary	≥USD1195	no	25-29 yo	1
477	2019-2021	35-45 yo	Not elligible	Malay	currently married	Tertiary and above	≥USD1195	no	19-24 yo	2
478	2019-2021	35-45 yo	Not elligible	Malay	currently married	Tertiary and above	≥USD1195	no	25-29 yo	1
479	2019-2021	35-45 yo	Not elligible	Malay	currently married	Secondary	≥USD1195	no	19-24 yo	1
481	2019-2021	35-45 yo	Not elligible	Chinese	currently married	Tertiary and above	≥USD1195	yes	25-29 yo	1
482	2019-2021	35-45 yo	Not elligible	Malay	currently married	Tertiary and above	≥USD1195	no	25-29 yo	1
483	2019-2021	35-45 yo	Not elligible	Malay	currently married	Secondary	≥USD1195	no	19-24 yo	1
484	2019-2021	35-45 yo	Not elligible	Malay	currently married	Secondary	≥USD1195	no	25-29 yo	1
485	2019-2021	35-45 yo	Not elligible	Malay	currently married	Secondary	<USD1195	no	19-24 yo	1
486	2019-2021	35-45 yo	Not elligible	Chinese	currently married	Tertiary and above	≥USD1195	yes	25-29 yo	1
487	2019-2021	35-45 yo	Not elligible	Malay	currently married	Tertiary and above	≥USD1195	no	25-29 yo	1

488	2019-2021	35-45 yo	Not elligible	Malay	currently married	Tertiary and above	≥USD1195	no	19-24 yo	1
489	2019-2021	35-45 yo	Not elligible	Malay	currently married	Tertiary and above	≥USD1195	no	25-29 yo	1
490	2019-2021	35-45 yo	Not elligible	Malay	currently married	Tertiary and above	≥USD1195	no	25-29 yo	1
491	2019-2021	35-45 yo	Not elligible	Chinese	currently married	Tertiary and above	≥USD1195	yes	25-29 yo	1
492	2019-2021	35-45 yo	Not elligible	Chinese	separated/divorced/w	Tertiary and above	≥USD1195	yes	19-24 yo	1
493	2019-2021	35-45 yo	Not elligible	Indian	currently married	Tertiary and above	≥USD1195	yes	25-29 yo	1
494	2019-2021	35-45 yo	Not elligible	Chinese	currently married	Tertiary and above	≥USD1195	no	19-24 yo	1
495	2019-2021	35-45 yo	Not elligible	Chinese	currently married	Tertiary and above	≥USD1195	no	19-24 yo	2
496	2019-2021	35-45 yo	Not elligible	Chinese	currently married	Tertiary and above	≥USD1195	yes	19-24 yo	1
497	2019-2021	35-45 yo	Not elligible	Indian	currently married	Tertiary and above	≥USD1195	no	18 yo and below	3 and above
498	2019-2021	35-45 yo	Not elligible	Chinese	currently married	Tertiary and above	≥USD1195	no	19-24 yo	2
499	2019-2021	35-45 yo	Not elligible	Chinese	currently married	Tertiary and above	≥USD1195	no	19-24 yo	1
500	2019-2021	35-45 yo	Not elligible	Malay	currently married	Tertiary and above	≥USD1195	no	19-24 yo	1
501	2019-2021	35-45 yo	Not elligible	Chinese	currently married	Tertiary and above	≥USD1195	no	19-24 yo	3 and above
502	2019-2021	35-45 yo	Not elligible	Chinese	currently married	Tertiary and above	≥USD1195	no	19-24 yo	1
503	2019-2021	35-45 yo	Not elligible	Chinese	currently married	Tertiary and above	≥USD1195	no	30 and above	1
504	2019-2021	35-45 yo	Not elligible	Chinese	currently married	Tertiary and above	≥USD1195	yes	30 and above	1
505	2019-2021	35-45 yo	Not elligible	Chinese	currently married	Tertiary and above	≥USD1195	yes	18 yo and below	
506	2019-2021	35-45 yo	Not elligible	Chinese	currently married	Tertiary and above	≥USD1195	yes	25-29 yo	1
507	2019-2021	35-45 yo	Not elligible	Malay	currently married	Tertiary and above	≥USD1195	no	25-29 yo	1
508	2019-2021	35-45 yo	Not elligible	Malay	currently married	Tertiary and above	≥USD1195	no	19-24 yo	1
509	2019-2021	18-24 yo	school-based	Malay	currently married	Secondary	<USD1195	yes	18 yo and below	1
510	2019-2021	18-24 yo	school-based	Malay	currently married	Secondary	<USD1195	yes	18 yo and below	1
511	2019-2021	35-45 yo	Not elligible	Indian	currently married	Tertiary and above	≥USD1195	yes	30 and above	1
512	2019-2021	35-45 yo	Not elligible	Malay	currently married	Tertiary and above	≥USD1195	no	25-29 yo	1
513	2019-2021	35-45 yo	Not elligible	Chinese	currently married	Tertiary and above	≥USD1195	yes	30 and above	1
514	2019-2021	35-45 yo	Not elligible	Malay	currently married	Secondary	<USD1195	no	25-29 yo	2
515	2019-2021	35-45 yo	Not elligible	Chinese	currently married	Tertiary and above	≥USD1195	yes	25-29 yo	1
516	2019-2021	35-45 yo	Not elligible	Malay	currently married	Tertiary and above	≥USD1195	no	19-24 yo	1
517	2019-2021	35-45 yo	Not elligible	Malay	currently married	Tertiary and above	<USD1195	no	19-24 yo	1
518	2019-2021	18-24 yo	catchup	Other	currently married	Tertiary and above	≥USD1195	yes	19-24 yo	1
519	2019-2021	35-45 yo	Not elligible	Malay	currently married	Tertiary and above	≥USD1195	no	25-29 yo	1
520	2019-2021	35-45 yo	Not elligible	Indian	currently married	Secondary	<USD1195	no	19-24 yo	1
521	2019-2021	35-45 yo	Not elligible	Malay	separated/divorced/w	Secondary	<USD1195	no	18 yo and below	1
522	2019-2021	18-24 yo	school-based	Other	currently married	Secondary	<USD1195	yes	18 yo and below	1
523	2019-2021	35-45 yo	Not elligible	Indian	separated/divorced/w	Secondary	<USD1195	no	18 yo and below	2
524	2019-2021	35-45 yo	Not elligible	Malay	currently married	Secondary	<USD1195	no	25-29 yo	1
525	2019-2021	35-45 yo	Not elligible	Chinese	currently married	Secondary	≥USD1195	no	19-24 yo	1
526	2019-2021	35-45 yo	Not elligible	Malay	currently married	Secondary	<USD1195	no	19-24 yo	1
527	2019-2021	35-45 yo	Not elligible	Chinese	currently married	Tertiary and above	≥USD1195	no	25-29 yo	1

528	2019-2021	35-45 yo	Not elligible	Chinese	currently married	Tertiary and above	≥USD1195	no	19-24 yo	2
529	2019-2021	35-45 yo	Not elligible	Malay	currently married	Tertiary and above	≥USD1195	no	25-29 yo	1
530	2019-2021	35-45 yo	Not elligible	Chinese	currently married	Tertiary and above	≥USD1195	yes	25-29 yo	1
531	2019-2021	35-45 yo	Not elligible	Chinese	currently married	Tertiary and above	≥USD1195	yes	25-29 yo	1
532	2019-2021	35-45 yo	Not elligible	Malay	currently married	Tertiary and above	≥USD1195	no	25-29 yo	1
533	2019-2021	35-45 yo	Not elligible	Chinese	currently married	Tertiary and above	≥USD1195	no	25-29 yo	1
534	2019-2021	35-45 yo	Not elligible	Indian	currently married	Tertiary and above	<USD1195	no	25-29 yo	1
536	2019-2021	35-45 yo	Not elligible	Malay	currently married	Secondary	<USD1195	no	19-24 yo	1
537	2019-2021	35-45 yo	Not elligible	Malay	currently married	Secondary	<USD1195	no	18 yo and below	1
538	2019-2021	35-45 yo	Not elligible	Malay	currently married	Tertiary and above	≥USD1195	no	25-29 yo	1
539	2019-2021	35-45 yo	Not elligible	Malay	currently married	None-primary	<USD1195	no	19-24 yo	1
540	2019-2021	35-45 yo	Not elligible	Indian	separated/divorced/w	Tertiary and above	<USD1195	no	19-24 yo	2
541	2019-2021	35-45 yo	Not elligible	Indian	currently married	Tertiary and above	<USD1195	no	25-29 yo	1
542	2019-2021	35-45 yo	Not elligible	Malay	currently married	None-primary	<USD1195	no	19-24 yo	1
543	2019-2021	35-45 yo	Not elligible	Indian	currently married	Secondary	<USD1195	no	19-24 yo	1
544	2019-2021	18-24 yo	school-based	Malay	currently married	Secondary	<USD1195	yes	19-24 yo	1
545	2019-2021	35-45 yo	Not elligible	Malay	currently married	Secondary	<USD1195	no	19-24 yo	2
546	2019-2021	35-45 yo	Not elligible	Malay	separated/divorced/w	Tertiary and above	<USD1195	no	25-29 yo	1
547	2019-2021	35-45 yo	Not elligible	Malay	currently married	Tertiary and above	≥USD1195	yes	30 and above	1
548	2019-2021	35-45 yo	Not elligible	Malay	currently married	Tertiary and above	≥USD1195	no	25-29 yo	1
549	2019-2021	35-45 yo	Not elligible	Chinese	currently married	Tertiary and above	≥USD1195	no	30 and above	1
550	2019-2021	35-45 yo	Not elligible	Indian	currently married	Tertiary and above	≥USD1195	no	25-29 yo	1
551	2019-2021	35-45 yo	Not elligible	Malay	currently married	Secondary	<USD1195	no	25-29 yo	1
552	2019-2021	35-45 yo	Not elligible	Indian	currently married	Tertiary and above	≥USD1195	no	25-29 yo	1
553	2019-2021	35-45 yo	Not elligible	Indian	currently married	Tertiary and above	≥USD1195	no	30 and above	1
554	2019-2021	35-45 yo	Not elligible	Malay	currently married	Secondary	≥USD1195	no	19-24 yo	1
555	2019-2021	35-45 yo	Not elligible	Malay	currently married	Tertiary and above	≥USD1195	no	19-24 yo	1
556	2019-2021	35-45 yo	Not elligible	Malay	currently married	Tertiary and above	≥USD1195	no	19-24 yo	1
557	2019-2021	35-45 yo	Not elligible	Malay	currently married	Tertiary and above	≥USD1195	no	25-29 yo	1
558	2019-2021	35-45 yo	Not elligible	Malay	currently married	Tertiary and above	≥USD1195	no	25-29 yo	1
559	2019-2021	35-45 yo	Not elligible	Malay	currently married	Tertiary and above	≥USD1195	no	25-29 yo	2
560	2019-2021	35-45 yo	Not elligible	Malay	currently married	Tertiary and above	≥USD1195	yes	25-29 yo	1
561	2019-2021	35-45 yo	Not elligible	Malay	currently married	Tertiary and above	≥USD1195	no	25-29 yo	1
562	2019-2021	35-45 yo	Not elligible	Malay	currently married	Secondary	<USD1195	no	19-24 yo	1
563	2019-2021	35-45 yo	Not elligible	Malay	currently married	Secondary	<USD1195	no	25-29 yo	1
564	2019-2021	35-45 yo	Not elligible	Malay	currently married	Tertiary and above	≥USD1195	no	25-29 yo	1
565	2019-2021	35-45 yo	Not elligible	Malay	currently married	Tertiary and above	<USD1195	no	19-24 yo	1
566	2019-2021	35-45 yo	Not elligible	Malay	currently married	Tertiary and above	≥USD1195	no	19-24 yo	1
567	2019-2021	35-45 yo	Not elligible	Chinese	currently married	Tertiary and above	≥USD1195	no	19-24 yo	1
568	2019-2021	35-45 yo	Not elligible	Malay	separated/divorced/w	Tertiary and above	<USD1195	no	25-29 yo	1

569	2019-2021	35-45 yo	Not elligible	Malay	currently married	Secondary	<USD1195	no	25-29 yo	1
570	2019-2021	35-45 yo	Not elligible	Malay	currently married	Tertiary and above	≥USD1195	no	25-29 yo	1
571	2019-2021	35-45 yo	Not elligible	Malay	currently married	Tertiary and above	≥USD1195	yes	25-29 yo	1
572	2019-2021	35-45 yo	Not elligible	Chinese	currently married	Tertiary and above	≥USD1195	no	25-29 yo	1
573	2019-2021	35-45 yo	Not elligible	Malay	separated/divorced/w	Secondary	<USD1195	no	25-29 yo	2
574	2019-2021	35-45 yo	Not elligible	Chinese	currently married	Tertiary and above	≥USD1195	yes	19-24 yo	1
575	2019-2021	35-45 yo	Not elligible	Chinese	currently married	Tertiary and above	≥USD1195	no	25-29 yo	1
576	2019-2021	35-45 yo	Not elligible	Malay	currently married	Tertiary and above	≥USD1195	no	19-24 yo	1
577	2019-2021	35-45 yo	Not elligible	Malay	currently married	Tertiary and above	≥USD1195	no	19-24 yo	1
578	2019-2021	35-45 yo	Not elligible	Malay	currently married	Tertiary and above	≥USD1195	no	25-29 yo	1
579	2019-2021	35-45 yo	Not elligible	Malay	currently married	Tertiary and above	≥USD1195	no	25-29 yo	1
580	2019-2021	35-45 yo	Not elligible	Malay	currently married	Tertiary and above	≥USD1195	no	25-29 yo	1
581	2019-2021	35-45 yo	Not elligible	Malay	currently married	Tertiary and above	≥USD1195	yes	19-24 yo	1
582	2019-2021	35-45 yo	Not elligible	Malay	currently married	Tertiary and above	≥USD1195	no	25-29 yo	1
583	2019-2021	35-45 yo	Not elligible	Malay	currently married	Tertiary and above	≥USD1195	yes	25-29 yo	1
584	2019-2021	35-45 yo	Not elligible	Malay	currently married	Tertiary and above	≥USD1195	no	19-24 yo	1
585	2019-2021	35-45 yo	Not elligible	Malay	currently married	Tertiary and above	≥USD1195	no	25-29 yo	1
586	2019-2021	35-45 yo	Not elligible	Chinese	currently married	Tertiary and above	≥USD1195	yes	25-29 yo	
587	2019-2021	35-45 yo	Not elligible	Other	currently married	Tertiary and above	≥USD1195	yes	25-29 yo	1
588	2019-2021	35-45 yo	Not elligible	Chinese	currently married	Tertiary and above	≥USD1195	no	19-24 yo	3 and above
589	2019-2021	35-45 yo	Not elligible	Malay	currently married	Tertiary and above	≥USD1195	no	25-29 yo	1
590	2019-2021	35-45 yo	Not elligible	Chinese	currently married	Tertiary and above	≥USD1195	no	19-24 yo	3 and above
591	2019-2021	35-45 yo	Not elligible	Chinese	currently married	Tertiary and above	≥USD1195	no	19-24 yo	1
592	2019-2021	35-45 yo	Not elligible	Chinese	currently married	Tertiary and above	≥USD1195	no	19-24 yo	2
593	2019-2021	35-45 yo	Not elligible	Chinese	currently married	Tertiary and above	≥USD1195	yes	25-29 yo	2
594	2019-2021	35-45 yo	Not elligible	Indian	currently married	Secondary	<USD1195	no	19-24 yo	1
595	2019-2021	35-45 yo	Not elligible	Indian	separated/divorced/w	Secondary	<USD1195	no	18 yo and below	1
596	2019-2021	35-45 yo	Not elligible	Malay	currently married	Tertiary and above	≥USD1195	no	19-24 yo	1
597	2019-2021	35-45 yo	Not elligible	Malay	currently married	Tertiary and above	<USD1195	no	25-29 yo	1
598	2019-2021	35-45 yo	Not elligible	Malay	currently married	Tertiary and above	<USD1195	no	25-29 yo	1
599	2019-2021	35-45 yo	Not elligible	Malay	currently married	Secondary	<USD1195	no	18 yo and below	1
600	2019-2021	35-45 yo	Not elligible	Malay	currently married	Tertiary and above	≥USD1195	no	30 and above	1
601	2019-2021	35-45 yo	Not elligible	Malay	currently married	Tertiary and above	<USD1195	no	19-24 yo	1
602	2019-2021	35-45 yo	Not elligible	Malay	currently married	Tertiary and above	<USD1195	no	19-24 yo	1
603	2019-2021	35-45 yo	Not elligible	Chinese	currently married	Tertiary and above	<USD1195	no	19-24 yo	3 and above
604	2019-2021	35-45 yo	Not elligible	Chinese	currently married	Secondary	<USD1195	no	18 yo and below	1
605	2019-2021	18-24 yo	catchup	Indian	currently married	Secondary	<USD1195	yes	19-24 yo	1
606	2019-2021	35-45 yo	Not elligible	Indian	currently married	Tertiary and above	<USD1195	no	25-29 yo	1
607	2019-2021	35-45 yo	Not elligible	Malay	currently married	Tertiary and above	≥USD1195	no	25-29 yo	1
608	2019-2021	35-45 yo	Not elligible	Malay	currently married	Secondary	≥USD1195	no	25-29 yo	1

609	2019-2021	35-45 yo	Not elligible	Malay	currently married	Tertiary and above	≥USD1195	no	19-24 yo	1
610	2019-2021	35-45 yo	Not elligible	Malay	currently married	Secondary	<USD1195	no	19-24 yo	1
611	2019-2021	35-45 yo	Not elligible	Chinese	currently married	Secondary	<USD1195	no	19-24 yo	2
612	2019-2021	35-45 yo	Not elligible	Indian	currently married	None-primary	<USD1195	no	19-24 yo	2
613	2019-2021	35-45 yo	Not elligible	Indian	currently married	Secondary	<USD1195	no	30 and above	1
614	2019-2021	35-45 yo	Not elligible	Indian	currently married	Secondary	<USD1195	no	25-29 yo	1
615	2019-2021	35-45 yo	Not elligible	Malay	currently married	Tertiary and above	≥USD1195	no	19-24 yo	1
616	2019-2021	35-45 yo	Not elligible	Malay	currently married	Tertiary and above	≥USD1195	no	25-29 yo	1
617	2019-2021	35-45 yo	Not elligible	Indian	currently married	Secondary	<USD1195	no	19-24 yo	1
618	2019-2021	35-45 yo	Not elligible	Malay	currently married	Secondary	<USD1195	no	25-29 yo	1
619	2019-2021	35-45 yo	Not elligible	Malay	currently married	Tertiary and above	<USD1195	no	25-29 yo	1
620	2019-2021	35-45 yo	Not elligible	Chinese	currently married	Tertiary and above	≥USD1195	no	19-24 yo	1
621	2019-2021	35-45 yo	Not elligible	Malay	currently married	Secondary	<USD1195	no	25-29 yo	1
622	2019-2021	35-45 yo	Not elligible	Malay	currently married	Secondary	≥USD1195	no	25-29 yo	1
623	2019-2021	35-45 yo	Not elligible	Malay	currently married	Secondary	<USD1195	no	19-24 yo	1
624	2019-2021	35-45 yo	Not elligible	Malay	currently married	Secondary	≥USD1195	no	19-24 yo	1
625	2019-2021	35-45 yo	Not elligible	Malay	currently married	Secondary	<USD1195	no	25-29 yo	1
626	2019-2021	35-45 yo	Not elligible	Malay	currently married	Secondary	≥USD1195	no	30 and above	1
627	2019-2021	35-45 yo	Not elligible	Malay	currently married	Secondary	≥USD1195	no	19-24 yo	1
628	2019-2021	35-45 yo	Not elligible	Malay	currently married	Tertiary and above	<USD1195	no	30 and above	1
629	2019-2021	35-45 yo	Not elligible	Malay	currently married	None-primary	<USD1195	no	25-29 yo	1
630	2019-2021	35-45 yo	Not elligible	Malay	currently married	Tertiary and above	≥USD1195	no	19-24 yo	1
631	2019-2021	35-45 yo	Not elligible	Malay	currently married	Tertiary and above	<USD1195	no	25-29 yo	1
632	2019-2021	35-45 yo	Not elligible	Malay	currently married	Tertiary and above	≥USD1195	no	19-24 yo	1
633	2019-2021	35-45 yo	Not elligible	Malay	currently married	Tertiary and above	<USD1195	no	25-29 yo	1
634	2019-2021	18-24 yo	school-based	Malay	currently married	Secondary	<USD1195	yes	18 yo and below	1
635	2019-2021	35-45 yo	Not elligible	Malay	currently married	Tertiary and above	≥USD1195	no	25-29 yo	1
636	2019-2021	35-45 yo	Not elligible	Malay	currently married	Tertiary and above	<USD1195	no	19-24 yo	1
637	2019-2021	18-24 yo	school-based	Indian	currently married	Secondary	<USD1195	yes	19-24 yo	1
638	2019-2021	35-45 yo	Not elligible	Malay	currently married	Secondary	<USD1195	no	25-29 yo	1
639	2019-2021	35-45 yo	Not elligible	Chinese	currently married	Tertiary and above	≥USD1195	no	25-29 yo	1
640	2019-2021	35-45 yo	Not elligible	Malay	currently married	Tertiary and above	≥USD1195	no	25-29 yo	1
641	2019-2021	35-45 yo	Not elligible	Chinese	currently married	Tertiary and above	≥USD1195	no	25-29 yo	1
642	2019-2021	18-24 yo	catchup	Indian	currently married	Secondary	<USD1195	yes	18 yo and below	3 and above
643	2019-2021	35-45 yo	Not elligible	Chinese	currently married	Tertiary and above	≥USD1195	no	19-24 yo	2
644	2019-2021	35-45 yo	Not elligible	Chinese	currently married	Tertiary and above	≥USD1195	no	30 and above	1
645	2019-2021	18-24 yo	catchup	Malay	currently married	None-primary	<USD1195	no	18 yo and below	1
646	2019-2021	35-45 yo	Not elligible	Malay	currently married	Secondary	<USD1195	no	19-24 yo	1
647	2019-2021	35-45 yo	Not elligible	Malay	currently married	Tertiary and above	≥USD1195	no	19-24 yo	1
648	2019-2021	35-45 yo	Not elligible	Indian	separated/divorced/w	Tertiary and above	≥USD1195	no	25-29 yo	3 and above

649	2019-2021	35-45 yo	Not elligible	Chinese	currently married	Tertiary and above	<USD1195	no	25-29 yo	1
650	2019-2021	35-45 yo	Not elligible	Chinese	currently married	Secondary	<USD1195	no	19-24 yo	2
651	2019-2021	35-45 yo	Not elligible	Malay	currently married	Secondary	<USD1195	no	25-29 yo	1
652	2019-2021	35-45 yo	Not elligible	Indian	currently married	Secondary	<USD1195	no	30 and above	1
653	2019-2021	35-45 yo	Not elligible	Malay	currently married	Secondary	<USD1195	no	19-24 yo	1
654	2019-2021	18-24 yo	catchup	Chinese	single	Tertiary and above	<USD1195	yes	19-24 yo	1
655	2019-2021	35-45 yo	Not elligible	Malay	currently married	Tertiary and above	≥USD1195	no	25-29 yo	1
656	2019-2021	35-45 yo	Not elligible	Malay	currently married	Tertiary and above	≥USD1195	no	25-29 yo	2
657	2019-2021	35-45 yo	Not elligible	Malay	currently married	Secondary	<USD1195	no	30 and above	1
658	2019-2021	35-45 yo	Not elligible	Indian	currently married	Tertiary and above	≥USD1195	no	30 and above	1
659	2019-2021	35-45 yo	Not elligible	Malay	currently married	Secondary	≥USD1195	no	25-29 yo	1
661	2019-2021	35-45 yo	Not elligible	Indian	currently married	Tertiary and above	≥USD1195	no	30 and above	1
662	2019-2021	35-45 yo	Not elligible	Malay	currently married	Tertiary and above	≥USD1195	no	19-24 yo	1
663	2019-2021	35-45 yo	Not elligible	Malay	currently married	Secondary	<USD1195	no	19-24 yo	2
664	2019-2021	35-45 yo	Not elligible	Malay	currently married	Secondary	<USD1195	no	25-29 yo	1
665	2019-2021	35-45 yo	Not elligible	Malay	currently married	Secondary	<USD1195	no	25-29 yo	1
666	2019-2021	35-45 yo	Not elligible	Indian	separated/divorced/w	Tertiary and above	≥USD1195	no	19-24 yo	3 and above
667	2019-2021	35-45 yo	Not elligible	Chinese	currently married	Secondary	≥USD1195	no	19-24 yo	1
668	2019-2021	35-45 yo	Not elligible	Malay	currently married	Tertiary and above	≥USD1195	no	19-24 yo	3 and above
669	2019-2021	35-45 yo	Not elligible	Malay	currently married	Secondary	<USD1195	no	25-29 yo	1
670	2019-2021	35-45 yo	Not elligible	Indian	currently married	Secondary	<USD1195	no	25-29 yo	1
671	2019-2021	35-45 yo	Not elligible	Malay	currently married	Secondary	<USD1195	no	19-24 yo	1
672	2019-2021	35-45 yo	Not elligible	Chinese	separated/divorced/w	None-primary	≥USD1195	no	18 yo and below	2
673	2019-2021	35-45 yo	Not elligible	Indian	currently married	Tertiary and above	<USD1195	no	30 and above	1
674	2019-2021	35-45 yo	Not elligible	Malay	currently married	Secondary	<USD1195	no	19-24 yo	1
675	2019-2021	35-45 yo	Not elligible	Chinese	currently married	Tertiary and above	≥USD1195	no	19-24 yo	2
676	2019-2021	35-45 yo	Not elligible	Malay	currently married	Secondary	<USD1195	no	19-24 yo	1
677	2019-2021	35-45 yo	Not elligible	Malay	currently married	Secondary	<USD1195	no	19-24 yo	1
678	2019-2021	35-45 yo	Not elligible	Indian	currently married	Tertiary and above	≥USD1195	no	19-24 yo	1
679	2019-2021	35-45 yo	Not elligible	Chinese	currently married	Secondary	<USD1195	no	19-24 yo	1
680	2019-2021	35-45 yo	Not elligible	Malay	currently married	Secondary	<USD1195	no	19-24 yo	1
681	2019-2021	35-45 yo	Not elligible	Malay	currently married	Tertiary and above	≥USD1195	yes	19-24 yo	1
682	2019-2021	35-45 yo	Not elligible	Chinese	currently married	Secondary	<USD1195	no	18 yo and below	2
683	2019-2021	35-45 yo	Not elligible	Malay	currently married	Tertiary and above	≥USD1195	no	25-29 yo	1
684	2019-2021	18-24 yo	catchup	Malay	currently married	Tertiary and above	<USD1195	yes	19-24 yo	1
685	2019-2021	35-45 yo	Not elligible	Chinese	currently married	Tertiary and above	≥USD1195	no	19-24 yo	1
686	2019-2021	35-45 yo	Not elligible	Other	currently married	Tertiary and above	<USD1195	no	19-24 yo	1
687	2019-2021	35-45 yo	Not elligible	Malay	currently married	Tertiary and above	<USD1195	no	25-29 yo	1
688	2019-2021	35-45 yo	Not elligible	Malay	currently married	Tertiary and above	<USD1195	yes	25-29 yo	1
689	2019-2021	18-24 yo	catchup	Indian	single	Tertiary and above	<USD1195	no	19-24 yo	1

690	2019-2021	18-24 yo	catchup	Indian	single	Tertiary and above	≥USD1195	no	19-24 yo	1
691	2019-2021	35-45 yo	Not elligible	Malay	currently married	Secondary	<USD1195	no	18 yo and below	1
692	2019-2021	18-24 yo	catchup	Chinese	currently married	Secondary	<USD1195	yes	18 yo and below	3 and above
693	2019-2021	35-45 yo	Not elligible	Chinese	currently married	Secondary	≥USD1195	no	25-29 yo	1
694	2019-2021	35-45 yo	Not elligible	Malay	currently married	Tertiary and above	≥USD1195	no	25-29 yo	1
695	2019-2021	35-45 yo	Not elligible	Malay	currently married	Tertiary and above	≥USD1195	no	30 and above	1
696	2019-2021	35-45 yo	Not elligible	Malay	currently married	Tertiary and above	<USD1195	no	19-24 yo	1
697	2019-2021	35-45 yo	Not elligible	Malay	currently married	Secondary	<USD1195	no	25-29 yo	1
698	2019-2021	35-45 yo	Not elligible	Indian	currently married	Secondary	<USD1195	no	19-24 yo	1
699	2019-2021	35-45 yo	Not elligible	Indian	currently married	Secondary	<USD1195	no	19-24 yo	1
701	2019-2021	35-45 yo	Not elligible	Chinese	currently married	Tertiary and above	<USD1195	no	19-24 yo	1
702	2019-2021	35-45 yo	Not elligible	Malay	currently married	Tertiary and above	≥USD1195	no	25-29 yo	2
703	2019-2021	35-45 yo	Not elligible	Malay	currently married	Tertiary and above	≥USD1195	no	25-29 yo	1
704	2019-2021	35-45 yo	Not elligible	Chinese	currently married	Secondary	≥USD1195	no	19-24 yo	2
705	2019-2021	35-45 yo	Not elligible	Chinese	currently married	Tertiary and above	≥USD1195	no	25-29 yo	1
706	2019-2021	35-45 yo	Not elligible	Chinese	currently married	Secondary	≥USD1195	no	18 yo and below	3 and above
707	2019-2021	35-45 yo	Not elligible	Chinese	currently married	Tertiary and above	<USD1195	no	25-29 yo	1
708	2019-2021	35-45 yo	Not elligible	Chinese	currently married	Secondary	<USD1195	no	18 yo and below	3 and above
709	2019-2021	35-45 yo	Not elligible	Chinese	currently married	Tertiary and above	≥USD1195	no	19-24 yo	1
710	2019-2021	35-45 yo	Not elligible	Chinese	currently married	Tertiary and above	≥USD1195	no	25-29 yo	1
711	2019-2021	35-45 yo	Not elligible	Chinese	currently married	Secondary	≥USD1195	no	18 yo and below	1
712	2019-2021	35-45 yo	Not elligible	Indian	currently married	Secondary	<USD1195	no	19-24 yo	1
713	2019-2021	18-24 yo	school-based	Indian	currently married	Tertiary and above	<USD1195	yes	18 yo and below	1
714	2019-2021	35-45 yo	Not elligible	Chinese	currently married	Secondary	≥USD1195	no	19-24 yo	3 and above
715	2019-2021	35-45 yo	Not elligible	Indian	currently married	Secondary	<USD1195	no	25-29 yo	1
716	2019-2021	35-45 yo	Not elligible	Chinese	currently married	Tertiary and above	≥USD1195	no	19-24 yo	1
717	2019-2021	35-45 yo	Not elligible	Malay	currently married	Secondary	<USD1195	no	25-29 yo	1
718	2019-2021	35-45 yo	Not elligible	Indian	separated/divorced/w	Tertiary and above	≥USD1195	no	25-29 yo	2
719	2019-2021	35-45 yo	Not elligible	Malay	currently married	Tertiary and above	≥USD1195	no	18 yo and below	3 and above
720	2019-2021	35-45 yo	Not elligible	Malay	currently married	Secondary	≥USD1195	no	19-24 yo	1
721	2019-2021	35-45 yo	Not elligible	Chinese	currently married	None-primary	<USD1195	no	25-29 yo	1
722	2019-2021	35-45 yo	Not elligible	Malay	currently married	Tertiary and above	≥USD1195	no	30 and above	2
723	2019-2021	35-45 yo	Not elligible	Malay	currently married	Tertiary and above	<USD1195	no	25-29 yo	1
724	2019-2021	35-45 yo	Not elligible	Indian	currently married	Secondary	<USD1195	no	19-24 yo	1
725	2019-2021	35-45 yo	Not elligible	Indian	single	Tertiary and above	<USD1195	no	19-24 yo	1
726	2019-2021	35-45 yo	Not elligible	Malay	currently married	Secondary	<USD1195	no	25-29 yo	1
728	2019-2021	35-45 yo	Not elligible	Chinese	currently married	Tertiary and above	≥USD1195	no	19-24 yo	3 and above
729	2019-2021	35-45 yo	Not elligible	Chinese	currently married	Tertiary and above	≥USD1195	no	25-29 yo	2
730	2019-2021	35-45 yo	Not elligible	Malay	currently married	Tertiary and above	≥USD1195	no	19-24 yo	1
731	2019-2021	35-45 yo	Not elligible	Indian	currently married	Secondary	<USD1195	no	19-24 yo	1

732	2019-2021	35-45 yo	Not elligible	Malay	currently married	Tertiary and above	≥USD1195	no	19-24 yo	1
733	2019-2021	35-45 yo	Not elligible	Chinese	currently married	Secondary	≥USD1195	no	25-29 yo	2
734	2019-2021	35-45 yo	Not elligible	Indian	currently married	Secondary	<USD1195	no	19-24 yo	1
735	2019-2021	35-45 yo	Not elligible	Malay	currently married	Secondary	<USD1195	no	19-24 yo	1
736	2019-2021	35-45 yo	Not elligible	Other	currently married	Secondary	<USD1195	no	18 yo and below	3 and above
737	2019-2021	35-45 yo	Not elligible	Indian	currently married	Secondary	<USD1195	no	19-24 yo	1
738	2019-2021	35-45 yo	Not elligible	Chinese	currently married	Secondary	<USD1195	no	19-24 yo	1
739	2019-2021	35-45 yo	Not elligible	Indian	currently married	Tertiary and above	≥USD1195	no	25-29 yo	1
740	2019-2021	35-45 yo	Not elligible	Indian	currently married	Secondary	<USD1195	no	19-24 yo	2
741	2019-2021	35-45 yo	Not elligible	Chinese	currently married	Secondary	≥USD1195	no	18 yo and below	2
742	2019-2021	35-45 yo	Not elligible	Malay	currently married	Secondary	<USD1195	no	19-24 yo	1
743	2019-2021	35-45 yo	Not elligible	Chinese	currently married	Secondary	≥USD1195	no	19-24 yo	3 and above
744	2019-2021	35-45 yo	Not elligible	Malay	currently married	Secondary	<USD1195	no	30 and above	1
745	2019-2021	18-24 yo	catchup	Other	separated/divorced/w	None-primary	<USD1195	no	18 yo and below	2
746	2019-2021	35-45 yo	Not elligible	Chinese	currently married	Secondary	≥USD1195	no	18 yo and below	1
747	2019-2021	35-45 yo	Not elligible	Indian	currently married	Secondary	≥USD1195	no	19-24 yo	1
748	2019-2021	35-45 yo	Not elligible	Chinese	currently married	Secondary	≥USD1195	no	19-24 yo	2
749	2019-2021	35-45 yo	Not elligible	Chinese	single	Tertiary and above	≥USD1195	no	18 yo and below	3 and above
750	2019-2021	35-45 yo	Not elligible	Malay	separated/divorced/w	Secondary	<USD1195	no	18 yo and below	2
751	2019-2021	35-45 yo	Not elligible	Malay	currently married	Tertiary and above	≥USD1195	no	25-29 yo	2
752	2019-2021	35-45 yo	Not elligible	Chinese	currently married	Tertiary and above	≥USD1195	no	30 and above	1
753	2019-2021	35-45 yo	Not elligible	Chinese	separated/divorced/w	Secondary	<USD1195	no	18 yo and below	3 and above
754	2019-2021	35-45 yo	Not elligible	Chinese	currently married	Secondary	≥USD1195	no	18 yo and below	1
755	2019-2021	35-45 yo	Not elligible	Chinese	currently married	Secondary	≥USD1195	no	19-24 yo	1
756	2019-2021	35-45 yo	Not elligible	Malay	currently married	Tertiary and above	≥USD1195	no	19-24 yo	1
757	2019-2021	35-45 yo	Not elligible	Malay	currently married	Secondary	<USD1195	no	19-24 yo	1
758	2019-2021	35-45 yo	Not elligible	Malay	currently married	Secondary	<USD1195	no	18 yo and below	3 and above
759	2019-2021	18-24 yo	school-based	Malay	single	Secondary	<USD1195	yes	18 yo and below	3 and above
760	2019-2021	35-45 yo	Not elligible	Malay	currently married	Secondary	<USD1195	no	25-29 yo	1
761	2019-2021	35-45 yo	Not elligible	Indian	currently married	Secondary	<USD1195	no	30 and above	1
762	2019-2021	35-45 yo	Not elligible	Indian	currently married	Tertiary and above	<USD1195	yes	25-29 yo	1
763	2019-2021	35-45 yo	Not elligible	Chinese	currently married	Tertiary and above	≥USD1195	yes	19-24 yo	1
764	2019-2021	35-45 yo	Not elligible	Malay	currently married	Secondary	<USD1195	no	19-24 yo	1
765	2019-2021	35-45 yo	Not elligible	Indian	currently married	Secondary	≥USD1195	no	19-24 yo	1
766	2019-2021	35-45 yo	Not elligible	Chinese	currently married	Secondary	≥USD1195	no	18 yo and below	1
767	2019-2021	35-45 yo	Not elligible	Chinese	currently married	Tertiary and above	≥USD1195	no	30 and above	1
768	2019-2021	35-45 yo	Not elligible	Chinese	currently married	Secondary	<USD1195	no	19-24 yo	3 and above
769	2019-2021	35-45 yo	Not elligible	Indian	currently married	Secondary	<USD1195	no	19-24 yo	1
770	2019-2021	35-45 yo	Not elligible	Indian	currently married	Tertiary and above	<USD1195	no	30 and above	1
771	2019-2021	35-45 yo	Not elligible	Malay	currently married	Tertiary and above	≥USD1195	no	19-24 yo	1

772	2019-2021	35-45 yo	Not elligible	Indian	currently married	Tertiary and above	<USD1195	no	30 and above	1
773	2019-2021	35-45 yo	Not elligible	Malay	currently married	Tertiary and above	≥USD1195	no	19-24 yo	1
774	2019-2021	35-45 yo	Not elligible	Malay	currently married	None-primary	<USD1195	no	25-29 yo	1
775	2019-2021	35-45 yo	Not elligible	Indian	currently married	Tertiary and above	≥USD1195	no	25-29 yo	2
776	2019-2021	35-45 yo	Not elligible	Malay	separated/divorced/w	Tertiary and above	<USD1195	no	25-29 yo	1
777	2019-2021	35-45 yo	Not elligible	Chinese	currently married	Tertiary and above	≥USD1195	no	18 yo and below	2
778	2019-2021	35-45 yo	Not elligible	Malay	currently married	Secondary	<USD1195	no	30 and above	1
779	2019-2021	35-45 yo	Not elligible	Malay	currently married	Secondary	<USD1195	no	19-24 yo	1
780	2019-2021	35-45 yo	Not elligible	Malay	separated/divorced/w	Secondary	<USD1195	no	19-24 yo	1
781	2019-2021	35-45 yo	Not elligible	Chinese	currently married	Tertiary and above	≥USD1195	yes	19-24 yo	2
782	2019-2021	35-45 yo	Not elligible	Chinese	currently married	Secondary	≥USD1195	no	19-24 yo	1
783	2019-2021	35-45 yo	Not elligible	Malay	currently married	Secondary	<USD1195	no	18 yo and below	1
784	2019-2021	35-45 yo	Not elligible	Chinese	currently married	Tertiary and above	≥USD1195	no	25-29 yo	1
785	2019-2021	18-24 yo	catchup	Malay	currently married	Tertiary and above	<USD1195	no	19-24 yo	1
786	2019-2021	35-45 yo	Not elligible	Malay	currently married	Tertiary and above	≥USD1195	no	30 and above	1
787	2019-2021	35-45 yo	Not elligible	Malay	currently married	Tertiary and above	≥USD1195	no	19-24 yo	1
788	2019-2021	35-45 yo	Not elligible	Malay	currently married	Tertiary and above	≥USD1195	no	25-29 yo	1
789	2019-2021	35-45 yo	Not elligible	Indian	currently married	Tertiary and above	≥USD1195	no	19-24 yo	2
790	2019-2021	35-45 yo	Not elligible	Malay	currently married	Tertiary and above	≥USD1195	no	19-24 yo	1
791	2019-2021	35-45 yo	Not elligible	Indian	currently married	Tertiary and above	≥USD1195	no	25-29 yo	1
792	2019-2021	35-45 yo	Not elligible	Indian	currently married	Secondary	<USD1195	no	19-24 yo	1
793	2019-2021	35-45 yo	Not elligible	Indian	currently married	Tertiary and above	≥USD1195	no	30 and above	1
794	2019-2021	35-45 yo	Not elligible	Malay	currently married	Tertiary and above	≥USD1195	no	19-24 yo	1
795	2019-2021	35-45 yo	Not elligible	Malay	currently married	Tertiary and above	<USD1195	no	25-29 yo	1
796	2019-2021	18-24 yo	school-based	Chinese	single	Tertiary and above	<USD1195	yes	18 yo and below	3 and above
797	2019-2021	18-24 yo	catchup	Chinese	single	Tertiary and above	≥USD1195	yes	19-24 yo	3 and above
798	2019-2021	18-24 yo	school-based	Chinese	single	Tertiary and above	≥USD1195	no	18 yo and below	1
799	2019-2021	18-24 yo	school-based	Chinese	single	Tertiary and above	≥USD1195	yes	18 yo and below	2
800	2019-2021	18-24 yo	school-based	Chinese	single	Tertiary and above	≥USD1195	yes	19-24 yo	1
801	2019-2021	18-24 yo	school-based	Chinese	single	Tertiary and above	≥USD1195	yes	18 yo and below	2
802	2019-2021	18-24 yo	school-based	Chinese	single	Tertiary and above	≥USD1195	yes	19-24 yo	1
803	2019-2021	35-45 yo	Not elligible	Chinese	currently married	Tertiary and above	≥USD1195	no	25-29 yo	1
804	2019-2021	18-24 yo	school-based	Chinese	single	Tertiary and above	<USD1195	yes	18 yo and below	2
805	2019-2021	18-24 yo	school-based	Chinese	single	Tertiary and above	<USD1195	yes	19-24 yo	1
806	2019-2021	18-24 yo	school-based	Chinese	single	Tertiary and above	<USD1195	yes	18 yo and below	3 and above
807	2019-2021	18-24 yo	catchup	Chinese	single	Tertiary and above	<USD1195	yes	19-24 yo	1
808	2019-2021	18-24 yo	school-based	Chinese	single	Tertiary and above	≥USD1195	yes	18 yo and below	1
809	2019-2021	18-24 yo	school-based	Indian	single	Tertiary and above	<USD1195	yes	18 yo and below	3 and above
810	2019-2021	18-24 yo	school-based	Chinese	single	Tertiary and above	<USD1195	yes	19-24 yo	3 and above
811	2019-2021	18-24 yo	school-based	Indian	single	Tertiary and above	<USD1195	yes	18 yo and below	1

812	2019-2021	35-45 yo	Not elligible	Indian	currently married	None-primary	<USD1195	no	18 yo and below	3 and above
813	2019-2021	35-45 yo	Not elligible	Chinese	currently married	Tertiary and above	≥USD1195	no	19-24 yo	3 and above
814	2019-2021	35-45 yo	Not elligible	Chinese	currently married	Tertiary and above	≥USD1195	no	19-24 yo	3 and above
815	2019-2021	35-45 yo	Not elligible	Chinese	currently married	Tertiary and above	≥USD1195	no	30 and above	1
816	2019-2021	35-45 yo	Not elligible	Chinese	currently married	Tertiary and above	≥USD1195	no	18 yo and below	1
817	2019-2021	35-45 yo	Not elligible	Chinese	currently married	Tertiary and above	≥USD1195	no	25-29 yo	2
818	2019-2021	35-45 yo	Not elligible	Indian	currently married	Tertiary and above	≥USD1195	no	30 and above	1
819	2019-2021	35-45 yo	Not elligible	Indian	currently married	Tertiary and above	≥USD1195	no	25-29 yo	2
820	2019-2021	35-45 yo	Not elligible	Malay	currently married	Tertiary and above	≥USD1195	no	19-24 yo	1
821	2019-2021	18-24 yo	school-based	Chinese	single	Tertiary and above	<USD1195	yes	18 yo and below	3 and above
822	2019-2021	18-24 yo	school-based	Chinese	single	Tertiary and above	≥USD1195	no	18 yo and below	1
823	2019-2021	18-24 yo	school-based	Malay	single	Tertiary and above	≥USD1195	yes	19-24 yo	2
824	2019-2021	35-45 yo	Not elligible	Indian	separated/divorced/w	Tertiary and above	≥USD1195	no	25-29 yo	
825	2019-2021	35-45 yo	Not elligible	Malay	currently married	Tertiary and above	≥USD1195	no	25-29 yo	1
826	2019-2021	35-45 yo	Not elligible	Indian	single	Tertiary and above	≥USD1195	no		
827	2019-2021	18-24 yo	school-based	Malay	single	Tertiary and above	<USD1195	yes	19-24 yo	1
828	2019-2021	18-24 yo	school-based	Malay	single	Tertiary and above	≥USD1195	yes	18 yo and below	3 and above
829	2019-2021	18-24 yo	school-based	Chinese	single	Tertiary and above	<USD1195	yes	19-24 yo	1
830	2019-2021	35-45 yo	Not elligible	Chinese	currently married	Tertiary and above	≥USD1195	no	30 and above	1
831	2019-2021	18-24 yo	school-based	Malay	single	Tertiary and above	<USD1195	yes	19-24 yo	1
832	2019-2021	18-24 yo	school-based	Chinese	single	Tertiary and above	≥USD1195	yes	19-24 yo	1
833	2019-2021	18-24 yo	school-based	Chinese	single	Tertiary and above	≥USD1195	yes	19-24 yo	1
834	2019-2021	35-45 yo	Not elligible	Indian	currently married	Tertiary and above	≥USD1195	no	19-24 yo	1
835	2019-2021	35-45 yo	Not elligible	Malay	currently married	Tertiary and above	≥USD1195	no	25-29 yo	1
836	2019-2021	35-45 yo	Not elligible	Malay	currently married	Tertiary and above	≥USD1195	no	25-29 yo	1
837	2019-2021	35-45 yo	Not elligible	Malay	currently married	Tertiary and above	≥USD1195	no	25-29 yo	1
838	2019-2021	35-45 yo	Not elligible	Malay	currently married	Tertiary and above	≥USD1195	no	19-24 yo	1
839	2019-2021	35-45 yo	Not elligible	Malay	currently married	Tertiary and above	≥USD1195	no	25-29 yo	1
840	2019-2021	35-45 yo	Not elligible	Malay	currently married	Secondary	<USD1195	no	19-24 yo	1
841	2019-2021	35-45 yo	Not elligible	Indian	currently married	Tertiary and above	≥USD1195	no	25-29 yo	1
842	2019-2021	35-45 yo	Not elligible	Malay	currently married	Secondary	≥USD1195	no	19-24 yo	1
843	2019-2021	18-24 yo	catchup	Malay	single	Tertiary and above	<USD1195	no	18 yo and below	1
844	2019-2021	35-45 yo	Not elligible	Malay	currently married	Secondary	<USD1195	no	19-24 yo	1
845	2019-2021	35-45 yo	Not elligible	Indian	currently married	Secondary	<USD1195	no	25-29 yo	1
846	2019-2021	35-45 yo	Not elligible	Malay	currently married	Secondary	<USD1195	no	19-24 yo	1
847	2019-2021	35-45 yo	Not elligible	Malay	currently married	Tertiary and above	≥USD1195	no	19-24 yo	1
848	2019-2021	35-45 yo	Not elligible	Chinese	currently married	Tertiary and above	≥USD1195	no	25-29 yo	1
849	2019-2021	18-24 yo	catchup	Chinese	single	Tertiary and above	<USD1195	no	19-24 yo	1
850	2019-2021	35-45 yo	Not elligible	Malay	currently married	Tertiary and above	≥USD1195	no	25-29 yo	1
851	2019-2021	35-45 yo	Not elligible	Malay	currently married	Tertiary and above	<USD1195	no	25-29 yo	1

852	2019-2021	35-45 yo	Not elligible	Malay	currently married	Tertiary and above	≥USD1195	no	25-29 yo	1
853	2019-2021	35-45 yo	Not elligible	Malay	currently married	Tertiary and above	≥USD1195	no	25-29 yo	1
854	2019-2021	35-45 yo	Not elligible	Malay	currently married	Tertiary and above	≥USD1195	no	25-29 yo	1
855	2019-2021	35-45 yo	Not elligible	Malay	single	Tertiary and above	<USD1195	no	25-29 yo	1
856	2019-2021	35-45 yo	Not elligible	Malay	currently married	Tertiary and above	<USD1195	no	19-24 yo	1
857	2019-2021	35-45 yo	Not elligible	Chinese	currently married	Tertiary and above	≥USD1195	no	18 yo and below	3 and above
858	2019-2021	35-45 yo	Not elligible	Indian	separated/divorced/w	Tertiary and above	<USD1195	no	30 and above	2
859	2019-2021	35-45 yo	Not elligible	Malay	currently married	Tertiary and above	<USD1195	no	25-29 yo	1
860	2019-2021	35-45 yo	Not elligible	Malay	currently married	Tertiary and above	≥USD1195	no	19-24 yo	1
861	2019-2021	35-45 yo	Not elligible	Malay	currently married	Secondary	<USD1195	no	30 and above	1
862	2019-2021	35-45 yo	Not elligible	Malay	currently married	Secondary	<USD1195	no	19-24 yo	1
863	2019-2021	35-45 yo	Not elligible	Malay	currently married	Secondary	≥USD1195	no	25-29 yo	1
864	2019-2021	35-45 yo	Not elligible	Malay	currently married	Secondary	<USD1195	no	25-29 yo	1
865	2019-2021	35-45 yo	Not elligible	Malay	currently married	Secondary	<USD1195	no	19-24 yo	1
866	2019-2021	35-45 yo	Not elligible	Indian	currently married	Tertiary and above	≥USD1195	no	25-29 yo	1
867	2019-2021	35-45 yo	Not elligible	Indian	separated/divorced/w	Secondary	<USD1195	no	30 and above	1
868	2019-2021	35-45 yo	Not elligible	Malay	currently married	Tertiary and above	≥USD1195	no	25-29 yo	1
869	2019-2021	35-45 yo	Not elligible	Malay	currently married	Tertiary and above	≥USD1195	no	25-29 yo	1
870	2019-2021	18-24 yo	catchup	Indian	single	Tertiary and above	<USD1195	no	19-24 yo	2
871	2019-2021	35-45 yo	Not elligible	Malay	currently married	Tertiary and above	<USD1195	no	19-24 yo	1
872	2019-2021	35-45 yo	Not elligible	Malay	currently married	Tertiary and above	≥USD1195	no	25-29 yo	1
873	2019-2021	35-45 yo	Not elligible	Malay	currently married	Tertiary and above	≥USD1195	no	25-29 yo	1
874	2019-2021	35-45 yo	Not elligible	Malay	currently married	Tertiary and above	≥USD1195	no	19-24 yo	1
875	2019-2021	35-45 yo	Not elligible	Malay	currently married	Tertiary and above	≥USD1195	no	30 and above	1
876	2019-2021	35-45 yo	Not elligible	Malay	currently married	Tertiary and above	≥USD1195	no	19-24 yo	1
877	2019-2021	35-45 yo	Not elligible	Malay	separated/divorced/w	Tertiary and above	<USD1195	no	19-24 yo	1
878	2019-2021	35-45 yo	Not elligible	Malay	currently married	Tertiary and above	<USD1195	no	25-29 yo	1
880	2019-2021	35-45 yo	Not elligible	Malay	currently married	Tertiary and above	≥USD1195	no	19-24 yo	1
881	2019-2021	35-45 yo	Not elligible	Malay	currently married	Tertiary and above	≥USD1195	no	25-29 yo	1
882	2019-2021	35-45 yo	Not elligible	Chinese	currently married	Tertiary and above	≥USD1195	yes	19-24 yo	3 and above
883	2019-2021	35-45 yo	Not elligible	Malay	currently married	Secondary	<USD1195	no	19-24 yo	1
884	2019-2021	35-45 yo	Not elligible	Malay	currently married	Secondary	<USD1195	no	25-29 yo	1
885	2019-2021	35-45 yo	Not elligible	Malay	currently married	Tertiary and above	≥USD1195	no	19-24 yo	1
886	2019-2021	18-24 yo	school-based	Chinese	single	Tertiary and above	≥USD1195	yes	18 yo and below	3 and above
887	2019-2021	18-24 yo	school-based	Chinese	single	Tertiary and above	≥USD1195	yes	19-24 yo	1
888	2019-2021	35-45 yo	Not elligible	Chinese	currently married	Tertiary and above	≥USD1195	no	19-24 yo	3 and above
889	2019-2021	18-24 yo	catchup	Chinese	single	Tertiary and above	≥USD1195	yes	18 yo and below	1
890	2019-2021	35-45 yo	Not elligible	Indian	currently married	Tertiary and above	≥USD1195	no	25-29 yo	1
891	2019-2021	18-24 yo	catchup	Malay	currently married	Tertiary and above	<USD1195	no	19-24 yo	1
893	2019-2021	35-45 yo	Not elligible	Malay	currently married	Secondary	≥USD1195	no	19-24 yo	1

894	2019-2021	18-24 yo	school-based	Malay	currently married	Secondary	<USD1195	yes	18 yo and below	1
896	2019-2021	35-45 yo	Not elligible	Malay	currently married	Tertiary and above	<USD1195	no	30 and above	1
897	2019-2021	35-45 yo	Not elligible	Malay	currently married	Tertiary and above	≥USD1195	no	25-29 yo	1
898	2019-2021	35-45 yo	Not elligible	Malay	currently married	Tertiary and above	≥USD1195	no	19-24 yo	1
899	2019-2021	35-45 yo	Not elligible	Malay	currently married	Tertiary and above	≥USD1195	no	25-29 yo	1
900	2019-2021	35-45 yo	Not elligible	Indian	currently married	Tertiary and above	≥USD1195	no	25-29 yo	2
901	2019-2021	35-45 yo	Not elligible	Malay	currently married	Tertiary and above	≥USD1195	no	19-24 yo	3 and above
902	2019-2021	35-45 yo	Not elligible	Chinese	currently married	Tertiary and above	≥USD1195	no	18 yo and below	3 and above
903	2019-2021	18-24 yo	catchup	Chinese	single	Tertiary and above	≥USD1195	no	19-24 yo	2
904	2019-2021	35-45 yo	Not elligible	Indian	currently married	Tertiary and above	≥USD1195	yes	19-24 yo	3 and above
905	2019-2021	35-45 yo	Not elligible	Malay	currently married	Secondary	<USD1195	no	30 and above	2
906	2019-2021	35-45 yo	Not elligible	Malay	currently married	Secondary	<USD1195	no	19-24 yo	1
907	2019-2021	35-45 yo	Not elligible	Malay	currently married	Tertiary and above	≥USD1195	no	25-29 yo	1
908	2019-2021	35-45 yo	Not elligible	Malay	currently married	Secondary	≥USD1195	yes	19-24 yo	1
909	2019-2021	35-45 yo	Not elligible	Other	currently married	Tertiary and above	≥USD1195	no	30 and above	1
910	2019-2021	35-45 yo	Not elligible	Malay	currently married	Tertiary and above	≥USD1195	no	25-29 yo	1
911	2019-2021	35-45 yo	Not elligible	Malay	currently married	Tertiary and above	≥USD1195	no	19-24 yo	1
912	2019-2021	18-24 yo	catchup	Malay	currently married	Tertiary and above	≥USD1195	yes	19-24 yo	1
913	2019-2021	35-45 yo	Not elligible	Malay	currently married	Tertiary and above	≥USD1195	yes	19-24 yo	1
914	2019-2021	18-24 yo	school-based	Malay	currently married	Tertiary and above	<USD1195	yes	18 yo and below	1
915	2019-2021	35-45 yo	Not elligible	Chinese	single	Tertiary and above	≥USD1195	yes	30 and above	1
916	2019-2021	18-24 yo	catchup	Chinese	single	Tertiary and above	≥USD1195	yes	19-24 yo	1
917	2019-2021	35-45 yo	Not elligible	Chinese	currently married	Tertiary and above	≥USD1195	no	19-24 yo	2
918	2019-2021	35-45 yo	Not elligible	Malay	separated/divorced/w	Secondary	<USD1195	no	19-24 yo	1
919	2019-2021	35-45 yo	Not elligible	Malay	currently married	Secondary	<USD1195	no	19-24 yo	2
920	2019-2021	35-45 yo	Not elligible	Chinese	currently married	Tertiary and above	≥USD1195	no	25-29 yo	1
921	2019-2021	35-45 yo	Not elligible	Malay	currently married	Tertiary and above	<USD1195	no	19-24 yo	1
922	2019-2021	35-45 yo	Not elligible	Malay	currently married	Tertiary and above	<USD1195	no	25-29 yo	1
923	2019-2021	35-45 yo	Not elligible	Malay	currently married	Tertiary and above	≥USD1195	no	19-24 yo	2
924	2019-2021	35-45 yo	Not elligible	Malay	currently married	Tertiary and above	≥USD1195	no	25-29 yo	1
925	2019-2021	35-45 yo	Not elligible	Malay	currently married	Secondary	<USD1195	no	25-29 yo	1
926	2019-2021	35-45 yo	Not elligible	Chinese	currently married	Tertiary and above	≥USD1195	no	30 and above	2
927	2019-2021	35-45 yo	Not elligible	Malay	currently married	Secondary	<USD1195	no	25-29 yo	1
928	2019-2021	35-45 yo	Not elligible	Malay	currently married	Secondary	<USD1195	no	18 yo and below	1
929	2019-2021	35-45 yo	Not elligible	Malay	currently married	Secondary	≥USD1195	no	25-29 yo	1
930	2019-2021	35-45 yo	Not elligible	Malay	currently married	Secondary	≥USD1195	no	19-24 yo	1
931	2019-2021	35-45 yo	Not elligible	Malay	currently married	Secondary	<USD1195	no	19-24 yo	1
932	2019-2021	35-45 yo	Not elligible	Malay	currently married	Tertiary and above	<USD1195	no	30 and above	
933	2019-2021	35-45 yo	Not elligible	Malay	currently married	Tertiary and above	≥USD1195	yes	25-29 yo	1
934	2019-2021	35-45 yo	Not elligible	Malay	currently married	Tertiary and above	≥USD1195	no	19-24 yo	1

935	2019-2021	35-45 yo	Not elligible	Malay	currently married	Tertiary and above	≥USD1195	no	25-29 yo	1
936	2019-2021	35-45 yo	Not elligible	Malay	currently married	Tertiary and above	<USD1195	no	25-29 yo	1
937	2019-2021	35-45 yo	Not elligible	Malay	currently married	Secondary	≥USD1195	no	25-29 yo	1
938	2019-2021	35-45 yo	Not elligible	Malay	currently married	Tertiary and above	≥USD1195	no	25-29 yo	1
939	2019-2021	18-24 yo	catchup	Malay	currently married	Tertiary and above	<USD1195	yes	19-24 yo	1
940	2019-2021	35-45 yo	Not elligible	Other	currently married	Tertiary and above	≥USD1195	no	25-29 yo	1
941	2019-2021	35-45 yo	Not elligible	Other	currently married	Tertiary and above	≥USD1195	no	25-29 yo	1
942	2019-2021	35-45 yo	Not elligible	Chinese	currently married	Tertiary and above	≥USD1195	no	19-24 yo	3 and above
943	2019-2021	35-45 yo	Not elligible	Other	currently married	Tertiary and above	<USD1195	no	25-29 yo	1
944	2019-2021	35-45 yo	Not elligible	Other	currently married	None-primary	<USD1195	no	19-24 yo	1
945	2019-2021	35-45 yo	Not elligible	Other	currently married	Tertiary and above	≥USD1195	no	30 and above	1
946	2019-2021	35-45 yo	Not elligible	Other	currently married	Tertiary and above	≥USD1195	yes	19-24 yo	1
947	2019-2021	35-45 yo	Not elligible	Other	currently married	Tertiary and above	<USD1195	no	25-29 yo	1
948	2019-2021	35-45 yo	Not elligible	Other	currently married	Tertiary and above	<USD1195	no	25-29 yo	1
949	2019-2021	35-45 yo	Not elligible	Other	currently married	Tertiary and above	≥USD1195	no	19-24 yo	2
950	2019-2021	35-45 yo	Not elligible	Other	currently married	Tertiary and above	≥USD1195	yes	25-29 yo	1
951	2019-2021	35-45 yo	Not elligible	Other	currently married	Tertiary and above	<USD1195	no	25-29 yo	1
952	2019-2021	35-45 yo	Not elligible	Chinese	currently married	Tertiary and above	≥USD1195	yes	30 and above	
953	2019-2021	35-45 yo	Not elligible	Other	currently married	Secondary	<USD1195	no	19-24 yo	1
954	2019-2021	35-45 yo	Not elligible	Other	currently married	Tertiary and above	<USD1195	no	25-29 yo	1
955	2019-2021	35-45 yo	Not elligible	Other	currently married	Tertiary and above	≥USD1195	no	25-29 yo	1
956	2019-2021	35-45 yo	Not elligible	Other	separated/divorced/w	Tertiary and above	≥USD1195	no	19-24 yo	2
957	2019-2021	35-45 yo	Not elligible	Malay	currently married	Tertiary and above	≥USD1195	no	25-29 yo	1
958	2019-2021	35-45 yo	Not elligible	Malay	currently married	Tertiary and above	≥USD1195	no	25-29 yo	1
959	2019-2021	18-24 yo	school-based	Chinese	single	Tertiary and above	≥USD1195	no	19-24 yo	2
960	2019-2021	18-24 yo	school-based	Chinese	single	Tertiary and above	≥USD1195	yes	19-24 yo	2
961	2019-2021	18-24 yo	school-based	Chinese	single	Secondary	≥USD1195	yes		
962	2019-2021	18-24 yo	school-based	Chinese	single	Tertiary and above	<USD1195	yes		
963	2019-2021	18-24 yo	school-based	Chinese	single	Tertiary and above	<USD1195	yes	18 yo and below	1
964	2019-2021	35-45 yo	Not elligible	Indian	currently married	Tertiary and above	<USD1195	no	19-24 yo	1
965	2019-2021	18-24 yo	school-based	Indian	currently married	Secondary	<USD1195	yes	19-24 yo	2
966	2019-2021	35-45 yo	Not elligible	Malay	currently married	Tertiary and above	≥USD1195	no	25-29 yo	1
967	2019-2021	35-45 yo	Not elligible	Malay	currently married	Tertiary and above	≥USD1195	no	25-29 yo	1
968	2019-2021	35-45 yo	Not elligible	Indian	currently married	Secondary	<USD1195	no	19-24 yo	1
969	2019-2021	35-45 yo	Not elligible	Malay	currently married	Tertiary and above	≥USD1195	no	19-24 yo	1
970	2019-2021	18-24 yo	school-based	Other	single	Tertiary and above	<USD1195	no	18 yo and below	
971	2019-2021	18-24 yo	school-based	Chinese	single	Tertiary and above	≥USD1195	yes	18 yo and below	3 and above
972	2019-2021	18-24 yo	catchup	Chinese	single	Tertiary and above	<USD1195	no	18 yo and below	3 and above
973	2019-2021	18-24 yo	school-based	Chinese	single	Tertiary and above	≥USD1195	yes	18 yo and below	3 and above
974	2019-2021	18-24 yo	school-based	Chinese	single	Tertiary and above	≥USD1195	no	19-24 yo	1

975	2019-2021	18-24 yo	school-based	Chinese	single	Tertiary and above	<USD1195	yes	18 yo and below	1
976	2019-2021	35-45 yo	Not elligible	Malay	currently married	Secondary	≥USD1195	no	25-29 yo	1
977	2019-2021	35-45 yo	Not elligible	Malay	currently married	Tertiary and above	<USD1195	no	25-29 yo	1
978	2019-2021	18-24 yo	school-based	Indian	single	Tertiary and above	<USD1195	yes	19-24 yo	1
979	2019-2021	18-24 yo	catchup	Chinese	single	Tertiary and above	<USD1195	no	19-24 yo	1
980	2019-2021	35-45 yo	Not elligible	Malay	currently married	Tertiary and above	≥USD1195	no	19-24 yo	1
981	2019-2021	35-45 yo	Not elligible	Indian	currently married	Tertiary and above	≥USD1195	no		
982	2019-2021	18-24 yo	school-based	Chinese	single	Tertiary and above	≥USD1195	yes	18 yo and below	2
983	2019-2021	35-45 yo	Not elligible	Chinese	separated/divorced/w	Secondary	<USD1195	no	18 yo and below	3 and above
984	2019-2021	18-24 yo	school-based	Malay	single	Tertiary and above	≥USD1195	yes	18 yo and below	2
985	2019-2021	18-24 yo	school-based	Chinese	single	Tertiary and above	<USD1195	yes	19-24 yo	2
986	2019-2021	18-24 yo	school-based	Chinese	single	Tertiary and above	≥USD1195	yes	18 yo and below	1
987	2019-2021	18-24 yo	catchup	Chinese	single	Tertiary and above	≥USD1195	no	19-24 yo	1
988	2019-2021	18-24 yo	catchup	Malay	single	Tertiary and above	≥USD1195	yes	18 yo and below	3 and above
989	2019-2021	18-24 yo	school-based	Malay	single	Tertiary and above	≥USD1195	yes	19-24 yo	1
990	2019-2021	18-24 yo	catchup	Malay	single	Tertiary and above	<USD1195	yes	19-24 yo	1
991	2019-2021	18-24 yo	school-based	Chinese	single	Tertiary and above	≥USD1195	yes	19-24 yo	1
992	2019-2021	18-24 yo	school-based	Malay	currently married	Tertiary and above	<USD1195	yes	19-24 yo	1
993	2019-2021	18-24 yo	school-based	Chinese	single	Tertiary and above	≥USD1195	yes	18 yo and below	1
994	2019-2021	18-24 yo	school-based	Indian	single	Tertiary and above	≥USD1195	yes	19-24 yo	3 and above
995	2019-2021	18-24 yo	school-based	Chinese	single	Tertiary and above	≥USD1195	yes	19-24 yo	2
996	2019-2021	35-45 yo	Not elligible	Chinese	currently married	Tertiary and above	≥USD1195	yes	18 yo and below	2
997	2019-2021	35-45 yo	Not elligible	Indian	separated/divorced/w	Tertiary and above	≥USD1195	no	25-29 yo	1
998	2019-2021	35-45 yo	Not elligible	Malay	currently married	Tertiary and above	≥USD1195	yes	25-29 yo	1
999	2019-2021	35-45 yo	Not elligible	Malay	currently married	Tertiary and above	≥USD1195	no	19-24 yo	1
###	2019-2021	35-45 yo	Not elligible	Chinese	separated/divorced/w	Tertiary and above	≥USD1195	no	25-29 yo	3 and above
###	2019-2021	35-45 yo	Not elligible	Malay	currently married	Secondary	<USD1195	no	19-24 yo	1
###	2019-2021	35-45 yo	Not elligible	Malay	currently married	Tertiary and above	<USD1195	no	25-29 yo	1
###	2019-2021	35-45 yo	Not elligible	Chinese	currently married	Secondary	≥USD1195	no	25-29 yo	1
###	2019-2021	35-45 yo	Not elligible	Malay	currently married	Tertiary and above	≥USD1195	no	19-24 yo	1
###	2019-2021	18-24 yo	catchup	Malay	currently married	Secondary	<USD1195	yes	18 yo and below	1
###	2019-2021	35-45 yo	Not elligible	Malay	currently married	Tertiary and above	≥USD1195	no	18 yo and below	
###	2019-2021	18-24 yo	school-based	Chinese	single	Secondary	<USD1195	yes	18 yo and below	3 and above
###	2019-2021	35-45 yo	Not elligible	Chinese	currently married	Tertiary and above	≥USD1195	no		
###	2019-2021	35-45 yo	Not elligible	Chinese	single	Tertiary and above	<USD1195	yes	19-24 yo	1
###	2019-2021	35-45 yo	Not elligible	Indian	currently married	Tertiary and above	≥USD1195	no	30 and above	1
###	2019-2021	35-45 yo	Not elligible	Malay	currently married	Tertiary and above	≥USD1195	no	25-29 yo	1
###	2019-2021	35-45 yo	Not elligible	Malay	currently married	Tertiary and above	<USD1195	no	25-29 yo	1
###	2019-2021	35-45 yo	Not elligible	Malay	currently married	Tertiary and above	<USD1195	no	25-29 yo	1
###	2019-2021	18-24 yo	catchup	Malay	currently married	Tertiary and above	≥USD1195	no	19-24 yo	1

###	2019-2021	35-45 yo	Not elligible	Indian	currently married	Tertiary and above	≥USD1195	no	25-29 yo	1
###	2019-2021	35-45 yo	Not elligible	Malay	currently married	Tertiary and above	≥USD1195	no	30 and above	1
###	2019-2021	35-45 yo	Not elligible	Malay	currently married	Secondary	<USD1195	no	19-24 yo	2
###	2019-2021	18-24 yo	catchup	Malay	single	Tertiary and above	<USD1195	yes	18 yo and below	3 and above
###	2019-2021	35-45 yo	Not elligible	Malay	currently married	Tertiary and above	≥USD1195	no	19-24 yo	
###	2019-2021	35-45 yo	Not elligible	Malay	currently married	Secondary	<USD1195	no	19-24 yo	1
###	2019-2021	35-45 yo	Not elligible	Chinese	currently married	Tertiary and above	≥USD1195	no	19-24 yo	2
###	2019-2021	35-45 yo	Not elligible	Malay	currently married	Secondary	≥USD1195	no	18 yo and below	1
###	2019-2021	35-45 yo	Not elligible	Malay	currently married	Secondary	<USD1195	no	19-24 yo	1
###	2019-2021	35-45 yo	Not elligible	Malay	currently married	Tertiary and above	≥USD1195	no	19-24 yo	2
###	2019-2021	35-45 yo	Not elligible	Chinese	single	Tertiary and above	≥USD1195	yes		
###	2019-2021	35-45 yo	Not elligible	Chinese	currently married	Tertiary and above	≥USD1195	yes	25-29 yo	1
###	2019-2021	35-45 yo	Not elligible	Chinese	single	Tertiary and above	≥USD1195	yes	18 yo and below	3 and above
###	2019-2021	35-45 yo	Not elligible	Malay	separated/divorced/w	Secondary	<USD1195	no	19-24 yo	1
###	2019-2021	35-45 yo	Not elligible	Chinese	currently married	Secondary	≥USD1195	yes	19-24 yo	1
###	2019-2021	35-45 yo	Not elligible	Malay	currently married	Tertiary and above	≥USD1195	no	19-24 yo	1
###	2019-2021	35-45 yo	Not elligible	Malay	separated/divorced/w	Secondary	<USD1195	no	25-29 yo	1
###	2019-2021	35-45 yo	Not elligible	Malay	currently married	Tertiary and above	≥USD1195	no	25-29 yo	1
###	2019-2021	35-45 yo	Not elligible	Indian	currently married	Tertiary and above	≥USD1195	no	25-29 yo	1
###	2019-2021	35-45 yo	Not elligible	Indian	currently married	Secondary	≥USD1195	no	19-24 yo	1
###	2019-2021	35-45 yo	Not elligible	Malay	currently married	Tertiary and above	≥USD1195	no	30 and above	1
###	2019-2021	35-45 yo	Not elligible	Indian	currently married	Tertiary and above	<USD1195	no	25-29 yo	1
###	2019-2021	35-45 yo	Not elligible	Malay	currently married	Tertiary and above	<USD1195	no	25-29 yo	1
###	2019-2021	35-45 yo	Not elligible	Indian	currently married	Secondary	<USD1195	no	30 and above	1
###	2019-2021	35-45 yo	Not elligible	Malay	currently married	Tertiary and above	≥USD1195	no	19-24 yo	1
###	2019-2021	35-45 yo	Not elligible	Malay	currently married	Tertiary and above	≥USD1195	no	25-29 yo	1
###	2019-2021	35-45 yo	Not elligible	Malay	currently married	Tertiary and above	≥USD1195	no	25-29 yo	1
###	2019-2021	35-45 yo	Not elligible	Malay	currently married	Tertiary and above	<USD1195	no	25-29 yo	1
###	2019-2021	35-45 yo	Not elligible	Chinese	currently married	Tertiary and above	<USD1195	no	19-24 yo	3 and above
###	2019-2021	35-45 yo	Not elligible	Malay	currently married	Secondary	≥USD1195	yes	19-24 yo	1
###	2019-2021	35-45 yo	Not elligible	Indian	currently married	Tertiary and above	≥USD1195	yes	25-29 yo	1
###	2019-2021	35-45 yo	Not elligible	Malay	currently married	Tertiary and above	≥USD1195	no	19-24 yo	1
###	2019-2021	35-45 yo	Not elligible	Chinese	currently married	Tertiary and above	≥USD1195	yes	30 and above	1
###	2019-2021	35-45 yo	Not elligible	Malay	currently married	Tertiary and above	≥USD1195	no	18 yo and below	1
###	2019-2021	35-45 yo	Not elligible	Malay	currently married	Tertiary and above	≥USD1195	no	19-24 yo	1
###	2019-2021	35-45 yo	Not elligible	Malay	currently married	Tertiary and above	≥USD1195	no	25-29 yo	1
###	2019-2021	35-45 yo	Not elligible	Malay	currently married	Tertiary and above	≥USD1195	no	19-24 yo	1
###	2019-2021	35-45 yo	Not elligible	Chinese	currently married	Tertiary and above	≥USD1195	no	19-24 yo	1
###	2019-2021	35-45 yo	Not elligible	Malay	currently married	Tertiary and above	≥USD1195	no	25-29 yo	1
###	2019-2021	35-45 yo	Not elligible	Malay	currently married	Tertiary and above	≥USD1195	no	25-29 yo	1

###	2019-2021	35-45 yo	Not elligible	Chinese	currently married	Tertiary and above	≥USD1195	yes	19-24 yo	3 and above
###	2019-2021	35-45 yo	Not elligible	Indian	currently married	Tertiary and above	≥USD1195	no	25-29 yo	2
###	2019-2021	35-45 yo	Not elligible	Malay	currently married	Secondary	<USD1195	no	19-24 yo	1
###	2019-2021	35-45 yo	Not elligible	Malay	separated/divorced/w	Tertiary and above	<USD1195	no	30 and above	1
###	2019-2021	35-45 yo	Not elligible	Chinese	currently married	Tertiary and above	≥USD1195	no	25-29 yo	1
###	2019-2021	35-45 yo	Not elligible	Malay	currently married	Tertiary and above	≥USD1195	no	25-29 yo	1
###	2019-2021	35-45 yo	Not elligible	Chinese	single	Tertiary and above	≥USD1195	yes	30 and above	2
###	2019-2021	35-45 yo	Not elligible	Chinese	currently married	Tertiary and above	≥USD1195	yes	30 and above	1
###	2019-2021	35-45 yo	Not elligible	Chinese	currently married	Secondary	≥USD1195	no	19-24 yo	2
###	2019-2021	35-45 yo	Not elligible	Chinese	currently married	Secondary	<USD1195	no	18 yo and below	3 and above
###	2019-2021	35-45 yo	Not elligible	Malay	currently married	Secondary	<USD1195	no	19-24 yo	1
###	2019-2021	18-24 yo	catchup	Chinese	single	Tertiary and above	<USD1195	no	19-24 yo	2
###	2019-2021	35-45 yo	Not elligible	Malay	currently married	Tertiary and above	≥USD1195	no	25-29 yo	1
###	2019-2021	35-45 yo	Not elligible	Malay	currently married	Secondary	<USD1195	no	30 and above	1
###	2019-2021	35-45 yo	Not elligible	Chinese	currently married	Tertiary and above	≥USD1195	no	25-29 yo	2
###	2019-2021	35-45 yo	Not elligible	Malay	currently married	Tertiary and above	<USD1195	no	25-29 yo	1
###	2019-2021	35-45 yo	Not elligible	Chinese	single	Tertiary and above	<USD1195	no	19-24 yo	3 and above
###	2019-2021	35-45 yo	Not elligible	Chinese	currently married	Tertiary and above	≥USD1195	no	19-24 yo	1
###	2019-2021	35-45 yo	Not elligible	Malay	currently married	Secondary	<USD1195	no	25-29 yo	1
###	2019-2021	35-45 yo	Not elligible	Malay	currently married	Secondary	≥USD1195	no	19-24 yo	1
###	2019-2021	35-45 yo	Not elligible	Malay	currently married	Tertiary and above	≥USD1195	no	25-29 yo	1
###	2019-2021	35-45 yo	Not elligible	Indian	currently married	Tertiary and above	<USD1195	no	19-24 yo	3 and above
###	2019-2021	35-45 yo	Not elligible	Other	currently married	Tertiary and above	≥USD1195	no	30 and above	1
###	2019-2021	35-45 yo	Not elligible	Malay	currently married	Secondary	<USD1195	no	19-24 yo	1
###	2019-2021	35-45 yo	Not elligible	Indian	single	Tertiary and above	<USD1195	yes		
###	2019-2021	35-45 yo	Not elligible	Malay	currently married	Tertiary and above	≥USD1195	no	19-24 yo	1
###	2019-2021	35-45 yo	Not elligible	Indian	currently married	Tertiary and above	≥USD1195	no	25-29 yo	1
###	2019-2021	35-45 yo	Not elligible	Malay	currently married	Tertiary and above	≥USD1195	no	25-29 yo	1
###	2019-2021	35-45 yo	Not elligible	Malay	currently married	Tertiary and above	<USD1195	no	30 and above	1
###	2019-2021	35-45 yo	Not elligible	Malay	currently married	Secondary	≥USD1195	no	19-24 yo	1
###	2019-2021	35-45 yo	Not elligible	Malay	currently married	Secondary	≥USD1195	no	19-24 yo	1
###	2019-2021	35-45 yo	Not elligible	Malay	currently married	Secondary	<USD1195	no	30 and above	1
###	2019-2021	35-45 yo	Not elligible	Indian	currently married	Tertiary and above	<USD1195	no	25-29 yo	1
###	2019-2021	35-45 yo	Not elligible	Malay	currently married	Tertiary and above	≥USD1195	no	19-24 yo	1
###	2019-2021	35-45 yo	Not elligible	Malay	currently married	Tertiary and above	≥USD1195	no	25-29 yo	1
###	2019-2021	35-45 yo	Not elligible	Malay	currently married	Tertiary and above	≥USD1195	no	25-29 yo	1
###	2019-2021	35-45 yo	Not elligible	Malay	currently married	Tertiary and above	<USD1195	no	25-29 yo	1
###	2019-2021	35-45 yo	Not elligible	Malay	currently married	Secondary	<USD1195	no	18 yo and below	1
###	2019-2021	35-45 yo	Not elligible	Malay	currently married	Tertiary and above	≥USD1195	no	25-29 yo	1
###	2019-2021	35-45 yo	Not elligible	Malay	currently married	Tertiary and above	≥USD1195	no	19-24 yo	1

###	2019-2021	35-45 yo	Not elligible	Indian	currently married	Secondary	≥USD1195	no	19-24 yo	1
###	2019-2021	35-45 yo	Not elligible	Malay	currently married	Tertiary and above	≥USD1195	no	25-29 yo	1
###	2019-2021	35-45 yo	Not elligible	Malay	separated/divorced/w	Secondary	<USD1195	no	18 yo and below	3 and above
###	2019-2021	35-45 yo	Not elligible	Malay	currently married	Secondary	<USD1195	no	19-24 yo	1
###	2019-2021	35-45 yo	Not elligible	Chinese	currently married	Secondary	≥USD1195	no	18 yo and below	3 and above
###	2019-2021	35-45 yo	Not elligible	Malay	currently married	Tertiary and above	≥USD1195	no	25-29 yo	1
###	2019-2021	35-45 yo	Not elligible	Malay	currently married	Secondary	<USD1195	no	19-24 yo	2
###	2019-2021	35-45 yo	Not elligible	Malay	currently married	Tertiary and above	<USD1195	no	25-29 yo	1
###	2019-2021	35-45 yo	Not elligible	Other	currently married	Tertiary and above	<USD1195	no	30 and above	2
###	2019-2021	35-45 yo	Not elligible	Malay	currently married	Secondary	<USD1195	no	19-24 yo	1
###	2019-2021	35-45 yo	Not elligible	Chinese	currently married	Tertiary and above	≥USD1195	no	25-29 yo	1
###	2019-2021	35-45 yo	Not elligible	Malay	currently married	Tertiary and above	≥USD1195	no	25-29 yo	1
###	2019-2021	35-45 yo	Not elligible	Malay	currently married	Tertiary and above	≥USD1195	no	25-29 yo	1
###	2019-2021	35-45 yo	Not elligible	Chinese	currently married	Tertiary and above	≥USD1195	no	19-24 yo	2
###	2019-2021	35-45 yo	Not elligible	Malay	currently married	Tertiary and above	≥USD1195	no	25-29 yo	1
###	2019-2021	35-45 yo	Not elligible	Malay	currently married	Tertiary and above	≥USD1195	no	19-24 yo	1
###	2019-2021	35-45 yo	Not elligible	Chinese	currently married	Tertiary and above	≥USD1195	no	25-29 yo	1
###	2019-2021	35-45 yo	Not elligible	Malay	currently married	Tertiary and above	≥USD1195	no	19-24 yo	1
###	2019-2021	35-45 yo	Not elligible	Indian	currently married	Tertiary and above	<USD1195	no	25-29 yo	1
###	2019-2021	35-45 yo	Not elligible	Chinese	separated/divorced/w	Secondary	<USD1195	no	19-24 yo	2
###	2019-2021	35-45 yo	Not elligible	Chinese	currently married	Tertiary and above	≥USD1195	no	18 yo and below	
###	2019-2021	35-45 yo	Not elligible	Malay	currently married	Tertiary and above	<USD1195	no	25-29 yo	1
###	2019-2021	35-45 yo	Not elligible	Malay	currently married	Tertiary and above	≥USD1195	no	25-29 yo	1
###	2019-2021	35-45 yo	Not elligible	Malay	currently married	Tertiary and above	<USD1195	no	30 and above	1
###	2019-2021	35-45 yo	Not elligible	Malay	currently married	Tertiary and above	<USD1195	no	19-24 yo	2
###	2019-2021	18-24 yo	school-based	Malay	currently married	Secondary	<USD1195	yes	19-24 yo	1
###	2019-2021	35-45 yo	Not elligible	Malay	currently married	Tertiary and above	≥USD1195	no	19-24 yo	1
###	2019-2021	35-45 yo	Not elligible	Malay	currently married	Tertiary and above	≥USD1195	no	25-29 yo	1
###	2019-2021	35-45 yo	Not elligible	Malay	currently married	Tertiary and above	≥USD1195	no	30 and above	1
###	2019-2021	35-45 yo	Not elligible	Malay	currently married	Tertiary and above	<USD1195	no	25-29 yo	1
###	2019-2021	35-45 yo	Not elligible	Malay	currently married	Tertiary and above	≥USD1195	no	25-29 yo	1
###	2019-2021	35-45 yo	Not elligible	Indian	currently married	Tertiary and above	≥USD1195	no	25-29 yo	1
###	2019-2021	35-45 yo	Not elligible	Indian	currently married	Secondary	<USD1195	no	25-29 yo	1
###	2019-2021	35-45 yo	Not elligible	Malay	currently married	Tertiary and above	≥USD1195	no	25-29 yo	1
###	2019-2021	18-24 yo	school-based	Malay	currently married	Secondary	<USD1195	yes	18 yo and below	1
###	2019-2021	35-45 yo	Not elligible	Malay	currently married	Tertiary and above	≥USD1195	no	25-29 yo	1
###	2019-2021	35-45 yo	Not elligible	Malay	currently married	Tertiary and above	≥USD1195	no	25-29 yo	1
###	2019-2021	35-45 yo	Not elligible	Malay	currently married	Secondary	<USD1195	no	25-29 yo	1
###	2019-2021	35-45 yo	Not elligible	Malay	currently married	Tertiary and above	<USD1195	no	25-29 yo	1
###	2019-2021	35-45 yo	Not elligible	Chinese	currently married	Tertiary and above	≥USD1195	no	19-24 yo	2

###	2019-2021	35-45 yo	Not elligible	Chinese	currently married	Secondary	<USD1195	no	18 yo and below	2
###	2019-2021	35-45 yo	Not elligible	Malay	currently married	Tertiary and above	≥USD1195	no	25-29 yo	1
###	2019-2021	35-45 yo	Not elligible	Malay	currently married	Tertiary and above	≥USD1195	no	25-29 yo	1
###	2019-2021	35-45 yo	Not elligible	Malay	currently married	Tertiary and above	≥USD1195	no	19-24 yo	2
###	2019-2021	35-45 yo	Not elligible	Malay	currently married	Tertiary and above	≥USD1195	no	25-29 yo	1
###	2019-2021	35-45 yo	Not elligible	Malay	currently married	Tertiary and above	≥USD1195	no	25-29 yo	1
###	2019-2021	35-45 yo	Not elligible	Malay	currently married	Tertiary and above	≥USD1195	no	19-24 yo	1
###	2019-2021	35-45 yo	Not elligible	Chinese	currently married	Tertiary and above	≥USD1195	yes	18 yo and below	3 and above
###	2019-2021	35-45 yo	Not elligible	Chinese	currently married	Secondary	≥USD1195	no	19-24 yo	3 and above
###	2019-2021	35-45 yo	Not elligible	Chinese	currently married	Tertiary and above	≥USD1195	no	25-29 yo	1
###	2019-2021	35-45 yo	Not elligible	Chinese	currently married	Tertiary and above	≥USD1195	no	19-24 yo	1
###	2019-2021	35-45 yo	Not elligible	Malay	currently married	Tertiary and above	<USD1195	no	25-29 yo	1
###	2019-2021	35-45 yo	Not elligible	Malay	currently married	Secondary	<USD1195	no	25-29 yo	1
###	2019-2021	35-45 yo	Not elligible	Malay	currently married	Tertiary and above	≥USD1195	no	25-29 yo	1
###	2019-2021	35-45 yo	Not elligible	Chinese	currently married	Tertiary and above	≥USD1195	no	19-24 yo	1
###	2019-2021	35-45 yo	Not elligible	Indian	currently married	Tertiary and above	<USD1195	no	19-24 yo	2
###	2019-2021	35-45 yo	Not elligible	Malay	currently married	Secondary	<USD1195	no	19-24 yo	3 and above
###	2019-2021	35-45 yo	Not elligible	Malay	currently married	Tertiary and above	<USD1195	no	25-29 yo	1
###	2019-2021	35-45 yo	Not elligible	Chinese	currently married	Tertiary and above	≥USD1195	yes	19-24 yo	3 and above
###	2019-2021	35-45 yo	Not elligible	Malay	currently married	Tertiary and above	<USD1195	no	25-29 yo	1
###	2019-2021	18-24 yo	school-based	Malay	currently married	Secondary	<USD1195	yes	19-24 yo	1
###	2019-2021	35-45 yo	Not elligible	Malay	currently married	Secondary	<USD1195	no	30 and above	1
###	2019-2021	35-45 yo	Not elligible	Chinese	currently married	Tertiary and above	≥USD1195	no	19-24 yo	1
###	2019-2021	18-24 yo	school-based	Malay	currently married	Secondary	<USD1195	yes	19-24 yo	1
###	2019-2021	35-45 yo	Not elligible	Malay	currently married	Tertiary and above	≥USD1195	no	25-29 yo	1
###	2019-2021	35-45 yo	Not elligible	Malay	currently married	Secondary	<USD1195	no	25-29 yo	1
###	2019-2021	35-45 yo	Not elligible	Malay	currently married	Tertiary and above	≥USD1195	no	25-29 yo	1
###	2019-2021	35-45 yo	Not elligible	Malay	currently married	Secondary	<USD1195	no	19-24 yo	1
###	2019-2021	35-45 yo	Not elligible	Chinese	currently married	Tertiary and above	≥USD1195	no	18 yo and below	2
###	2019-2021	35-45 yo	Not elligible	Malay	currently married	Tertiary and above	≥USD1195	no	19-24 yo	2
###	2019-2021	35-45 yo	Not elligible	Malay	currently married	Tertiary and above	<USD1195	no	25-29 yo	1
###	2019-2021	35-45 yo	Not elligible	Malay	currently married	Secondary	<USD1195	no	19-24 yo	1
###	2019-2021	35-45 yo	Not elligible	Malay	currently married	Tertiary and above	≥USD1195	no	25-29 yo	1
###	2019-2021	35-45 yo	Not elligible	Chinese	currently married	Tertiary and above	<USD1195	no	19-24 yo	3 and above
###	2019-2021	35-45 yo	Not elligible	Malay	currently married	Secondary	<USD1195	no	25-29 yo	1
###	2019-2021	35-45 yo	Not elligible	Malay	currently married	Tertiary and above	≥USD1195	no	19-24 yo	
###	2019-2021	35-45 yo	Not elligible	Malay	currently married	Tertiary and above	≥USD1195	no	19-24 yo	1
###	2019-2021	18-24 yo	school-based	Malay	currently married	None-primary	<USD1195	yes	18 yo and below	1
###	2019-2021	35-45 yo	Not elligible	Malay	currently married	Tertiary and above	<USD1195	no	25-29 yo	1
###	2019-2021	35-45 yo	Not elligible	Malay	currently married	Secondary	<USD1195	no	30 and above	1

###	2019-2021	35-45 yo	Not elligible	Malay	currently married	Secondary	<USD1195	no	25-29 yo	1
###	2019-2021	18-24 yo	school-based	Indian	currently married	Secondary	≥USD1195	yes	19-24 yo	1
###	2019-2021	35-45 yo	Not elligible	Malay	currently married	Secondary	≥USD1195	no	25-29 yo	1
###	2019-2021	35-45 yo	Not elligible	Malay	currently married	Tertiary and above	≥USD1195	no	19-24 yo	1
###	2019-2021	35-45 yo	Not elligible	Chinese	currently married	Secondary	≥USD1195	no	25-29 yo	1
###	2019-2021	35-45 yo	Not elligible	Malay	currently married	Tertiary and above	≥USD1195	no	25-29 yo	1
###	2019-2021	35-45 yo	Not elligible	Malay	currently married	Secondary	<USD1195	no	25-29 yo	1
###	2019-2021	35-45 yo	Not elligible	Chinese	currently married	Tertiary and above	≥USD1195	no	25-29 yo	1
###	2019-2021	35-45 yo	Not elligible	Chinese	currently married	None-primary	<USD1195	no	19-24 yo	2
###	2019-2021	35-45 yo	Not elligible	Malay	currently married	Secondary	<USD1195	no	19-24 yo	1
###	2019-2021	35-45 yo	Not elligible	Other	currently married	Secondary	<USD1195	no	19-24 yo	1
###	2019-2021	35-45 yo	Not elligible	Malay	currently married	Secondary	<USD1195	no	19-24 yo	1
###	2019-2021	35-45 yo	Not elligible	Malay	currently married	None-primary	<USD1195	no	19-24 yo	2
###	2019-2021	35-45 yo	Not elligible	Malay	currently married	Tertiary and above	≥USD1195	no	19-24 yo	1
###	2019-2021	35-45 yo	Not elligible	Malay	currently married	Tertiary and above	≥USD1195	no	19-24 yo	1
###	2019-2021	35-45 yo	Not elligible	Malay	currently married	Secondary	≥USD1195	no	25-29 yo	1
###	2019-2021	35-45 yo	Not elligible	Malay	currently married	Tertiary and above	<USD1195	no	25-29 yo	1
###	2019-2021	35-45 yo	Not elligible	Malay	currently married	Tertiary and above	<USD1195	no	25-29 yo	1
###	2019-2021	35-45 yo	Not elligible	Chinese	currently married	Tertiary and above	≥USD1195	yes	25-29 yo	1
###	2019-2021	18-24 yo	catchup	Malay	currently married	Secondary	<USD1195	yes	19-24 yo	1
###	2019-2021	18-24 yo	school-based	Malay	currently married	Secondary	<USD1195	yes	19-24 yo	1
###	2019-2021	35-45 yo	Not elligible	Malay	currently married	Tertiary and above	<USD1195	no	25-29 yo	2
###	2019-2021	35-45 yo	Not elligible	Malay	currently married	Tertiary and above	≥USD1195	no	25-29 yo	1
###	2019-2021	35-45 yo	Not elligible	Chinese	currently married	Tertiary and above	≥USD1195	no	19-24 yo	1
###	2019-2021	18-24 yo	school-based	Malay	currently married	Secondary	<USD1195	yes	18 yo and below	2
###	2019-2021	18-24 yo	school-based	Malay	currently married	Secondary	<USD1195	yes	18 yo and below	1
###	2019-2021	35-45 yo	Not elligible	Malay	currently married	Tertiary and above	≥USD1195	yes	19-24 yo	1
###	2019-2021	35-45 yo	Not elligible	Malay	currently married	Secondary	<USD1195	no	19-24 yo	1
###	2019-2021	35-45 yo	Not elligible	Malay	currently married	Tertiary and above	<USD1195	no	25-29 yo	1
###	2019-2021	18-24 yo	catchup	Malay	currently married	Secondary	≥USD1195	no	19-24 yo	3 and above
###	2019-2021	35-45 yo	Not elligible	Malay	currently married	Secondary	≥USD1195	no	19-24 yo	1
###	2019-2021	35-45 yo	Not elligible	Indian	currently married	Tertiary and above	<USD1195	no	25-29 yo	1
###	2019-2021	35-45 yo	Not elligible	Chinese	currently married	Tertiary and above	≥USD1195	no	19-24 yo	2
###	2019-2021	35-45 yo	Not elligible	Malay	currently married	Secondary	≥USD1195	no	25-29 yo	1
###	2019-2021	18-24 yo	school-based	Malay	currently married	Secondary	≥USD1195	yes	18 yo and below	1
###	2019-2021	35-45 yo	Not elligible	Malay	currently married	Secondary	≥USD1195	no	25-29 yo	1
###	2019-2021	35-45 yo	Not elligible	Malay	currently married	Secondary	<USD1195	no	19-24 yo	1
###	2019-2021	35-45 yo	Not elligible	Malay	currently married	Secondary	<USD1195	no	18 yo and below	1
###	2019-2021	35-45 yo	Not elligible	Malay	currently married	Secondary	<USD1195	no	25-29 yo	1
###	2019-2021	18-24 yo	school-based	Other	currently married	None-primary	<USD1195	no	18 yo and below	1

###	2019-2021	35-45 yo	Not elligible	Malay	currently married	Tertiary and above	<USD1195	no	19-24 yo	1
###	2019-2021	35-45 yo	Not elligible	Malay	currently married	Tertiary and above	<USD1195	no	30 and above	1
###	2019-2021	35-45 yo	Not elligible	Malay	currently married	Secondary	≥USD1195	no	25-29 yo	1
###	2019-2021	18-24 yo	school-based	Malay	currently married	Secondary	<USD1195	yes	18 yo and below	3 and above
###	2019-2021	35-45 yo	Not elligible	Malay	currently married	Tertiary and above	<USD1195	no	25-29 yo	1
###	2019-2021	35-45 yo	Not elligible	Malay	currently married	Tertiary and above	<USD1195	no	25-29 yo	1
###	2019-2021	35-45 yo	Not elligible	Malay	currently married	Secondary	<USD1195	no	30 and above	1
###	2019-2021	35-45 yo	Not elligible	Chinese	currently married	Secondary	≥USD1195	no	19-24 yo	2
###	2019-2021	35-45 yo	Not elligible	Malay	currently married	Tertiary and above	≥USD1195	no	30 and above	1
###	2019-2021	35-45 yo	Not elligible	Chinese	currently married	Tertiary and above	≥USD1195	no	30 and above	1
###	2019-2021	18-24 yo	catchup	Malay	currently married	Tertiary and above	<USD1195	yes	19-24 yo	1
###	2019-2021	35-45 yo	Not elligible	Malay	currently married	None-primary	<USD1195	no	19-24 yo	1
###	2019-2021	35-45 yo	Not elligible	Indian	currently married	Secondary	<USD1195	no	25-29 yo	1
###	2019-2021	35-45 yo	Not elligible	Chinese	currently married	Tertiary and above	≥USD1195	no	18 yo and below	1
###	2019-2021	18-24 yo	school-based	Malay	currently married	Secondary	<USD1195	yes	18 yo and below	3 and above
###	2019-2021	35-45 yo	Not elligible	Chinese	separated/divorced/w	Secondary	≥USD1195	no	18 yo and below	3 and above
###	2019-2021	18-24 yo	catchup	Malay	currently married	Tertiary and above	<USD1195	yes	19-24 yo	3 and above
###	2019-2021	35-45 yo	Not elligible	Malay	currently married	Tertiary and above	≥USD1195	no	25-29 yo	1
###	2019-2021	18-24 yo	school-based	Chinese	single	Secondary	<USD1195	yes	18 yo and below	1
###	2019-2021	35-45 yo	Not elligible	Indian	currently married	Tertiary and above	≥USD1195	no	19-24 yo	1
###	2019-2021	35-45 yo	Not elligible	Malay	currently married	Tertiary and above	<USD1195	no	19-24 yo	1
###	2019-2021	35-45 yo	Not elligible	Malay	currently married	Secondary	<USD1195	no	19-24 yo	2
###	2019-2021	35-45 yo	Not elligible	Chinese	separated/divorced/w	Tertiary and above	<USD1195	no	19-24 yo	3 and above
###	2019-2021	35-45 yo	Not elligible	Malay	currently married	Tertiary and above	≥USD1195	no	19-24 yo	1
###	2019-2021	35-45 yo	Not elligible	Malay	currently married	Secondary	<USD1195	no	25-29 yo	1
###	2019-2021	18-24 yo	school-based	Chinese	currently married	Secondary	≥USD1195	no	18 yo and below	3 and above
###	2019-2021	35-45 yo	Not elligible	Malay	currently married	Tertiary and above	≥USD1195	no	19-24 yo	1
###	2019-2021	18-24 yo	school-based	Malay	currently married	Secondary	<USD1195	yes	18 yo and below	1
###	2019-2021	35-45 yo	Not elligible	Indian	currently married	Secondary	<USD1195	no	19-24 yo	1
###	2019-2021	35-45 yo	Not elligible	Malay	currently married	Secondary	<USD1195	no	25-29 yo	1
###	2019-2021	35-45 yo	Not elligible	Malay	currently married	Secondary	≥USD1195	no	19-24 yo	2
###	2019-2021	35-45 yo	Not elligible	Malay	currently married	Secondary	≥USD1195	no	18 yo and below	1
###	2019-2021	35-45 yo	Not elligible	Chinese	currently married	Tertiary and above	≥USD1195	no	18 yo and below	3 and above
###	2019-2021	18-24 yo	school-based	Chinese	single	Tertiary and above	≥USD1195	yes	19-24 yo	1
###	2019-2021	35-45 yo	Not elligible	Malay	currently married	Tertiary and above	<USD1195	no	25-29 yo	1
###	2019-2021	35-45 yo	Not elligible	Chinese	currently married	Tertiary and above	≥USD1195	no	18 yo and below	2
###	2019-2021	18-24 yo	school-based	Malay	currently married	Secondary	<USD1195	yes	18 yo and below	3 and above
###	2019-2021	35-45 yo	Not elligible	Chinese	currently married	Tertiary and above	<USD1195	no	19-24 yo	2
###	2019-2021	18-24 yo	catchup	Malay	currently married	Tertiary and above	<USD1195	yes	19-24 yo	1
###	2019-2021	35-45 yo	Not elligible	Malay	currently married	Tertiary and above	≥USD1195	no	19-24 yo	2

###	2019-2021	35-45 yo	Not elligible	Malay	currently married	Secondary	<USD1195	no	19-24 yo	1
###	2019-2021	35-45 yo	Not elligible	Malay	currently married	Tertiary and above	<USD1195	no	19-24 yo	1
###	2019-2021	35-45 yo	Not elligible	Malay	currently married	Tertiary and above	≥USD1195	no	30 and above	1
###	2019-2021	35-45 yo	Not elligible	Other	separated/divorced/w	Secondary	<USD1195	no	19-24 yo	
###	2019-2021	35-45 yo	Not elligible	Malay	currently married	Secondary	<USD1195	no	19-24 yo	1
###	2019-2021	35-45 yo	Not elligible	Indian	currently married	Secondary	<USD1195	no	19-24 yo	1
###	2019-2021	35-45 yo	Not elligible	Malay	currently married	Secondary	<USD1195	no	19-24 yo	1
###	2019-2021	35-45 yo	Not elligible	Other	currently married	Tertiary and above	≥USD1195	no	25-29 yo	1
###	2019-2021	35-45 yo	Not elligible	Chinese	currently married	Tertiary and above	≥USD1195	no	19-24 yo	1
###	2019-2021	35-45 yo	Not elligible	Malay	currently married	Tertiary and above	≥USD1195	no	25-29 yo	1
###	2019-2021	35-45 yo	Not elligible	Malay	currently married	Secondary	≥USD1195	no	25-29 yo	1
###	2019-2021	35-45 yo	Not elligible	Chinese	currently married	Tertiary and above	≥USD1195	yes	30 and above	1
###	2019-2021	35-45 yo	Not elligible	Malay	currently married	Tertiary and above	≥USD1195	no	19-24 yo	1
###	2019-2021	35-45 yo	Not elligible	Chinese	currently married	Tertiary and above	≥USD1195	no	19-24 yo	1
###	2019-2021	35-45 yo	Not elligible	Chinese	currently married	Tertiary and above	≥USD1195	no	19-24 yo	1
###	2019-2021	35-45 yo	Not elligible	Malay	currently married	Tertiary and above	≥USD1195	no	25-29 yo	1
###	2019-2021	35-45 yo	Not elligible	Other	currently married	None-primary	<USD1195	no	25-29 yo	1
###	2019-2021	18-24 yo	school-based	Malay	currently married	Secondary	<USD1195	yes	18 yo and below	3 and above
###	2019-2021	35-45 yo	Not elligible	Malay	currently married	Tertiary and above	≥USD1195	no	19-24 yo	1
###	2019-2021	35-45 yo	Not elligible	Malay	currently married	Secondary	<USD1195	no	18 yo and below	2
###	2019-2021	18-24 yo	catchup	Malay	currently married	Secondary	<USD1195	yes	18 yo and below	2
###	2019-2021	35-45 yo	Not elligible	Malay	currently married	Secondary	<USD1195	no	19-24 yo	1
###	2019-2021	35-45 yo	Not elligible	Malay	currently married	Tertiary and above	≥USD1195	yes	25-29 yo	1
###	2019-2021	35-45 yo	Not elligible	Malay	currently married	Secondary	<USD1195	no	25-29 yo	1
###	2019-2021	35-45 yo	Not elligible	Malay	currently married	Tertiary and above	≥USD1195	no	30 and above	
###	2019-2021	35-45 yo	Not elligible	Malay	currently married	Tertiary and above	≥USD1195	yes	25-29 yo	1
###	2019-2021	18-24 yo	catchup	Malay	currently married	Secondary	<USD1195	no	19-24 yo	1
###	2019-2021	35-45 yo	Not elligible	Malay	currently married	Tertiary and above	<USD1195	no	25-29 yo	1
###	2019-2021	35-45 yo	Not elligible	Malay	currently married	Tertiary and above	<USD1195	no	25-29 yo	1
###	2019-2021	18-24 yo	school-based	Malay	currently married	Secondary	<USD1195	no	19-24 yo	1
###	2019-2021	35-45 yo	Not elligible	Indian	currently married	Tertiary and above	≥USD1195	no	25-29 yo	1
###	2019-2021	35-45 yo	Not elligible	Malay	currently married	Tertiary and above	<USD1195	no	25-29 yo	1
###	2019-2021	35-45 yo	Not elligible	Malay	currently married	Tertiary and above	<USD1195	no	25-29 yo	1
###	2019-2021	35-45 yo	Not elligible	Malay	currently married	Tertiary and above	<USD1195	no	25-29 yo	1
###	2019-2021	18-24 yo	school-based	Malay	separated/divorced/w	Secondary	<USD1195	no	18 yo and below	3 and above
###	2019-2021	18-24 yo	school-based	Malay	currently married	Secondary	<USD1195	yes	18 yo and below	1
###	2019-2021	35-45 yo	Not elligible	Malay	currently married	Secondary	<USD1195	no	19-24 yo	1
###	2019-2021	35-45 yo	Not elligible	Malay	currently married	Tertiary and above	≥USD1195	no	19-24 yo	1
###	2019-2021	35-45 yo	Not elligible	Malay	currently married	Tertiary and above	<USD1195	no	30 and above	1
###	2019-2021	35-45 yo	Not elligible	Malay	currently married	Secondary	≥USD1195	no	19-24 yo	1

###	2019-2021	35-45 yo	Not elligible	Chinese	currently married	Secondary	<USD1195	no	25-29 yo	2
###	2019-2021	35-45 yo	Not elligible	Indian	currently married	Tertiary and above	<USD1195	no	19-24 yo	1
###	2019-2021	18-24 yo	school-based	Malay	currently married	Secondary	<USD1195	yes	19-24 yo	1
###	2019-2021	35-45 yo	Not elligible	Malay	currently married	Tertiary and above	<USD1195	no	25-29 yo	1
###	2019-2021	35-45 yo	Not elligible	Chinese	currently married	Tertiary and above	<USD1195	no	19-24 yo	3 and above
###	2019-2021	35-45 yo	Not elligible	Indian	currently married	Tertiary and above	≥USD1195	no	25-29 yo	1
###	2019-2021	18-24 yo	catchup	Other	single	Tertiary and above	<USD1195	no	19-24 yo	2
###	2019-2021	35-45 yo	Not elligible	Malay	currently married	Tertiary and above	≥USD1195	no	25-29 yo	1
###	2019-2021	35-45 yo	Not elligible	Malay	currently married	Tertiary and above	≥USD1195	no	25-29 yo	1
###	2019-2021	35-45 yo	Not elligible	Malay	currently married	Secondary	<USD1195	no	25-29 yo	1
###	2019-2021	35-45 yo	Not elligible	Chinese	currently married	Secondary	≥USD1195	no	19-24 yo	3 and above
###	2019-2021	35-45 yo	Not elligible	Malay	currently married	Secondary	<USD1195	no	19-24 yo	1
###	2019-2021	35-45 yo	Not elligible	Malay	currently married	Secondary	<USD1195	no	19-24 yo	1
###	2019-2021	18-24 yo	catchup	Malay	currently married	Secondary	<USD1195	yes	19-24 yo	1
###	2019-2021	35-45 yo	Not elligible	Chinese	currently married	Tertiary and above	≥USD1195	yes	19-24 yo	1
###	2019-2021	35-45 yo	Not elligible	Chinese	currently married	Secondary	<USD1195	no	25-29 yo	1
###	2019-2021	35-45 yo	Not elligible	Malay	currently married	Secondary	<USD1195	no	19-24 yo	3 and above
###	2019-2021	35-45 yo	Not elligible	Chinese	currently married	Tertiary and above	≥USD1195	no	25-29 yo	1
###	2019-2021	35-45 yo	Not elligible	Malay	currently married	Secondary	<USD1195	no	30 and above	1
###	2019-2021	35-45 yo	Not elligible	Chinese	currently married	Tertiary and above	≥USD1195	no	19-24 yo	1
###	2019-2021	35-45 yo	Not elligible	Chinese	currently married	Secondary	≥USD1195	no	18 yo and below	2
###	2019-2021	35-45 yo	Not elligible	Indian	currently married	Secondary	<USD1195	no	19-24 yo	1
###	2019-2021	35-45 yo	Not elligible	Malay	currently married	Secondary	<USD1195	no	19-24 yo	1
###	2019-2021	35-45 yo	Not elligible	Malay	currently married	Tertiary and above	<USD1195	no	19-24 yo	1
###	2019-2021	35-45 yo	Not elligible	Malay	currently married	None-primary	<USD1195	no	18 yo and below	3 and above
###	2019-2021	35-45 yo	Not elligible	Malay	currently married	Secondary	≥USD1195	no	19-24 yo	1
###	2019-2021	35-45 yo	Not elligible	Malay	currently married	Secondary	<USD1195	no	30 and above	1
###	2019-2021	35-45 yo	Not elligible	Malay	currently married	Tertiary and above	<USD1195	no	30 and above	1
###	2019-2021	35-45 yo	Not elligible	Malay	currently married	Tertiary and above	≥USD1195	no	25-29 yo	1
###	2019-2021	35-45 yo	Not elligible	Malay	currently married	Tertiary and above	≥USD1195	no	19-24 yo	1
###	2019-2021	35-45 yo	Not elligible	Malay	currently married	Tertiary and above	≥USD1195	no	19-24 yo	1
###	2019-2021	35-45 yo	Not elligible	Malay	currently married	Tertiary and above	≥USD1195	no	25-29 yo	1
###	2019-2021	18-24 yo	school-based	Malay	currently married	Tertiary and above	≥USD1195	yes	19-24 yo	1
###	2019-2021	35-45 yo	Not elligible	Malay	currently married	Tertiary and above	<USD1195	no	25-29 yo	1
###	2019-2021	35-45 yo	Not elligible	Indian	currently married	Tertiary and above	<USD1195	no	25-29 yo	1
###	2019-2021	35-45 yo	Not elligible	Malay	currently married	Secondary	<USD1195	no	30 and above	2
###	2019-2021	35-45 yo	Not elligible	Malay	currently married	Secondary	<USD1195	no	19-24 yo	1
###	2019-2021	35-45 yo	Not elligible	Chinese	currently married	Tertiary and above	≥USD1195	no	25-29 yo	1
###	2019-2021	35-45 yo	Not elligible	Malay	currently married	Secondary	<USD1195	no	30 and above	1
###	2019-2021	35-45 yo	Not elligible	Malay	currently married	Tertiary and above	≥USD1195	no	25-29 yo	1

###	2019-2021	35-45 yo	Not elligible	Malay	currently married	Tertiary and above	<USD1195	no	25-29 yo	1
###	2019-2021	18-24 yo	catchup	Malay	currently married	Secondary	<USD1195	no	19-24 yo	1
###	2019-2021	18-24 yo	catchup	Malay	currently married	Secondary	<USD1195	no	19-24 yo	1
###	2019-2021	35-45 yo	Not elligible	Chinese	currently married	Tertiary and above	<USD1195	no	19-24 yo	1
###	2019-2021	18-24 yo	school-based	Malay	currently married	Secondary	<USD1195	yes	19-24 yo	1
###	2019-2021	35-45 yo	Not elligible	Malay	currently married	Secondary	<USD1195	no	19-24 yo	1
###	2019-2021	35-45 yo	Not elligible	Malay	currently married	Tertiary and above	≥USD1195	no	30 and above	1
###	2019-2021	35-45 yo	Not elligible	Indian	currently married	Secondary	<USD1195	no	19-24 yo	1
###	2019-2021	35-45 yo	Not elligible	Malay	currently married	Secondary	<USD1195	no	25-29 yo	1
###	2019-2021	35-45 yo	Not elligible	Malay	currently married	Tertiary and above	≥USD1195	no	25-29 yo	1
###	2019-2021	35-45 yo	Not elligible	Malay	currently married	Tertiary and above	≥USD1195	no	19-24 yo	1
###	2019-2021	35-45 yo	Not elligible	Malay	currently married	Secondary	<USD1195	no	25-29 yo	1
###	2019-2021	35-45 yo	Not elligible	Malay	currently married	Tertiary and above	<USD1195	no	25-29 yo	1
###	2019-2021	35-45 yo	Not elligible	Malay	currently married	Tertiary and above	≥USD1195	no	25-29 yo	1
###	2019-2021	35-45 yo	Not elligible	Malay	separated/divorced/w	Tertiary and above	≥USD1195	no	19-24 yo	2
###	2019-2021	35-45 yo	Not elligible	Malay	single	Tertiary and above	<USD1195	no	30 and above	1
###	2019-2021	35-45 yo	Not elligible	Malay	single	Tertiary and above	≥USD1195	no	19-24 yo	1
###	2019-2021	35-45 yo	Not elligible	Malay	currently married	Secondary	<USD1195	no	19-24 yo	1
###	2019-2021	18-24 yo	school-based	Malay	currently married	Secondary	<USD1195	yes	18 yo and below	1
###	2019-2021	35-45 yo	Not elligible	Malay	currently married	Tertiary and above	≥USD1195	no	19-24 yo	1
###	2019-2021	35-45 yo	Not elligible	Malay	currently married	Tertiary and above	≥USD1195	no	19-24 yo	1
###	2019-2021	35-45 yo	Not elligible	Malay	currently married	Tertiary and above	<USD1195	yes	25-29 yo	1
###	2019-2021	35-45 yo	Not elligible	Malay	currently married	Secondary	<USD1195	no	30 and above	1
###	2019-2021	35-45 yo	Not elligible	Malay	separated/divorced/w	Tertiary and above	<USD1195	no	19-24 yo	1
###	2019-2021	35-45 yo	Not elligible	Malay	currently married	Secondary	<USD1195	no	18 yo and below	1
###	2019-2021	35-45 yo	Not elligible	Malay	currently married	Tertiary and above	≥USD1195	no	19-24 yo	1
###	2019-2021	35-45 yo	Not elligible	Malay	currently married	Tertiary and above	≥USD1195	no	25-29 yo	1
###	2019-2021	35-45 yo	Not elligible	Malay	currently married	Tertiary and above	≥USD1195	no	19-24 yo	1
###	2019-2021	35-45 yo	Not elligible	Malay	currently married	Tertiary and above	≥USD1195	yes	25-29 yo	2
###	2019-2021	35-45 yo	Not elligible	Chinese	currently married	Tertiary and above	≥USD1195	no	19-24 yo	1
###	2019-2021	35-45 yo	Not elligible	Chinese	currently married	Tertiary and above	≥USD1195	no	19-24 yo	
###	2019-2021	35-45 yo	Not elligible	Malay	currently married	None-primary	<USD1195	no	18 yo and below	2
###	2019-2021	35-45 yo	Not elligible	Malay	currently married	Tertiary and above	<USD1195	no	30 and above	1
###	2019-2021	18-24 yo	school-based	Malay	currently married	Secondary	<USD1195	yes	19-24 yo	1
###	2019-2021	35-45 yo	Not elligible	Malay	currently married	Secondary	<USD1195	no	19-24 yo	1
###	2019-2021	35-45 yo	Not elligible	Malay	currently married	Tertiary and above	≥USD1195	no	25-29 yo	1
###	2019-2021	35-45 yo	Not elligible	Malay	currently married	Secondary	<USD1195	no	19-24 yo	1
###	2019-2021	35-45 yo	Not elligible	Malay	currently married	Secondary	<USD1195	no	19-24 yo	2
###	2019-2021	35-45 yo	Not elligible	Chinese	currently married	Tertiary and above	≥USD1195	no	19-24 yo	1
###	2019-2021	18-24 yo	catchup	Malay	single	Secondary	<USD1195	no	19-24 yo	3 and above

###	2019-2021	35-45 yo	Not elligible	Malay	currently married	Tertiary and above	≥USD1195	no	19-24 yo	1
###	2019-2021	35-45 yo	Not elligible	Malay	currently married	Secondary	<USD1195	no	30 and above	1
###	2019-2021	35-45 yo	Not elligible	Malay	currently married	Secondary	<USD1195	no	25-29 yo	2
###	2019-2021	18-24 yo	school-based	Malay	currently married	Tertiary and above	<USD1195	yes	19-24 yo	1
###	2019-2021	18-24 yo	school-based	Malay	currently married	Secondary	<USD1195	yes	18 yo and below	1
###	2019-2021	35-45 yo	Not elligible	Malay	currently married	Tertiary and above	≥USD1195	no	25-29 yo	1
###	2019-2021	35-45 yo	Not elligible	Malay	currently married	Tertiary and above	<USD1195	no	19-24 yo	1
###	2019-2021	35-45 yo	Not elligible	Malay	currently married	Secondary	<USD1195	no	25-29 yo	1
###	2019-2021	18-24 yo	catchup	Chinese	single	Tertiary and above	<USD1195	yes	18 yo and below	3 and above
###	2019-2021	35-45 yo	Not elligible	Malay	currently married	Tertiary and above	<USD1195	no	19-24 yo	1
###	2019-2021	18-24 yo	school-based	Malay	currently married	Secondary	<USD1195	yes	19-24 yo	1
###	2019-2021	35-45 yo	Not elligible	Malay	separated/divorced/w	Secondary	<USD1195	no	18 yo and below	3 and above
###	2019-2021	35-45 yo	Not elligible	Malay	currently married	Tertiary and above	<USD1195	no	19-24 yo	1
###	2019-2021	35-45 yo	Not elligible	Malay	currently married	Tertiary and above	<USD1195	no	30 and above	1
###	2019-2021	35-45 yo	Not elligible	Chinese	currently married	Tertiary and above	≥USD1195	no	30 and above	1
###	2019-2021	35-45 yo	Not elligible	Indian	currently married	Tertiary and above	≥USD1195	no	30 and above	1
###	2019-2021	35-45 yo	Not elligible	Indian	currently married	Tertiary and above	≥USD1195	no	25-29 yo	1
###	2019-2021	35-45 yo	Not elligible	Malay	currently married	Tertiary and above	≥USD1195	no	25-29 yo	1
###	2019-2021	35-45 yo	Not elligible	Malay	currently married	Tertiary and above	<USD1195	no	25-29 yo	1
###	2019-2021	35-45 yo	Not elligible	Malay	currently married	Tertiary and above	≥USD1195	no	25-29 yo	1
###	2019-2021	35-45 yo	Not elligible	Chinese	separated/divorced/w	Secondary	<USD1195	no	19-24 yo	1
###	2019-2021	35-45 yo	Not elligible	Malay	currently married	Tertiary and above	<USD1195	no	25-29 yo	1
###	2019-2021	35-45 yo	Not elligible	Other	currently married	Tertiary and above	<USD1195	no	25-29 yo	1
###	2019-2021	35-45 yo	Not elligible	Chinese	currently married	Tertiary and above	≥USD1195	no	25-29 yo	1
###	2019-2021	35-45 yo	Not elligible	Malay	currently married	Tertiary and above	≥USD1195	no	25-29 yo	1
###	2019-2021	18-24 yo	catchup	Chinese	single	Tertiary and above	≥USD1195	yes	18 yo and below	3 and above
###	2019-2021	35-45 yo	Not elligible	Malay	currently married	Secondary	<USD1195	no	19-24 yo	2
###	2019-2021	35-45 yo	Not elligible	Malay	separated/divorced/w	Tertiary and above	<USD1195	no	19-24 yo	1
###	2019-2021	35-45 yo	Not elligible	Malay	currently married	Tertiary and above	≥USD1195	no	25-29 yo	2
###	2019-2021	18-24 yo	school-based	Malay	currently married	None-primary	<USD1195	no	18 yo and below	3 and above
###	2019-2021	35-45 yo	Not elligible	Malay	currently married	Tertiary and above	<USD1195	no	30 and above	1
###	2019-2021	18-24 yo	school-based	Malay	currently married	Secondary	<USD1195	yes	19-24 yo	1
###	2019-2021	35-45 yo	Not elligible	Malay	currently married	Secondary	≥USD1195	no	30 and above	1
###	2019-2021	35-45 yo	Not elligible	Malay	currently married	Secondary	<USD1195	no	19-24 yo	1
###	2019-2021	18-24 yo	catchup	Malay	currently married	Secondary	<USD1195	yes	18 yo and below	1
###	2019-2021	35-45 yo	Not elligible	Malay	currently married	Secondary	<USD1195	no	25-29 yo	2
###	2019-2021	18-24 yo	school-based	Malay	currently married	Secondary	<USD1195	yes	19-24 yo	1
###	2019-2021	35-45 yo	Not elligible	Malay	currently married	Tertiary and above	<USD1195	no	19-24 yo	1
###	2019-2021	35-45 yo	Not elligible	Malay	currently married	Tertiary and above	≥USD1195	no	25-29 yo	1
###	2019-2021	35-45 yo	Not elligible	Chinese	currently married	Tertiary and above	≥USD1195	no	19-24 yo	1

###	2019-2021	35-45 yo	Not elligible	Malay	currently married	Secondary	<USD1195	no	19-24 yo	2
###	2019-2021	35-45 yo	Not elligible	Malay	currently married	Tertiary and above	≥USD1195	no	19-24 yo	1
###	2019-2021	35-45 yo	Not elligible	Malay	currently married	Secondary	≥USD1195	no	25-29 yo	1
###	2019-2021	35-45 yo	Not elligible	Malay	currently married	Secondary	≥USD1195	no	19-24 yo	1
###	2019-2021	35-45 yo	Not elligible	Malay	currently married	Tertiary and above	≥USD1195	no	19-24 yo	1
###	2019-2021	35-45 yo	Not elligible	Malay	currently married	Tertiary and above	≥USD1195	no	19-24 yo	1
###	2019-2021	35-45 yo	Not elligible	Malay	currently married	Secondary	<USD1195	no	25-29 yo	1
###	2019-2021	35-45 yo	Not elligible	Malay	currently married	Tertiary and above	≥USD1195	no	19-24 yo	2
###	2019-2021	18-24 yo	school-based	Malay	currently married	Secondary	<USD1195	yes	19-24 yo	1
###	2019-2021	18-24 yo	school-based	Malay	currently married	Tertiary and above	<USD1195	yes	19-24 yo	1
###	2019-2021	35-45 yo	Not elligible	Malay	currently married	Tertiary and above	<USD1195	no	25-29 yo	1
###	2019-2021	18-24 yo	school-based	Malay	separated/divorced/w	Secondary	<USD1195	yes	18 yo and below	1
###	2019-2021	35-45 yo	Not elligible	Malay	currently married	Tertiary and above	<USD1195	no	19-24 yo	2
###	2019-2021	35-45 yo	Not elligible	Malay	currently married	Secondary	<USD1195	no	19-24 yo	3 and above
###	2019-2021	18-24 yo	catchup	Malay	currently married	Tertiary and above	<USD1195	yes	19-24 yo	1
###	2019-2021	35-45 yo	Not elligible	Malay	currently married	Tertiary and above	≥USD1195	no	25-29 yo	1
###	2019-2021	18-24 yo	school-based	Malay	currently married	Secondary	<USD1195	yes	18 yo and below	2
###	2019-2021	18-24 yo	catchup	Chinese	currently married	None-primary	<USD1195	no		3 and above
###	2019-2021	18-24 yo	school-based	Malay	currently married	Secondary	<USD1195	yes	19-24 yo	1
###	2019-2021	18-24 yo	school-based	Chinese	separated/divorced/w	Secondary	<USD1195	yes	18 yo and below	2
###	2019-2021	35-45 yo	Not elligible	Other	currently married	None-primary	<USD1195	no	18 yo and below	1
###	2019-2021	18-24 yo	school-based	Malay	currently married	Tertiary and above	<USD1195	yes	18 yo and below	1
###	2019-2021	18-24 yo	school-based	Malay	separated/divorced/w	Secondary	<USD1195	yes	18 yo and below	3 and above
###	2019-2021	18-24 yo	catchup	Malay	currently married	Secondary	<USD1195	no	19-24 yo	1
###	2019-2021	35-45 yo	Not elligible	Malay	currently married	Tertiary and above	≥USD1195	no	19-24 yo	1
###	2019-2021	18-24 yo	school-based	Malay	currently married	Secondary	<USD1195	yes	19-24 yo	1
###	2019-2021	18-24 yo	catchup	Other	currently married	Tertiary and above	≥USD1195	yes	19-24 yo	2
###	2019-2021	35-45 yo	Not elligible	Malay	currently married	Tertiary and above	<USD1195	no	25-29 yo	1
###	2019-2021	35-45 yo	Not elligible	Malay	currently married	Tertiary and above	<USD1195	no	25-29 yo	1
###	2019-2021	18-24 yo	school-based	Malay	currently married	None-primary	<USD1195	no	18 yo and below	1
###	2019-2021	18-24 yo	catchup	Malay	single	Secondary	<USD1195	yes	18 yo and below	3 and above
###	2019-2021	18-24 yo	catchup	Chinese	single	Tertiary and above	≥USD1195	yes	19-24 yo	3 and above
###	2019-2021	18-24 yo	catchup	Malay	single	Tertiary and above	≥USD1195	no	19-24 yo	1
###	2019-2021	35-45 yo	Not elligible	Chinese	currently married	Secondary	<USD1195	no	25-29 yo	1
###	2019-2021	35-45 yo	Not elligible	Malay	currently married	Tertiary and above	≥USD1195	no	18 yo and below	3 and above
###	2019-2021	18-24 yo	school-based	Malay	single	Secondary	<USD1195	yes	19-24 yo	1
###	2019-2021	18-24 yo	school-based	Chinese	currently married	None-primary	<USD1195	yes	19-24 yo	1
###	2019-2021	18-24 yo	school-based	Malay	currently married	Secondary	<USD1195	yes	18 yo and below	2
###	2019-2021	18-24 yo	catchup	Other	currently married	None-primary	<USD1195	no	18 yo and below	1
###	2019-2021	18-24 yo	catchup	Malay	currently married	Tertiary and above	<USD1195	no	19-24 yo	1

###	2019-2021	35-45 yo	Not elligible	Chinese	currently married	Tertiary and above	≥USD1195	yes	25-29 yo	1
###	2019-2021	18-24 yo	catchup	Malay	currently married	Secondary	<USD1195	no	19-24 yo	2
###	2019-2021	35-45 yo	Not elligible	Chinese	currently married	Tertiary and above	≥USD1195	no	25-29 yo	2
###	2019-2021	18-24 yo	school-based	Other	currently married	None-primary	<USD1195	no	19-24 yo	2
###	2019-2021	18-24 yo	school-based	Malay	currently married	Secondary	<USD1195	yes	18 yo and below	1
###	2019-2021	18-24 yo	school-based	Chinese	single	Tertiary and above	≥USD1195	yes	18 yo and below	1
###	2019-2021	18-24 yo	school-based	Malay	currently married	Tertiary and above	<USD1195	no	19-24 yo	1
###	2019-2021	35-45 yo	Not elligible	Indian	currently married	None-primary	<USD1195	no	18 yo and below	1
###	2019-2021	18-24 yo	catchup	Chinese	single	Tertiary and above	≥USD1195	no	19-24 yo	2
###	2019-2021	18-24 yo	catchup	Malay	currently married	Secondary	<USD1195	yes	19-24 yo	1
###	2019-2021	18-24 yo	catchup	Malay	currently married	Secondary	<USD1195	yes	19-24 yo	1
###	2019-2021	18-24 yo	school-based	Malay	currently married	Secondary	<USD1195	yes	18 yo and below	1
###	2019-2021	18-24 yo	school-based	Other	currently married	Secondary	<USD1195	yes	19-24 yo	1
###	2019-2021	18-24 yo	catchup	Malay	currently married	Tertiary and above	<USD1195	yes	19-24 yo	1
###	2019-2021	18-24 yo	catchup	Malay	currently married	Secondary	<USD1195	no	19-24 yo	1
###	2019-2021	18-24 yo	catchup	Chinese	currently married	Secondary	<USD1195	no	19-24 yo	1
###	2019-2021	18-24 yo	school-based	Malay	currently married	Secondary	<USD1195	yes	18 yo and below	1
###	2019-2021	18-24 yo	school-based	Malay	currently married	Tertiary and above	<USD1195	yes	19-24 yo	1
###	2019-2021	18-24 yo	school-based	Chinese	single	Secondary	<USD1195	yes	18 yo and below	1
###	2019-2021	18-24 yo	catchup	Chinese	currently married	Secondary	<USD1195	yes	19-24 yo	2
###	2019-2021	18-24 yo	school-based	Malay	currently married	Secondary	<USD1195	yes	19-24 yo	1
###	2019-2021	18-24 yo	school-based	Malay	currently married	Tertiary and above	<USD1195	yes	19-24 yo	1
###	2019-2021	18-24 yo	catchup	Chinese	currently married	Secondary	<USD1195	yes	18 yo and below	3 and above
###	2019-2021	18-24 yo	school-based	Malay	currently married	Secondary	<USD1195	yes	18 yo and below	1
###	2019-2021	18-24 yo	school-based	Malay	currently married	Secondary	≥USD1195	yes	18 yo and below	3 and above
###	2019-2021	18-24 yo	catchup	Chinese	single	Tertiary and above	<USD1195	yes	19-24 yo	1
###	2019-2021	18-24 yo	school-based	Other	single	Secondary	<USD1195	yes	19-24 yo	1
###	2019-2021	18-24 yo	school-based	Malay	currently married	Secondary	<USD1195	yes	19-24 yo	1
###	2019-2021	18-24 yo	school-based	Malay	single	Secondary	<USD1195	yes	18 yo and below	3 and above
###	2019-2021	18-24 yo	school-based	Malay	currently married	Secondary	<USD1195	yes	18 yo and below	3 and above
###	2019-2021	18-24 yo	school-based	Malay	single	Secondary	<USD1195	yes	18 yo and below	3 and above
###	2019-2021	18-24 yo	catchup	Malay	currently married	Secondary	<USD1195	yes	19-24 yo	2
###	2019-2021	18-24 yo	school-based	Malay	single	Tertiary and above	<USD1195	yes	18 yo and below	1
###	2019-2021	18-24 yo	school-based	Other	currently married	Secondary	<USD1195	yes	19-24 yo	1
###	2019-2021	18-24 yo	catchup	Malay	currently married	Tertiary and above	<USD1195	yes	19-24 yo	1
###	2019-2021	18-24 yo	catchup	Chinese	single	Tertiary and above	≥USD1195	yes	19-24 yo	1
###	2019-2021	18-24 yo	school-based	Other	single	Secondary	<USD1195	yes	19-24 yo	2
###	2019-2021	18-24 yo	school-based	Malay	single	Tertiary and above	<USD1195	yes	18 yo and below	1
###	2019-2021	18-24 yo	school-based	Indian	currently married	Secondary	<USD1195	yes	18 yo and below	2
###	2019-2021	18-24 yo	school-based	Malay	currently married	None-primary	<USD1195	no	18 yo and below	1

###	2019-2021	18-24 yo	school-based	Other	single	None-primary	<USD1195	no	18 yo and below	1
###	2019-2021	18-24 yo	school-based	Other	currently married	Secondary	<USD1195	yes	18 yo and below	3 and above
###	2019-2021	18-24 yo	school-based	Other	currently married	Secondary	<USD1195	yes	19-24 yo	1
###	2019-2021	18-24 yo	catchup	Malay	currently married	Tertiary and above	<USD1195	no	19-24 yo	1
###	2019-2021	18-24 yo	school-based	Malay	single	Secondary	<USD1195	yes	18 yo and below	2
###	2019-2021	18-24 yo	school-based	Malay	currently married	Secondary	<USD1195	no	18 yo and below	2
###	2019-2021	18-24 yo	school-based	Malay	single	Secondary	<USD1195	yes	18 yo and below	1
###	2019-2021	18-24 yo	school-based	Indian	currently married	Secondary	<USD1195	yes	18 yo and below	1
###	2019-2021	18-24 yo	school-based	Indian	currently married	Secondary	<USD1195	yes	18 yo and below	2
###	2019-2021	18-24 yo	school-based	Indian	currently married	Secondary	<USD1195	yes	19-24 yo	1
###	2019-2021	18-24 yo	school-based	Malay	currently married	None-primary	<USD1195	no	18 yo and below	1
###	2019-2021	18-24 yo	catchup	Malay	currently married	Tertiary and above	<USD1195	no	19-24 yo	1
###	2019-2021	18-24 yo	school-based	Malay	currently married	None-primary	<USD1195	no	18 yo and below	1
###	2019-2021	18-24 yo	school-based	Malay	currently married	Secondary	<USD1195	yes	18 yo and below	1
###	2019-2021	18-24 yo	catchup	Malay	currently married	Tertiary and above	≥USD1195	yes	19-24 yo	1
###	2019-2021	18-24 yo	catchup	Malay	currently married	Secondary	<USD1195	no	19-24 yo	1
###	2019-2021	18-24 yo	catchup	Malay	currently married	Secondary	<USD1195	no	19-24 yo	3 and above
###	2019-2021	18-24 yo	school-based	Malay	currently married	Secondary	<USD1195	yes	19-24 yo	1
###	2019-2021	18-24 yo	catchup	Malay	currently married	Secondary	<USD1195	no	19-24 yo	1
###	2019-2021	18-24 yo	catchup	Malay	separated/divorced/w	None-primary	<USD1195	no	18 yo and below	1
###	2019-2021	18-24 yo	school-based	Malay	currently married	Secondary	<USD1195	yes	19-24 yo	1
###	2019-2021	18-24 yo	school-based	Other	currently married	Secondary	<USD1195	yes	18 yo and below	1
###	2019-2021	18-24 yo	school-based	Other	currently married	Tertiary and above	<USD1195	yes	18 yo and below	3 and above
###	2019-2021	18-24 yo	school-based	Malay	currently married	Tertiary and above	<USD1195	yes	19-24 yo	1
###	2019-2021	18-24 yo	school-based	Malay	currently married	Secondary	<USD1195	yes	19-24 yo	2
###	2019-2021	18-24 yo	school-based	Other	currently married	Secondary	<USD1195	yes	19-24 yo	1
###	2019-2021	18-24 yo	school-based	Malay	currently married	Secondary	<USD1195	yes	19-24 yo	1
###	2019-2021	18-24 yo	school-based	Malay	currently married	Secondary	<USD1195	yes	19-24 yo	1
###	2019-2021	18-24 yo	school-based	Malay	currently married	Secondary	<USD1195	yes	19-24 yo	1
###	2019-2021	18-24 yo	catchup	Malay	currently married	Tertiary and above	<USD1195	no	19-24 yo	1
###	2019-2021	18-24 yo	catchup	Malay	currently married	Secondary	<USD1195	yes	19-24 yo	1
###	2019-2021	18-24 yo	school-based	Malay	separated/divorced/w	Secondary	<USD1195	yes	18 yo and below	3 and above
###	2019-2021	18-24 yo	school-based	Malay	currently married	Secondary	<USD1195	yes	19-24 yo	1
###	2019-2021	35-45 yo	Not elligible	Indian	currently married	Tertiary and above	≥USD1195	no	19-24 yo	1
###	2019-2021	18-24 yo	catchup	Malay	currently married	Tertiary and above	<USD1195	no	19-24 yo	1
###	2019-2021	18-24 yo	school-based	Malay	currently married	Tertiary and above	<USD1195	yes	19-24 yo	2
###	2019-2021	18-24 yo	school-based	Other	currently married	Secondary	<USD1195	yes	19-24 yo	1
###	2019-2021	18-24 yo	school-based	Malay	currently married	Secondary	<USD1195	no	19-24 yo	1
###	2019-2021	18-24 yo	catchup	Malay	currently married	Secondary	<USD1195	no	18 yo and below	1
###	2019-2021	18-24 yo	school-based	Malay	currently married	Secondary	<USD1195	yes	19-24 yo	2

###	2019-2021	18-24 yo	school-based	Malay	currently married	Secondary	<USD1195	yes	19-24 yo	1
###	2019-2021	18-24 yo	catchup	Malay	currently married	Secondary	<USD1195	yes	19-24 yo	1
###	2019-2021	18-24 yo	catchup	Malay	currently married	Secondary	<USD1195	yes	19-24 yo	1
###	2019-2021	18-24 yo	school-based	Malay	currently married	Secondary	<USD1195	yes	18 yo and below	1
###	2019-2021	18-24 yo	school-based	Other	currently married	None-primary	<USD1195	no	18 yo and below	1
###	2019-2021	18-24 yo	catchup	Malay	currently married	Tertiary and above	<USD1195	yes	19-24 yo	1
###	2019-2021	18-24 yo	school-based	Malay	currently married	Secondary	<USD1195	yes	19-24 yo	1
###	2019-2021	18-24 yo	school-based	Malay	currently married	Tertiary and above	<USD1195	yes	19-24 yo	1
###	2019-2021	18-24 yo	catchup	Malay	currently married	Tertiary and above	<USD1195	no	19-24 yo	1
###	2019-2021	18-24 yo	school-based	Malay	currently married	Secondary	<USD1195	yes	19-24 yo	1
###	2019-2021	18-24 yo	school-based	Malay	currently married	Tertiary and above	<USD1195	yes	19-24 yo	1
###	2019-2021	18-24 yo	school-based	Malay	currently married	Secondary	<USD1195	yes	19-24 yo	1
###	2019-2021	18-24 yo	school-based	Malay	currently married	Secondary	<USD1195	yes	18 yo and below	1
###	2019-2021	18-24 yo	school-based	Malay	currently married	Secondary	<USD1195	yes	18 yo and below	1
###	2019-2021	18-24 yo	catchup	Malay	currently married	Secondary	<USD1195	yes	19-24 yo	1
###	2019-2021	18-24 yo	school-based	Malay	separated/divorced/w	Secondary	<USD1195	yes	19-24 yo	1
###	2019-2021	18-24 yo	catchup	Malay	currently married	Secondary	<USD1195	yes	19-24 yo	2
###	2019-2021	18-24 yo	catchup	Malay	currently married	Tertiary and above	<USD1195	yes	19-24 yo	1
###	2019-2021	18-24 yo	school-based	Malay	currently married	Secondary	<USD1195	yes	18 yo and below	1
###	2019-2021	18-24 yo	school-based	Other	currently married	Secondary	<USD1195	yes	18 yo and below	3 and above
###	2019-2021	18-24 yo	school-based	Malay	currently married	Secondary	<USD1195	yes	18 yo and below	2
###	2019-2021	18-24 yo	school-based	Malay	currently married	Secondary	<USD1195	yes	19-24 yo	1
###	2019-2021	18-24 yo	school-based	Other	currently married	Secondary	<USD1195	yes	18 yo and below	3 and above
###	2019-2021	18-24 yo	school-based	Malay	currently married	Tertiary and above	<USD1195	yes	19-24 yo	1
###	2019-2021	18-24 yo	catchup	Chinese	currently married	Secondary	<USD1195	no	18 yo and below	3 and above
###	2019-2021	18-24 yo	school-based	Other	single	None-primary	<USD1195	no	18 yo and below	3 and above
###	2019-2021	18-24 yo	catchup	Malay	currently married	Secondary	<USD1195	no	19-24 yo	3 and above
###	2019-2021	18-24 yo	school-based	Malay	currently married	Secondary	≥USD1195	yes	19-24 yo	1
###	2019-2021	18-24 yo	school-based	Chinese	currently married	Tertiary and above	<USD1195	yes	18 yo and below	2
###	2019-2021	35-45 yo	Not elligible	Indian	currently married	Secondary	<USD1195	no	18 yo and below	1
###	2019-2021	18-24 yo	catchup	Malay	currently married	Secondary	<USD1195	no	19-24 yo	1
###	2019-2021	18-24 yo	school-based	Malay	currently married	Secondary	<USD1195	yes	19-24 yo	1
###	2019-2021	18-24 yo	school-based	Malay	currently married	Secondary	<USD1195	yes	18 yo and below	1
###	2019-2021	18-24 yo	catchup	Indian	currently married	Secondary	<USD1195	no	18 yo and below	1
###	2019-2021	18-24 yo	school-based	Malay	single	Secondary	<USD1195	yes	18 yo and below	3 and above
###	2019-2021	18-24 yo	catchup	Malay	currently married	Secondary	<USD1195	no	19-24 yo	1
###	2019-2021	18-24 yo	catchup	Malay	currently married	Tertiary and above	<USD1195	yes	19-24 yo	1
###	2019-2021	18-24 yo	catchup	Malay	currently married	Secondary	<USD1195	yes	19-24 yo	1
###	2019-2021	18-24 yo	school-based	Malay	currently married	Secondary	<USD1195	yes	18 yo and below	2
###	2019-2021	18-24 yo	school-based	Chinese	currently married	Secondary	≥USD1195	yes	18 yo and below	3 and above

###	2019-2021	18-24 yo	catchup	Chinese	single	Tertiary and above	≥USD1195	no	18 yo and below	1
###	2019-2021	18-24 yo	catchup	Malay	currently married	Tertiary and above	≥USD1195	yes	19-24 yo	1
###	2019-2021	18-24 yo	catchup	Malay	single	Tertiary and above	≥USD1195	no	19-24 yo	3 and above

newpart_12	circum	partner_behav	Anyhpv_16types	HRHPV	C2	G4	G9	5_G9	HPV6	HPV11	HPV16	HPV18
no	yes	Stay at home, no other partners	0	no	0	0	0	0	0	0	0	0
no	yes	Stay at home, no other partners	0	no	0	0	0	0	0	0	0	0
no	yes	Stay at home, no other partners	0	no	0	0	0	0	0	0	0	0
no	yes	Stay at home, no other partners	0	no	0	0	0	0	0	0	0	0
no	yes	Stay at home, no other partners	0	no	0	0	0	0	0	0	0	0
no	yes	Stay at home, no other partners	0	no	0	0	0	0	0	0	0	0
no	yes	Stay at home, no other partners	0	no	0	0	0	0	0	0	0	0
no	yes	Stay at home, no other partners	0	no	0	0	0	0	0	0	0	0
no	yes	Stay at home, no other partners	0	no	0	0	0	0	0	0	0	0
no	no	Stay at home, no other partners	0	no	0	0	0	0	0	0	0	0
no	yes	Stay at home, no other partners	0	no	0	0	0	0	0	0	0	0
no	no	Stay at home, no other partners	0	no	0	0	0	0	0	0	0	0
no	yes	Stay at home, no other partners	0	no	0	0	0	0	0	0	0	0
no	no	Stay at home, no other partners	0	no	0	0	0	0	0	0	0	0
no	yes	Stay at home, no other partners	0	no	0	0	0	0	0	0	0	0
no	yes	Stay at home, no other partners	0	no	0	0	0	0	0	0	0	0
no	yes	Stay at home, no other partners	0	no	0	0	0	0	0	0	0	0
no	no	Stay at home, no other partners	0	no	0	0	0	0	0	0	0	0
no	yes	Stay at home, no other partners	0	no	0	0	0	0	0	0	0	0
no	yes	Stay at home, no other partners	0	no	0	0	0	0	0	0	0	0
no	yes	Stay at home, no other partners	0	no	0	0	0	0	0	0	0	0
no		Stay at home, no other partners	0	no	0	0	0	0	0	0	0	0
no	no	Stay at home, no other partners	0	no	0	0	0	0	0	0	0	0
no	yes	Stay at home, no other partners	0	no	0	0	0	0	0	0	0	0
yes	yes	Stay at home, no other partners	0	no	0	0	0	0	0	0	0	0
no	yes	Stay at home, no other partners	0	no	0	0	0	0	0	0	0	0
no	yes	Stay at home, no other partners	0	no	0	0	0	0	0	0	0	0
no	no	Stay at home, no other partners	0	no	0	0	0	0	0	0	0	0
no	yes	Stay at home, no other partners	0	no	0	0	0	0	0	0	0	0
no	yes	Stay at home, no other partners	0	no	0	0	0	0	0	0	0	0
yes	yes	Stay at home, no other partners	1	yes	0	0	0	0	0	0	0	0
no	yes	Stay at home, no other partners	0	no	0	0	0	0	0	0	0	0
no	yes	Stay at home, no other partners	0	no	0	0	0	0	0	0	0	0
no	yes	Stay at home, no other partners	0	no	0	0	0	0	0	0	0	0
no	yes	Stay at home, no other partners	0	no	0	0	0	0	0	0	0	0
no	no	Stay at home, no other partners	0	no	0	0	0	0	0	0	0	0
no	yes	Stay at home, no other partners	0	no	0	0	0	0	0	0	0	0
no	no	Stay at home, no other partners	0	no	0	0	0	0	0	0	0	0
no	yes	Stay at home, no other partners	0	no	0	0	0	0	0	0	0	0

no	yes	Stay at home, no other partners	0	no	0	0	0	0	0	0	0	0
no	yes	Stay at home, no other partners	0	no	0	0	0	0	0	0	0	0
no	yes	Stay at home, no other partners	0	no	0	0	0	0	0	0	0	0
no	no	Stay at home, no other partners	0	no	0	0	0	0	0	0	0	0
no	no	Stay at home, no other partners	0	no	0	0	0	0	0	0	0	0
no	no	Stay at home, no other partners	0	no	0	0	0	0	0	0	0	0
no	no	Stay at home, no other partners	0	no	0	0	0	0	0	0	0	0
no	no	Stay at home, no other partners	0	no	0	0	0	0	0	0	0	0
no	no	Stay at home, no other partners	0	no	0	0	0	0	0	0	0	0
no	yes	Stay at home, no other partners	0	no	0	0	0	0	0	0	0	0
no	no	Stay at home, no other partners	0	no	0	0	0	0	0	0	0	0
no	no	Stay at home, no other partners	0	no	0	0	0	0	0	0	0	0
no	no	Stay at home, no other partners	0	no	0	0	0	0	0	0	0	0
no	no	Stay at home, no other partners	0	no	0	0	0	0	0	0	0	0
no	yes	Stay at home, no other partners	0	no	0	0	0	0	0	0	0	0
no	yes	Stay at home, no other partners	0	no	0	0	0	0	0	0	0	0
no	yes	Stay at home, no other partners	0	no	0	0	0	0	0	0	0	0
no		Stay at home, no other partners	0	no	0	0	0	0	0	0	0	0
no	no	Stay at home, no other partners	0	no	0	0	0	0	0	0	0	0
no	yes	Stay at home, no other partners	0	no	0	0	0	0	0	0	0	0
no	yes	Stay at home, no other partners	0	no	0	0	0	0	0	0	0	0
no	no	Stay at home, no other partners	0	no	0	0	0	0	0	0	0	0
no	yes	Stay at home, no other partners	0	no	0	0	0	0	0	0	0	0
no	yes	Stay at home, no other partners	0	no	0	0	0	0	0	0	0	0
no	no	Stay at home, no other partners	0	no	0	0	0	0	0	0	0	0
no		Stay at home, no other partners	0	no	0	0	0	0	0	0	0	0
no	yes	Stay at home, no other partners	0	no	0	0	0	0	0	0	0	0
no	yes	Stay at home, no other partners	0	no	0	0	0	0	0	0	0	0
no	no	Stay at home, no other partners	0	no	0	0	0	0	0	0	0	0
no	no	Stay at home, no other partners	0	no	0	0	0	0	0	0	0	0
no	yes	Stay at home, no other partners	0	no	0	0	0	0	0	0	0	0
no	no	Stay at home, no other partners	0	no	0	0	0	0	0	0	0	0
no	yes	Stay at home, no other partners	0	no	0	0	0	0	0	0	0	0
no	no	Stay at home, no other partners	0	no	0	0	0	0	0	0	0	0
no	yes	Stay at home, no other partners	0	no	0	0	0	0	0	0	0	0
no	yes	Stay at home, no other partners	0	no	0	0	0	0	0	0	0	0
no	no	Stay at home, no other partners	0	no	0	0	0	0	0	0	0	0
no	yes	Stay at home, has other partners	1	no	0	1	1	0	0	1	0	0
no	no	Stay at home, no other partners	0	no	0	0	0	0	0	0	0	0
no	no	Stay at home, no other partners	0	no	0	0	0	0	0	0	0	0

no	yes	Stay at home, no other partners	0	no	0	0	0	0	0	0	0	0
no	yes	Stay at home, no other partners	1	yes	1	1	1	0	0	0	1	0
no	yes	Stay at home, no other partners	0	no	0	0	0	0	0	0	0	0
no	yes	Stay at home, no other partners	0	no	0	0	0	0	0	0	0	0
no	yes	Stay at home, no other partners	0	no	0	0	0	0	0	0	0	0
no	yes	Stay at home, no other partners	1	yes	0	0	0	0	0	0	0	0
no	yes	Stay at home, no other partners	0	no	0	0	0	0	0	0	0	0
no	yes	Stay at home, no other partners	0	no	0	0	0	0	0	0	0	0
no	no	Stay at home, no other partners	0	no	0	0	0	0	0	0	0	0
no	yes	Stay at home, no other partners	0	no	0	0	0	0	0	0	0	0
no	yes	Stay at home, no other partners	0	no	0	0	0	0	0	0	0	0
no	no	Stay at home, no other partners	0	no	0	0	0	0	0	0	0	0
no	yes	Stay at home, no other partners	0	no	0	0	0	0	0	0	0	0
no	yes	Stay at home, no other partners	0	no	0	0	0	0	0	0	0	0
no		Stay at home, no other partners	0	no	0	0	0	0	0	0	0	0
no	yes	Stay at home, no other partners	0	no	0	0	0	0	0	0	0	0
no	yes	Stay at home, no other partners	0	no	0	0	0	0	0	0	0	0
no	no	Stay at home, no other partners	0	no	0	0	0	0	0	0	0	0
no	yes	Stay at home, no other partners	0	no	0	0	0	0	0	0	0	0
no	yes	Stay at home, has other partners	1	yes	1	1	1	0	0	0	1	0
no	yes	Stay at home, no other partners	0	no	0	0	0	0	0	0	0	0
no	yes	Stay at home, no other partners	0	no	0	0	0	0	0	0	0	0
no	yes	Stay at home, no other partners	0	no	0	0	0	0	0	0	0	0
no	yes	Stay at home, no other partners	0	no	0	0	0	0	0	0	0	0
no	yes	Stay at home, no other partners	0	no	0	0	0	0	0	0	0	0
no		Stay at home, no other partners	0	no	0	0	0	0	0	0	0	0
no	yes	Stay at home, no other partners	0	no	0	0	0	0	0	0	0	0
no	yes	Stay at home, no other partners	0	no	0	0	0	0	0	0	0	0
no	yes	Stay at home, no other partners	0	no	0	0	0	0	0	0	0	0
no	yes	Stay at home, no other partners	0	no	0	0	0	0	0	0	0	0
no	yes	Stay at home, no other partners	0	no	0	0	0	0	0	0	0	0
no	no	Stay at home, no other partners	0	no	0	0	0	0	0	0	0	0
no	yes	Stay at home, no other partners	0	no	0	0	0	0	0	0	0	0
no	no	Stay at home, no other partners	0	no	0	0	0	0	0	0	0	0
no	no	Stay at home, no other partners	0	no	0	0	0	0	0	0	0	0
no	yes	Stay at home, no other partners	0	no	0	0	0	0	0	0	0	0
no	yes	Stay at home, no other partners	0	no	0	0	0	0	0	0	0	0
no	yes	Stay away, no other partners	1	yes	1	1	1	0	0	0	1	0
no	yes	Stay at home, no other partners	0	no	0	0	0	0	0	0	0	0
no	no	Stay at home, no other partners	0	no	0	0	0	0	0	0	0	0

no	no	Stay at home, no other partners	0	no	0	0	0	0	0	0	0	0
no	yes	Stay at home, no other partners	0	no	0	0	0	0	0	0	0	0
no	no	Stay at home, no other partners	0	no	0	0	0	0	0	0	0	0
no	yes	Stay at home, no other partners	0	no	0	0	0	0	0	0	0	0
no	no	Stay at home, no other partners	0	no	0	0	0	0	0	0	0	0
no	yes	Stay at home, no other partners	0	no	0	0	0	0	0	0	0	0
no	yes	Stay at home, no other partners	0	no	0	0	0	0	0	0	0	0
no	yes	Stay at home, no other partners	0	no	0	0	0	0	0	0	0	0
no	yes	Stay at home, no other partners	0	no	0	0	0	0	0	0	0	0
no	no	Stay at home, no other partners	0	no	0	0	0	0	0	0	0	0
no	yes	Stay at home, no other partners	0	no	0	0	0	0	0	0	0	0
no	no	Stay at home, no other partners	0	no	0	0	0	0	0	0	0	0
no	yes	Stay at home, no other partners	0	no	0	0	0	0	0	0	0	0
no	yes	Stay at home, no other partners	0	no	0	0	0	0	0	0	0	0
no	no	Stay away, has other partners	1	yes	1	1	1	0	0	0	1	0
no	no	Stay at home, no other partners	0	no	0	0	0	0	0	0	0	0
no	yes	Stay at home, no other partners	0	no	0	0	0	0	0	0	0	0
no	yes	Stay at home, no other partners	0	no	0	0	0	0	0	0	0	0
no	yes	Stay at home, no other partners	0	no	0	0	0	0	0	0	0	0
no	yes	Stay at home, no other partners	0	no	0	0	0	0	0	0	0	0
no	yes	Stay at home, no other partners	0	no	0	0	0	0	0	0	0	0
no		Stay at home, no other partners	0	no	0	0	0	0	0	0	0	0
no	no	Stay at home, no other partners	0	no	0	0	0	0	0	0	0	0
no	no	Stay at home, no other partners	0	no	0	0	0	0	0	0	0	0
no	no	Stay at home, no other partners	0	no	0	0	0	0	0	0	0	0
no	no	Stay at home, no other partners	0	no	0	0	0	0	0	0	0	0
no		Stay at home, no other partners	0	no	0	0	0	0	0	0	0	0
no	no	Stay at home, no other partners	0	no	0	0	0	0	0	0	0	0
no	yes	Stay at home, no other partners	0	no	0	0	0	0	0	0	0	0
no	no	Stay at home, no other partners	0	no	0	0	0	0	0	0	0	0
no	yes	Stay at home, no other partners	0	no	0	0	0	0	0	0	0	0
no	no	Stay at home, no other partners	0	no	0	0	0	0	0	0	0	0
no	no	Stay at home, no other partners	0	no	0	0	0	0	0	0	0	0
no	yes	Stay at home, no other partners	0	no	0	0	0	0	0	0	0	0
no	no	Stay at home, no other partners	0	no	0	0	0	0	0	0	0	0
no	yes	Stay at home, no other partners	0	no	0	0	0	0	0	0	0	0
no	no	Stay at home, no other partners	0	no	0	0	0	0	0	0	0	0
no	no	Stay at home, no other partners	0	no	0	0	0	0	0	0	0	0
no	yes	Stay at home, no other partners	0	no	0	0	0	0	0	0	0	0
no	yes	Stay at home, no other partners	0	no	0	0	0	0	0	0	0	0

no	yes	Stay at home, no other partners	0	no	0	0	0	0	0	0	0	0
no		no current partner	1	yes	1	1	1	0	0	0	1	0
no	yes	Stay at home, no other partners	0	no	0	0	0	0	0	0	0	0
no	yes	Stay at home, no other partners	0	no	0	0	0	0	0	0	0	0
no	yes	Stay at home, no other partners	0	no	0	0	0	0	0	0	0	0
no	yes	Stay at home, no other partners	0	no	0	0	0	0	0	0	0	0
no	yes	Stay at home, no other partners	0	no	0	0	0	0	0	0	0	0
no	no	Stay at home, no other partners	0	no	0	0	0	0	0	0	0	0
no	yes	Stay at home, no other partners	0	no	0	0	0	0	0	0	0	0
no	no	Stay at home, no other partners	1	yes	0	0	0	0	0	0	0	0
no	yes	Stay at home, no other partners	0	no	0	0	0	0	0	0	0	0
no	no	Stay at home, no other partners	0	no	0	0	0	0	0	0	0	0
no	yes	Stay at home, no other partners	0	no	0	0	0	0	0	0	0	0
no	yes	Stay at home, no other partners	0	no	0	0	0	0	0	0	0	0
no	no	Stay at home, no other partners	0	no	0	0	0	0	0	0	0	0
no	no	Stay at home, no other partners	0	no	0	0	0	0	0	0	0	0
no	yes	Stay at home, no other partners	0	no	0	0	0	0	0	0	0	0
no	no	Stay at home, no other partners	0	no	0	0	0	0	0	0	0	0
no	yes	Stay at home, no other partners	0	no	0	0	0	0	0	0	0	0
no	yes	Stay at home, no other partners	0	no	0	0	0	0	0	0	0	0
no	yes	Stay at home, no other partners	0	no	0	0	0	0	0	0	0	0
no	yes	Stay at home, no other partners	0	no	0	0	0	0	0	0	0	0
no	no	Stay at home, no other partners	0	no	0	0	0	0	0	0	0	0
no	no	Stay at home, no other partners	1	yes	0	0	0	0	0	0	0	0
no	no	Stay at home, no other partners	0	no	0	0	0	0	0	0	0	0
no	yes	Stay at home, no other partners	0	no	0	0	0	0	0	0	0	0
no	yes	Stay at home, no other partners	0	no	0	0	0	0	0	0	0	0
no	no	Stay at home, no other partners	0	no	0	0	0	0	0	0	0	0
no	no	Stay at home, no other partners	0	no	0	0	0	0	0	0	0	0
no	no	Stay at home, no other partners	0	no	0	0	0	0	0	0	0	0
no	no	Stay at home, no other partners	0	no	0	0	0	0	0	0	0	0
no	no	Stay at home, no other partners	0	no	0	0	0	0	0	0	0	0
no	yes	Stay at home, no other partners	0	no	0	0	0	0	0	0	0	0
no	yes	Stay at home, no other partners	0	no	0	0	0	0	0	0	0	0
no	yes	Stay at home, no other partners	0	no	0	0	0	0	0	0	0	0
no	yes	Stay at home, no other partners	0	no	0	0	0	0	0	0	0	0
no	no	Stay at home, no other partners	0	no	0	0	0	0	0	0	0	0
no	yes	Stay at home, no other partners	0	no	0	0	0	0	0	0	0	0
no	yes	Stay at home, no other partners	0	no	0	0	0	0	0	0	0	0
no	no	Stay at home, no other partners	0	no	0	0	0	0	0	0	0	0

no	no	Stay at home, no other partners	0	no	0	0	0	0	0	0	0	0
no	no	Stay at home, no other partners	0	no	0	0	0	0	0	0	0	0
no	yes	Stay away, no other partners	1	yes	1	1	1	1	0	0	1	0
no	no	Stay at home, no other partners	0	no	0	0	0	0	0	0	0	0
no	yes	Stay at home, no other partners	0	no	0	0	0	0	0	0	0	0
no		Stay at home, no other partners	0	no	0	0	0	0	0	0	0	0
no	no	Stay at home, no other partners	0	no	0	0	0	0	0	0	0	0
no	yes	Stay at home, no other partners	0	no	0	0	0	0	0	0	0	0
no	yes	Stay at home, no other partners	0	no	0	0	0	0	0	0	0	0
no	yes	Stay at home, no other partners	0	no	0	0	0	0	0	0	0	0
no	yes	Stay at home, no other partners	0	no	0	0	0	0	0	0	0	0
no	no	Stay at home, no other partners	0	no	0	0	0	0	0	0	0	0
no	no	Stay at home, no other partners	0	no	0	0	0	0	0	0	0	0
no	yes	Stay at home, no other partners	0	no	0	0	0	0	0	0	0	0
no	yes	Stay at home, no other partners	0	no	0	0	0	0	0	0	0	0
no	no	Stay at home, no other partners	0	no	0	0	0	0	0	0	0	0
no	no	Stay at home, no other partners	0	no	0	0	0	0	0	0	0	0
no	no	Stay at home, no other partners	0	no	0	0	0	0	0	0	0	0
no	no	Stay at home, no other partners	0	no	0	0	0	0	0	0	0	0
no	yes	Stay at home, no other partners	0	no	0	0	0	0	0	0	0	0
no	yes	Stay at home, no other partners	0	no	0	0	0	0	0	0	0	0
no	no	Stay at home, no other partners	0	no	0	0	0	0	0	0	0	0
no	no	Stay at home, no other partners	0	no	0	0	0	0	0	0	0	0
no	no	Stay at home, no other partners	0	no	0	0	0	0	0	0	0	0
no	yes	Stay at home, no other partners	0	no	0	0	0	0	0	0	0	0
no	yes	Stay at home, no other partners	0	no	0	0	0	0	0	0	0	0
no	yes	Stay at home, no other partners	0	no	0	0	0	0	0	0	0	0
no	yes	Stay at home, no other partners	0	no	0	0	0	0	0	0	0	0
no	no	Stay at home, no other partners	0	no	0	0	0	0	0	0	0	0
no	yes	Stay at home, no other partners	0	no	0	0	0	0	0	0	0	0
no	no	Stay at home, no other partners	0	no	0	0	0	0	0	0	0	0
no	no	Stay at home, no other partners	0	no	0	0	0	0	0	0	0	0
no	yes	Stay at home, no other partners	0	no	0	0	0	0	0	0	0	0
no	yes	Stay at home, no other partners	0	no	0	0	0	0	0	0	0	0
no	no	Stay at home, no other partners	0	no	0	0	0	0	0	0	0	0
no	no	Stay at home, no other partners	0	no	0	0	0	0	0	0	0	0
no	yes	Stay at home, no other partners	0	no	0	0	0	0	0	0	0	0
no	yes	Stay at home, no other partners	0	no	0	0	0	0	0	0	0	0
no	yes	Stay at home, no other partners	0	no	0	0	0	0	0	0	0	0
no	yes	Stay at home, no other partners	0	no	0	0	0	0	0	0	0	0

no	no	Stay at home, no other partners	0	no	0	0	0	0	0	0	0	0
no	no	Stay at home, no other partners	0	no	0	0	0	0	0	0	0	0
no	yes	Stay at home, no other partners	0	no	0	0	0	0	0	0	0	0
no	yes	Stay at home, no other partners	0	no	0	0	0	0	0	0	0	0
no	yes	Stay at home, no other partners	0	no	0	0	0	0	0	0	0	0
no	no	Stay at home, no other partners	0	no	0	0	0	0	0	0	0	0
no	yes	Stay at home, no other partners	0	no	0	0	0	0	0	0	0	0
no	yes	Stay at home, no other partners	0	no	0	0	0	0	0	0	0	0
no	no	Stay at home, no other partners	0	no	0	0	0	0	0	0	0	0
no	no	Stay at home, no other partners	0	no	0	0	0	0	0	0	0	0
no	no	Stay at home, no other partners	0	no	0	0	0	0	0	0	0	0
no	no	Stay at home, no other partners	0	no	0	0	0	0	0	0	0	0
no	yes	Stay at home, no other partners	0	no	0	0	0	0	0	0	0	0
no	no	Stay at home, no other partners	0	no	0	0	0	0	0	0	0	0
no	no	Stay at home, no other partners	0	no	0	0	0	0	0	0	0	0
		no current partner	1	yes	1	1	1	0	0	0	1	0
no	no	Stay at home, no other partners	0	no	0	0	0	0	0	0	0	0
no	no	Stay at home, no other partners	0	no	0	0	0	0	0	0	0	0
no	yes	Stay at home, no other partners	0	no	0	0	0	0	0	0	0	0
no	yes	Stay at home, no other partners	0	no	0	0	0	0	0	0	0	0
no	yes	Stay at home, no other partners	0	no	0	0	0	0	0	0	0	0
no	yes	Stay at home, no other partners	0	no	0	0	0	0	0	0	0	0
no	yes	Stay at home, no other partners	0	no	0	0	0	0	0	0	0	0
no		Stay at home, no other partners	0	no	0	0	0	0	0	0	0	0
no	yes	Stay at home, no other partners	0	no	0	0	0	0	0	0	0	0
no	yes	Stay at home, no other partners	0	no	0	0	0	0	0	0	0	0
no	yes	Stay at home, no other partners	0	no	0	0	0	0	0	0	0	0
no	no	Stay at home, no other partners	0	no	0	0	0	0	0	0	0	0
no	yes	Stay at home, no other partners	0	no	0	0	0	0	0	0	0	0
no	yes	Stay at home, no other partners	0	no	0	0	0	0	0	0	0	0
no	yes	Stay at home, no other partners	0	no	0	0	0	0	0	0	0	0
no	yes	Stay at home, no other partners	0	no	0	0	0	0	0	0	0	0
no	yes	Stay at home, no other partners	0	no	0	0	0	0	0	0	0	0
no	yes	Stay at home, no other partners	0	no	0	0	0	0	0	0	0	0
no	yes	Stay at home, no other partners	0	no	0	0	0	0	0	0	0	0
no	yes	Stay at home, no other partners	0	no	0	0	0	0	0	0	0	0
no	yes	Stay at home, no other partners	0	no	0	0	0	0	0	0	0	0
no	yes	Stay at home, no other partners	0	no	0	0	0	0	0	0	0	0
no	yes	Stay at home, no other partners	0	no	0	0	0	0	0	0	0	0
no	yes	Stay at home, no other partners	0	no	0	0	0	0	0	0	0	0

no	yes	Stay at home, has other partners	0	no	0	0	0	0	0	0	0	0
no	no	Stay at home, has other partners	0	no	0	0	0	0	0	0	0	0
no	no	Stay at home, has other partners	0	no	0	0	0	0	0	0	0	0
no	yes	Stay at home, no other partners	1	yes	1	1	1	0	0	0	0	1
yes	yes	Stay at home, has other partners	0	no	0	0	0	0	0	0	0	0
	yes	Stay at home, has other partners	0	no	0	0	0	0	0	0	0	0
no	yes	Stay at home, has other partners	0	no	0	0	0	0	0	0	0	0
no	yes	Stay at home, has other partners	0	no	0	0	0	0	0	0	0	0
no	yes	Stay at home, has other partners	0	no	0	0	0	0	0	0	0	0
no		Stay at home, has other partners	1	yes	0	0	0	0	0	0	0	0
no	yes	Stay at home, has other partners	0	no	0	0	0	0	0	0	0	0
no	yes	Stay at home, has other partners	0	no	0	0	0	0	0	0	0	0
no	yes	Stay at home, has other partners	0	no	0	0	0	0	0	0	0	0
no	yes	Stay at home, has other partners	0	no	0	0	0	0	0	0	0	0
	yes	Stay at home, has other partners	0	no	0	0	0	0	0	0	0	0
no	no	Stay at home, has other partners	0	no	0	0	0	0	0	0	0	0
no	no	Stay at home, has other partners	0	no	0	0	0	0	0	0	0	0
no	no	Stay at home, has other partners	0	no	0	0	0	0	0	0	0	0
no	yes	Stay at home, has other partners	0	no	0	0	0	0	0	0	0	0
no	yes	Stay at home, has other partners	0	no	0	0	0	0	0	0	0	0
no	yes	Stay at home, has other partners	0	no	0	0	0	0	0	0	0	0
no	yes	Stay at home, has other partners	0	no	0	0	0	0	0	0	0	0
no	no	Stay at home, has other partners	0	no	0	0	0	0	0	0	0	0
no	no	Stay at home, has other partners	0	no	0	0	0	0	0	0	0	0
no	no	Stay at home, has other partners	0	no	0	0	0	0	0	0	0	0
no	yes	Stay away, no other partners	1	yes	1	1	1	0	0	0	0	1
no	yes	Stay at home, has other partners	1	yes	0	0	0	0	0	0	0	0
no	yes	Stay at home, has other partners	0	no	0	0	0	0	0	0	0	0
no	yes	Stay at home, has other partners	0	no	0	0	0	0	0	0	0	0
no	yes	Stay at home, has other partners	0	no	0	0	0	0	0	0	0	0
no	yes	Stay at home, has other partners	0	no	0	0	0	0	0	0	0	0
no	no	Stay at home, has other partners	0	no	0	0	0	0	0	0	0	0
no	yes	Stay at home, has other partners	0	no	0	0	0	0	0	0	0	0
no	no	Stay at home, has other partners	0	no	0	0	0	0	0	0	0	0
no	yes	Stay at home, has other partners	0	no	0	0	0	0	0	0	0	0
no	yes	Stay at home, has other partners	0	no	0	0	0	0	0	0	0	0
no	yes	Stay at home, has other partners	0	no	0	0	0	0	0	0	0	0
no	no	Stay at home, has other partners	0	no	0	0	0	0	0	0	0	0
no	yes	Stay at home, has other partners	0	no	0	0	0	0	0	0	0	0
no	yes	Stay at home, has other partners	0	no	0	0	0	0	0	0	0	0

no	no	Stay at home, has other partners	0	no	0	0	0	0	0	0	0	0
no	yes	Stay at home, has other partners	0	no	0	0	0	0	0	0	0	0
no	yes	Stay at home, has other partners	0	no	0	0	0	0	0	0	0	0
no	no	Stay away, no other partners	0	no	0	0	0	0	0	0	0	0
no	yes	Stay away, no other partners	0	no	0	0	0	0	0	0	0	0
no	no	Stay away, no other partners	0	no	0	0	0	0	0	0	0	0
no	no	Stay away, no other partners	0	no	0	0	0	0	0	0	0	0
no	no	Stay away, no other partners	0	no	0	0	0	0	0	0	0	0
no	yes	Stay away, no other partners	1	yes	0	0	1	1	0	0	0	0
no	yes	Stay away, no other partners	0	no	0	0	0	0	0	0	0	0
no	no	Stay away, no other partners	0	no	0	0	0	0	0	0	0	0
no	yes	Stay away, no other partners	0	no	0	0	0	0	0	0	0	0
no	yes	Stay away, no other partners	0	no	0	0	0	0	0	0	0	0
no	no	Stay away, no other partners	0	no	0	0	0	0	0	0	0	0
no	yes	Stay away, no other partners	0	no	0	0	0	0	0	0	0	0
no	yes	Stay away, no other partners	0	no	0	0	0	0	0	0	0	0
no	yes	Stay away, no other partners	0	no	0	0	0	0	0	0	0	0
no	yes	Stay away, no other partners	0	no	0	0	0	0	0	0	0	0
no	yes	Stay away, no other partners	0	no	0	0	0	0	0	0	0	0
no	yes	Stay away, has other partners	1	yes	0	0	1	1	0	0	0	0
no	yes	Stay away, no other partners	0	no	0	0	0	0	0	0	0	0
no	no	Stay away, no other partners	0	no	0	0	0	0	0	0	0	0
no	yes	Stay away, no other partners	0	no	0	0	0	0	0	0	0	0
no	yes	Stay away, no other partners	0	no	0	0	0	0	0	0	0	0
no	yes	Stay away, no other partners	0	no	0	0	0	0	0	0	0	0
no	no	Stay away, no other partners	1	yes	0	0	1	1	0	0	0	0
no	yes	Stay at home, no other partners	1	yes	0	0	1	1	0	0	0	0
no	yes	Stay away, no other partners	0	no	0	0	0	0	0	0	0	0
no	no	Stay away, no other partners	0	no	0	0	0	0	0	0	0	0
no	yes	Stay away, no other partners	0	no	0	0	0	0	0	0	0	0
no	no	Stay away, no other partners	0	no	0	0	0	0	0	0	0	0
no	no	Stay away, no other partners	0	no	0	0	0	0	0	0	0	0
no	no	Stay away, no other partners	0	no	0	0	0	0	0	0	0	0
no	no	Stay away, no other partners	0	no	0	0	0	0	0	0	0	0
no	no	Stay away, no other partners	0	no	0	0	0	0	0	0	0	0
no	yes	Stay away, no other partners	0	no	0	0	0	0	0	0	0	0
no		no current partner	1	yes	0	0	1	1	0	0	0	0
no	no	Stay at home, no other partners	1	yes	0	1	1	0	1	0	0	0
no	yes	Stay away, no other partners	0	no	0	0	0	0	0	0	0	0
no	no	Stay away, no other partners	0	no	0	0	0	0	0	0	0	0

no	no	Stay away, has other partners	0	no	0	0	0	0	0	0	0	0
no	no	Stay away, has other partners	0	no	0	0	0	0	0	0	0	0
no	yes	Stay away, has other partners	0	no	0	0	0	0	0	0	0	0
no	yes	Stay at home, no other partners	1	yes	0	0	1	1	0	0	0	0
no	yes	Stay away, has other partners	0	no	0	0	0	0	0	0	0	0
no	no	Stay away, has other partners	0	no	0	0	0	0	0	0	0	0
no	yes	Stay away, has other partners	0	no	0	0	0	0	0	0	0	0
no	yes	Stay away, has other partners	0	no	0	0	0	0	0	0	0	0
no	no	Stay away, has other partners	0	no	0	0	0	0	0	0	0	0
no	no	Stay at home, no other partners	1	yes	0	0	1	1	0	0	0	0
no	yes	Stay away, has other partners	0	no	0	0	0	0	0	0	0	0
no		Stay away, has other partners	0	no	0	0	0	0	0	0	0	0
no	yes	Stay at home, no other partners	1	yes	0	0	1	1	0	0	0	0
no	yes	Stay away, has other partners	0	no	0	0	0	0	0	0	0	0
no	yes	Stay away, has other partners	0	no	0	0	0	0	0	0	0	0
no	yes	Stay away, has other partners	0	no	0	0	0	0	0	0	0	0
no	no	Stay away, has other partners	1	yes	0	0	0	0	0	0	0	0
no	yes	Stay away, has other partners	0	no	0	0	0	0	0	0	0	0
no	yes	Stay away, has other partners	0	no	0	0	0	0	0	0	0	0
no	no	Stay away, has other partners	0	no	0	0	0	0	0	0	0	0
no	yes	Stay away, has other partners	0	no	0	0	0	0	0	0	0	0
no		no current partner	0	no	0	0	0	0	0	0	0	0
no		no current partner	0	no	0	0	0	0	0	0	0	0
no	no	Stay at home, no other partners	1	yes	0	0	1	1	0	0	0	0
		no current partner	0	no	0	0	0	0	0	0	0	0
no		no current partner	0	no	0	0	0	0	0	0	0	0
no	yes	Stay away, has other partners	1	yes	0	0	1	1	0	0	0	0
no		no current partner	1	yes	0	0	0	0	0	0	0	0
no		no current partner	1	yes	0	0	1	1	0	0	0	0
no		no current partner	0	no	0	0	0	0	0	0	0	0
no		no current partner	0	no	0	0	0	0	0	0	0	0
no		no current partner	0	no	0	0	0	0	0	0	0	0
no		no current partner	0	no	0	0	0	0	0	0	0	0
no		no current partner	0	no	0	0	0	0	0	0	0	0
no		no current partner	0	no	0	0	0	0	0	0	0	0
		no current partner	0	no	0	0	0	0	0	0	0	0
no		no current partner	0	no	0	0	0	0	0	0	0	0
no		no current partner	0	no	0	0	0	0	0	0	0	0
no		no current partner	0	no	0	0	0	0	0	0	0	0
no		no current partner	0	no	0	0	0	0	0	0	0	0

no		no current partner	1	yes	0	0	0	0	0	0	0	0
no		no current partner	0	no	0	0	0	0	0	0	0	0
no		no current partner	0	no	0	0	0	0	0	0	0	0
no		no current partner	0	no	0	0	0	0	0	0	0	0
no		no current partner	1	yes	0	0	1	1	0	0	0	0
no		no current partner	0	no	0	0	0	0	0	0	0	0
no		no current partner	0	no	0	0	0	0	0	0	0	0
no		no current partner	0	no	0	0	0	0	0	0	0	0
no		no current partner	0	no	0	0	0	0	0	0	0	0
no	yes	Stay away, no other partners	1	no	0	1	1	0	1	0	0	0
no		no current partner	0	no	0	0	0	0	0	0	0	0
no		no current partner	0	no	0	0	0	0	0	0	0	0
no		no current partner	0	no	0	0	0	0	0	0	0	0
no		no current partner	0	no	0	0	0	0	0	0	0	0
no		no current partner	1	yes	0	0	0	1	0	0	0	0
no		no current partner	0	no	0	0	0	0	0	0	0	0
no		no current partner	0	no	0	0	0	0	0	0	0	0
no		no current partner	0	no	0	0	0	0	0	0	0	0
no		no current partner	1	no	0	1	1	0	1	0	0	0
no		no current partner	0	no	0	0	0	0	0	0	0	0
			0	no	0	0	0	0	0	0	0	0
			0	no	0	0	0	0	0	0	0	0
			0	no	0	0	0	0	0	0	0	0
			0	no	0	0	0	0	0	0	0	0
			0	no	0	0	0	0	0	0	0	0
			0	no	0	0	0	0	0	0	0	0
no			0	no	0	0	0	0	0	0	0	0
no	yes	Stay away, no other partners	0	no	0	0	0	0	0	0	0	0
no	yes	Stay away, has other partners	0	no	0	0	0	0	0	0	0	0
no		no current partner	0	no	0	0	0	0	0	0	0	0
no	no	Stay at home, no other partners	0	no	0	0	0	0	0	0	0	0
no	yes	Stay at home, no other partners	0	no	0	0	0	0	0	0	0	0
no		no current partner	0	no	0	0	0	0	0	0	0	0
no	yes	Stay away, has other partners	0	no	0	0	0	0	0	0	0	0
no		no current partner	0	no	0	0	0	0	0	0	0	0
no	yes	Stay at home, no other partners	0	no	0	0	0	0	0	0	0	0
no	yes	Stay at home, no other partners	0	no	0	0	0	0	0	0	0	0
no		no current partner	0	no	0	0	0	0	0	0	0	0
no	yes	Stay at home, no other partners	0	no	0	0	0	0	0	0	0	0
no	yes	Stay at home, no other partners	0	no	0	0	0	0	0	0	0	0

no	yes	Stay at home, has other partners	0	no	0	0	0	0	0	0	0	0
no		no current partner	0	no	0	0	0	0	0	0	0	0
no	yes	Stay at home, no other partners	0	no	0	0	0	0	0	0	0	0
no	yes	Stay at home, no other partners	0	no	0	0	0	0	0	0	0	0
no	yes	Stay at home, no other partners	1	no	0	1	1	0	1	0	0	0
no	yes	Stay at home, no other partners	0	no	0	0	0	0	0	0	0	0
no	yes	Stay at home, no other partners	0	no	0	0	0	0	0	0	0	0
no	yes	Stay at home, no other partners	0	no	0	0	0	0	0	0	0	0
no	yes	Stay away, no other partners	0	no	0	0	0	0	0	0	0	0
no	yes	Stay at home, no other partners	0	no	0	0	0	0	0	0	0	0
no	yes	Stay at home, no other partners	0	no	0	0	0	0	0	0	0	0
no	no	Stay at home, no other partners	0	no	0	0	0	0	0	0	0	0
no	no	Stay at home, no other partners	0	no	0	0	0	0	0	0	0	0
no	yes	Stay away, has other partners	0	no	0	0	0	0	0	0	0	0
no	no	Stay at home, no other partners	0	no	0	0	0	0	0	0	0	0
no	no	Stay at home, has other partners	0	no	0	0	0	0	0	0	0	0
no	yes	Stay at home, no other partners	0	no	0	0	0	0	0	0	0	0
no	yes	Stay away, no other partners	0	no	0	0	0	0	0	0	0	0
yes	yes	Stay away, no other partners	0	no	0	0	0	0	0	0	0	0
no	yes	Stay at home, no other partners	0	no	0	0	0	0	0	0	0	0
no	yes	Stay at home, no other partners	0	no	0	0	0	0	0	0	0	0
no		no current partner	0	no	0	0	0	0	0	0	0	0
no	yes	Stay at home, no other partners	0	no	0	0	0	0	0	0	0	0
no	yes	Stay at home, no other partners	0	no	0	0	0	0	0	0	0	0
no	yes	Stay at home, has other partners	0	no	0	0	0	0	0	0	0	0
no	yes	Stay at home, no other partners	0	no	0	0	0	0	0	0	0	0
no	yes	Stay at home, has other partners	0	no	0	0	0	0	0	0	0	0
no		no current partner	0	no	0	0	0	0	0	0	0	0
no	yes	Stay at home, no other partners	0	no	0	0	0	0	0	0	0	0
no	yes	Stay at home, no other partners	0	no	0	0	0	0	0	0	0	0
no	yes	Stay at home, has other partners	0	no	0	0	0	0	0	0	0	0
no	yes	Stay at home, no other partners	0	no	0	0	0	0	0	0	0	0
no	yes	Stay away, no other partners	1	yes	1	1	1	0	0	0	0	1
no	no	Stay at home, no other partners	0	no	0	0	0	0	0	0	0	0
no	yes	Stay at home, no other partners	0	no	0	0	0	0	0	0	0	0
no	yes	Stay at home, no other partners	0	no	0	0	0	0	0	0	0	0
no	yes	Stay at home, no other partners	0	no	0	0	0	0	0	0	0	0
no	yes	Stay at home, no other partners	0	no	0	0	0	0	0	0	0	0
no	no	Stay away, has other partners	0	no	0	0	0	0	0	0	0	0
no	yes	Stay at home, no other partners	0	no	0	0	0	0	0	0	0	0

no	yes	Stay at home, no other partners	0	no	0	0	0	0	0	0	0	0
no	yes	Stay at home, no other partners	0	no	0	0	0	0	0	0	0	0
no	yes	Stay at home, has other partners	0	no	0	0	0	0	0	0	0	0
no	no	Stay at home, no other partners	0	no	0	0	0	0	0	0	0	0
no		no current partner	1	yes	0	0	0	0	0	0	0	0
no	no	Stay at home, no other partners	0	no	0	0	0	0	0	0	0	0
no		Stay at home, no other partners	0	no	0	0	0	0	0	0	0	0
no	no	Stay away, no other partners	0	no	0	0	0	0	0	0	0	0
no	no	Stay away, no other partners	0	no	0	0	0	0	0	0	0	0
no	no	Stay at home, no other partners	0	no	0	0	0	0	0	0	0	0
no	no	Stay at home, no other partners	0	no	0	0	0	0	0	0	0	0
no	no	Stay at home, no other partners	0	no	0	0	0	0	0	0	0	0
no	yes	Stay at home, no other partners	0	no	0	0	0	0	0	0	0	0
no	no	Stay at home, no other partners	0	no	0	0	0	0	0	0	0	0
no	no	Stay at home, no other partners	0	no	0	0	0	0	0	0	0	0
no	no	Stay at home, no other partners	0	no	0	0	0	0	0	0	0	0
no	no	Stay at home, no other partners	0	no	0	0	0	0	0	0	0	0
no	no	Stay at home, no other partners	0	no	0	0	0	0	0	0	0	0
no	no	Stay at home, no other partners	0	no	0	0	0	0	0	0	0	0
no	yes	Stay at home, no other partners	0	no	0	0	0	0	0	0	0	0
no	yes	Stay at home, no other partners	0	no	0	0	0	0	0	0	0	0
no	yes	Stay at home, no other partners	0	no	0	0	0	0	0	0	0	0
no	yes	Stay at home, no other partners	0	no	0	0	0	0	0	0	0	0
no	no	Stay at home, no other partners	0	no	0	0	0	0	0	0	0	0
no	yes	Stay at home, no other partners	0	no	0	0	0	0	0	0	0	0
no	no	Stay at home, no other partners	0	no	0	0	0	0	0	0	0	0
no	yes	Stay at home, has other partners	0	no	0	0	0	0	0	0	0	0
no	yes	Stay at home, no other partners	0	no	0	0	0	0	0	0	0	0
no	yes	Stay at home, no other partners	0	no	0	0	0	0	0	0	0	0
no	yes	Stay away, has other partners	0	no	0	0	0	0	0	0	0	0
no		Stay at home, no other partners	0	no	0	0	0	0	0	0	0	0
no	yes	Stay at home, no other partners	0	no	0	0	0	0	0	0	0	0
no	no	Stay at home, no other partners	1	yes	0	0	0	0	0	0	0	0
yes		no current partner	0	no	0	0	0	0	0	0	0	0
no	yes	Stay at home, no other partners	0	no	0	0	0	0	0	0	0	0
no		no current partner	0	no	0	0	0	0	0	0	0	0
no	yes	Stay at home, no other partners	0	no	0	0	0	0	0	0	0	0
no	no	Stay at home, no other partners	0	no	0	0	0	0	0	0	0	0
no	yes	Stay at home, no other partners	0	no	0	0	0	0	0	0	0	0
no	no	Stay at home, no other partners	0	no	0	0	0	0	0	0	0	0

no	no	Stay at home, no other partners	0	no	0	0	0	0	0	0	0	0
no	yes	Stay at home, no other partners	0	no	0	0	0	0	0	0	0	0
no	no	Stay at home, no other partners	0	no	0	0	0	0	0	0	0	0
no	no	Stay at home, no other partners	0	no	0	0	0	0	0	0	0	0
no	yes	Stay at home, no other partners	0	no	0	0	0	0	0	0	0	0
no	no	Stay at home, no other partners	0	no	0	0	0	0	0	0	0	0
no	no	Stay at home, has other partners	0	no	0	0	0	0	0	0	0	0
no	yes	Stay at home, no other partners	0	no	0	0	0	0	0	0	0	0
no	yes	Stay at home, no other partners	0	no	0	0	0	0	0	0	0	0
no	yes	Stay at home, no other partners	0	no	0	0	0	0	0	0	0	0
no	yes	Stay at home, no other partners	0	no	0	0	0	0	0	0	0	0
no	no	Stay away, has other partners	1	yes	0	0	1	1	0	0	0	0
no	no	Stay at home, no other partners	0	no	0	0	0	0	0	0	0	0
no	yes	Stay at home, no other partners	0	no	0	0	0	0	0	0	0	0
no	no	Stay away, no other partners	0	no	0	0	0	0	0	0	0	0
no	yes	Stay at home, no other partners	0	no	0	0	0	0	0	0	0	0
no	yes	Stay at home, no other partners	0	no	0	0	0	0	0	0	0	0
no		no current partner	0	no	0	0	0	0	0	0	0	0
yes	yes	Stay away, no other partners	0	no	0	0	0	0	0	0	0	0
no	yes	Stay at home, no other partners	0	no	0	0	0	0	0	0	0	0
no	no	Stay away, no other partners	0	no	0	0	0	0	0	0	0	0
no	no	Stay at home, no other partners	0	no	0	0	0	0	0	0	0	0
no	yes	Stay at home, no other partners	0	no	0	0	0	0	0	0	0	0
no	no	Stay at home, no other partners	0	no	0	0	0	0	0	0	0	0
no		Stay at home, no other partners	0	no	0	0	0	0	0	0	0	0
no	yes	Stay at home, no other partners	0	no	0	0	0	0	0	0	0	0
no	yes	Stay at home, no other partners	0	no	0	0	0	0	0	0	0	0
no	yes	Stay at home, no other partners	0	no	0	0	0	0	0	0	0	0
no	yes	Stay at home, no other partners	0	no	0	0	0	0	0	0	0	0
no	yes	Stay at home, no other partners	0	no	0	0	0	0	0	0	0	0
no		Stay at home, no other partners	1	yes	0	0	0	0	0	0	0	0
no	yes	Stay away, no other partners	0	no	0	0	0	0	0	0	0	0
no	yes	Stay at home, no other partners	0	no	0	0	0	0	0	0	0	0
no	yes	Stay at home, no other partners	0	no	0	0	0	0	0	0	0	0
no	yes	Stay at home, no other partners	0	no	0	0	0	0	0	0	0	0
no	yes	Stay at home, no other partners	0	no	0	0	0	0	0	0	0	0
no	yes	Stay at home, no other partners	0	no	0	0	0	0	0	0	0	0
no	yes	Stay at home, no other partners	0	no	0	0	0	0	0	0	0	0
no	yes	Stay at home, no other partners	0	no	0	0	0	0	0	0	0	0
no		no current partner	0	no	0	0	0	0	0	0	0	0

no	yes	Stay at home, no other partners	0	no	0	0	0	0	0	0	0	0
no	yes	Stay at home, no other partners	0	no	0	0	0	0	0	0	0	0
no	yes	Stay at home, no other partners	0	no	0	0	0	0	0	0	0	0
no	no	Stay at home, no other partners	0	no	0	0	0	0	0	0	0	0
no		no current partner	0	no	0	0	0	0	0	0	0	0
no	no	Stay at home, no other partners	0	no	0	0	0	0	0	0	0	0
no	no	Stay at home, no other partners	0	no	0	0	0	0	0	0	0	0
no	yes	Stay at home, no other partners	0	no	0	0	0	0	0	0	0	0
no	yes	Stay at home, no other partners	0	no	0	0	0	0	0	0	0	0
no	yes	Stay at home, no other partners	0	no	0	0	0	0	0	0	0	0
no	yes	Stay at home, no other partners	0	no	0	0	0	0	0	0	0	0
no	yes	Stay at home, no other partners	0	no	0	0	0	0	0	0	0	0
no	yes	Stay at home, no other partners	0	no	0	0	0	0	0	0	0	0
no	yes	Stay at home, no other partners	0	no	0	0	0	0	0	0	0	0
no	yes	Stay at home, no other partners	0	no	0	0	0	0	0	0	0	0
no	yes	Stay at home, no other partners	0	no	0	0	0	0	0	0	0	0
no	yes	Stay at home, no other partners	0	no	0	0	0	0	0	0	0	0
no	no	Stay at home, no other partners	0	no	0	0	0	0	0	0	0	0
no	no	Stay at home, no other partners	0	no	0	0	0	0	0	0	0	0
no	no	Stay at home, no other partners	0	no	0	0	0	0	0	0	0	0
no	yes	Stay away, no other partners	0	no	0	0	0	0	0	0	0	0
no	yes	Stay at home, no other partners	0	no	0	0	0	0	0	0	0	0
no	no	Stay at home, no other partners	0	no	0	0	0	0	0	0	0	0
no	no	Stay at home, no other partners	0	no	0	0	0	0	0	0	0	0
no	yes	Stay at home, no other partners	0	no	0	0	0	0	0	0	0	0
no	no	Stay at home, no other partners	0	no	0	0	0	0	0	0	0	0
no	no	Stay at home, no other partners	0	no	0	0	0	0	0	0	0	0
no	yes	Stay at home, no other partners	0	no	0	0	0	0	0	0	0	0
no	yes	Stay at home, has other partners	0	no	0	0	0	0	0	0	0	0
no	yes	Stay at home, no other partners	0	no	0	0	0	0	0	0	0	0
no	yes	Stay at home, no other partners	0	no	0	0	0	0	0	0	0	0
no	yes	Stay at home, no other partners	0	no	0	0	0	0	0	0	0	0
no	yes	Stay at home, no other partners	0	no	0	0	0	0	0	0	0	0
no	yes	Stay away, has other partners	0	no	0	0	0	0	0	0	0	0
no	yes	Stay at home, no other partners	0	no	0	0	0	0	0	0	0	0
no	no	Stay at home, no other partners	0	no	0	0	0	0	0	0	0	0
no	no	Stay at home, no other partners	0	no	0	0	0	0	0	0	0	0
no		no current partner	0	no	0	0	0	0	0	0	0	0
no	yes	Stay at home, no other partners	0	no	0	0	0	0	0	0	0	0
no	yes	Stay at home, has other partners	0	no	0	0	0	0	0	0	0	0

no	yes	Stay at home, no other partners	0	no	0	0	0	0	0	0	0	0
no	yes	Stay at home, no other partners	0	no	0	0	0	0	0	0	0	0
no	yes	Stay at home, no other partners	0	no	0	0	0	0	0	0	0	0
no	no	Stay at home, no other partners	0	no	0	0	0	0	0	0	0	0
no	no	Stay at home, no other partners	1	no	0	1	1	0	1	0	0	0
no	no	Stay at home, no other partners	0	no	0	0	0	0	0	0	0	0
no	yes	Stay at home, no other partners	0	no	0	0	0	0	0	0	0	0
no	yes	Stay away, no other partners	0	no	0	0	0	0	0	0	0	0
no	no	Stay at home, no other partners	0	no	0	0	0	0	0	0	0	0
no	yes	Stay at home, no other partners	0	no	0	0	0	0	0	0	0	0
no	yes	Stay at home, has other partners	0	no	0	0	0	0	0	0	0	0
no	no	Stay at home, no other partners	0	no	0	0	0	0	0	0	0	0
no	yes	Stay at home, no other partners	0	no	0	0	0	0	0	0	0	0
no	yes	Stay at home, no other partners	0	no	0	0	0	0	0	0	0	0
no	yes	Stay at home, no other partners	0	no	0	0	0	0	0	0	0	0
no	yes	Stay at home, no other partners	0	no	0	0	0	0	0	0	0	0
no	yes	Stay at home, no other partners	0	no	0	0	0	0	0	0	0	0
no	yes	Stay at home, no other partners	0	no	0	0	0	0	0	0	0	0
no	yes	Stay at home, no other partners	0	no	0	0	0	0	0	0	0	0
no	yes	Stay at home, no other partners	0	no	0	0	0	0	0	0	0	0
no	yes	Stay at home, no other partners	0	no	0	0	0	0	0	0	0	0
no	yes	Stay away, no other partners	0	no	0	0	0	0	0	0	0	0
no	yes	Stay away, no other partners	0	no	0	0	0	0	0	0	0	0
no	yes	Stay away, no other partners	0	no	0	0	0	0	0	0	0	0
no	yes	Stay at home, no other partners	0	no	0	0	0	0	0	0	0	0
no	yes	Stay at home, no other partners	0	no	0	0	0	0	0	0	0	0
no	yes	Stay away, no other partners	0	no	0	0	0	0	0	0	0	0
no	yes	Stay at home, has other partners	0	no	0	0	0	0	0	0	0	0
no	no	Stay at home, no other partners	1	yes	0	0	1	1	0	0	0	0
no	yes	Stay at home, no other partners	0	no	0	0	0	0	0	0	0	0
no	no	Stay at home, no other partners	0	no	0	0	0	0	0	0	0	0
no	yes	Stay at home, no other partners	0	no	0	0	0	0	0	0	0	0
no	no	Stay at home, no other partners	0	no	0	0	0	0	0	0	0	0
no	yes	Stay at home, has other partners	0	no	0	0	0	0	0	0	0	0
no	no	Stay away, no other partners	0	no	0	0	0	0	0	0	0	0
no	no	Stay away, no other partners	0	no	0	0	0	0	0	0	0	0
no	yes	Stay at home, no other partners	0	no	0	0	0	0	0	0	0	0
no	yes	Stay at home, no other partners	1	yes	0	0	1	1	0	0	0	0
no	yes	Stay at home, no other partners	0	no	0	0	0	0	0	0	0	0
no		no current partner	0	no	0	0	0	0	0	0	0	0

no		Stay away, no other partners	0	no	0	0	0	0	0	0	0	0
no	yes	Stay at home, no other partners	0	no	0	0	0	0	0	0	0	0
no	yes	Stay at home, no other partners	0	no	0	0	0	0	0	0	0	0
no		no current partner	0	no	0	0	0	0	0	0	0	0
no	yes	Stay at home, no other partners	0	no	0	0	0	0	0	0	0	0
no		no current partner	0	no	0	0	0	0	0	0	0	0
no	yes	Stay at home, no other partners	0	no	0	0	0	0	0	0	0	0
no	yes	Stay at home, no other partners	0	no	0	0	0	0	0	0	0	0
no	yes	Stay at home, no other partners	0	no	0	0	0	0	0	0	0	0
no	yes	Stay at home, no other partners	0	no	0	0	0	0	0	0	0	0
no	yes	Stay at home, no other partners	0	no	0	0	0	0	0	0	0	0
no	no	Stay at home, no other partners	0	no	0	0	0	0	0	0	0	0
no	yes	Stay at home, no other partners	0	no	0	0	0	0	0	0	0	0
no	yes	Stay away, no other partners	1	yes	1	1	1	0	0	0	1	0
no	yes	Stay at home, no other partners	0	no	0	0	0	0	0	0	0	0
no	yes	Stay at home, no other partners	0	no	0	0	0	0	0	0	0	0
no		no current partner	0	no	0	0	0	0	0	0	0	0
no	no	Stay away, no other partners	0	no	0	0	0	0	0	0	0	0
no	yes	Stay at home, no other partners	0	no	0	0	0	0	0	0	0	0
no	yes	Stay at home, no other partners	0	no	0	0	0	0	0	0	0	0
no	no	Stay away, no other partners	0	no	0	0	0	0	0	0	0	0
no	yes	Stay at home, no other partners	0	no	0	0	0	0	0	0	0	0
no		no current partner	0	no	0	0	0	0	0	0	0	0
no	no	Stay at home, no other partners	0	no	0	0	0	0	0	0	0	0
no	yes	Stay away, has other partners	0	no	0	0	0	0	0	0	0	0
no	yes	Stay at home, no other partners	0	no	0	0	0	0	0	0	0	0
no	yes	Stay at home, no other partners	0	no	0	0	0	0	0	0	0	0
no	yes	Stay at home, no other partners	0	no	0	0	0	0	0	0	0	0
no	no	Stay at home, no other partners	0	no	0	0	0	0	0	0	0	0
no	no	Stay at home, no other partners	0	no	0	0	0	0	0	0	0	0
no	yes	Stay at home, no other partners	0	no	0	0	0	0	0	0	0	0
no	yes	Stay at home, no other partners	0	no	0	0	0	0	0	0	0	0
no	no	Stay at home, no other partners	0	no	0	0	0	0	0	0	0	0
no	yes	Stay at home, no other partners	0	no	0	0	0	0	0	0	0	0
no	yes	Stay at home, no other partners	0	no	0	0	0	0	0	0	0	0
no	no	Stay at home, no other partners	0	no	0	0	0	0	0	0	0	0
no	no	Stay at home, no other partners	0	no	0	0	0	0	0	0	0	0
no	yes	Stay away, no other partners	0	no	0	0	0	0	0	0	0	0
no	yes	Stay at home, no other partners	0	no	0	0	0	0	0	0	0	0
no	no	Stay at home, no other partners	0	no	0	0	0	0	0	0	0	0

yes	no	Stay at home, no other partners	0	no	0	0	0	0	0	0	0	0
no	yes	Stay at home, no other partners	0	no	0	0	0	0	0	0	0	0
no	yes	Stay at home, no other partners	0	no	0	0	0	0	0	0	0	0
no	no	Stay at home, no other partners	0	no	0	0	0	0	0	0	0	0
no	yes	Stay at home, no other partners	0	no	0	0	0	0	0	0	0	0
no	yes	Stay at home, has other partners	0	no	0	0	0	0	0	0	0	0
no	yes	Stay at home, no other partners	0	no	0	0	0	0	0	0	0	0
no	yes	Stay at home, no other partners	0	no	0	0	0	0	0	0	0	0
no	no	Stay at home, no other partners	0	no	0	0	0	0	0	0	0	0
no		no current partner	0	no	0	0	0	0	0	0	0	0
no	no	Stay at home, no other partners	0	no	0	0	0	0	0	0	0	0
no	yes	Stay at home, no other partners	0	no	0	0	0	0	0	0	0	0
no	yes	Stay at home, no other partners	0	no	0	0	0	0	0	0	0	0
no	no	Stay at home, no other partners	0	no	0	0	0	0	0	0	0	0
no	yes	Stay at home, no other partners	0	no	0	0	0	0	0	0	0	0
no	yes	Stay at home, has other partners	0	no	0	0	0	0	0	0	0	0
no	no	Stay at home, no other partners	0	no	0	0	0	0	0	0	0	0
no	no	Stay at home, has other partners	1	yes	0	0	1	1	0	0	0	0
no	no	Stay at home, no other partners	0	no	0	0	0	0	0	0	0	0
no	no	Stay at home, no other partners	0	no	0	0	0	0	0	0	0	0
no	no	Stay at home, no other partners	0	no	0	0	0	0	0	0	0	0
no	no	Stay at home, no other partners	0	no	0	0	0	0	0	0	0	0
no	no	Stay at home, no other partners	0	no	0	0	0	0	0	0	0	0
no	no	Stay at home, no other partners	0	no	0	0	0	0	0	0	0	0
no	no	Stay at home, no other partners	0	no	0	0	0	0	0	0	0	0
no	no	Stay at home, no other partners	0	no	0	0	0	0	0	0	0	0
no	yes	Stay at home, no other partners	0	no	0	0	0	0	0	0	0	0
no		no current partner	0	no	0	0	0	0	0	0	0	0
no	yes	Stay at home, no other partners	0	no	0	0	0	0	0	0	0	0
no	yes	Stay at home, no other partners	0	no	0	0	0	0	0	0	0	0
no	no	Stay at home, has other partners	0	no	0	0	0	0	0	0	0	0
no	yes	Stay at home, no other partners	1	yes	0	0	0	0	0	0	0	0
no	yes	Stay at home, no other partners	0	no	0	0	0	0	0	0	0	0
no		no current partner	0	no	0	0	0	0	0	0	0	0
no		no current partner	0	no	0	0	0	0	0	0	0	0
no	yes	Stay at home, has other partners	0	no	0	0	0	0	0	0	0	0
no	no	Stay away, no other partners	0	no	0	0	0	0	0	0	0	0
yes	no	Stay at home, no other partners	0	no	0	0	0	0	0	0	0	0
no	yes	Stay at home, no other partners	0	no	0	0	0	0	0	0	0	0
no	yes	Stay at home, no other partners	0	no	0	0	0	0	0	0	0	0

no	yes	Stay at home, no other partners	0	no	0	0	0	0	0	0	0	0
no	no	Stay at home, no other partners	0	no	0	0	0	0	0	0	0	0
no		Stay at home, no other partners	0	no	0	0	0	0	0	0	0	0
no	yes	Stay at home, no other partners	0	no	0	0	0	0	0	0	0	0
no	yes	Stay at home, has other partners	0	no	0	0	0	0	0	0	0	0
no	no	Stay at home, no other partners	0	no	0	0	0	0	0	0	0	0
no	no	Stay at home, has other partners	0	no	0	0	0	0	0	0	0	0
no	no	Stay away, no other partners	0	no	0	0	0	0	0	0	0	0
no	no	Stay away, has other partners	0	no	0	0	0	0	0	0	0	0
no	no	Stay at home, no other partners	0	no	0	0	0	0	0	0	0	0
no	yes	Stay at home, no other partners	0	no	0	0	0	0	0	0	0	0
no	no	Stay away, no other partners	0	no	0	0	0	0	0	0	0	0
no	yes	Stay at home, no other partners	0	no	0	0	0	0	0	0	0	0
no		no current partner	0	no	0	0	0	0	0	0	0	0
no	no	Stay at home, has other partners	0	no	0	0	0	0	0	0	0	0
no	no	Stay away, has other partners	0	no	0	0	0	0	0	0	0	0
no		Stay at home, no other partners	1	yes	0	0	0	0	0	0	0	0
yes		Stay at home, no other partners	0	no	0	0	0	0	0	0	0	0
no		no current partner	1	yes	1	1	1	0	0	0	0	1
no	yes	Stay away, no other partners	0	no	0	0	0	0	0	0	0	0
no	no	Stay at home, no other partners	0	no	0	0	0	0	0	0	0	0
yes	no	Stay at home, no other partners	0	no	0	0	0	0	0	0	0	0
no	yes	Stay away, no other partners	0	no	0	0	0	0	0	0	0	0
no	no	Stay at home, has other partners	0	no	0	0	0	0	0	0	0	0
no	yes	Stay at home, no other partners	0	no	0	0	0	0	0	0	0	0
no	yes	Stay at home, no other partners	0	no	0	0	0	0	0	0	0	0
no	yes	Stay at home, no other partners	0	no	0	0	0	0	0	0	0	0
yes	no	Stay at home, no other partners	0	no	0	0	0	0	0	0	0	0
no	yes	Stay at home, no other partners	0	no	0	0	0	0	0	0	0	0
no	no	Stay away, no other partners	0	no	0	0	0	0	0	0	0	0
no	no	Stay at home, no other partners	0	no	0	0	0	0	0	0	0	0
no	no	Stay at home, no other partners	0	no	0	0	0	0	0	0	0	0
no	yes	Stay at home, no other partners	0	no	0	0	0	0	0	0	0	0
no	no	Stay at home, no other partners	0	no	0	0	0	0	0	0	0	0
no	no	Stay away, no other partners	0	no	0	0	0	0	0	0	0	0
no	no	Stay away, no other partners	0	no	0	0	0	0	0	0	0	0
no	no	Stay at home, no other partners	0	no	0	0	0	0	0	0	0	0
no	yes	Stay at home, no other partners	1	yes	0	0	0	0	0	0	0	0
no	no	Stay at home, has other partners	0	no	0	0	0	0	0	0	0	0
no	yes	Stay at home, no other partners	0	no	0	0	0	0	0	0	0	0

no	yes	Stay at home, has other partners	0	no	0	0	0	0	0	0	0	0
no	yes	Stay at home, no other partners	0	no	0	0	0	0	0	0	0	0
no	yes	Stay away, no other partners	0	no	0	0	0	0	0	0	0	0
no		Stay at home, no other partners	1	yes	0	0	1	1	0	0	0	0
no		no current partner	0	no	0	0	0	0	0	0	0	0
no	no	Stay at home, no other partners	0	no	0	0	0	0	0	0	0	0
no	yes	Stay at home, no other partners	0	no	0	0	0	0	0	0	0	0
no	yes	Stay at home, no other partners	0	no	0	0	0	0	0	0	0	0
no	yes	Stay at home, no other partners	0	no	0	0	0	0	0	0	0	0
no	no	Stay away, has other partners	0	no	0	0	0	0	0	0	0	0
no	no	Stay at home, no other partners	0	no	0	0	0	0	0	0	0	0
no	yes	Stay at home, no other partners	0	no	0	0	0	0	0	0	0	0
no	no	Stay at home, no other partners	0	no	0	0	0	0	0	0	0	0
no	yes	Stay at home, no other partners	0	no	0	0	0	0	0	0	0	0
no	yes	Stay away, no other partners	0	no	0	0	0	0	0	0	0	0
no	yes	Stay at home, no other partners	0	no	0	0	0	0	0	0	0	0
no	yes	Stay at home, no other partners	0	no	0	0	0	0	0	0	0	0
yes	yes	Stay at home, no other partners	0	no	0	0	0	0	0	0	0	0
no	yes	Stay at home, no other partners	0	no	0	0	0	0	0	0	0	0
no	no	Stay at home, no other partners	0	no	0	0	0	0	0	0	0	0
no	no	Stay at home, no other partners	0	no	0	0	0	0	0	0	0	0
yes	no	Stay at home, no other partners	1	yes	0	0	1	1	0	0	0	0
no	yes	Stay at home, no other partners	0	no	0	0	0	0	0	0	0	0
no	yes	Stay at home, has other partners	0	no	0	0	0	0	0	0	0	0
no	no	Stay away, no other partners	0	no	0	0	0	0	0	0	0	0
no	no	Stay away, no other partners	0	no	0	0	0	0	0	0	0	0
no		Stay at home, no other partners	0	no	0	0	0	0	0	0	0	0
yes	no	Stay at home, no other partners	0	no	0	0	0	0	0	0	0	0
no	no	Stay away, no other partners	0	no	0	0	0	0	0	0	0	0
yes		Stay away, no other partners	0	no	0	0	0	0	0	0	0	0
no		no current partner	0	no	0	0	0	0	0	0	0	0
no	no	Stay at home, no other partners	1	yes	0	0	1	1	0	0	0	0
yes	yes	Stay at home, no other partners	0	no	0	0	0	0	0	0	0	0
no	yes	Stay away, no other partners	0	no	0	0	0	0	0	0	0	0
yes		Stay at home, has other partners	0	no	0	0	0	0	0	0	0	0
no	no	Stay at home, no other partners	0	no	0	0	0	0	0	0	0	0
no	no	Stay at home, no other partners	0	no	0	0	0	0	0	0	0	0
no	no	Stay away, no other partners	0	no	0	0	0	0	0	0	0	0
yes	no	Stay away, no other partners	1	yes	0	0	1	1	0	0	0	0
no	no	Stay at home, no other partners	0	no	0	0	0	0	0	0	0	0

yes		no current partner	0	no	0	0	0	0	0	0	0	0
no	no	Stay at home, no other partners	0	no	0	0	0	0	0	0	0	0
no	no	Stay at home, has other partners	0	no	0	0	0	0	0	0	0	0
no	no	Stay at home, no other partners	0	no	0	0	0	0	0	0	0	0
no	no	Stay at home, no other partners	0	no	0	0	0	0	0	0	0	0
no	no	Stay at home, no other partners	0	no	0	0	0	0	0	0	0	0
no	yes	Stay at home, no other partners	1	yes	1	1	1	0	0	0	0	1
no	no	Stay away, no other partners	0	no	0	0	0	0	0	0	0	0
no	yes	Stay at home, no other partners	0	no	0	0	0	0	0	0	0	0
no	no	Stay at home, no other partners	0	no	0	0	0	0	0	0	0	0
no	no	Stay at home, no other partners	0	no	0	0	0	0	0	0	0	0
no	yes	Stay away, no other partners	0	no	0	0	0	0	0	0	0	0
yes		no current partner	1	yes	1	1	1	0	0	0	1	0
no	yes	Stay away, no other partners	0	no	0	0	0	0	0	0	0	0
			0	no	0	0	0	0	0	0	0	0
no	yes	Stay away, no other partners	0	no	0	0	0	0	0	0	0	0
yes	yes	Stay away, no other partners	0	no	0	0	0	0	0	0	0	0
no		Stay at home, no other partners	0	no	0	0	0	0	0	0	0	0
no	no	Stay at home, no other partners	0	no	0	0	0	0	0	0	0	0
no	no	Stay away, no other partners	0	no	0	0	0	0	0	0	0	0
no	yes	Stay away, has other partners	0	no	0	0	0	0	0	0	0	0
no	no	Stay away, has other partners	0	no	0	0	0	0	0	0	0	0
no	no	Stay away, no other partners	0	no	0	0	0	0	0	0	0	0
no	yes	Stay at home, no other partners	0	no	0	0	0	0	0	0	0	0
no	yes	Stay at home, no other partners	0	no	0	0	0	0	0	0	0	0
no	yes	Stay away, no other partners	0	no	0	0	0	0	0	0	0	0
no	yes	Stay at home, no other partners	0	no	0	0	0	0	0	0	0	0
no	yes	Stay at home, no other partners	0	no	0	0	0	0	0	0	0	0
no	yes	Stay at home, no other partners	0	no	0	0	0	0	0	0	0	0
no	no	Stay at home, no other partners	0	no	0	0	0	0	0	0	0	0
no	yes	Stay at home, no other partners	0	no	0	0	0	0	0	0	0	0
no		no current partner	0	no	0	0	0	0	0	0	0	0
no	yes	Stay at home, no other partners	0	no	0	0	0	0	0	0	0	0
no	no	Stay away, has other partners	0	no	0	0	0	0	0	0	0	0
no	yes	Stay at home, no other partners	0	no	0	0	0	0	0	0	0	0
no	yes	Stay at home, no other partners	0	no	0	0	0	0	0	0	0	0
no	no	Stay at home, no other partners	0	no	0	0	0	0	0	0	0	0
no		no current partner	0	no	0	0	0	0	0	0	0	0
yes	yes	Stay away, no other partners	0	no	0	0	0	0	0	0	0	0
no	yes	Stay at home, no other partners	0	no	0	0	0	0	0	0	0	0

no	yes	Stay away, has other partners	0	no	0	0	0	0	0	0	0	0
no	yes	Stay at home, no other partners	0	no	0	0	0	0	0	0	0	0
no	yes	Stay at home, no other partners	0	no	0	0	0	0	0	0	0	0
no	yes	Stay at home, no other partners	0	no	0	0	0	0	0	0	0	0
no	yes	Stay at home, no other partners	0	no	0	0	0	0	0	0	0	0
no	no	Stay away, no other partners	0	no	0	0	0	0	0	0	0	0
no		no current partner	0	no	0	0	0	0	0	0	0	0
no	yes	Stay at home, no other partners	0	no	0	0	0	0	0	0	0	0
no	yes	Stay away, no other partners	0	no	0	0	0	0	0	0	0	0
no	yes	Stay at home, no other partners	0	no	0	0	0	0	0	0	0	0
no	yes	Stay at home, has other partners	0	no	0	0	0	0	0	0	0	0
no	yes	Stay at home, no other partners	0	no	0	0	0	0	0	0	0	0
no	yes	Stay at home, no other partners	0	no	0	0	0	0	0	0	0	0
no	yes	Stay at home, no other partners	0	no	0	0	0	0	0	0	0	0
no	no	Stay at home, no other partners	0	no	0	0	0	0	0	0	0	0
no		no current partner	0	no	0	0	0	0	0	0	0	0
no	yes	Stay at home, no other partners	0	no	0	0	0	0	0	0	0	0
no	yes	Stay away, no other partners	0	no	0	0	0	0	0	0	0	0
yes		no current partner	1	yes	0	0	1	1	0	0	0	0
no	yes	Stay at home, no other partners	0	no	0	0	0	0	0	0	0	0
no	yes	Stay at home, no other partners	0	no	0	0	0	0	0	0	0	0
no	yes	Stay at home, no other partners	0	no	0	0	0	0	0	0	0	0
no	yes	Stay at home, no other partners	0	no	0	0	0	0	0	0	0	0
no	yes	Stay at home, no other partners	0	no	0	0	0	0	0	0	0	0
no	yes	Stay at home, no other partners	0	no	0	0	0	0	0	0	0	0
no		no current partner	1	yes	0	0	1	1	0	0	0	0
no	yes	Stay at home, no other partners	1	yes	1	1	1	0	0	0	0	1
no	yes	Stay at home, no other partners	0	no	0	0	0	0	0	0	0	0
no	yes	Stay at home, no other partners	0	no	0	0	0	0	0	0	0	0
no	no	Stay at home, no other partners	0	no	0	0	0	0	0	0	0	0
no	yes	Stay at home, no other partners	0	no	0	0	0	0	0	0	0	0
no	yes	Stay at home, has other partners	0	no	0	0	0	0	0	0	0	0
no	yes	Stay at home, has other partners	0	no	0	0	0	0	0	0	0	0
yes	no	Stay at home, no other partners	1	yes	0	0	0	0	0	0	0	0
no	no	Stay away, has other partners	0	no	0	0	0	0	0	0	0	0
no	no	Stay at home, no other partners	0	no	0	0	0	0	0	0	0	0
no		no current partner	0	no	0	0	0	0	0	0	0	0
no	no	Stay away, no other partners	0	no	0	0	0	0	0	0	0	0
no	yes	Stay at home, no other partners	0	no	0	0	0	0	0	0	0	0
no	yes	Stay at home, no other partners	0	no	0	0	0	0	0	0	0	0

no	yes	Stay at home, no other partners	1	yes	0	0	0	0	0	0	0	0
no	yes	Stay away, has other partners	0	no	0	0	0	0	0	0	0	0
no	yes	Stay at home, no other partners	0	no	0	0	0	0	0	0	0	0
no	yes	Stay at home, no other partners	0	no	0	0	0	0	0	0	0	0
no	yes	Stay at home, no other partners	0	no	0	0	0	0	0	0	0	0
no	no	Stay at home, no other partners	1	yes	0	0	1	1	0	0	0	0
no	yes	Stay away, no other partners	1	yes	1	1	1	0	0	0	0	1
no	no	Stay at home, no other partners	0	no	0	0	0	0	0	0	0	0
no	no	Stay away, no other partners	0	no	0	0	0	0	0	0	0	0
no	no	Stay at home, has other partners	0	no	0	0	0	0	0	0	0	0
no	yes	Stay at home, no other partners	0	no	0	0	0	0	0	0	0	0
no	yes	Stay at home, no other partners	0	no	0	0	0	0	0	0	0	0
no	yes	Stay away, has other partners	0	no	0	0	0	0	0	0	0	0
no	yes	Stay at home, no other partners	0	no	0	0	0	0	0	0	0	0
no	no	Stay away, no other partners	0	no	0	0	0	0	0	0	0	0
no	yes	Stay at home, no other partners	0	no	0	0	0	0	0	0	0	0
no	yes	Stay at home, no other partners	0	no	0	0	0	0	0	0	0	0
no	yes	Stay away, no other partners	0	no	0	0	0	0	0	0	0	0
no	yes	Stay at home, no other partners	0	no	0	0	0	0	0	0	0	0
no	yes	Stay away, no other partners	0	no	0	0	0	0	0	0	0	0
no		Stay away, has other partners	0	no	0	0	0	0	0	0	0	0
no	no	Stay at home, no other partners	0	no	0	0	0	0	0	0	0	0
no	no	Stay at home, no other partners	0	no	0	0	0	0	0	0	0	0
no		no current partner	0	no	0	0	0	0	0	0	0	0
no	yes	Stay at home, has other partners	0	no	0	0	0	0	0	0	0	0
no	no	Stay away, no other partners	1	yes	0	0	1	1	0	0	0	0
no	yes	Stay at home, no other partners	0	no	0	0	0	0	0	0	0	0
no	yes	Stay at home, no other partners	0	no	0	0	0	0	0	0	0	0
no	yes	Stay away, no other partners	0	no	0	0	0	0	0	0	0	0
yes	yes	Stay at home, no other partners	0	no	0	0	0	0	0	0	0	0
no	yes	Stay at home, no other partners	0	no	0	0	0	0	0	0	0	0
no	no	Stay at home, no other partners	0	no	0	0	0	0	0	0	0	0
no	yes	Stay at home, no other partners	0	no	0	0	0	0	0	0	0	0
no	yes	Stay at home, no other partners	1	yes	0	0	0	0	0	0	0	0
no	yes	Stay away, has other partners	0	no	0	0	0	0	0	0	0	0
no	yes	Stay at home, no other partners	0	no	0	0	0	0	0	0	0	0
no	yes	Stay at home, no other partners	0	no	0	0	0	0	0	0	0	0
no	yes	Stay at home, no other partners	0	no	0	0	0	0	0	0	0	0
no	yes	Stay at home, has other partners	0	no	0	0	0	0	0	0	0	0
no	yes	Stay at home, no other partners	0	no	0	0	0	0	0	0	0	0

no	yes	Stay at home, no other partners	0	no	0	0	0	0	0	0	0	0
no	yes	Stay at home, no other partners	0	no	0	0	0	0	0	0	0	0
no	yes	Stay at home, no other partners	0	no	0	0	0	0	0	0	0	0
no	yes	Stay at home, no other partners	0	no	0	0	0	0	0	0	0	0
no	yes	Stay away, no other partners	0	no	0	0	0	0	0	0	0	0
no	no	Stay at home, no other partners	0	no	0	0	0	0	0	0	0	0
no	no	Stay at home, no other partners	0	no	0	0	0	0	0	0	0	0
no	no	Stay away, no other partners	0	no	0	0	0	0	0	0	0	0
no	no	Stay away, no other partners	0	no	0	0	0	0	0	0	0	0
no	no	Stay at home, no other partners	0	no	0	0	0	0	0	0	0	0
no		Stay at home, has other partners	0	no	0	0	0	0	0	0	0	0
no	no	Stay at home, no other partners	1	yes	0	0	0	0	0	0	0	0
no	yes	Stay away, has other partners	1	yes	0	0	0	0	0	0	0	0
no	no	Stay at home, no other partners	0	no	0	0	0	0	0	0	0	0
no	no	Stay at home, no other partners	0	no	0	0	0	0	0	0	0	0
no		Stay at home, no other partners	0	no	0	0	0	0	0	0	0	0
no		no current partner	0	no	0	0	0	0	0	0	0	0
			0	no	0	0	0	0	0	0	0	0
no	no	Stay at home, no other partners	0	no	0	0	0	0	0	0	0	0
no	no	Stay at home, no other partners	0	no	0	0	0	0	0	0	0	0
no	no	Stay at home, no other partners	0	no	0	0	0	0	0	0	0	0
yes	no	Stay away, has other partners	0	no	0	0	0	0	0	0	0	0
no	yes	Stay at home, no other partners	0	no	0	0	0	0	0	0	0	0
no	yes	Stay at home, no other partners	0	no	0	0	0	0	0	0	0	0
yes	no	Stay away, no other partners	0	no	0	0	0	0	0	0	0	0
no	no	Stay at home, no other partners	0	no	0	0	0	0	0	0	0	0
			0	no	0	0	0	0	0	0	0	0
			0	no	0	0	0	0	0	0	0	0
no	yes	Stay away, no other partners	0	no	0	0	0	0	0	0	0	0
no	no	Stay at home, no other partners	0	no	0	0	0	0	0	0	0	0
no	no	Stay away, no other partners	0	no	0	0	0	0	0	0	0	0
no	yes	Stay away, has other partners	0	no	0	0	0	0	0	0	0	0
no	yes	Stay at home, no other partners	0	no	0	0	0	0	0	0	0	0
no	no	Stay at home, no other partners	0	no	0	0	0	0	0	0	0	0
no	yes	Stay at home, no other partners	0	no	0	0	0	0	0	0	0	0
yes			0	no	0	0	0	0	0	0	0	0
yes	no	Stay at home, no other partners	1	yes	0	0	0	0	0	0	0	0
no	no	Stay away, has other partners	0	no	0	0	0	0	0	0	0	0
yes	no	Stay at home, has other partners	0	no	0	0	0	0	0	0	0	0
yes		no current partner	0	no	0	0	0	0	0	0	0	0

no	no	Stay away, no other partners	0	no	0	0	0	0	0	0	0	0
no	yes	Stay at home, no other partners	0	no	0	0	0	0	0	0	0	0
yes		no current partner	0	no	0	0	0	0	0	0	0	0
no		no current partner	0	no	0	0	0	0	0	0	0	0
yes	no	Stay at home, no other partners	0	no	0	0	0	0	0	0	0	0
no	yes	Stay at home, no other partners	0	no	0	0	0	0	0	0	0	0
			0	no	0	0	0	0	0	0	0	0
no		no current partner	0	no	0	0	0	0	0	0	0	0
yes	no	Stay away, no other partners	1	yes	0	0	0	0	0	0	0	0
yes	yes	Stay at home, no other partners	0	no	0	0	0	0	0	0	0	0
yes	no	Stay away, no other partners	0	no	0	0	0	0	0	0	0	0
yes	no	Stay away, no other partners	0	no	0	0	0	0	0	0	0	0
no		no current partner	0	no	0	0	0	0	0	0	0	0
yes	yes	Stay away, no other partners	0	no	0	0	0	0	0	0	0	0
no	no	Stay away, no other partners	0	no	0	0	0	0	0	0	0	0
no		no current partner	0	no	0	0	0	0	0	0	0	0
no	no	Stay away, no other partners	0	no	0	0	0	0	0	0	0	0
no	yes	Stay at home, no other partners	0	no	0	0	0	0	0	0	0	0
no	no	Stay away, no other partners	0	no	0	0	0	0	0	0	0	0
yes		no current partner	0	no	0	0	0	0	0	0	0	0
no		Stay away, no other partners	0	no	0	0	0	0	0	0	0	0
no	no	Stay away, has other partners	0	no	0	0	0	0	0	0	0	0
no		no current partner	0	no	0	0	0	0	0	0	0	0
no	yes	Stay away, no other partners	1	yes	0	0	0	0	0	0	0	0
no	yes	Stay at home, no other partners	0	no	0	0	0	0	0	0	0	0
yes	yes	Stay at home, has other partners	0	no	0	0	0	0	0	0	0	0
no	yes	Stay at home, no other partners	0	no	0	0	0	0	0	0	0	0
no	yes	Stay at home, no other partners	0	no	0	0	0	0	0	0	0	0
no	no	Stay at home, no other partners	1	yes	0	0	0	0	0	0	0	0
no	yes	Stay away, no other partners	0	no	0	0	0	0	0	0	0	0
no	yes	Stay at home, no other partners	0	no	0	0	0	0	0	0	0	0
yes	yes	Stay at home, no other partners	0	no	0	0	0	0	0	0	0	0
yes		no current partner	1	yes	0	0	0	0	0	0	0	0
			0	no	0	0	0	0	0	0	0	0
no		no current partner	0	no	0	0	0	0	0	0	0	0
no		no current partner	0	no	0	0	0	0	0	0	0	0
no	yes	Stay at home, no other partners	0	no	0	0	0	0	0	0	0	0
no	yes	Stay at home, no other partners	0	no	0	0	0	0	0	0	0	0
no	yes	Stay at home, no other partners	1	no	0	1	1	0	1	0	0	0
no	yes	Stay at home, no other partners	0	no	0	0	0	0	0	0	0	0

no	no	Stay away, no other partners	0	no	0	0	0	0	0	0	0	0
no	yes	Stay away, has other partners	0	no	0	0	0	0	0	0	0	0
no	yes	Stay at home, no other partners	0	no	0	0	0	0	0	0	0	0
yes	yes	Stay away, no other partners	1	yes	0	0	0	0	0	0	0	0
yes	yes	Stay away, no other partners	0	no	0	0	0	0	0	0	0	0
no	yes	Stay away, no other partners	0	no	0	0	0	0	0	0	0	0
no		Stay at home, no other partners	0	no	0	0	0	0	0	0	0	0
no	yes	Stay at home, no other partners	0	no	0	0	0	0	0	0	0	0
no	yes	Stay at home, no other partners	0	no	0	0	0	0	0	0	0	0
no	yes	Stay at home, no other partners	0	no	0	0	0	0	0	0	0	0
			0	no	0	0	0	0	0	0	0	0
no	no	Stay at home, no other partners	0	no	0	0	0	0	0	0	0	0
no	yes	Stay at home, no other partners	1	yes	0	0	1	1	0	0	0	0
no		no current partner	0	no	0	0	0	0	0	0	0	0
no	yes	Stay at home, no other partners	0	no	0	0	0	0	0	0	0	0
no	yes	Stay at home, no other partners	0	no	0	0	0	0	0	0	0	0
no		no current partner	0	no	0	0	0	0	0	0	0	0
no	yes	Stay at home, no other partners	0	no	0	0	0	0	0	0	0	0
no	no	Stay at home, no other partners	0	no	0	0	0	0	0	0	0	0
no	yes	Stay at home, no other partners	0	no	0	0	0	0	0	0	0	0
no	yes	Stay at home, has other partners	0	no	0	0	0	0	0	0	0	0
no	no	Stay at home, no other partners	0	no	0	0	0	0	0	0	0	0
no	yes	Stay away, has other partners	0	no	0	0	0	0	0	0	0	0
no	no	Stay at home, no other partners	0	no	0	0	0	0	0	0	0	0
no	yes	Stay away, no other partners	0	no	0	0	0	0	0	0	0	0
no	yes	Stay at home, no other partners	0	no	0	0	0	0	0	0	0	0
no	yes	Stay away, no other partners	0	no	0	0	0	0	0	0	0	0
no	yes	Stay at home, no other partners	0	no	0	0	0	0	0	0	0	0
no	no	Stay at home, no other partners	0	no	0	0	0	0	0	0	0	0
no	yes	Stay at home, no other partners	0	no	0	0	0	0	0	0	0	0
no	no	Stay at home, no other partners	0	no	0	0	0	0	0	0	0	0
no	yes	Stay at home, no other partners	0	no	0	0	0	0	0	0	0	0
no	no	Stay away, no other partners	0	no	0	0	0	0	0	0	0	0
no	yes	Stay at home, no other partners	0	no	0	0	0	0	0	0	0	0
no	yes	Stay at home, no other partners	0	no	0	0	0	0	0	0	0	0
no	yes	Stay at home, no other partners	0	no	0	0	0	0	0	0	0	0
no	yes	Stay at home, no other partners	0	no	0	0	0	0	0	0	0	0
no	no	Stay at home, no other partners	0	no	0	0	0	0	0	0	0	0
no	yes	Stay at home, no other partners	0	no	0	0	0	0	0	0	0	0
no	yes	Stay at home, no other partners	0	no	0	0	0	0	0	0	0	0

no	no	Stay at home, has other partners	0	no	0	0	0	0	0	0	0	0
no	no	Stay at home, no other partners	0	no	0	0	0	0	0	0	0	0
no	yes	Stay away, no other partners	0	no	0	0	0	0	0	0	0	0
no		no current partner	0	no	0	0	0	0	0	0	0	0
no	yes	Stay at home, no other partners	1	yes	0	0	0	0	0	0	0	0
no	yes	Stay away, no other partners	0	no	0	0	0	0	0	0	0	0
no	no	Stay at home, no other partners	1	yes	0	0	1	1	0	0	0	0
no	no	Stay at home, no other partners	0	no	0	0	0	0	0	0	0	0
no		Stay at home, no other partners	0	no	0	0	0	0	0	0	0	0
yes		Stay at home, no other partners	0	no	0	0	0	0	0	0	0	0
no	yes	Stay away, no other partners	0	no	0	0	0	0	0	0	0	0
no		no current partner	1	yes	1	1	1	1	0	0	0	1
no	yes	Stay away, no other partners	0	no	0	0	0	0	0	0	0	0
no	yes	Stay at home, no other partners	0	no	0	0	0	0	0	0	0	0
no	no	Stay at home, has other partners	0	no	0	0	0	0	0	0	0	0
no	yes	Stay at home, has other partners	1	yes	1	1	1	0	0	0	0	1
no	no	Stay at home, no other partners	0	no	0	0	0	0	0	0	0	0
no	no	Stay at home, no other partners	0	no	0	0	0	0	0	0	0	0
no	yes	Stay at home, no other partners	0	no	0	0	0	0	0	0	0	0
no	yes	Stay at home, has other partners	0	no	0	0	0	0	0	0	0	0
no	yes	Stay at home, no other partners	0	no	0	0	0	0	0	0	0	0
no	yes	Stay at home, no other partners	0	no	0	0	0	0	0	0	0	0
no	no	Stay away, no other partners	0	no	0	0	0	0	0	0	0	0
no	yes	Stay at home, no other partners	0	no	0	0	0	0	0	0	0	0
			1	yes	0	0	0	0	0	0	0	0
no	yes	Stay at home, no other partners	0	no	0	0	0	0	0	0	0	0
no	yes	Stay at home, no other partners	0	no	0	0	0	0	0	0	0	0
no	yes	Stay at home, no other partners	0	no	0	0	0	0	0	0	0	0
no	yes	Stay at home, no other partners	0	no	0	0	0	0	0	0	0	0
no	yes	Stay away, no other partners	1	yes	0	0	1	1	0	0	0	0
no	yes	Stay at home, no other partners	0	no	0	0	0	0	0	0	0	0
no	yes	Stay at home, has other partners	0	no	0	0	0	0	0	0	0	0
no		no current partner	0	no	0	0	0	0	0	0	0	0
no	yes	Stay at home, no other partners	0	no	0	0	0	0	0	0	0	0
no	yes	Stay at home, no other partners	0	no	0	0	0	0	0	0	0	0
no	yes	Stay away, no other partners	0	no	0	0	0	0	0	0	0	0
no	yes	Stay away, no other partners	1	yes	0	0	0	0	0	0	0	0
no	yes	Stay at home, no other partners	0	no	0	0	0	0	0	0	0	0
no	yes	Stay away, no other partners	0	no	0	0	0	0	0	0	0	0
no	yes	Stay at home, no other partners	0	no	0	0	0	0	0	0	0	0

no	yes	Stay at home, no other partners	0	no	0	0	0	0	0	0	0	0
no	yes	Stay at home, no other partners	0	no	0	0	0	0	0	0	0	0
no		no current partner	0	no	0	0	0	0	0	0	0	0
no	yes	Stay at home, no other partners	0	no	0	0	0	0	0	0	0	0
no	no	Stay at home, no other partners	0	no	0	0	0	0	0	0	0	0
no	yes	Stay at home, no other partners	0	no	0	0	0	0	0	0	0	0
no	yes	Stay at home, has other partners	0	no	0	0	0	0	0	0	0	0
no	yes	Stay at home, no other partners	0	no	0	0	0	0	0	0	0	0
no	yes	Stay at home, no other partners	0	no	0	0	0	0	0	0	0	0
no	yes	Stay at home, no other partners	0	no	0	0	0	0	0	0	0	0
no	no	Stay at home, no other partners	0	no	0	0	0	0	0	0	0	0
no	yes	Stay at home, no other partners	0	no	0	0	0	0	0	0	0	0
no	yes	Stay at home, no other partners	0	no	0	0	0	0	0	0	0	0
no	no	Stay at home, has other partners	0	no	0	0	0	0	0	0	0	0
no	yes	Stay at home, no other partners	0	no	0	0	0	0	0	0	0	0
no	yes	Stay at home, no other partners	0	no	0	0	0	0	0	0	0	0
no	no	Stay at home, no other partners	0	no	0	0	0	0	0	0	0	0
no	yes	Stay at home, no other partners	0	no	0	0	0	0	0	0	0	0
no	yes	Stay at home, has other partners	0	no	0	0	0	0	0	0	0	0
no		no current partner	0	no	0	0	0	0	0	0	0	0
no	no	Stay at home, has other partners	1	yes	0	1	1	1	1	0	0	0
no	yes	Stay at home, no other partners	0	no	0	0	0	0	0	0	0	0
no	yes	Stay at home, no other partners	0	no	0	0	0	0	0	0	0	0
no	yes	Stay at home, has other partners	0	no	0	0	0	0	0	0	0	0
no	yes	Stay at home, has other partners	0	no	0	0	0	0	0	0	0	0
no	yes	Stay at home, no other partners	0	no	0	0	0	0	0	0	0	0
no	yes	Stay at home, no other partners	0	no	0	0	0	0	0	0	0	0
no	yes	Stay away, no other partners	1	yes	0	0	1	1	0	0	0	0
no	yes	Stay away, has other partners	0	no	0	0	0	0	0	0	0	0
no	yes	Stay at home, no other partners	0	no	0	0	0	0	0	0	0	0
no	yes	Stay at home, no other partners	0	no	0	0	0	0	0	0	0	0
no	no	Stay at home, has other partners	0	no	0	0	0	0	0	0	0	0
no	yes	Stay at home, no other partners	0	no	0	0	0	0	0	0	0	0
no	yes	Stay at home, no other partners	0	no	0	0	0	0	0	0	0	0
no	yes	Stay away, has other partners	0	no	0	0	0	0	0	0	0	0
no	yes	Stay at home, no other partners	0	no	0	0	0	0	0	0	0	0
no	yes	Stay at home, no other partners	0	no	0	0	0	0	0	0	0	0
no	yes	Stay away, no other partners	0	no	0	0	0	0	0	0	0	0
no	yes	Stay away, no other partners	0	no	0	0	0	0	0	0	0	0
no	no	Stay away, has other partners	0	no	0	0	0	0	0	0	0	0

no		no current partner	0	no	0	0	0	0	0	0	0	0
no	yes	Stay at home, no other partners	0	no	0	0	0	0	0	0	0	0
no	yes	Stay at home, no other partners	0	no	0	0	0	0	0	0	0	0
no	yes	Stay at home, no other partners	0	no	0	0	0	0	0	0	0	0
no	yes	Stay at home, no other partners	0	no	0	0	0	0	0	0	0	0
no	yes	Stay away, no other partners	0	no	0	0	0	0	0	0	0	0
no	yes	Stay at home, no other partners	0	no	0	0	0	0	0	0	0	0
no	yes	Stay at home, no other partners	0	no	0	0	0	0	0	0	0	0
no	no	Stay at home, no other partners	0	no	0	0	0	0	0	0	0	0
no	no	Stay at home, has other partners	0	no	0	0	0	0	0	0	0	0
no	no	Stay away, no other partners	0	no	0	0	0	0	0	0	0	0
no	yes	Stay away, has other partners	0	no	0	0	0	0	0	0	0	0
no	yes	Stay at home, no other partners	0	no	0	0	0	0	0	0	0	0
no	yes	Stay at home, no other partners	0	no	0	0	0	0	0	0	0	0
no	yes	Stay at home, no other partners	0	no	0	0	0	0	0	0	0	0
no	no	Stay at home, has other partners	0	no	0	0	0	0	0	0	0	0
no	yes	Stay at home, has other partners	0	no	0	0	0	0	0	0	0	0
no	yes	Stay at home, no other partners	0	no	0	0	0	0	0	0	0	0
no	no	Stay at home, no other partners	0	no	0	0	0	0	0	0	0	0
no	yes	Stay away, no other partners	0	no	0	0	0	0	0	0	0	0
no	yes	Stay at home, no other partners	0	no	0	0	0	0	0	0	0	0
no	yes	Stay at home, has other partners	0	no	0	0	0	0	0	0	0	0
no	no	Stay at home, no other partners	1	yes	0	0	0	0	0	0	0	0
no	yes	Stay at home, no other partners	0	no	0	0	0	0	0	0	0	0
no	yes	Stay away, no other partners	0	no	0	0	0	0	0	0	0	0
no	yes	Stay at home, no other partners	0	no	0	0	0	0	0	0	0	0
no	yes	Stay away, no other partners	0	no	0	0	0	0	0	0	0	0
no	yes	Stay at home, no other partners	0	no	0	0	0	0	0	0	0	0
no	no	Stay at home, no other partners	0	no	0	0	0	0	0	0	0	0
no	yes	Stay at home, no other partners	0	no	0	0	0	0	0	0	0	0
no	yes	Stay at home, no other partners	0	no	0	0	0	0	0	0	0	0
no	yes	Stay at home, no other partners	0	no	0	0	0	0	0	0	0	0
no	yes	Stay at home, no other partners	0	no	0	0	0	0	0	0	0	0
no	no	Stay at home, no other partners	0	no	0	0	0	0	0	0	0	0
no	yes	Stay at home, no other partners	0	no	0	0	0	0	0	0	0	0
yes	yes	Stay at home, no other partners	0	no	0	0	0	0	0	0	0	0
no	yes	Stay at home, no other partners	0	no	0	0	0	0	0	0	0	0
no	yes	Stay away, no other partners	0	no	0	0	0	0	0	0	0	0
no	yes	Stay at home, no other partners	0	no	0	0	0	0	0	0	0	0
no	yes	Stay at home, no other partners	0	no	0	0	0	0	0	0	0	0

no	yes	Stay away, no other partners	0	no	0	0	0	0	0	0	0	0
no	no	Stay at home, has other partners	1	yes	0	1	1	0	1	0	0	0
no	yes	Stay at home, has other partners	0	no	0	0	0	0	0	0	0	0
no	yes	Stay at home, no other partners	0	no	0	0	0	0	0	0	0	0
no	no	Stay at home, no other partners	0	no	0	0	0	0	0	0	0	0
no	yes	Stay away, no other partners	0	no	0	0	0	0	0	0	0	0
no	yes	Stay at home, no other partners	0	no	0	0	0	0	0	0	0	0
no	no	Stay at home, no other partners	0	no	0	0	0	0	0	0	0	0
no	yes	Stay at home, no other partners	0	no	0	0	0	0	0	0	0	0
no	yes	Stay at home, no other partners	0	no	0	0	0	0	0	0	0	0
no	yes	Stay at home, no other partners	0	no	0	0	0	0	0	0	0	0
no	yes	Stay at home, no other partners	0	no	0	0	0	0	0	0	0	0
no	yes	Stay at home, no other partners	0	no	0	0	0	0	0	0	0	0
no	yes	Stay at home, no other partners	0	no	0	0	0	0	0	0	0	0
no	yes	Stay at home, no other partners	0	no	0	0	0	0	0	0	0	0
yes	yes	Stay at home, has other partners	0	no	0	0	0	0	0	0	0	0
no	yes	Stay at home, no other partners	0	no	0	0	0	0	0	0	0	0
no	yes	Stay at home, no other partners	0	no	0	0	0	0	0	0	0	0
no	yes	Stay at home, no other partners	0	no	0	0	0	0	0	0	0	0
no	yes	Stay at home, no other partners	0	no	0	0	0	0	0	0	0	0
yes	yes	Stay at home, no other partners	0	no	0	0	0	0	0	0	0	0
no	yes	Stay at home, no other partners	0	no	0	0	0	0	0	0	0	0
no	yes	Stay at home, has other partners	0	no	0	0	0	0	0	0	0	0
no	no	Stay at home, has other partners	0	no	0	0	0	0	0	0	0	0
no	yes	Stay at home, no other partners	0	no	0	0	0	0	0	0	0	0
no	yes	Stay at home, no other partners	0	no	0	0	0	0	0	0	0	0
no	yes	Stay at home, no other partners	0	no	0	0	0	0	0	0	0	0
no	yes	Stay at home, no other partners	0	no	0	0	0	0	0	0	0	0
no		no current partner	0	no	0	0	0	0	0	0	0	0
no		no current partner	1	yes	0	0	0	0	0	0	0	0
no	yes	Stay at home, no other partners	0	no	0	0	0	0	0	0	0	0
no	no	Stay at home, no other partners	0	no	0	0	0	0	0	0	0	0
no	no	Stay at home, no other partners	0	no	0	0	0	0	0	0	0	0
no	yes	Stay at home, no other partners	0	no	0	0	0	0	0	0	0	0
no	yes	Stay at home, no other partners	0	no	0	0	0	0	0	0	0	0
no	yes	Stay at home, no other partners	0	no	0	0	0	0	0	0	0	0
no	yes	Stay at home, no other partners	0	no	0	0	0	0	0	0	0	0
no	yes	Stay at home, no other partners	0	no	0	0	0	0	0	0	0	0
no	yes	Stay at home, no other partners	0	no	0	0	0	0	0	0	0	0
no	yes	Stay at home, no other partners	0	no	0	0	0	0	0	0	0	0

no	yes	Stay at home, has other partners	0	no	0	0	0	0	0	0	0	0
no	yes	Stay at home, no other partners	0	no	0	0	0	0	0	0	0	0
no	yes	Stay at home, no other partners	0	no	0	0	0	0	0	0	0	0
no	yes	Stay at home, has other partners	0	no	0	0	0	0	0	0	0	0
no	yes	Stay at home, has other partners	0	no	0	0	0	0	0	0	0	0
no	yes	Stay at home, no other partners	0	no	0	0	0	0	0	0	0	0
no	yes	Stay at home, no other partners	0	no	0	0	0	0	0	0	0	0
no	no	Stay at home, no other partners	0	no	0	0	0	0	0	0	0	0
no	yes	Stay at home, no other partners	0	no	0	0	0	0	0	0	0	0
no	no	Stay at home, no other partners	0	no	0	0	0	0	0	0	0	0
no	yes	Stay at home, no other partners	0	no	0	0	0	0	0	0	0	0
no	yes	Stay at home, has other partners	0	no	0	0	0	0	0	0	0	0
no		no current partner	0	no	0	0	0	0	0	0	0	0
no	no	Stay at home, no other partners	0	no	0	0	0	0	0	0	0	0
no	yes	Stay at home, has other partners	0	no	0	0	0	0	0	0	0	0
no	yes	Stay at home, no other partners	0	no	0	0	0	0	0	0	0	0
no	yes	Stay at home, no other partners	0	no	0	0	0	0	0	0	0	0
no	yes	Stay at home, no other partners	0	no	0	0	0	0	0	0	0	0
no	no	Stay at home, no other partners	0	no	0	0	0	0	0	0	0	0
no	no	Stay at home, no other partners	0	no	0	0	0	0	0	0	0	0
no	yes	Stay at home, no other partners	0	no	0	0	0	0	0	0	0	0
no	yes	Stay at home, no other partners	0	no	0	0	0	0	0	0	0	0
no	no	Stay at home, no other partners	0	no	0	0	0	0	0	0	0	0
no	yes	Stay at home, has other partners	1	yes	0	0	0	0	0	0	0	0
no		no current partner	0	no	0	0	0	0	0	0	0	0
no	yes	Stay at home, no other partners	0	no	0	0	0	0	0	0	0	0
no	yes	Stay at home, no other partners	0	no	0	0	0	0	0	0	0	0
no	yes	Stay at home, no other partners	0	no	0	0	0	0	0	0	0	0
no	no	Stay at home, no other partners	0	no	0	0	0	0	0	0	0	0
no	yes	Stay at home, no other partners	0	no	0	0	0	0	0	0	0	0
no	yes	Stay at home, no other partners	0	no	0	0	0	0	0	0	0	0
no	yes	Stay at home, no other partners	0	no	0	0	0	0	0	0	0	0
no	no	Stay at home, no other partners	0	no	0	0	0	0	0	0	0	0
no	no	Stay away, no other partners	0	no	0	0	0	0	0	0	0	0
no	yes	Stay at home, no other partners	0	no	0	0	0	0	0	0	0	0
no	yes	Stay at home, no other partners	0	no	0	0	0	0	0	0	0	0
no	yes	Stay at home, no other partners	0	no	0	0	0	0	0	0	0	0
no	no	Stay at home, no other partners	0	no	0	0	0	0	0	0	0	0
no	yes	Stay at home, no other partners	0	no	0	0	0	0	0	0	0	0
no	yes	Stay at home, no other partners	0	no	0	0	0	0	0	0	0	0

no	yes	Stay at home, no other partners	0	no	0	0	0	0	0	0	0	0
no	yes	Stay at home, no other partners	0	no	0	0	0	0	0	0	0	0
no	yes	Stay away, no other partners	0	no	0	0	0	0	0	0	0	0
no	yes	Stay at home, no other partners	0	no	0	0	0	0	0	0	0	0
no	yes	Stay at home, has other partners	0	no	0	0	0	0	0	0	0	0
no	no	Stay at home, has other partners	0	no	0	0	0	0	0	0	0	0
no	yes	Stay at home, no other partners	0	no	0	0	0	0	0	0	0	0
no	yes	Stay at home, no other partners	1	yes	0	0	1	1	0	0	0	0
no	no	Stay at home, no other partners	0	no	0	0	0	0	0	0	0	0
no	yes	Stay at home, no other partners	0	no	0	0	0	0	0	0	0	0
no	yes	Stay at home, no other partners	0	no	0	0	0	0	0	0	0	0
no	no	Stay at home, no other partners	0	no	0	0	0	0	0	0	0	0
no	yes	Stay at home, no other partners	0	no	0	0	0	0	0	0	0	0
no	no	Stay at home, no other partners	0	no	0	0	0	0	0	0	0	0
no	no	Stay at home, no other partners	0	no	0	0	0	0	0	0	0	0
no	yes	Stay at home, no other partners	0	no	0	0	0	0	0	0	0	0
no	no	Stay at home, no other partners	0	no	0	0	0	0	0	0	0	0
no	yes	Stay at home, has other partners	0	no	0	0	0	0	0	0	0	0
no	yes	Stay at home, no other partners	0	no	0	0	0	0	0	0	0	0
no	yes	Stay at home, no other partners	0	no	0	0	0	0	0	0	0	0
no	yes	Stay at home, no other partners	0	no	0	0	0	0	0	0	0	0
no	yes	Stay at home, no other partners	0	no	0	0	0	0	0	0	0	0
no	yes	Stay at home, no other partners	0	no	0	0	0	0	0	0	0	0
no	yes	Stay at home, no other partners	0	no	0	0	0	0	0	0	0	0
no	yes	Stay at home, no other partners	0	no	0	0	0	0	0	0	0	0
no	yes	Stay at home, no other partners	0	no	0	0	0	0	0	0	0	0
no	yes	Stay at home, no other partners	0	no	0	0	0	0	0	0	0	0
no	yes	Stay at home, no other partners	0	no	0	0	0	0	0	0	0	0
no	yes	Stay at home, no other partners	0	no	0	0	0	0	0	0	0	0
no	yes	Stay at home, no other partners	0	no	0	0	0	0	0	0	0	0
no	no	Stay at home, no other partners	0	no	0	0	0	0	0	0	0	0
no	yes	Stay at home, no other partners	0	no	0	0	0	0	0	0	0	0
no	yes	Stay at home, no other partners	0	no	0	0	0	0	0	0	0	0
no		no current partner	0	no	0	0	0	0	0	0	0	0
no		no current partner	0	no	0	0	0	0	0	0	0	0
no	yes	Stay at home, no other partners	0	no	0	0	0	0	0	0	0	0
no	yes	Stay at home, no other partners	0	no	0	0	0	0	0	0	0	0
no	yes	Stay at home, no other partners	0	no	0	0	0	0	0	0	0	0
no	yes	Stay at home, has other partners	0	no	0	0	0	0	0	0	0	0
no	yes	Stay at home, no other partners	0	no	0	0	0	0	0	0	0	0

no	no	Stay at home, no other partners	0	no	0	0	0	0	0	0	0	0
no	no	Stay away, has other partners	0	no	0	0	0	0	0	0	0	0
no	yes	Stay at home, no other partners	0	no	0	0	0	0	0	0	0	0
no	yes	Stay at home, no other partners	0	no	0	0	0	0	0	0	0	0
no	no	Stay at home, has other partners	0	no	0	0	0	0	0	0	0	0
no	no	Stay away, has other partners	0	no	0	0	0	0	0	0	0	0
no		no current partner	0	no	0	0	0	0	0	0	0	0
no	yes	Stay away, no other partners	0	no	0	0	0	0	0	0	0	0
no	yes	Stay at home, no other partners	0	no	0	0	0	0	0	0	0	0
no	yes	Stay at home, no other partners	0	no	0	0	0	0	0	0	0	0
no	no	Stay at home, has other partners	0	no	0	0	0	0	0	0	0	0
no	yes	Stay at home, no other partners	0	no	0	0	0	0	0	0	0	0
no	yes	Stay at home, no other partners	0	no	0	0	0	0	0	0	0	0
no	yes	Stay at home, no other partners	0	no	0	0	0	0	0	0	0	0
no	no	Stay at home, no other partners	0	no	0	0	0	0	0	0	0	0
no	yes	Stay at home, no other partners	0	no	0	0	0	0	0	0	0	0
no	yes	Stay at home, no other partners	0	no	0	0	0	0	0	0	0	0
no	no	Stay at home, no other partners	0	no	0	0	0	0	0	0	0	0
no	yes	Stay at home, no other partners	0	no	0	0	0	0	0	0	0	0
no	no	Stay at home, no other partners	0	no	0	0	0	0	0	0	0	0
no	no	Stay at home, no other partners	0	no	0	0	0	0	0	0	0	0
no	no	Stay at home, no other partners	0	no	0	0	0	0	0	0	0	0
no	yes	Stay at home, no other partners	0	no	0	0	0	0	0	0	0	0
no	yes	Stay at home, no other partners	0	no	0	0	0	0	0	0	0	0
yes	yes	Stay at home, no other partners	0	no	0	0	0	0	0	0	0	0
no	yes	Stay at home, no other partners	0	no	0	0	0	0	0	0	0	0
no	yes	Stay at home, no other partners	0	no	0	0	0	0	0	0	0	0
no	yes	Stay at home, no other partners	0	no	0	0	0	0	0	0	0	0
no	yes	Stay at home, no other partners	0	no	0	0	0	0	0	0	0	0
no	yes	Stay at home, no other partners	0	no	0	0	0	0	0	0	0	0
no	yes	Stay at home, no other partners	0	no	0	0	0	0	0	0	0	0
no	yes	Stay at home, no other partners	0	no	0	0	0	0	0	0	0	0
no	yes	Stay at home, no other partners	0	no	0	0	0	0	0	0	0	0
no	yes	Stay at home, no other partners	0	no	0	0	0	0	0	0	0	0
no		Stay at home, no other partners	0	no	0	0	0	0	0	0	0	0
no	yes	Stay at home, no other partners	0	no	0	0	0	0	0	0	0	0
no	yes	Stay at home, no other partners	1	yes	0	0	0	0	0	0	0	0
no	yes	Stay at home, no other partners	0	no	0	0	0	0	0	0	0	0
no	yes	Stay at home, no other partners	0	no	0	0	0	0	0	0	0	0
no	yes	Stay at home, no other partners	0	no	0	0	0	0	0	0	0	0

no	yes	Stay at home, no other partners	0	no	0	0	0	0	0	0	0	0
no	yes	Stay at home, no other partners	0	no	0	0	0	0	0	0	0	0
no	yes	Stay at home, no other partners	0	no	0	0	0	0	0	0	0	0
no	no	Stay at home, no other partners	1	yes	0	0	1	1	0	0	0	0
no	yes	Stay at home, no other partners	0	no	0	0	0	0	0	0	0	0
no	yes	Stay at home, no other partners	0	no	0	0	0	0	0	0	0	0
no	yes	Stay at home, no other partners	0	no	0	0	0	0	0	0	0	0
no		Stay at home, no other partners	0	no	0	0	0	0	0	0	0	0
no	yes	Stay at home, has other partners	0	no	0	0	0	0	0	0	0	0
no	yes	Stay at home, no other partners	0	no	0	0	0	0	0	0	0	0
no	yes	Stay at home, no other partners	1	yes	0	0	1	1	0	0	0	0
no	yes	Stay at home, no other partners	0	no	0	0	0	0	0	0	0	0
no	yes	Stay at home, no other partners	0	no	0	0	0	0	0	0	0	0
no	yes	Stay at home, no other partners	0	no	0	0	0	0	0	0	0	0
no	yes	Stay at home, no other partners	0	no	0	0	0	0	0	0	0	0
no	yes	Stay at home, no other partners	0	no	0	0	0	0	0	0	0	0
no	yes	Stay at home, no other partners	0	no	0	0	0	0	0	0	0	0
no	yes	Stay at home, no other partners	0	no	0	0	0	0	0	0	0	0
no	yes	Stay at home, has other partners	0	no	0	0	0	0	0	0	0	0
no	yes	Stay at home, no other partners	0	no	0	0	0	0	0	0	0	0
no	yes	Stay at home, no other partners	0	no	0	0	0	0	0	0	0	0
no	yes	Stay at home, no other partners	0	no	0	0	0	0	0	0	0	0
no	yes	Stay at home, no other partners	0	no	0	0	0	0	0	0	0	0
no		no current partner	0	no	0	0	0	0	0	0	0	0
no	yes	Stay at home, no other partners	0	no	0	0	0	0	0	0	0	0
no	yes	Stay at home, no other partners	0	no	0	0	0	0	0	0	0	0
no	yes	Stay at home, no other partners	0	no	0	0	0	0	0	0	0	0
no	yes	Stay at home, no other partners	1	yes	0	0	0	0	0	0	0	0
no	yes	Stay at home, no other partners	0	no	0	0	0	0	0	0	0	0
no	no	Stay at home, no other partners	0	no	0	0	0	0	0	0	0	0
no	yes	Stay at home, no other partners	0	no	0	0	0	0	0	0	0	0
no	yes	Stay at home, no other partners	0	no	0	0	0	0	0	0	0	0
no	yes	Stay at home, no other partners	0	no	0	0	0	0	0	0	0	0
no	yes	Stay at home, no other partners	0	no	0	0	0	0	0	0	0	0
no	yes	Stay at home, no other partners	0	no	0	0	0	0	0	0	0	0
no	yes	Stay at home, no other partners	0	no	0	0	0	0	0	0	0	0
no	yes	Stay at home, no other partners	0	no	0	0	0	0	0	0	0	0
no	yes	Stay at home, no other partners	0	no	0	0	0	0	0	0	0	0
no	no	Stay at home, no other partners	0	no	0	0	0	0	0	0	0	0
yes	yes	Stay away, no other partners	0	no	0	0	0	0	0	0	0	0

no	yes	Stay away, no other partners	0	no	0	0	0	0	0	0	0	0
no	yes	Stay at home, no other partners	0	no	0	0	0	0	0	0	0	0
no	no	Stay at home, no other partners	0	no	0	0	0	0	0	0	0	0
no	yes	Stay at home, no other partners	0	no	0	0	0	0	0	0	0	0
no	yes	Stay at home, has other partners	0	no	0	0	0	0	0	0	0	0
no	yes	Stay away, no other partners	0	no	0	0	0	0	0	0	0	0
no	yes	Stay at home, has other partners	0	no	0	0	0	0	0	0	0	0
no	yes	Stay at home, no other partners	0	no	0	0	0	0	0	0	0	0
yes	no	Stay away, has other partners	1	yes	0	0	1	1	0	0	0	0
no		Stay at home, no other partners	0	no	0	0	0	0	0	0	0	0
no	yes	Stay at home, no other partners	0	no	0	0	0	0	0	0	0	0
no		no current partner	0	no	0	0	0	0	0	0	0	0
no	yes	Stay at home, no other partners	0	no	0	0	0	0	0	0	0	0
no	yes	Stay away, no other partners	0	no	0	0	0	0	0	0	0	0
no	no	Stay at home, no other partners	0	no	0	0	0	0	0	0	0	0
no	no	Stay at home, no other partners	0	no	0	0	0	0	0	0	0	0
no		Stay at home, no other partners	0	no	0	0	0	0	0	0	0	0
no	yes	Stay at home, no other partners	0	no	0	0	0	0	0	0	0	0
no	yes	Stay at home, no other partners	0	no	0	0	0	0	0	0	0	0
no	yes	Stay at home, no other partners	0	no	0	0	0	0	0	0	0	0
no		no current partner	0	no	0	0	0	0	0	0	0	0
no	yes	Stay away, no other partners	0	no	0	0	0	0	0	0	0	0
no	yes	Stay at home, no other partners	0	no	0	0	0	0	0	0	0	0
no	no	Stay at home, no other partners	0	no	0	0	0	0	0	0	0	0
no	yes	Stay away, has other partners	0	no	0	0	0	0	0	0	0	0
yes		no current partner	0	no	0	0	0	0	0	0	0	0
no	yes	Stay at home, no other partners	0	no	0	0	0	0	0	0	0	0
no	yes	Stay at home, no other partners	0	no	0	0	0	0	0	0	0	0
no	yes	Stay at home, no other partners	0	no	0	0	0	0	0	0	0	0
no	yes	Stay at home, no other partners	0	no	0	0	0	0	0	0	0	0
no	yes	Stay away, no other partners	0	no	0	0	0	0	0	0	0	0
no	yes	Stay at home, no other partners	0	no	0	0	0	0	0	0	0	0
no	yes	Stay at home, no other partners	0	no	0	0	0	0	0	0	0	0
no	yes	Stay away, no other partners	0	no	0	0	0	0	0	0	0	0
no	yes	Stay at home, no other partners	0	no	0	0	0	0	0	0	0	0
no	yes	Stay at home, no other partners	0	no	0	0	0	0	0	0	0	0
no	yes	Stay at home, no other partners	0	no	0	0	0	0	0	0	0	0
no	yes	Stay at home, no other partners	0	no	0	0	0	0	0	0	0	0
no	yes	Stay at home, no other partners	0	no	0	0	0	0	0	0	0	0
no	no	Stay at home, no other partners	0	no	0	0	0	0	0	0	0	0

no	yes	Stay at home, has other partners	0	no	0	0	0	0	0	0	0	0
no	yes	Stay at home, no other partners	0	no	0	0	0	0	0	0	0	0
no	yes	Stay at home, no other partners	0	no	0	0	0	0	0	0	0	0
no	yes	Stay at home, no other partners	0	no	0	0	0	0	0	0	0	0
no	yes	Stay at home, no other partners	0	no	0	0	0	0	0	0	0	0
no	yes	Stay at home, no other partners	1	yes	1	1	1	0	0	0	1	0
no	yes	Stay at home, no other partners	0	no	0	0	0	0	0	0	0	0
no	yes	Stay at home, no other partners	0	no	0	0	0	0	0	0	0	0
no	yes	Stay at home, no other partners	1	yes	0	0	0	0	0	0	0	0
no	yes	Stay at home, no other partners	0	no	0	0	0	0	0	0	0	0
no	yes	Stay at home, no other partners	0	no	0	0	0	0	0	0	0	0
no	yes	Stay away, no other partners	0	no	0	0	0	0	0	0	0	0
no	yes	Stay at home, no other partners	0	no	0	0	0	0	0	0	0	0
no	yes	Stay at home, no other partners	0	no	0	0	0	0	0	0	0	0
no	yes	Stay at home, no other partners	0	no	0	0	0	0	0	0	0	0
no	yes	Stay at home, no other partners	0	no	0	0	0	0	0	0	0	0
no	yes	Stay at home, no other partners	0	no	0	0	0	0	0	0	0	0
no		Stay at home, no other partners	0	no	0	0	0	0	0	0	0	0
no	yes	Stay at home, no other partners	0	no	0	0	0	0	0	0	0	0
yes	yes	Stay away, no other partners	0	no	0	0	0	0	0	0	0	0
no	yes	Stay at home, no other partners	0	no	0	0	0	0	0	0	0	0
no	yes	Stay at home, no other partners	0	no	0	0	0	0	0	0	0	0
no	yes	Stay at home, no other partners	0	no	0	0	0	0	0	0	0	0
no	yes	Stay at home, no other partners	0	no	0	0	0	0	0	0	0	0
no	yes	Stay at home, has other partners	0	no	0	0	0	0	0	0	0	0
no	yes	Stay at home, no other partners	0	no	0	0	0	0	0	0	0	0
no	no	Stay away, has other partners	1	yes	0	0	1	1	0	0	0	0
no	yes	Stay at home, no other partners	1	yes	0	0	1	1	0	0	0	0
no	yes	Stay at home, no other partners	0	no	0	0	0	0	0	0	0	0
no	yes	Stay at home, no other partners	0	no	0	0	0	0	0	0	0	0
no		no current partner	0	no	0	0	0	0	0	0	0	0
no	yes	Stay at home, no other partners	0	no	0	0	0	0	0	0	0	0
no		no current partner	0	no	0	0	0	0	0	0	0	0
no	no	Stay at home, no other partners	0	no	0	0	0	0	0	0	0	0
no	yes	Stay at home, no other partners	0	no	0	0	0	0	0	0	0	0
no	yes	Stay away, no other partners	0	no	0	0	0	0	0	0	0	0
no		Stay at home, no other partners	0	no	0	0	0	0	0	0	0	0
no	yes	Stay at home, has other partners	0	no	0	0	0	0	0	0	0	0
no	yes	Stay at home, no other partners	0	no	0	0	0	0	0	0	0	0
no	yes	Stay at home, has other partners	0	no	0	0	0	0	0	0	0	0

no	no	Stay at home, no other partners	0	no	0	0	0	0	0	0	0	0
no	yes	Stay at home, no other partners	0	no	0	0	0	0	0	0	0	0
no	no	Stay at home, no other partners	0	no	0	0	0	0	0	0	0	0
no	yes	Stay at home, no other partners	0	no	0	0	0	0	0	0	0	0
no	yes	Stay at home, no other partners	1	yes	0	0	1	1	0	0	0	0
no		Stay at home, no other partners	0	no	0	0	0	0	0	0	0	0
no	yes	Stay at home, no other partners	0	no	0	0	0	0	0	0	0	0
no	no	Stay at home, no other partners	0	no	0	0	0	0	0	0	0	0
no	yes	Stay at home, no other partners	1	yes	0	0	0	0	0	0	0	0
no	yes	Stay at home, no other partners	0	no	0	0	0	0	0	0	0	0
no	yes	Stay at home, no other partners	0	no	0	0	0	0	0	0	0	0
no	yes	Stay at home, no other partners	0	no	0	0	0	0	0	0	0	0
no	no	Stay at home, no other partners	0	no	0	0	0	0	0	0	0	0
no	yes	Stay at home, no other partners	0	no	0	0	0	0	0	0	0	0
no	yes	Stay at home, no other partners	0	no	0	0	0	0	0	0	0	0
no	no	Stay at home, no other partners	0	no	0	0	0	0	0	0	0	0
no	yes	Stay at home, no other partners	0	no	0	0	0	0	0	0	0	0
no	yes	Stay at home, no other partners	0	no	0	0	0	0	0	0	0	0
no	no	Stay at home, no other partners	0	no	0	0	0	0	0	0	0	0
no		Stay at home, no other partners	0	no	0	0	0	0	0	0	0	0
no	yes	Stay at home, no other partners	0	no	0	0	0	0	0	0	0	0
no	yes	Stay at home, no other partners	0	no	0	0	0	0	0	0	0	0
no	no	Stay away, no other partners	0	no	0	0	0	0	0	0	0	0
no	yes	Stay at home, no other partners	0	no	0	0	0	0	0	0	0	0
no	yes	Stay at home, no other partners	0	no	0	0	0	0	0	0	0	0
no	no	Stay at home, no other partners	0	no	0	0	0	0	0	0	0	0
no	no	Stay away, no other partners	1	yes	0	0	1	1	0	0	0	0
no	yes	Stay at home, no other partners	0	no	0	0	0	0	0	0	0	0
no	yes	Stay away, has other partners	1	yes	0	0	0	0	0	0	0	0
no	yes	Stay at home, no other partners	0	no	0	0	0	0	0	0	0	0
no	yes	Stay at home, has other partners	0	no	0	0	0	0	0	0	0	0
no	yes	Stay at home, no other partners	0	no	0	0	0	0	0	0	0	0
no	yes	Stay away, no other partners	0	no	0	0	0	0	0	0	0	0
no	yes	Stay at home, no other partners	0	no	0	0	0	0	0	0	0	0
no	yes	Stay at home, no other partners	0	no	0	0	0	0	0	0	0	0
no	no	Stay at home, no other partners	0	no	0	0	0	0	0	0	0	0
no	yes	Stay at home, no other partners	0	no	0	0	0	0	0	0	0	0
no	yes	Stay away, has other partners	1	yes	0	0	0	0	0	0	0	0
no	yes	Stay at home, no other partners	0	no	0	0	0	0	0	0	0	0
no	yes	Stay at home, has other partners	0	no	0	0	0	0	0	0	0	0

no	yes	Stay away, no other partners	0	no	0	0	0	0	0	0	0	0
no	yes	Stay at home, no other partners	0	no	0	0	0	0	0	0	0	0
no	yes	Stay at home, no other partners	0	no	0	0	0	0	0	0	0	0
no	yes	Stay at home, no other partners	0	no	0	0	0	0	0	0	0	0
no	yes	Stay away, has other partners	0	no	0	0	0	0	0	0	0	0
no	yes	Stay at home, no other partners	0	no	0	0	0	0	0	0	0	0
no	yes	Stay away, no other partners	0	no	0	0	0	0	0	0	0	0
no	no	Stay at home, no other partners	0	no	0	0	0	0	0	0	0	0
no		no current partner	0	no	0	0	0	0	0	0	0	0
no	no	Stay at home, no other partners	0	no	0	0	0	0	0	0	0	0
no	yes	Stay at home, no other partners	0	no	0	0	0	0	0	0	0	0
no	yes	Stay at home, no other partners	0	no	0	0	0	0	0	0	0	0
no	yes	Stay away, no other partners	0	no	0	0	0	0	0	0	0	0
no	yes	Stay at home, no other partners	0	no	0	0	0	0	0	0	0	0
no	yes	Stay at home, no other partners	0	no	0	0	0	0	0	0	0	0
no	yes	Stay away, no other partners	0	no	0	0	0	0	0	0	0	0
no	yes	Stay at home, no other partners	0	no	0	0	0	0	0	0	0	0
no	yes	Stay at home, no other partners	0	no	0	0	0	0	0	0	0	0
no	yes	Stay at home, has other partners	0	no	0	0	0	0	0	0	0	0
no		no current partner	0	no	0	0	0	0	0	0	0	0
no	yes	Stay at home, no other partners	0	no	0	0	0	0	0	0	0	0
no	yes	Stay at home, no other partners	0	no	0	0	0	0	0	0	0	0
no	yes	Stay away, has other partners	0	no	0	0	0	0	0	0	0	0
no	yes	Stay at home, no other partners	0	no	0	0	0	0	0	0	0	0
no	yes	Stay at home, has other partners	0	no	0	0	0	0	0	0	0	0
no	yes	Stay at home, no other partners	0	no	0	0	0	0	0	0	0	0
no	yes	Stay at home, no other partners	0	no	0	0	0	0	0	0	0	0
no	yes	Stay at home, no other partners	0	no	0	0	0	0	0	0	0	0
no	yes	Stay away, no other partners	0	no	0	0	0	0	0	0	0	0
no	yes	Stay at home, no other partners	0	no	0	0	0	0	0	0	0	0
no	yes	Stay at home, no other partners	1	yes	0	0	0	0	0	0	0	0
no		no current partner	0	no	0	0	0	0	0	0	0	0
no	yes	Stay at home, no other partners	0	no	0	0	0	0	0	0	0	0
no	no	Stay at home, no other partners	0	no	0	0	0	0	0	0	0	0
no	yes	Stay at home, no other partners	0	no	0	0	0	0	0	0	0	0
no	no	Stay at home, no other partners	0	no	0	0	0	0	0	0	0	0
no	yes	Stay at home, no other partners	0	no	0	0	0	0	0	0	0	0
no	yes	Stay away, has other partners	0	no	0	0	0	0	0	0	0	0
no	yes	Stay at home, no other partners	0	no	0	0	0	0	0	0	0	0
no	yes	Stay at home, no other partners	0	no	0	0	0	0	0	0	0	0

no	yes	Stay at home, no other partners	0	no	0	0	0	0	0	0	0	0
no	yes	Stay at home, no other partners	0	no	0	0	0	0	0	0	0	0
no	yes	Stay at home, no other partners	0	no	0	0	0	0	0	0	0	0
no	yes	Stay at home, no other partners	0	no	0	0	0	0	0	0	0	0
no	no	Stay at home, has other partners	0	no	0	0	0	0	0	0	0	0
no	no	Stay at home, no other partners	1	yes	0	0	1	1	0	0	0	0
no	yes	Stay at home, no other partners	0	no	0	0	0	0	0	0	0	0
no	yes	Stay at home, no other partners	0	no	0	0	0	0	0	0	0	0
no	yes	Stay at home, no other partners	0	no	0	0	0	0	0	0	0	0
no	yes	Stay at home, no other partners	0	no	0	0	0	0	0	0	0	0
no	yes	Stay at home, no other partners	0	no	0	0	0	0	0	0	0	0
no	yes	Stay at home, no other partners	1	yes	0	0	0	0	0	0	0	0
no	yes	Stay at home, no other partners	0	no	0	0	0	0	0	0	0	0
no	yes	Stay away, no other partners	0	no	0	0	0	0	0	0	0	0
no	yes	Stay at home, no other partners	0	no	0	0	0	0	0	0	0	0
no		no current partner	0	no	0	0	0	0	0	0	0	0
no	yes	Stay at home, no other partners	0	no	0	0	0	0	0	0	0	0
no	yes	Stay at home, no other partners	0	no	0	0	0	0	0	0	0	0
no	yes	Stay away, no other partners	0	no	0	0	0	0	0	0	0	0
no	yes	Stay away, no other partners	0	no	0	0	0	0	0	0	0	0
no	yes	Stay away, has other partners	0	no	0	0	0	0	0	0	0	0
no	yes	Stay at home, no other partners	0	no	0	0	0	0	0	0	0	0
no	yes	Stay at home, has other partners	0	no	0	0	0	0	0	0	0	0
no	yes	Stay at home, no other partners	0	no	0	0	0	0	0	0	0	0
no	no	Stay away, no other partners	0	no	0	0	0	0	0	0	0	0
no	yes	Stay at home, no other partners	0	no	0	0	0	0	0	0	0	0
no	yes	Stay away, no other partners	0	no	0	0	0	0	0	0	0	0
no	yes	Stay away, no other partners	0	no	0	0	0	0	0	0	0	0
no	no	Stay at home, no other partners	0	no	0	0	0	0	0	0	0	0
no	no	Stay at home, no other partners	0	no	0	0	0	0	0	0	0	0
no	yes	Stay away, no other partners	0	no	0	0	0	0	0	0	0	0
no	yes	Stay away, no other partners	0	no	0	0	0	0	0	0	0	0
no	yes	Stay away, no other partners	0	no	0	0	0	0	0	0	0	0
no	yes	Stay away, has other partners	0	no	0	0	0	0	0	0	0	0
no		no current partner	0	no	0	0	0	0	0	0	0	0
yes	yes	Stay away, no other partners	0	no	0	0	0	0	0	0	0	0
no	yes	Stay at home, no other partners	0	no	0	0	0	0	0	0	0	0
no	yes	Stay at home, no other partners	0	no	0	0	0	0	0	0	0	0
	yes	Stay at home, no other partners	0	no	0	0	0	0	0	0	0	0
no	no	Stay away, no other partners	0	no	0	0	0	0	0	0	0	0

no	yes	Stay away, no other partners	0	no	0	0	0	0	0	0	0	0
no	yes	Stay at home, no other partners	0	no	0	0	0	0	0	0	0	0
yes	no	Stay at home, no other partners	1	yes	0	0	0	0	0	0	0	0

HPV31	HPV33	HPV35	HPV39	HPV45	HPV51	HPV52	HPV56	HPV58	HPV59	HPV66	HPV68	non_1618
0	0	0	0	0	0	0	0	0	0	0	0	no
0	0	0	0	0	0	0	0	0	0	0	0	no
0	0	0	0	0	0	0	0	0	0	0	0	no
0	0	0	0	0	0	0	0	0	0	0	0	no
0	0	0	0	0	0	0	0	0	0	0	0	no
0	0	0	0	0	0	0	0	0	0	0	0	no
0	0	0	0	0	0	0	0	0	0	0	0	no
0	0	0	0	0	0	0	0	0	0	0	0	no
0	0	0	0	0	0	0	0	0	0	0	0	no
0	0	0	0	0	0	0	0	0	0	0	0	no
0	0	0	0	0	0	0	0	0	0	0	0	no
0	0	0	0	0	0	0	0	0	0	0	0	no
0	0	0	0	0	0	0	0	0	0	0	0	no
0	0	0	0	0	0	0	0	0	0	0	0	no
0	0	0	0	0	0	0	0	0	0	0	0	no
0	0	0	0	0	0	0	0	0	0	0	0	no
0	0	0	0	0	0	0	0	0	0	0	0	no
0	0	0	0	0	0	0	0	0	0	0	0	no
0	0	0	0	0	0	0	0	0	0	0	0	no
0	0	0	0	0	0	0	0	0	0	0	0	no
0	0	0	0	0	0	0	0	0	0	0	0	no
0	0	0	0	0	0	0	0	0	0	0	0	no
0	0	0	0	0	0	0	0	0	0	0	0	no
0	0	0	0	0	0	0	0	0	0	0	0	no
0	0	0	0	0	0	0	0	0	0	0	0	no
0	0	0	0	0	0	0	0	0	0	0	0	no
0	0	0	0	0	0	0	0	0	0	0	0	no
0	0	0	0	0	0	0	0	0	0	0	0	no
0	0	0	0	0	0	0	0	0	0	0	0	no
0	0	0	0	0	0	0	0	0	0	0	0	no
0	0	0	0	0	0	0	0	0	0	1	0	yes
0	0	0	0	0	0	0	0	0	0	0	0	no
0	0	0	0	0	0	0	0	0	0	0	0	no
0	0	0	0	0	0	0	0	0	0	0	0	no
0	0	0	0	0	0	0	0	0	0	0	0	no
0	0	0	0	0	0	0	0	0	0	0	0	no
0	0	0	0	0	0	0	0	0	0	0	0	no
0	0	0	0	0	0	0	0	0	0	0	0	no
0	0	0	0	0	0	0	0	0	0	0	0	no

0	0	0	0	0	0	0	0	0	0	0	0	no
0	0	0	0	0	0	0	0	0	0	0	0	no
0	0	0	0	0	0	0	0	0	0	0	0	no
0	0	0	0	0	0	0	0	0	0	0	0	no
0	0	0	0	0	0	0	0	0	0	0	0	no
0	0	0	0	0	0	0	0	0	0	0	0	no
0	0	0	0	0	0	0	0	0	0	0	0	no
0	0	0	0	0	0	0	0	0	0	0	0	no
0	0	0	0	0	0	0	0	0	0	0	0	no
0	0	0	0	0	0	0	0	0	0	0	0	no
0	0	0	0	0	0	0	0	0	0	0	0	no
0	0	0	0	0	0	0	0	0	0	0	0	no
0	0	0	0	0	0	0	0	0	0	0	0	no
0	0	0	0	0	0	0	0	0	0	0	0	no
0	0	0	0	0	0	0	0	0	0	0	0	no
0	0	0	0	0	0	0	0	0	0	0	0	no
0	0	0	0	0	0	0	0	0	0	0	0	no
0	0	0	0	0	0	0	0	0	0	0	0	no
0	0	0	0	0	0	0	0	0	0	0	0	no
0	0	0	0	0	0	0	0	0	0	0	0	no
0	0	0	0	0	0	0	0	0	0	0	0	no
0	0	0	0	0	0	0	0	0	0	0	0	no
0	0	0	0	0	0	0	0	0	0	0	0	no
0	0	0	0	0	0	0	0	0	0	0	0	no
0	0	0	0	0	0	0	0	0	0	0	0	no
0	0	0	0	0	0	0	0	0	0	0	0	no
0	0	0	0	0	0	0	0	0	0	0	0	no
0	0	0	0	0	0	0	0	0	0	0	0	no
0	0	0	0	0	0	0	0	0	0	0	0	no
0	0	0	0	0	0	0	0	0	0	0	0	no
0	0	0	0	0	0	0	0	0	0	0	0	no
0	0	0	0	0	0	0	0	0	0	0	0	no
0	0	0	0	0	0	0	0	0	0	0	0	no
0	0	0	0	0	0	0	0	0	0	0	0	no
0	0	0	0	0	0	0	0	0	0	0	0	no
0	0	0	0	0	0	0	0	0	0	0	0	no
0	0	0	0	0	0	0	0	0	0	0	0	no
0	0	0	0	0	0	0	0	0	0	0	0	no
0	0	0	0	0	0	0	0	0	0	0	0	no
0	0	0	0	0	0	0	0	0	0	0	0	no

0	0	0	0	0	0	0	0	0	0	0	0	no
0	0	0	0	0	0	0	0	0	0	0	0	no
0	0	0	0	0	0	0	0	0	0	0	0	no
0	0	0	0	0	0	0	0	0	0	0	0	no
0	0	0	0	0	0	0	0	0	0	0	0	no
0	0	0	0	0	1	0	0	0	1	0	0	yes
0	0	0	0	0	0	0	0	0	0	0	0	no
0	0	0	0	0	0	0	0	0	0	0	0	no
0	0	0	0	0	0	0	0	0	0	0	0	no
0	0	0	0	0	0	0	0	0	0	0	0	no
0	0	0	0	0	0	0	0	0	0	0	0	no
0	0	0	0	0	0	0	0	0	0	0	0	no
0	0	0	0	0	0	0	0	0	0	0	0	no
0	0	0	0	0	0	0	0	0	0	0	0	no
0	0	0	0	0	0	0	0	0	0	0	0	no
0	0	0	0	0	0	0	0	0	0	0	0	no
0	0	0	0	0	0	0	0	0	0	0	0	no
0	0	0	0	0	0	0	0	0	0	0	0	no
0	0	0	0	0	0	0	0	0	0	0	0	no
0	0	0	0	0	0	0	0	0	0	0	0	no
0	0	0	0	0	0	0	0	0	0	0	0	no
0	0	0	0	0	0	0	0	0	0	0	0	no
0	0	0	0	0	0	0	0	0	0	0	0	no
0	0	0	0	0	0	0	0	0	0	0	0	no
0	0	0	0	0	0	0	0	0	0	0	0	no
0	0	0	0	0	0	0	0	0	0	0	0	no
0	0	0	0	0	0	0	0	0	0	0	0	no
0	0	0	0	0	0	0	0	0	0	0	0	no
0	0	0	0	0	0	0	0	0	0	0	0	no
0	0	0	0	0	0	0	0	0	0	0	0	no
0	0	0	0	0	0	0	0	0	0	0	0	no
0	0	0	0	0	0	0	0	0	0	0	0	no
0	0	0	0	0	0	0	0	0	0	0	0	no
0	0	0	0	0	0	0	0	0	0	0	0	no
0	0	0	0	0	0	0	0	0	0	0	0	no
0	0	0	0	0	0	0	0	0	0	0	0	no
0	0	0	0	0	0	0	0	0	0	0	0	no
0	0	0	0	0	0	0	0	0	0	0	0	no
0	0	0	0	0	0	0	0	0	0	0	0	no
0	0	0	0	0	0	0	0	0	0	0	0	no

0	0	0	0	0	0	0	0	0	0	0	0	no
0	0	0	0	0	0	0	0	0	0	0	0	no
0	0	0	0	0	0	0	0	0	0	0	0	no
0	0	0	0	0	0	0	0	0	0	0	0	no
0	0	0	0	0	0	0	0	0	0	0	0	no
0	0	0	0	0	0	0	0	0	0	0	0	no
0	0	0	0	0	0	0	0	0	0	0	0	no
0	0	0	0	0	0	0	0	0	0	0	0	no
0	0	0	0	0	0	0	0	0	0	0	0	no
0	0	0	0	0	0	0	0	0	0	0	0	no
0	0	0	0	0	0	0	0	0	0	0	0	no
0	0	0	0	0	0	0	0	0	0	0	0	no
0	0	0	0	0	0	0	0	0	0	0	0	no
0	0	0	0	0	0	0	0	0	0	0	0	no
0	0	0	0	0	0	0	0	0	0	0	0	no
0	0	0	0	0	0	0	0	0	0	0	0	no
0	0	0	0	0	0	0	0	0	0	0	0	no
0	0	0	0	0	0	0	0	0	0	0	0	no
0	0	0	0	0	0	0	0	0	0	0	0	no
0	0	0	0	0	0	0	0	0	0	0	0	no
0	0	0	0	0	0	0	0	0	0	0	0	no
0	0	0	0	0	0	0	0	0	0	0	0	no
0	0	0	0	0	0	0	0	0	0	0	0	no
0	0	0	0	0	0	0	0	0	0	0	0	no
0	0	0	0	0	0	0	0	0	0	0	0	no
0	0	0	0	0	0	0	0	0	0	0	0	no
0	0	0	0	0	0	0	0	0	0	0	0	no
0	0	0	0	0	0	0	0	0	0	0	0	no
0	0	0	0	0	0	0	0	0	0	0	0	no
0	0	0	0	0	0	0	0	0	0	0	0	no
0	0	0	0	0	0	0	0	0	0	0	0	no
0	0	0	0	0	0	0	0	0	0	0	0	no
0	0	0	0	0	0	0	0	0	0	0	0	no
0	0	0	0	0	0	0	0	0	0	0	0	no
0	0	0	0	0	0	0	0	0	0	0	0	no
0	0	0	0	0	0	0	0	0	0	0	0	no
0	0	0	0	0	0	0	0	0	0	0	0	no
0	0	0	0	0	0	0	0	0	0	0	0	no
0	0	0	0	0	0	0	0	0	0	0	0	no
0	0	0	0	0	0	0	0	0	0	0	0	no

0	0	0	0	0	0	0	0	0	0	0	0	no
0	0	0	0	0	0	0	0	0	0	0	0	no
0	0	0	0	0	0	0	0	0	0	0	0	no
0	0	0	0	0	0	0	0	0	0	0	0	no
0	0	0	0	0	0	0	0	0	0	0	0	no
0	0	0	0	0	0	0	0	0	0	0	0	no
0	0	0	0	0	0	0	0	0	0	0	0	no
0	0	0	0	0	0	0	0	0	0	0	0	no
0	0	0	0	0	0	0	0	0	0	0	0	no
0	0	0	0	0	0	0	0	0	0	1	0	yes
0	0	0	0	0	0	0	0	0	0	0	0	no
0	0	0	0	0	0	0	0	0	0	0	0	no
0	0	0	0	0	0	0	0	0	0	0	0	no
0	0	0	0	0	0	0	0	0	0	0	0	no
0	0	0	0	0	0	0	0	0	0	0	0	no
0	0	0	0	0	0	0	0	0	0	0	0	no
0	0	0	0	0	0	0	0	0	0	0	0	no
0	0	0	0	0	0	0	0	0	0	0	0	no
0	0	0	0	0	0	0	0	0	0	0	0	no
0	0	0	0	0	0	0	0	0	0	0	0	no
0	0	0	0	0	0	0	0	0	0	0	0	no
0	0	0	0	0	0	0	0	0	0	0	0	no
0	0	0	0	0	0	0	0	0	0	0	0	no
0	0	0	0	0	0	0	0	0	0	0	1	yes
0	0	0	0	0	0	0	0	0	0	0	0	no
0	0	0	0	0	0	0	0	0	0	0	0	no
0	0	0	0	0	0	0	0	0	0	0	0	no
0	0	0	0	0	0	0	0	0	0	0	0	no
0	0	0	0	0	0	0	0	0	0	0	0	no
0	0	0	0	0	0	0	0	0	0	0	0	no
0	0	0	0	0	0	0	0	0	0	0	0	no
0	0	0	0	0	0	0	0	0	0	0	0	no
0	0	0	0	0	0	0	0	0	0	0	0	no
0	0	0	0	0	0	0	0	0	0	0	0	no
0	0	0	0	0	0	0	0	0	0	0	0	no
0	0	0	0	0	0	0	0	0	0	0	0	no
0	0	0	0	0	0	0	0	0	0	0	0	no
0	0	0	0	0	0	0	0	0	0	0	0	no
0	0	0	0	0	0	0	0	0	0	0	0	no
0	0	0	0	0	0	0	0	0	0	0	0	no

0	0	0	0	0	0	0	0	0	0	0	0	no
0	0	0	0	0	0	0	0	0	0	0	0	no
0	1	0	0	0	0	0	0	0	0	0	0	no
0	0	0	0	0	0	0	0	0	0	0	0	no
0	0	0	0	0	0	0	0	0	0	0	0	no
0	0	0	0	0	0	0	0	0	0	0	0	no
0	0	0	0	0	0	0	0	0	0	0	0	no
0	0	0	0	0	0	0	0	0	0	0	0	no
0	0	0	0	0	0	0	0	0	0	0	0	no
0	0	0	0	0	0	0	0	0	0	0	0	no
0	0	0	0	0	0	0	0	0	0	0	0	no
0	0	0	0	0	0	0	0	0	0	0	0	no
0	0	0	0	0	0	0	0	0	0	0	0	no
0	0	0	0	0	0	0	0	0	0	0	0	no
0	0	0	0	0	0	0	0	0	0	0	0	no
0	0	0	0	0	0	0	0	0	0	0	0	no
0	0	0	0	0	0	0	0	0	0	0	0	no
0	0	0	0	0	0	0	0	0	0	0	0	no
0	0	0	0	0	0	0	0	0	0	0	0	no
0	0	0	0	0	0	0	0	0	0	0	0	no
0	0	0	0	0	0	0	0	0	0	0	0	no
0	0	0	0	0	0	0	0	0	0	0	0	no
0	0	0	0	0	0	0	0	0	0	0	0	no
0	0	0	0	0	0	0	0	0	0	0	0	no
0	0	0	0	0	0	0	0	0	0	0	0	no
0	0	0	0	0	0	0	0	0	0	0	0	no
0	0	0	0	0	0	0	0	0	0	0	0	no
0	0	0	0	0	0	0	0	0	0	0	0	no
0	0	0	0	0	0	0	0	0	0	0	0	no
0	0	0	0	0	0	0	0	0	0	0	0	no
0	0	0	0	0	0	0	0	0	0	0	0	no
0	0	0	0	0	0	0	0	0	0	0	0	no
0	0	0	0	0	0	0	0	0	0	0	0	no
0	0	0	0	0	0	0	0	0	0	0	0	no
0	0	0	0	0	0	0	0	0	0	0	0	no
0	0	0	0	0	0	0	0	0	0	0	0	no
0	0	0	0	0	0	0	0	0	0	0	0	no
0	0	0	0	0	0	0	0	0	0	0	0	no
0	0	0	0	0	0	0	0	0	0	0	0	no
0	0	0	0	0	0	0	0	0	0	0	0	no

0	0	0	0	0	0	0	0	0	0	0	0	no
0	0	0	0	0	0	0	0	0	0	0	0	no
0	0	0	0	0	0	0	0	0	0	0	0	no
0	0	0	0	0	0	0	0	0	0	0	0	no
0	0	0	0	0	0	0	0	0	0	0	0	no
0	0	0	0	0	0	0	0	0	0	0	0	no
0	0	0	0	0	0	0	0	0	0	0	0	no
0	0	0	0	0	0	0	0	0	0	0	0	no
0	0	0	0	0	0	0	0	0	0	0	0	no
0	0	0	0	0	0	0	0	0	0	0	0	no
0	0	0	0	0	0	0	0	0	0	0	0	no
0	0	0	0	0	0	0	0	0	0	0	0	no
0	0	0	0	0	0	0	0	0	0	0	0	no
0	0	0	0	0	0	0	0	0	0	0	0	no
0	0	0	0	0	0	0	0	0	0	0	0	no
0	0	0	0	0	0	0	0	0	0	1	0	no
0	0	0	0	0	0	0	0	0	0	0	0	no
0	0	0	0	0	0	0	0	0	0	0	0	no
0	0	0	0	0	0	0	0	0	0	0	0	no
0	0	0	0	0	0	0	0	0	0	0	0	no
0	0	0	0	0	0	0	0	0	0	0	0	no
0	0	0	0	0	0	0	0	0	0	0	0	no
0	0	0	0	0	0	0	0	0	0	0	0	no
0	0	0	0	0	0	0	0	0	0	0	0	no
0	0	0	0	0	0	0	0	0	0	0	0	no
0	0	0	0	0	0	0	0	0	0	0	0	no
0	0	0	0	0	0	0	0	0	0	0	0	no
0	0	0	0	0	0	0	0	0	0	0	0	no
0	0	0	0	0	0	0	0	0	0	0	0	no
0	0	0	0	0	0	0	0	0	0	0	0	no
0	0	0	0	0	0	0	0	0	0	0	0	no
0	0	0	0	0	0	0	0	0	0	0	0	no
0	0	0	0	0	0	0	0	0	0	0	0	no
0	0	0	0	0	0	0	0	0	0	0	0	no
0	0	0	0	0	0	0	0	0	0	0	0	no
0	0	0	0	0	0	0	0	0	0	0	0	no
0	0	0	0	0	0	0	0	0	0	0	0	no
0	0	0	0	0	0	0	0	0	0	0	0	no
0	0	0	0	0	0	0	0	0	0	0	0	no
0	0	0	0	0	0	0	0	0	0	0	0	no

0	0	0	0	0	0	0	0	0	0	0	0	no
0	0	0	0	0	0	0	0	0	0	0	0	no
0	0	0	0	0	0	0	0	0	0	0	0	no
0	0	0	0	0	0	0	0	0	0	0	0	no
0	0	0	0	0	0	0	0	0	0	0	0	no
0	0	0	0	0	0	0	0	0	0	0	0	no
0	0	0	0	0	0	0	0	0	0	0	0	no
0	0	0	0	0	0	0	0	0	0	0	0	no
0	0	0	0	0	0	0	0	0	0	0	0	no
0	0	0	0	0	0	0	0	0	0	0	1	yes
0	0	0	0	0	0	0	0	0	0	0	0	no
0	0	0	0	0	0	0	0	0	0	0	0	no
0	0	0	0	0	0	0	0	0	0	0	0	no
0	0	0	0	0	0	0	0	0	0	0	0	no
0	0	0	0	0	0	0	0	0	0	0	0	no
0	0	0	0	0	0	0	0	0	0	0	0	no
0	0	0	0	0	0	0	0	0	0	0	0	no
0	0	0	0	0	0	0	0	0	0	0	0	no
0	0	0	0	0	0	0	0	0	0	0	0	no
0	0	0	0	0	0	0	0	0	0	0	0	no
0	0	0	0	0	0	0	0	0	0	0	0	no
0	0	0	0	0	0	0	0	0	0	0	0	no
0	0	0	0	0	0	0	0	0	0	0	0	no
0	0	0	0	0	0	0	0	0	0	0	0	no
0	0	0	0	0	0	0	0	0	0	0	0	no
0	0	0	0	0	0	0	0	0	0	0	0	no
0	0	0	0	0	1	0	0	0	0	0	0	yes
0	0	0	0	0	0	0	0	0	0	0	0	no
0	0	0	0	0	0	0	0	0	0	0	0	no
0	0	0	0	0	0	0	0	0	0	0	0	no
0	0	0	0	0	0	0	0	0	0	0	0	no
0	0	0	0	0	0	0	0	0	0	0	0	no
0	0	0	0	0	0	0	0	0	0	0	0	no
0	0	0	0	0	0	0	0	0	0	0	0	no
0	0	0	0	0	0	0	0	0	0	0	0	no
0	0	0	0	0	0	0	0	0	0	0	0	no
0	0	0	0	0	0	0	0	0	0	0	0	no
0	0	0	0	0	0	0	0	0	0	0	0	no
0	0	0	0	0	0	0	0	0	0	0	0	no
0	0	0	0	0	0	0	0	0	0	0	0	no

0	0	0	0	0	0	0	0	0	0	0	0	no
0	0	0	0	0	0	0	0	0	0	0	0	no
0	0	0	0	0	0	0	0	0	0	0	0	no
0	0	0	0	0	0	0	0	0	0	0	0	no
0	0	0	0	0	0	0	0	0	0	0	0	no
0	0	0	0	0	0	0	0	0	0	0	0	no
0	0	0	0	0	0	0	0	0	0	0	0	no
0	0	0	0	0	0	0	0	0	0	0	0	no
1	0	0	0	0	0	0	0	0	0	0	0	yes
0	0	0	0	0	0	0	0	0	0	0	0	no
0	0	0	0	0	0	0	0	0	0	0	0	no
0	0	0	0	0	0	0	0	0	0	0	0	no
0	0	0	0	0	0	0	0	0	0	0	0	no
0	0	0	0	0	0	0	0	0	0	0	0	no
0	0	0	0	0	0	0	0	0	0	0	0	no
0	0	0	0	0	0	0	0	0	0	0	0	no
0	0	0	0	0	0	0	0	0	0	0	0	no
0	0	0	0	0	0	0	0	0	0	0	0	no
0	0	0	0	0	0	0	0	0	0	0	0	no
1	0	0	0	0	0	0	0	0	0	0	0	yes
0	0	0	0	0	0	0	0	0	0	0	0	no
0	0	0	0	0	0	0	0	0	0	0	0	no
0	0	0	0	0	0	0	0	0	0	0	0	no
0	0	0	0	0	0	0	0	0	0	0	0	no
0	0	0	0	0	0	0	0	0	0	0	0	no
0	1	0	0	0	0	0	0	0	0	0	0	yes
0	0	0	0	1	0	0	0	0	0	0	0	yes
0	0	0	0	0	0	0	0	0	0	0	0	no
0	0	0	0	0	0	0	0	0	0	0	0	no
0	0	0	0	0	0	0	0	0	0	0	0	no
0	0	0	0	0	0	0	0	0	0	0	0	no
0	0	0	0	0	0	0	0	0	0	0	0	no
0	0	0	0	0	0	0	0	0	0	0	0	no
0	0	0	0	0	0	0	0	0	0	0	0	no
0	0	0	0	0	0	0	0	0	0	0	0	no
0	0	0	0	0	0	0	0	0	0	0	0	no
0	0	0	0	1	0	0	0	0	0	0	0	yes
0	0	0	0	0	1	0	0	0	0	0	0	yes
0	0	0	0	0	0	0	0	0	0	0	0	no
0	0	0	0	0	0	0	0	0	0	0	0	no

0	0	0	0	0	0	0	0	0	0	0	0	no
0	0	0	0	0	0	0	0	0	0	0	0	no
0	0	0	0	0	0	0	0	0	0	0	0	no
0	0	0	0	0	0	1	0	0	0	0	0	yes
0	0	0	0	0	0	0	0	0	0	0	0	no
0	0	0	0	0	0	0	0	0	0	0	0	no
0	0	0	0	0	0	0	0	0	0	0	0	no
0	0	0	0	0	0	0	0	0	0	0	0	no
0	0	0	0	0	0	0	0	0	0	0	0	no
0	0	0	0	0	0	1	0	0	0	0	0	yes
0	0	0	0	0	0	0	0	0	0	0	0	no
0	0	0	0	0	0	0	0	0	0	0	0	no
0	0	0	0	0	0	1	0	0	0	0	0	yes
0	0	0	0	0	0	0	0	0	0	0	0	no
0	0	0	0	0	0	0	0	0	0	0	0	no
0	0	0	0	0	0	0	0	0	0	0	0	no
0	0	0	0	0	0	0	0	0	0	0	1	yes
0	0	0	0	0	0	0	0	0	0	0	0	no
0	0	0	0	0	0	0	0	0	0	0	0	no
0	0	0	0	0	0	0	0	0	0	0	0	no
0	0	0	0	0	0	0	0	0	0	0	0	no
0	0	0	0	0	0	0	0	0	0	0	0	no
0	0	0	0	0	0	0	0	0	0	0	0	no
0	0	0	0	0	0	1	0	0	0	0	0	yes
0	0	0	0	0	0	0	0	0	0	0	0	no
0	0	0	0	0	0	0	0	0	0	0	0	no
0	0	0	0	0	0	1	0	0	0	0	0	yes
0	0	0	0	0	0	0	0	0	0	0	1	yes
0	0	0	0	0	0	1	0	0	0	0	0	yes
0	0	0	0	0	0	0	0	0	0	0	0	no
0	0	0	0	0	0	0	0	0	0	0	0	no
0	0	0	0	0	0	0	0	0	0	0	0	no
0	0	0	0	0	0	0	0	0	0	0	0	no
0	0	0	0	0	0	0	0	0	0	0	0	no
0	0	0	0	0	0	0	0	0	0	0	0	no
0	0	0	0	0	0	0	0	0	0	0	0	no
0	0	0	0	0	0	0	0	0	0	0	0	no
0	0	0	0	0	0	0	0	0	0	0	0	no
0	0	0	0	0	0	0	0	0	0	0	0	no
0	0	0	0	0	0	0	0	0	0	0	0	no

0	0	0	0	0	0	0	0	0	0	0	1	yes
0	0	0	0	0	0	0	0	0	0	0	0	no
0	0	0	0	0	0	0	0	0	0	0	0	no
0	0	0	0	0	0	0	0	0	0	0	0	no
0	0	0	0	0	0	1	0	0	0	0	0	yes
0	0	0	0	0	0	0	0	0	0	0	0	no
0	0	0	0	0	0	0	0	0	0	0	0	no
0	0	0	0	0	0	0	0	0	0	0	0	no
0	0	0	0	0	0	0	0	0	0	0	0	no
0	0	0	0	0	0	0	0	0	0	0	0	no
0	0	0	0	0	0	0	0	0	0	0	0	no
0	0	0	0	0	0	0	0	0	0	0	0	no
0	0	0	0	0	0	0	0	0	0	0	0	no
0	0	0	0	0	0	0	0	0	0	0	0	no
1	0	0	0	0	1	0	0	0	0	0	0	yes
0	0	0	0	0	0	0	0	0	0	0	0	no
0	0	0	0	0	0	0	0	0	0	0	0	no
0	0	0	0	0	0	0	0	0	0	0	0	no
0	0	0	0	0	0	0	0	0	0	0	0	no
0	0	0	0	0	0	0	0	0	0	0	0	no
0	0	0	0	0	0	0	0	0	0	0	0	no
0	0	0	0	0	0	0	0	0	0	0	0	no
0	0	0	0	0	0	0	0	0	0	0	0	no
0	0	0	0	0	0	0	0	0	0	0	0	no
0	0	0	0	0	0	0	0	0	0	0	0	no
0	0	0	0	0	0	0	0	0	0	0	0	no
0	0	0	0	0	0	0	0	0	0	0	0	no
0	0	0	0	0	0	0	0	0	0	0	0	no
0	0	0	0	0	0	0	0	0	0	0	0	no
0	0	0	0	0	0	0	0	0	0	0	0	no
0	0	0	0	0	0	0	0	0	0	0	0	no
0	0	0	0	0	0	0	0	0	0	0	0	no
0	0	0	0	0	0	0	0	0	0	0	0	no
0	0	0	0	0	0	0	0	0	0	0	0	no
0	0	0	0	0	0	0	0	0	0	0	0	no
0	0	0	0	0	0	0	0	0	0	0	0	no
0	0	0	0	0	0	0	0	0	0	0	0	no
0	0	0	0	0	0	0	0	0	0	0	0	no
0	0	0	0	0	0	0	0	0	0	0	0	no
0	0	0	0	0	0	0	0	0	0	0	0	no

0	0	0	0	0	0	0	0	0	0	0	0	no
0	0	0	0	0	0	0	0	0	0	0	0	no
0	0	0	0	0	0	0	0	0	0	0	0	no
0	0	0	0	0	0	0	0	0	0	0	0	no
0	0	0	0	0	0	0	0	0	0	0	0	no
0	0	0	0	0	0	0	0	0	0	0	0	no
0	0	0	0	0	0	0	0	0	0	0	0	no
0	0	0	0	0	0	0	0	0	0	0	0	no
0	0	0	0	0	0	0	0	0	0	0	0	no
0	0	0	0	0	0	0	0	0	0	0	0	no
0	0	0	0	0	0	0	0	0	0	0	0	no
0	0	0	0	0	0	0	0	0	0	0	0	no
0	0	0	0	0	0	0	0	0	0	0	0	no
0	0	0	0	0	0	0	0	0	0	0	0	no
0	0	0	0	0	0	0	0	0	0	0	0	no
0	0	0	0	0	0	0	0	0	0	0	0	no
0	0	0	0	0	0	0	0	0	0	0	0	no
0	0	0	0	0	0	0	0	0	0	0	0	no
0	0	0	0	0	0	0	0	0	0	0	0	no
0	0	0	0	0	0	0	0	0	0	0	0	no
0	0	0	0	0	0	0	0	0	0	0	0	no
0	0	0	0	0	0	0	0	0	0	0	0	no
0	0	0	0	0	0	0	0	0	0	0	0	no
0	0	0	0	0	0	0	0	0	0	0	0	no
0	0	0	0	0	0	0	0	0	0	0	0	no
0	0	0	0	0	0	0	0	0	0	0	0	no
0	0	0	0	0	0	0	0	0	0	0	0	no
0	0	0	0	0	0	0	0	0	0	0	0	no
0	0	0	0	0	0	0	0	0	0	0	0	no
0	0	0	0	0	0	0	0	0	0	0	0	no
0	0	0	0	0	0	0	0	0	0	0	0	no
0	0	0	0	0	0	0	0	0	0	0	0	no
0	0	0	0	0	0	0	0	0	0	0	0	no
0	0	0	0	0	0	0	0	0	0	0	0	no
0	0	0	0	0	0	0	0	0	0	0	0	no
0	0	0	0	0	0	0	0	0	0	0	0	no
0	0	0	0	0	0	0	0	0	0	0	0	no
0	0	0	0	0	0	0	0	0	0	0	0	no
0	0	0	0	0	0	0	0	0	0	0	0	no
0	0	0	0	0	0	0	0	0	0	0	0	no

0	0	0	0	0	0	0	0	0	0	0	0	no
0	0	0	0	0	0	0	0	0	0	0	0	no
0	0	0	0	0	0	0	0	0	0	0	0	no
0	0	0	0	0	0	0	0	0	0	0	0	no
0	0	0	0	0	0	0	0	0	0	0	1	yes
0	0	0	0	0	0	0	0	0	0	0	0	no
0	0	0	0	0	0	0	0	0	0	0	0	no
0	0	0	0	0	0	0	0	0	0	0	0	no
0	0	0	0	0	0	0	0	0	0	0	0	no
0	0	0	0	0	0	0	0	0	0	0	0	no
0	0	0	0	0	0	0	0	0	0	0	0	no
0	0	0	0	0	0	0	0	0	0	0	0	no
0	0	0	0	0	0	0	0	0	0	0	0	no
0	0	0	0	0	0	0	0	0	0	0	0	no
0	0	0	0	0	0	0	0	0	0	0	0	no
0	0	0	0	0	0	0	0	0	0	0	0	no
0	0	0	0	0	0	0	0	0	0	0	0	no
0	0	0	0	0	0	0	0	0	0	0	0	no
0	0	0	0	0	0	0	0	0	0	0	0	no
0	0	0	0	0	0	0	0	0	0	0	0	no
0	0	0	0	0	0	0	0	0	0	0	0	no
0	0	0	0	0	0	0	0	0	0	0	0	no
0	0	0	0	0	0	0	0	0	0	0	0	no
0	0	0	0	0	0	0	0	0	0	0	0	no
0	0	0	0	0	0	0	0	0	0	0	0	no
0	0	0	0	0	0	0	0	0	0	0	0	no
0	0	0	0	0	0	0	0	0	0	0	0	no
0	0	0	0	0	0	0	0	0	0	0	0	no
0	0	0	0	0	0	0	0	0	0	0	0	no
0	0	0	0	0	0	0	0	0	0	0	0	no
0	0	0	0	0	0	0	0	0	0	0	0	no
0	0	0	0	0	0	0	0	0	0	0	0	no
0	0	0	0	0	0	0	0	0	0	1	0	yes
0	0	0	0	0	0	0	0	0	0	0	0	no
0	0	0	0	0	0	0	0	0	0	0	0	no
0	0	0	0	0	0	0	0	0	0	0	0	no
0	0	0	0	0	0	0	0	0	0	0	0	no
0	0	0	0	0	0	0	0	0	0	0	0	no
0	0	0	0	0	0	0	0	0	0	0	0	no
0	0	0	0	0	0	0	0	0	0	0	0	no

0	0	0	0	0	0	0	0	0	0	0	0	no
0	0	0	0	0	0	0	0	0	0	0	0	no
0	0	0	0	0	0	0	0	0	0	0	0	no
0	0	0	0	0	0	0	0	0	0	0	0	no
0	0	0	0	0	0	0	0	0	0	0	0	no
0	0	0	0	0	0	0	0	0	0	0	0	no
0	0	0	0	0	0	0	0	0	0	0	0	no
0	0	0	0	0	0	0	0	0	0	0	0	no
0	0	0	0	0	0	0	0	0	0	0	0	no
0	0	0	0	0	0	0	0	0	0	0	0	no
0	0	0	0	0	0	0	0	0	0	0	0	no
1	0	0	0	0	0	0	1	0	0	0	0	yes
0	0	0	0	0	0	0	0	0	0	0	0	no
0	0	0	0	0	0	0	0	0	0	0	0	no
0	0	0	0	0	0	0	0	0	0	0	0	no
0	0	0	0	0	0	0	0	0	0	0	0	no
0	0	0	0	0	0	0	0	0	0	0	0	no
0	0	0	0	0	0	0	0	0	0	0	0	no
0	0	0	0	0	0	0	0	0	0	0	0	no
0	0	0	0	0	0	0	0	0	0	0	0	no
0	0	0	0	0	0	0	0	0	0	0	0	no
0	0	0	0	0	0	0	0	0	0	0	0	no
0	0	0	0	0	0	0	0	0	0	0	0	no
0	0	0	0	0	0	0	0	0	0	0	0	no
0	0	0	0	0	0	0	0	0	0	0	0	no
0	0	0	0	0	0	0	0	0	0	0	0	no
0	0	0	0	0	0	0	0	0	0	0	0	no
0	0	0	0	0	0	0	0	0	0	0	0	no
0	0	0	0	0	0	0	0	0	0	0	0	no
0	0	0	0	0	0	0	0	0	0	0	0	no
0	0	0	0	0	0	0	0	0	0	1	0	yes
0	0	0	0	0	0	0	0	0	0	0	0	no
0	0	0	0	0	0	0	0	0	0	0	0	no
0	0	0	0	0	0	0	0	0	0	0	0	no
0	0	0	0	0	0	0	0	0	0	0	0	no
0	0	0	0	0	0	0	0	0	0	0	0	no
0	0	0	0	0	0	0	0	0	0	0	0	no
0	0	0	0	0	0	0	0	0	0	0	0	no
0	0	0	0	0	0	0	0	0	0	0	0	no
0	0	0	0	0	0	0	0	0	0	0	0	no

0	0	0	0	0	0	0	0	0	0	0	0	no
0	0	0	0	0	0	0	0	0	0	0	0	no
0	0	0	0	0	0	0	0	0	0	0	0	no
0	0	0	0	0	0	0	0	0	0	0	0	no
0	0	0	0	0	0	0	0	0	0	0	0	no
0	0	0	0	0	0	0	0	0	0	0	0	no
0	0	0	0	0	0	0	0	0	0	0	0	no
0	0	0	0	0	0	0	0	0	0	0	0	no
0	0	0	0	0	0	0	0	0	0	0	0	no
0	0	0	0	0	0	0	0	0	0	0	0	no
0	0	0	0	0	0	0	0	0	0	0	0	no
0	0	0	0	0	0	0	0	0	0	0	0	no
0	0	0	0	0	0	0	0	0	0	0	0	no
0	0	0	0	0	0	0	0	0	0	0	0	no
0	0	0	0	0	0	0	0	0	0	0	0	no
0	0	0	0	0	0	0	0	0	0	0	0	no
0	0	0	0	0	0	0	0	0	0	0	0	no
0	0	0	0	0	0	0	0	0	0	0	0	no
0	0	0	0	0	0	0	0	0	0	0	0	no
0	0	0	0	0	0	0	0	0	0	0	0	no
0	0	0	0	0	0	0	0	0	0	0	0	no
0	0	0	0	0	0	0	0	0	0	0	0	no
0	0	0	0	0	0	0	0	0	0	0	0	no
0	0	0	0	0	0	0	0	0	0	0	0	no
0	0	0	0	0	0	0	0	0	0	0	0	no
0	0	0	0	0	0	0	0	0	0	0	0	no
0	0	0	0	0	0	0	0	0	0	0	0	no
0	0	0	0	0	0	0	0	0	0	0	0	no
0	0	0	0	0	0	0	0	0	0	0	0	no
0	0	0	0	0	0	0	0	0	0	0	0	no
0	0	0	0	0	0	0	0	0	0	0	0	no
0	0	0	0	0	0	0	0	0	0	0	0	no
0	0	0	0	0	0	0	0	0	0	0	0	no
0	0	0	0	0	0	0	0	0	0	0	0	no
0	0	0	0	0	0	0	0	0	0	0	0	no
0	0	0	0	0	0	0	0	0	0	0	0	no
0	0	0	0	0	0	0	0	0	0	0	0	no
0	0	0	0	0	0	0	0	0	0	0	0	no
0	0	0	0	0	0	0	0	0	0	0	0	no
0	0	0	0	0	0	0	0	0	0	0	0	no

0	0	0	0	0	0	0	0	0	0	0	0	no
0	0	0	0	0	0	0	0	0	0	0	0	no
0	0	0	0	0	0	0	0	0	0	0	0	no
0	0	0	0	0	0	0	0	0	0	0	0	no
0	0	0	0	0	0	0	0	0	0	0	0	no
0	0	0	0	0	0	0	0	0	0	0	0	no
0	0	0	0	0	0	0	0	0	0	0	0	no
0	0	0	0	0	0	0	0	0	0	0	0	no
0	0	0	0	0	0	0	0	0	0	0	0	no
0	0	0	0	0	0	0	0	0	0	0	0	no
0	0	0	0	0	0	0	0	0	0	0	0	no
0	0	0	0	0	0	0	0	0	0	0	0	no
0	0	0	0	0	0	0	0	0	0	0	0	no
0	0	0	0	0	0	0	0	0	0	0	0	no
0	0	0	0	0	0	0	0	0	0	0	0	no
0	0	0	0	0	0	0	0	0	0	0	0	no
0	0	0	0	0	0	0	0	0	0	0	0	no
0	0	0	0	0	0	0	0	0	0	0	0	no
0	0	0	0	0	0	0	0	0	0	0	0	no
0	0	0	0	0	0	0	0	0	0	0	0	no
0	0	0	0	0	0	0	0	0	0	0	0	no
0	0	0	0	0	0	0	0	0	0	0	0	no
0	0	0	0	0	0	0	0	0	0	0	0	no
0	0	0	0	0	0	0	0	0	0	0	0	no
0	0	0	0	0	0	0	0	0	0	0	0	no
0	0	0	0	0	0	0	0	0	0	0	0	no
0	0	0	0	0	0	0	0	0	0	0	0	no
0	0	0	0	0	0	0	0	0	0	0	0	no
1	0	0	0	0	0	0	0	0	0	0	0	yes
0	0	0	0	0	0	0	0	0	0	0	0	no
0	0	0	0	0	0	0	0	0	0	0	0	no
0	0	0	0	0	0	0	0	0	0	0	0	no
0	0	0	0	0	0	0	0	0	0	0	0	no
0	0	0	0	0	0	0	0	0	0	0	0	no
0	0	0	0	0	0	0	0	0	0	0	0	no
0	0	0	0	0	0	0	0	0	0	0	0	no
0	0	0	0	0	0	0	0	0	0	0	0	no
0	0	0	0	0	0	1	0	0	0	0	0	yes
0	0	0	0	0	0	0	0	0	0	0	0	no
0	0	0	0	0	0	0	0	0	0	0	0	no

0	0	0	0	0	0	0	0	0	0	0	0	no
0	0	0	0	0	0	0	0	0	0	0	0	no
0	0	0	0	0	0	0	0	0	0	0	0	no
0	0	0	0	0	0	0	0	0	0	0	0	no
0	0	0	0	0	0	0	0	0	0	0	0	no
0	0	0	0	0	0	0	0	0	0	0	0	no
0	0	0	0	0	0	0	0	0	0	0	0	no
0	0	0	0	0	0	0	0	0	0	0	0	no
0	0	0	0	0	0	0	0	0	0	0	0	no
0	0	0	0	0	0	0	0	0	0	0	0	no
0	0	0	0	0	0	0	0	0	0	0	0	no
0	0	0	0	0	0	0	0	0	0	0	0	no
0	0	0	0	0	0	0	0	0	0	0	0	no
0	0	0	0	0	0	0	0	0	0	0	0	no
0	0	0	0	0	0	0	0	0	0	0	0	no
0	0	0	0	0	0	0	0	0	0	0	0	no
0	0	0	0	0	0	0	0	0	0	0	0	no
0	0	0	0	0	0	0	0	0	0	0	0	no
0	0	0	0	0	0	0	0	0	0	0	0	no
0	0	0	0	0	0	0	0	0	0	0	0	no
0	0	0	0	0	0	0	0	0	0	0	0	no
0	0	0	0	0	0	0	0	0	0	0	0	no
0	0	0	0	0	0	0	0	0	0	0	0	no
0	0	0	0	0	0	0	0	0	0	0	0	no
0	0	0	0	0	0	0	0	0	0	0	0	no
0	0	0	0	0	0	0	0	0	0	0	0	no
0	0	0	0	0	0	0	0	0	0	0	0	no
0	0	0	0	0	0	0	0	0	0	0	0	no
0	0	0	0	0	0	0	0	0	0	0	0	no
0	0	0	0	0	0	0	0	0	0	0	0	no
0	0	0	0	0	0	0	0	0	0	0	0	no
0	0	0	0	0	0	0	0	0	0	0	0	no
0	0	0	0	0	0	0	0	0	0	0	0	no
0	0	0	0	0	0	0	0	0	0	0	0	no
0	0	0	0	0	0	0	0	0	0	0	0	no
0	0	0	0	0	0	0	0	0	0	0	0	no
0	0	0	0	0	0	0	0	0	0	0	0	no
0	0	0	0	0	0	0	0	0	0	0	0	no
0	0	0	0	0	0	0	0	0	0	0	0	no
0	0	0	0	0	0	0	0	0	0	0	0	no

0	0	0	0	0	0	0	0	0	0	0	0	no
0	0	0	0	0	0	0	0	0	0	0	0	no
0	0	0	0	0	0	0	0	0	0	0	0	no
0	0	0	0	0	0	0	0	0	0	0	0	no
0	0	0	0	0	0	0	0	0	0	0	0	no
0	0	0	0	0	0	0	0	0	0	0	0	no
0	0	0	0	0	0	0	0	0	0	0	0	no
0	0	0	0	0	0	0	0	0	0	0	0	no
0	0	0	0	0	0	0	0	0	0	0	0	no
0	0	0	0	0	0	0	0	0	0	0	0	no
0	0	0	0	0	0	0	0	0	0	0	0	no
0	0	0	0	0	0	0	0	0	0	0	0	no
0	0	0	0	0	0	0	0	0	0	0	0	no
0	0	0	0	0	0	0	0	0	0	0	0	no
0	0	0	0	0	0	0	0	0	0	0	0	no
0	0	0	0	0	0	0	0	0	0	0	0	no
0	0	0	0	0	0	0	0	0	0	0	0	no
0	0	0	0	0	0	1	0	0	0	0	0	yes
0	0	0	0	0	0	0	0	0	0	0	0	no
0	0	0	0	0	0	0	0	0	0	0	0	no
0	0	0	0	0	0	0	0	0	0	0	0	no
0	0	0	0	0	0	0	0	0	0	0	0	no
0	0	0	0	0	0	0	0	0	0	0	0	no
0	0	0	0	0	0	0	0	0	0	0	0	no
0	0	0	0	0	0	0	0	0	0	0	0	no
0	0	0	0	0	0	0	0	0	0	0	0	no
0	0	0	0	0	0	0	0	0	0	0	0	no
0	0	0	0	0	0	0	0	0	0	0	0	no
0	0	0	0	0	0	0	0	0	0	0	0	no
0	0	0	0	0	0	0	0	0	0	0	0	no
0	0	0	0	0	0	0	0	0	0	0	0	no
0	0	0	0	0	0	0	1	0	0	0	0	yes
0	0	0	0	0	0	0	0	0	0	0	0	no
0	0	0	0	0	0	0	0	0	0	0	0	no
0	0	0	0	0	0	0	0	0	0	0	0	no
0	0	0	0	0	0	0	0	0	0	0	0	no
0	0	0	0	0	0	0	0	0	0	0	0	no
0	0	0	0	0	0	0	0	0	0	0	0	no
0	0	0	0	0	0	0	0	0	0	0	0	no
0	0	0	0	0	0	0	0	0	0	0	0	no

0	0	0	0	0	0	0	0	0	0	0	0	no
0	0	0	0	0	0	0	0	0	0	0	0	no
0	0	0	0	0	0	0	0	0	0	0	0	no
0	0	0	0	0	0	0	0	0	0	0	0	no
0	0	0	0	0	0	0	0	0	0	0	0	no
0	0	0	0	0	0	0	0	0	0	0	0	no
0	0	0	0	0	0	0	0	0	0	0	0	no
0	0	0	0	0	0	0	0	0	0	0	0	no
0	0	0	0	0	0	0	0	0	0	0	0	no
0	0	0	0	0	0	0	0	0	0	0	0	no
0	0	0	0	0	0	0	0	0	0	0	0	no
0	0	0	0	0	0	0	0	0	0	0	0	no
0	0	0	0	0	0	0	0	0	0	0	0	no
0	0	0	0	0	0	0	0	0	0	0	0	no
0	0	0	0	0	0	0	0	0	0	0	0	no
0	0	0	0	0	0	0	0	0	0	0	0	no
0	0	0	0	0	0	0	0	0	0	1	0	yes
0	0	0	0	0	0	0	0	0	0	0	0	no
0	0	0	0	0	0	0	0	0	0	0	0	no
0	0	0	0	0	0	0	0	0	0	0	0	no
0	0	0	0	0	0	0	0	0	0	0	0	no
0	0	0	0	0	0	0	0	0	0	0	0	no
0	0	0	0	0	0	0	0	0	0	0	0	no
0	0	0	0	0	0	0	0	0	0	0	0	no
0	0	0	0	0	0	0	0	0	0	0	0	no
0	0	0	0	0	0	0	0	0	0	0	0	no
0	0	0	0	0	0	0	0	0	0	0	0	no
0	0	0	0	0	0	0	0	0	0	0	0	no
0	0	0	0	0	0	0	0	0	0	0	0	no
0	0	0	0	0	0	0	0	0	0	0	0	no
0	0	0	0	0	0	0	0	0	0	0	0	no
0	0	0	0	0	0	0	0	0	0	0	0	no
0	0	0	0	0	0	0	0	0	0	0	0	no
0	0	0	0	0	0	0	0	0	0	0	0	no
0	0	0	0	0	0	0	0	0	0	0	0	no
0	0	0	0	0	0	0	0	0	0	0	0	no
0	0	0	0	0	0	0	0	0	0	0	0	no
0	0	0	0	0	0	0	1	0	0	0	0	yes
0	0	0	0	0	0	0	0	0	0	0	0	no
0	0	0	0	0	0	0	0	0	0	0	0	no

0	0	0	0	0	0	0	0	0	0	0	0	no
0	0	0	0	0	0	0	0	0	0	0	0	no
0	0	0	0	0	0	0	0	0	0	0	0	no
0	1	0	0	1	0	0	0	0	0	0	0	yes
0	0	0	0	0	0	0	0	0	0	0	0	no
0	0	0	0	0	0	0	0	0	0	0	0	no
0	0	0	0	0	0	0	0	0	0	0	0	no
0	0	0	0	0	0	0	0	0	0	0	0	no
0	0	0	0	0	0	0	0	0	0	0	0	no
0	0	0	0	0	0	0	0	0	0	0	0	no
0	0	0	0	0	0	0	0	0	0	0	0	no
0	0	0	0	0	0	0	0	0	0	0	0	no
0	0	0	0	0	0	0	0	0	0	0	0	no
0	0	0	0	0	0	0	0	0	0	0	0	no
0	0	0	0	0	0	0	0	0	0	0	0	no
0	0	0	0	0	0	0	0	0	0	0	0	no
0	0	0	0	0	0	0	0	0	0	0	0	no
0	0	0	0	0	0	0	0	0	0	0	0	no
0	0	0	0	0	0	0	0	0	0	0	0	no
0	0	0	0	0	0	0	0	0	0	0	0	no
0	0	0	0	0	0	0	0	0	0	0	0	no
0	0	0	0	0	0	1	0	0	0	0	0	yes
0	0	0	0	0	0	0	0	0	0	0	0	no
0	0	0	0	0	0	0	0	0	0	0	0	no
0	0	0	0	0	0	0	0	0	0	0	0	no
0	0	0	0	0	0	0	0	0	0	0	0	no
0	0	0	0	0	0	0	0	0	0	0	0	no
0	0	0	0	0	0	0	0	0	0	0	0	no
0	0	0	0	0	0	0	0	0	0	0	0	no
0	0	0	0	0	0	0	0	0	0	0	0	no
0	0	0	0	0	0	0	0	0	0	0	0	no
0	0	0	0	0	0	0	0	1	0	0	0	yes
0	0	0	0	0	0	0	0	0	0	0	0	no
0	0	0	0	0	0	0	0	0	0	0	0	no
0	0	0	0	0	0	0	0	0	0	0	0	no
0	0	0	0	0	0	0	0	0	0	0	0	no
0	0	0	0	0	0	0	0	0	0	0	0	no
0	0	0	0	0	0	0	0	0	0	0	0	no
0	0	0	0	0	0	0	0	1	1	0	0	yes
0	0	0	0	0	0	0	0	0	0	0	0	no

0	0	0	0	0	0	0	0	0	0	0	0	no
0	0	0	0	0	0	0	0	0	0	0	0	no
0	0	0	0	0	0	0	0	0	0	0	0	no
0	0	0	0	0	0	0	0	0	0	0	0	no
0	0	0	0	0	0	0	0	0	0	0	0	no
0	0	0	0	0	0	0	0	0	0	0	0	no
0	0	0	0	0	0	0	0	0	0	0	0	no
0	0	0	0	0	0	0	0	0	0	0	0	no
0	0	0	0	0	0	0	0	0	0	0	0	no
0	0	0	0	0	0	0	0	0	0	0	0	no
0	0	0	0	0	0	0	0	0	0	0	0	no
0	0	0	0	0	0	0	0	0	0	0	0	no
0	0	0	0	0	0	0	0	0	0	0	0	no
0	0	0	0	0	0	0	0	0	0	0	0	no
0	0	0	0	0	0	0	0	0	0	0	0	no
0	0	0	0	0	0	0	0	0	0	0	0	no
0	0	0	0	0	0	0	0	0	0	0	0	no
0	0	0	0	0	0	0	0	0	0	0	0	no
0	0	0	0	0	0	0	0	0	0	0	0	no
0	0	0	0	0	0	0	0	0	0	0	0	no
0	0	0	0	0	0	0	0	0	0	0	0	no
0	0	0	0	0	0	0	0	0	0	0	0	no
0	0	0	0	0	0	0	0	0	0	0	0	no
0	0	0	0	0	0	0	0	0	0	0	0	no
0	0	0	0	0	0	0	0	0	0	0	0	no
0	0	0	0	0	0	0	0	0	0	0	0	no
0	0	0	0	0	0	0	0	0	0	0	0	no
0	0	0	0	0	0	0	0	0	0	0	0	no
0	0	0	0	0	0	0	0	0	0	0	0	no
0	0	0	0	0	0	0	0	0	0	0	0	no
0	0	0	0	0	0	0	0	0	0	0	0	no
0	0	0	0	0	0	0	0	0	0	0	0	no
0	0	0	0	0	0	0	0	0	0	0	0	no
0	0	0	0	0	0	0	0	0	0	0	0	no
0	0	0	0	0	0	0	0	0	0	0	0	no
0	0	0	0	0	0	0	0	0	0	0	0	no
0	0	0	0	0	0	0	0	0	0	0	0	no
0	0	0	0	0	0	0	0	0	0	0	0	no
0	0	0	0	0	0	0	0	0	0	0	0	no
0	0	0	0	0	0	0	0	0	0	0	0	no

0	0	0	0	0	0	0	0	0	0	0	0	no
0	0	0	0	0	0	0	0	0	0	0	0	no
0	0	0	0	0	0	0	0	0	0	0	0	no
0	0	0	0	0	0	0	0	0	0	0	0	no
0	0	0	0	0	0	0	0	0	0	0	0	no
0	0	0	0	0	0	0	0	0	0	0	0	no
0	0	0	0	0	0	0	0	0	0	0	0	no
0	0	0	0	0	0	0	0	0	0	0	0	no
0	0	0	0	0	0	0	0	0	0	0	0	no
0	0	0	0	0	0	0	0	0	0	0	0	no
0	0	0	0	0	0	0	0	0	0	0	0	no
0	0	0	0	0	0	0	0	0	0	0	0	no
0	0	0	0	0	0	0	0	0	0	0	0	no
0	0	0	0	0	0	0	0	0	0	0	0	no
0	0	0	0	0	0	0	0	0	0	0	0	no
0	0	0	0	0	0	0	0	0	0	0	0	no
0	0	0	0	0	0	0	0	0	0	0	0	no
0	0	0	0	0	0	0	0	0	0	0	0	no
0	0	0	0	1	1	0	1	0	0	1	0	yes
0	0	0	0	0	0	0	0	0	0	0	0	no
0	0	0	0	0	0	0	0	0	0	0	0	no
0	0	0	0	0	0	0	0	0	0	0	0	no
0	0	0	0	0	0	0	0	0	0	0	0	no
0	0	0	0	0	0	0	0	0	0	0	0	no
0	0	0	0	0	0	0	0	0	0	0	0	no
0	0	0	0	0	0	1	0	0	0	0	0	yes
0	0	0	0	0	0	0	0	0	0	0	0	no
0	0	0	0	0	0	0	0	0	0	0	0	no
0	0	0	0	0	0	0	0	0	0	0	0	no
0	0	0	0	0	0	0	0	0	0	0	0	no
0	0	0	0	0	0	0	0	0	0	0	0	no
0	0	0	0	0	0	0	0	0	0	0	0	no
0	0	0	0	0	0	0	0	0	0	0	0	no
0	0	0	0	0	0	0	0	0	0	1	0	yes
0	0	0	0	0	0	0	0	0	0	0	0	no
0	0	0	0	0	0	0	0	0	0	0	0	no
0	0	0	0	0	0	0	0	0	0	0	0	no
0	0	0	0	0	0	0	0	0	0	0	0	no
0	0	0	0	0	0	0	0	0	0	0	0	no
0	0	0	0	0	0	0	0	0	0	0	0	no

0	0	1	0	0	0	0	0	0	0	0	0	yes
0	0	0	0	0	0	0	0	0	0	0	0	no
0	0	0	0	0	0	0	0	0	0	0	0	no
0	0	0	0	0	0	0	0	0	0	0	0	no
0	0	0	0	0	0	0	0	0	0	0	0	no
1	0	0	0	0	0	0	0	0	0	0	0	yes
0	0	0	0	0	0	0	0	0	0	0	0	no
0	0	0	0	0	0	0	0	0	0	0	0	no
0	0	0	0	0	0	0	0	0	0	0	0	no
0	0	0	0	0	0	0	0	0	0	0	0	no
0	0	0	0	0	0	0	0	0	0	0	0	no
0	0	0	0	0	0	0	0	0	0	0	0	no
0	0	0	0	0	0	0	0	0	0	0	0	no
0	0	0	0	0	0	0	0	0	0	0	0	no
0	0	0	0	0	0	0	0	0	0	0	0	no
0	0	0	0	0	0	0	0	0	0	0	0	no
0	0	0	0	0	0	0	0	0	0	0	0	no
0	0	0	0	0	0	0	0	0	0	0	0	no
0	0	0	0	0	0	0	0	0	0	0	0	no
0	0	0	0	0	0	0	0	0	0	0	0	no
0	0	0	0	0	0	0	0	0	0	0	0	no
0	0	0	0	0	0	0	0	0	0	0	0	no
0	0	0	0	0	0	0	0	0	0	0	0	no
0	0	0	0	0	0	0	0	0	0	0	0	no
0	0	0	0	0	0	0	0	0	0	0	0	no
0	1	0	0	1	0	0	1	0	0	1	0	yes
0	0	0	0	0	0	0	0	0	0	0	0	no
0	0	0	0	0	0	0	0	0	0	0	0	no
0	0	0	0	0	0	0	0	0	0	0	0	no
0	0	0	0	0	0	0	0	0	0	0	0	no
0	0	0	0	0	0	0	0	0	0	0	0	no
0	0	0	0	0	0	0	0	0	0	0	0	no
0	0	0	0	0	0	0	0	0	0	0	0	no
0	0	0	0	0	0	0	0	0	0	1	0	yes
0	0	0	0	0	0	0	0	0	0	0	0	no
0	0	0	0	0	0	0	0	0	0	0	0	no
0	0	0	0	0	0	0	0	0	0	0	0	no
0	0	0	0	0	0	0	0	0	0	0	0	no
0	0	0	0	0	0	0	0	0	0	0	0	no
0	0	0	0	0	0	0	0	0	0	0	0	no

0	0	0	0	0	0	0	0	0	0	0	0	no
0	0	0	0	0	0	0	0	0	0	0	0	no
0	0	0	0	0	0	0	0	0	0	0	0	no
0	0	0	0	0	0	0	0	0	0	0	0	no
0	0	0	0	0	0	0	0	0	0	0	0	no
0	0	0	0	0	0	0	0	0	0	0	0	no
0	0	0	0	0	0	0	0	0	0	0	0	no
0	0	0	0	0	0	0	0	0	0	0	0	no
0	0	0	0	0	0	0	0	0	0	0	0	no
0	0	0	0	0	0	0	0	0	0	0	0	no
0	0	0	0	0	0	0	0	0	0	0	0	no
0	0	0	0	0	1	0	0	0	0	0	0	yes
0	0	0	0	0	1	0	0	0	0	0	0	yes
0	0	0	0	0	0	0	0	0	0	0	0	no
0	0	0	0	0	0	0	0	0	0	0	0	no
0	0	0	0	0	0	0	0	0	0	0	0	no
0	0	0	0	0	0	0	0	0	0	0	0	no
0	0	0	0	0	0	0	0	0	0	0	0	no
0	0	0	0	0	0	0	0	0	0	0	0	no
0	0	0	0	0	0	0	0	0	0	0	0	no
0	0	0	0	0	0	0	0	0	0	0	0	no
0	0	0	0	0	0	0	0	0	0	0	0	no
0	0	0	0	0	0	0	0	0	0	0	0	no
0	0	0	0	0	0	0	0	0	0	0	0	no
0	0	0	0	0	0	0	0	0	0	0	0	no
0	0	0	0	0	0	0	0	0	0	0	0	no
0	0	0	0	0	0	0	0	0	0	0	0	no
0	0	0	0	0	0	0	0	0	0	0	0	no
0	0	0	0	0	0	0	0	0	0	0	0	no
0	0	0	0	0	0	0	0	0	0	0	0	no
0	0	0	0	0	0	0	0	0	0	0	0	no
0	0	0	0	0	0	0	0	0	0	0	0	no
0	0	0	0	0	0	0	0	0	0	0	0	no
0	0	0	0	0	0	0	0	0	0	0	0	no
0	0	0	0	0	0	0	0	0	0	0	0	no
0	0	0	0	0	0	0	0	0	0	0	0	no
0	0	0	0	0	0	0	0	0	0	0	1	yes
0	0	0	0	0	0	0	0	0	0	0	0	no
0	0	0	0	0	0	0	0	0	0	0	0	no
0	0	0	0	0	0	0	0	0	0	0	0	no

0	0	0	0	0	0	0	0	0	0	0	0	no
0	0	0	0	0	0	0	0	0	0	0	0	no
0	0	0	0	0	0	0	0	0	0	0	0	no
0	0	0	0	0	0	0	0	0	0	0	0	no
0	0	0	0	0	0	0	0	0	0	0	0	no
0	0	0	0	0	0	0	0	0	0	0	0	no
0	0	0	0	0	0	0	0	0	0	0	0	no
0	0	0	0	0	0	0	0	0	0	0	0	no
0	0	1	0	0	0	0	0	0	0	0	0	yes
0	0	0	0	0	0	0	0	0	0	0	0	no
0	0	0	0	0	0	0	0	0	0	0	0	no
0	0	0	0	0	0	0	0	0	0	0	0	no
0	0	0	0	0	0	0	0	0	0	0	0	no
0	0	0	0	0	0	0	0	0	0	0	0	no
0	0	0	0	0	0	0	0	0	0	0	0	no
0	0	0	0	0	0	0	0	0	0	0	0	no
0	0	0	0	0	0	0	0	0	0	0	0	no
0	0	0	0	0	0	0	0	0	0	0	0	no
0	0	0	0	0	0	0	0	0	0	0	0	no
0	0	0	0	0	0	0	0	0	0	0	0	no
0	0	0	0	0	0	0	0	0	0	0	0	no
0	0	0	0	0	0	0	0	0	0	0	0	no
0	0	0	0	0	0	0	0	0	0	0	0	no
0	0	0	0	0	0	0	0	0	0	1	0	yes
0	0	0	0	0	0	0	0	0	0	0	0	no
0	0	0	0	0	0	0	0	0	0	0	0	no
0	0	0	0	0	0	0	0	0	0	0	0	no
0	0	0	0	0	0	0	0	0	0	0	0	no
0	0	0	0	0	1	0	0	0	1	0	0	yes
0	0	0	0	0	0	0	0	0	0	0	0	no
0	0	0	0	0	0	0	0	0	0	0	0	no
0	0	0	0	0	0	0	0	0	0	0	0	no
0	0	0	1	0	0	0	0	0	0	0	0	yes
0	0	0	0	0	0	0	0	0	0	0	0	no
0	0	0	0	0	0	0	0	0	0	0	0	no
0	0	0	0	0	0	0	0	0	0	0	0	no
0	0	0	0	0	0	0	0	0	0	0	0	no
0	0	0	0	0	0	0	0	0	0	0	0	no
0	0	0	0	0	0	0	0	0	0	0	0	no
0	0	0	0	0	0	0	0	0	0	0	0	no

0	0	0	0	0	0	0	0	0	0	0	0	no
0	0	0	0	0	0	0	0	0	0	0	0	no
0	0	0	0	0	0	0	0	0	0	0	0	no
0	0	0	1	0	0	0	0	0	0	0	0	yes
0	0	0	0	0	0	0	0	0	0	0	0	no
0	0	0	0	0	0	0	0	0	0	0	0	no
0	0	0	0	0	0	0	0	0	0	0	0	no
0	0	0	0	0	0	0	0	0	0	0	0	no
0	0	0	0	0	0	0	0	0	0	0	0	no
0	0	0	0	0	0	0	0	0	0	0	0	no
0	0	0	0	0	0	0	0	0	0	0	0	no
0	0	0	0	0	0	0	0	0	0	0	0	no
0	0	0	0	1	0	0	0	0	0	0	0	yes
0	0	0	0	0	0	0	0	0	0	0	0	no
0	0	0	0	0	0	0	0	0	0	0	0	no
0	0	0	0	0	0	0	0	0	0	0	0	no
0	0	0	0	0	0	0	0	0	0	0	0	no
0	0	0	0	0	0	0	0	0	0	0	0	no
0	0	0	0	0	0	0	0	0	0	0	0	no
0	0	0	0	0	0	0	0	0	0	0	0	no
0	0	0	0	0	0	0	0	0	0	0	0	no
0	0	0	0	0	0	0	0	0	0	0	0	no
0	0	0	0	0	0	0	0	0	0	0	0	no
0	0	0	0	0	0	0	0	0	0	0	0	no
0	0	0	0	0	0	0	0	0	0	0	0	no
0	0	0	0	0	0	0	0	0	0	0	0	no
0	0	0	0	0	0	0	0	0	0	0	0	no
0	0	0	0	0	0	0	0	0	0	0	0	no
0	0	0	0	0	0	0	0	0	0	0	0	no
0	0	0	0	0	0	0	0	0	0	0	0	no
0	0	0	0	0	0	0	0	0	0	0	0	no
0	0	0	0	0	0	0	0	0	0	0	0	no
0	0	0	0	0	0	0	0	0	0	0	0	no
0	0	0	0	0	0	0	0	0	0	0	0	no
0	0	0	0	0	0	0	0	0	0	0	0	no
0	0	0	0	0	0	0	0	0	0	0	0	no
0	0	0	0	0	0	0	0	0	0	0	0	no
0	0	0	0	0	0	0	0	0	0	0	0	no
0	0	0	0	0	0	0	0	0	0	0	0	no
0	0	0	0	0	0	0	0	0	0	0	0	no

0	0	0	0	0	0	0	0	0	0	0	0	no
0	0	0	0	0	0	0	0	0	0	0	0	no
0	0	0	0	0	0	0	0	0	0	0	0	no
0	0	0	0	0	0	0	0	0	0	0	0	no
0	0	0	0	0	0	0	0	0	0	0	1	yes
0	0	0	0	0	0	0	0	0	0	0	0	no
0	0	0	0	0	0	0	0	1	0	0	0	yes
0	0	0	0	0	0	0	0	0	0	0	0	no
0	0	0	0	0	0	0	0	0	0	0	0	no
0	0	0	0	0	0	0	0	0	0	0	0	no
0	0	0	0	0	0	0	0	0	0	0	0	no
0	0	0	1	0	0	1	0	0	0	1	0	no
0	0	0	0	0	0	0	0	0	0	0	0	no
0	0	0	0	0	0	0	0	0	0	0	0	no
0	0	0	0	0	0	0	0	0	0	0	0	no
0	0	0	0	0	0	0	0	0	0	0	0	no
0	0	0	0	0	0	0	0	0	0	0	0	no
0	0	0	0	0	0	0	0	0	0	0	0	no
0	0	0	0	0	0	0	0	0	0	0	0	no
0	0	0	0	0	0	0	0	0	0	0	0	no
0	0	0	0	0	0	0	0	0	0	0	0	no
0	0	0	0	0	0	0	0	0	0	0	0	no
0	0	0	0	0	0	0	0	0	0	0	0	no
0	0	0	0	0	0	0	0	0	0	0	0	no
0	0	0	0	0	0	0	1	0	0	0	0	yes
0	0	0	0	0	0	0	0	0	0	0	0	no
0	0	0	0	0	0	0	0	0	0	0	0	no
0	0	0	0	0	0	0	0	0	0	0	0	no
0	0	0	0	0	0	0	0	0	0	0	0	no
0	0	0	0	1	0	0	0	0	0	0	0	yes
0	0	0	0	0	0	0	0	0	0	0	0	no
0	0	0	0	0	0	0	0	0	0	0	0	no
0	0	0	0	0	0	0	0	0	0	0	0	no
0	0	0	0	0	0	0	0	0	0	0	0	no
0	0	0	0	0	0	0	0	0	0	0	0	no
0	0	0	0	0	0	0	0	0	0	0	0	no
0	0	0	0	0	0	0	0	0	0	1	0	yes
0	0	0	0	0	0	0	0	0	0	0	0	no
0	0	0	0	0	0	0	0	0	0	0	0	no
0	0	0	0	0	0	0	0	0	0	0	0	no

0	0	0	0	0	0	0	0	0	0	0	0	no
0	0	0	0	0	0	0	0	0	0	0	0	no
0	0	0	0	0	0	0	0	0	0	0	0	no
0	0	0	0	0	0	0	0	0	0	0	0	no
0	0	0	0	0	0	0	0	0	0	0	0	no
0	0	0	0	0	0	0	0	0	0	0	0	no
0	0	0	0	0	0	0	0	0	0	0	0	no
0	0	0	0	0	0	0	0	0	0	0	0	no
0	0	0	0	0	0	0	0	0	0	0	0	no
0	0	0	0	0	0	0	0	0	0	0	0	no
0	0	0	0	0	0	0	0	0	0	0	0	no
0	0	0	0	0	0	0	0	0	0	0	0	no
0	0	0	0	0	0	0	0	0	0	0	0	no
0	0	0	0	0	0	0	0	0	0	0	0	no
0	0	0	0	0	0	0	0	0	0	0	0	no
0	0	0	0	0	0	0	0	0	0	0	0	no
0	0	0	0	0	0	0	0	0	0	0	0	no
0	0	0	0	0	0	0	0	0	0	0	0	no
0	0	0	0	0	0	0	0	0	0	0	0	no
0	0	0	0	0	0	0	0	0	0	0	0	no
0	0	0	0	0	0	1	0	0	0	0	0	yes
0	0	0	0	0	0	0	0	0	0	0	0	no
0	0	0	0	0	0	0	0	0	0	0	0	no
0	0	0	0	0	0	0	0	0	0	0	0	no
0	0	0	0	0	0	0	0	0	0	0	0	no
0	0	0	0	0	0	0	0	0	0	0	0	no
0	0	0	0	0	0	0	0	0	0	0	0	no
0	0	0	0	0	0	1	0	0	0	0	0	yes
0	0	0	0	0	0	0	0	0	0	0	0	no
0	0	0	0	0	0	0	0	0	0	0	0	no
0	0	0	0	0	0	0	0	0	0	0	0	no
0	0	0	0	0	0	0	0	0	0	0	0	no
0	0	0	0	0	0	0	0	0	0	0	0	no
0	0	0	0	0	0	0	0	0	0	0	0	no
0	0	0	0	0	0	0	0	0	0	0	0	no
0	0	0	0	0	0	0	0	0	0	0	0	no
0	0	0	0	0	0	0	0	0	0	0	0	no
0	0	0	0	0	0	0	0	0	0	0	0	no
0	0	0	0	0	0	0	0	0	0	0	0	no
0	0	0	0	0	0	0	0	0	0	0	0	no

0	0	0	0	0	0	0	0	0	0	0	0	no
0	0	0	0	0	0	0	0	0	0	0	0	no
0	0	0	0	0	0	0	0	0	0	0	0	no
0	0	0	0	0	0	0	0	0	0	0	0	no
0	0	0	0	0	0	0	0	0	0	0	0	no
0	0	0	0	0	0	0	0	0	0	0	0	no
0	0	0	0	0	0	0	0	0	0	0	0	no
0	0	0	0	0	0	0	0	0	0	0	0	no
0	0	0	0	0	0	0	0	0	0	0	0	no
0	0	0	0	0	0	0	0	0	0	0	0	no
0	0	0	0	0	0	0	0	0	0	0	0	no
0	0	0	0	0	0	0	0	0	0	0	0	no
0	0	0	0	0	0	0	0	0	0	0	0	no
0	0	0	0	0	0	0	0	0	0	0	0	no
0	0	0	0	0	0	0	0	0	0	0	0	no
0	0	0	0	0	0	0	0	0	0	0	0	no
0	0	0	0	0	0	0	0	0	0	0	0	no
0	0	0	0	0	0	0	0	0	0	0	0	no
0	0	0	0	0	0	0	0	0	0	0	0	no
0	0	0	0	0	0	0	0	0	0	0	0	no
0	0	0	0	0	0	0	0	0	0	0	0	no
0	0	0	0	0	0	0	0	0	0	0	0	no
0	0	0	0	0	1	0	0	0	0	0	0	yes
0	0	0	0	0	0	0	0	0	0	0	0	no
0	0	0	0	0	0	0	0	0	0	0	0	no
0	0	0	0	0	0	0	0	0	0	0	0	no
0	0	0	0	0	0	0	0	0	0	0	0	no
0	0	0	0	0	0	0	0	0	0	0	0	no
0	0	0	0	0	0	0	0	0	0	0	0	no
0	0	0	0	0	0	0	0	0	0	0	0	no
0	0	0	0	0	0	0	0	0	0	0	0	no
0	0	0	0	0	0	0	0	0	0	0	0	no
0	0	0	0	0	0	0	0	0	0	0	0	no
0	0	0	0	0	0	0	0	0	0	0	0	no
0	0	0	0	0	0	0	0	0	0	0	0	no
0	0	0	0	0	0	0	0	0	0	0	0	no
0	0	0	0	0	0	0	0	0	0	0	0	no
0	0	0	0	0	0	0	0	0	0	0	0	no
0	0	0	0	0	0	0	0	0	0	0	0	no
0	0	0	0	0	0	0	0	0	0	0	0	no

0	0	0	0	0	0	0	0	0	0	0	0	no
0	0	0	0	0	0	0	0	0	1	0	0	yes
0	0	0	0	0	0	0	0	0	0	0	0	no
0	0	0	0	0	0	0	0	0	0	0	0	no
0	0	0	0	0	0	0	0	0	0	0	0	no
0	0	0	0	0	0	0	0	0	0	0	0	no
0	0	0	0	0	0	0	0	0	0	0	0	no
0	0	0	0	0	0	0	0	0	0	0	0	no
0	0	0	0	0	0	0	0	0	0	0	0	no
0	0	0	0	0	0	0	0	0	0	0	0	no
0	0	0	0	0	0	0	0	0	0	0	0	no
0	0	0	0	0	0	0	0	0	0	0	0	no
0	0	0	0	0	0	0	0	0	0	0	0	no
0	0	0	0	0	0	0	0	0	0	0	0	no
0	0	0	0	0	0	0	0	0	0	0	0	no
0	0	0	0	0	0	0	0	0	0	0	0	no
0	0	0	0	0	0	0	0	0	0	0	0	no
0	0	0	0	0	0	0	0	0	0	0	0	no
0	0	0	0	0	0	0	0	0	0	0	0	no
0	0	0	0	0	0	0	0	0	0	0	0	no
0	0	0	0	0	0	0	0	0	0	0	0	no
0	0	0	0	0	0	0	0	0	0	0	0	no
0	0	0	0	0	0	0	0	0	0	0	0	no
0	0	0	0	0	0	0	0	0	0	0	0	no
0	0	0	0	0	0	0	0	0	0	0	0	no
0	0	0	0	0	0	0	0	0	0	0	0	no
0	0	0	0	0	0	0	0	0	0	0	0	no
0	0	0	0	0	0	0	0	0	0	0	0	no
0	0	0	0	0	0	0	0	0	0	0	0	no
0	0	0	0	0	0	0	0	0	0	0	1	yes
0	0	0	0	0	0	0	0	0	0	0	0	no
0	0	0	0	0	0	0	0	0	0	0	0	no
0	0	0	0	0	0	0	0	0	0	0	0	no
0	0	0	0	0	0	0	0	0	0	0	0	no
0	0	0	0	0	0	0	0	0	0	0	0	no
0	0	0	0	0	0	0	0	0	0	0	0	no
0	0	0	0	0	0	0	0	0	0	0	0	no
0	0	0	0	0	0	0	0	0	0	0	0	no
0	0	0	0	0	0	0	0	0	0	0	0	no
0	0	0	0	0	0	0	0	0	0	0	0	no

0	0	0	0	0	0	0	0	0	0	0	0	no
0	0	0	0	0	0	0	0	0	0	0	0	no
0	0	0	0	0	0	0	0	0	0	0	0	no
0	0	0	0	0	0	0	0	0	0	0	0	no
0	0	0	0	0	0	0	0	0	0	0	0	no
0	0	0	0	0	0	0	0	0	0	0	0	no
0	0	0	0	0	0	0	0	0	0	0	0	no
0	0	0	0	0	0	0	0	0	0	0	0	no
0	0	0	0	0	0	0	0	0	0	0	0	no
0	0	0	0	0	0	0	0	0	0	0	0	no
0	0	0	0	0	0	0	0	0	0	0	0	no
0	0	0	0	0	0	0	0	0	0	0	0	no
0	0	0	0	0	0	0	0	0	0	0	0	no
0	0	0	0	0	0	0	0	0	0	0	0	no
0	0	0	0	0	0	0	0	0	0	0	0	no
0	0	0	0	0	0	0	0	0	0	0	0	no
0	0	0	0	0	0	0	0	0	0	0	0	no
0	0	0	0	0	0	0	0	0	0	0	0	no
0	0	0	0	0	0	0	0	0	0	0	0	no
0	0	0	0	0	0	0	0	0	0	0	0	no
0	0	0	0	0	0	0	0	0	0	0	0	no
0	0	0	0	0	0	0	0	0	0	0	0	no
0	0	0	0	0	0	0	0	0	0	0	0	no
0	0	0	0	0	0	0	0	0	0	1	0	yes
0	0	0	0	0	0	0	0	0	0	0	0	no
0	0	0	0	0	0	0	0	0	0	0	0	no
0	0	0	0	0	0	0	0	0	0	0	0	no
0	0	0	0	0	0	0	0	0	0	0	0	no
0	0	0	0	0	0	0	0	0	0	0	0	no
0	0	0	0	0	0	0	0	0	0	0	0	no
0	0	0	0	0	0	0	0	0	0	0	0	no
0	0	0	0	0	0	0	0	0	0	0	0	no
0	0	0	0	0	0	0	0	0	0	0	0	no
0	0	0	0	0	0	0	0	0	0	0	0	no
0	0	0	0	0	0	0	0	0	0	0	0	no
0	0	0	0	0	0	0	0	0	0	0	0	no
0	0	0	0	0	0	0	0	0	0	0	0	no
0	0	0	0	0	0	0	0	0	0	0	0	no
0	0	0	0	0	0	0	0	0	0	0	0	no
0	0	0	0	0	0	0	0	0	0	0	0	no

0	0	0	0	0	0	0	0	0	0	0	0	no
0	0	0	0	0	0	0	0	0	0	0	0	no
0	0	0	0	0	0	0	0	0	0	0	0	no
0	0	0	0	0	0	0	0	0	0	0	0	no
0	0	0	0	0	0	0	0	0	0	0	0	no
0	0	0	0	0	0	0	0	0	0	0	0	no
0	0	0	0	0	0	0	0	0	0	0	0	no
0	0	0	0	0	0	1	0	0	0	0	0	yes
0	0	0	0	0	0	0	0	0	0	0	0	no
0	0	0	0	0	0	0	0	0	0	0	0	no
0	0	0	0	0	0	0	0	0	0	0	0	no
0	0	0	0	0	0	0	0	0	0	0	0	no
0	0	0	0	0	0	0	0	0	0	0	0	no
0	0	0	0	0	0	0	0	0	0	0	0	no
0	0	0	0	0	0	0	0	0	0	0	0	no
0	0	0	0	0	0	0	0	0	0	0	0	no
0	0	0	0	0	0	0	0	0	0	0	0	no
0	0	0	0	0	0	0	0	0	0	0	0	no
0	0	0	0	0	0	0	0	0	0	0	0	no
0	0	0	0	0	0	0	0	0	0	0	0	no
0	0	0	0	0	0	0	0	0	0	0	0	no
0	0	0	0	0	0	0	0	0	0	0	0	no
0	0	0	0	0	0	0	0	0	0	0	0	no
0	0	0	0	0	0	0	0	0	0	0	0	no
0	0	0	0	0	0	0	0	0	0	0	0	no
0	0	0	0	0	0	0	0	0	0	0	0	no
0	0	0	0	0	0	0	0	0	0	0	0	no
0	0	0	0	0	0	0	0	0	0	0	0	no
0	0	0	0	0	0	0	0	0	0	0	0	no
0	0	0	0	0	0	0	0	0	0	0	0	no
0	0	0	0	0	0	0	0	0	0	0	0	no
0	0	0	0	0	0	0	0	0	0	0	0	no
0	0	0	0	0	0	0	0	0	0	0	0	no
0	0	0	0	0	0	0	0	0	0	0	0	no
0	0	0	0	0	0	0	0	0	0	0	0	no
0	0	0	0	0	0	0	0	0	0	0	0	no
0	0	0	0	0	0	0	0	0	0	0	0	no
0	0	0	0	0	0	0	0	0	0	0	0	no
0	0	0	0	0	0	0	0	0	0	0	0	no
0	0	0	0	0	0	0	0	0	0	0	0	no

0	0	0	0	0	0	0	0	0	0	0	0	no
0	0	0	0	0	0	0	0	0	0	0	0	no
0	0	0	0	0	0	0	0	0	0	0	0	no
0	0	0	0	0	0	0	0	0	0	0	0	no
0	0	0	0	0	0	0	0	0	0	0	0	no
0	0	0	0	0	0	0	0	0	0	0	0	no
0	0	0	0	0	0	0	0	0	0	0	0	no
0	0	0	0	0	0	0	0	0	0	0	0	no
0	0	0	0	0	0	0	0	0	0	0	0	no
0	0	0	0	0	0	0	0	0	0	0	0	no
0	0	0	0	0	0	0	0	0	0	0	0	no
0	0	0	0	0	0	0	0	0	0	0	0	no
0	0	0	0	0	0	0	0	0	0	0	0	no
0	0	0	0	0	0	0	0	0	0	0	0	no
0	0	0	0	0	0	0	0	0	0	0	0	no
0	0	0	0	0	0	0	0	0	0	0	0	no
0	0	0	0	0	0	0	0	0	0	0	0	no
0	0	0	0	0	0	0	0	0	0	0	0	no
0	0	0	0	0	0	0	0	0	0	0	0	no
0	0	0	0	0	0	0	0	0	0	0	0	no
0	0	0	0	0	0	0	0	0	0	0	0	no
0	0	0	0	0	0	0	0	0	0	0	0	no
0	0	0	0	0	0	0	0	0	0	0	0	no
0	0	0	0	0	0	0	0	0	0	0	0	no
0	0	0	0	0	0	0	0	0	0	0	0	no
0	0	0	0	0	0	0	0	0	0	0	0	no
0	0	0	0	0	0	0	0	0	0	0	0	no
0	0	0	0	0	0	0	0	0	0	0	0	no
0	0	0	0	0	0	0	0	0	0	0	0	no
0	0	0	0	0	0	0	0	0	0	0	0	no
0	0	0	0	0	0	0	0	0	0	0	0	no
0	0	0	0	0	0	0	0	0	0	0	0	no
0	0	0	0	0	0	0	0	0	0	0	0	no
0	0	0	0	0	0	0	0	0	0	0	0	no
0	0	0	0	0	0	0	0	0	0	0	0	no
0	0	0	0	0	0	0	0	0	0	0	0	no
0	0	0	0	0	1	0	0	0	0	0	0	yes
0	0	0	0	0	0	0	0	0	0	0	0	no
0	0	0	0	0	0	0	0	0	0	0	0	no
0	0	0	0	0	0	0	0	0	0	0	0	no

0	0	0	0	0	0	0	0	0	0	0	0	no
0	0	0	0	0	0	0	0	0	0	0	0	no
0	0	0	0	0	0	0	0	0	0	0	0	no
0	0	0	0	0	0	1	0	0	0	0	0	yes
0	0	0	0	0	0	0	0	0	0	0	0	no
0	0	0	0	0	0	0	0	0	0	0	0	no
0	0	0	0	0	0	0	0	0	0	0	0	no
0	0	0	0	0	0	0	0	0	0	0	0	no
0	0	0	0	0	0	0	0	0	0	0	0	no
0	0	0	0	0	0	0	0	0	0	0	0	no
0	0	0	0	0	0	1	0	0	0	0	0	yes
0	0	0	0	0	0	0	0	0	0	0	0	no
0	0	0	0	0	0	0	0	0	0	0	0	no
0	0	0	0	0	0	0	0	0	0	0	0	no
0	0	0	0	0	0	0	0	0	0	0	0	no
0	0	0	0	0	0	0	0	0	0	0	0	no
0	0	0	0	0	0	0	0	0	0	0	0	no
0	0	0	0	0	0	0	0	0	0	0	0	no
0	0	0	0	0	0	0	0	0	0	0	0	no
0	0	0	0	0	0	0	0	0	0	0	0	no
0	0	0	0	0	0	0	0	0	0	0	0	no
0	0	0	0	0	0	0	0	0	0	0	0	no
0	0	0	0	0	0	0	0	0	0	0	0	no
0	0	0	0	0	0	0	0	0	0	0	0	no
0	0	0	0	0	0	0	0	0	0	0	0	no
0	0	0	0	0	0	0	0	0	0	0	0	no
0	0	0	0	0	0	0	0	0	0	0	0	no
0	0	0	0	0	0	0	0	0	1	0	0	yes
0	0	0	0	0	0	0	0	0	0	0	0	no
0	0	0	0	0	0	0	0	0	0	0	0	no
0	0	0	0	0	0	0	0	0	0	0	0	no
0	0	0	0	0	0	0	0	0	0	0	0	no
0	0	0	0	0	0	0	0	0	0	0	0	no
0	0	0	0	0	0	0	0	0	0	0	0	no
0	0	0	0	0	0	0	0	0	0	0	0	no
0	0	0	0	0	0	0	0	0	0	0	0	no
0	0	0	0	0	0	0	0	0	0	0	0	no
0	0	0	0	0	0	0	0	0	0	0	0	no
0	0	0	0	0	0	0	0	0	0	0	0	no
0	0	0	0	0	0	0	0	0	0	0	0	no

0	0	0	0	0	0	0	0	0	0	0	0	no
0	0	0	0	0	0	0	0	0	0	0	0	no
0	0	0	0	0	0	0	0	0	0	0	0	no
0	0	0	0	0	0	0	0	0	0	0	0	no
0	0	0	0	0	0	0	0	0	0	0	0	no
0	0	0	0	0	0	0	0	0	0	0	0	no
0	0	0	0	0	0	0	0	0	0	0	0	no
0	0	0	0	0	0	0	0	0	0	0	0	no
0	0	0	0	0	1	0	0	1	0	0	0	yes
0	0	0	0	0	0	0	0	0	0	0	0	no
0	0	0	0	0	0	0	0	0	0	0	0	no
0	0	0	0	0	0	0	0	0	0	0	0	no
0	0	0	0	0	0	0	0	0	0	0	0	no
0	0	0	0	0	0	0	0	0	0	0	0	no
0	0	0	0	0	0	0	0	0	0	0	0	no
0	0	0	0	0	0	0	0	0	0	0	0	no
0	0	0	0	0	0	0	0	0	0	0	0	no
0	0	0	0	0	0	0	0	0	0	0	0	no
0	0	0	0	0	0	0	0	0	0	0	0	no
0	0	0	0	0	0	0	0	0	0	0	0	no
0	0	0	0	0	0	0	0	0	0	0	0	no
0	0	0	0	0	0	0	0	0	0	0	0	no
0	0	0	0	0	0	0	0	0	0	0	0	no
0	0	0	0	0	0	0	0	0	0	0	0	no
0	0	0	0	0	0	0	0	0	0	0	0	no
0	0	0	0	0	0	0	0	0	0	0	0	no
0	0	0	0	0	0	0	0	0	0	0	0	no
0	0	0	0	0	0	0	0	0	0	0	0	no
0	0	0	0	0	0	0	0	0	0	0	0	no
0	0	0	0	0	0	0	0	0	0	0	0	no
0	0	0	0	0	0	0	0	0	0	0	0	no
0	0	0	0	0	0	0	0	0	0	0	0	no
0	0	0	0	0	0	0	0	0	0	0	0	no
0	0	0	0	0	0	0	0	0	0	0	0	no
0	0	0	0	0	0	0	0	0	0	0	0	no
0	0	0	0	0	0	0	0	0	0	0	0	no
0	0	0	0	0	0	0	0	0	0	0	0	no
0	0	0	0	0	0	0	0	0	0	0	0	no
0	0	0	0	0	0	0	0	0	0	0	0	no
0	0	0	0	0	0	0	0	0	0	0	0	no

0	0	0	0	0	0	0	0	0	0	0	0	no
0	0	0	0	0	0	0	0	0	0	0	0	no
0	0	0	0	0	0	0	0	0	0	0	0	no
0	0	0	0	0	0	0	0	0	0	0	0	no
0	0	0	0	0	0	0	0	0	0	0	0	no
0	0	0	0	0	0	0	0	0	0	0	1	no
0	0	0	0	0	0	0	0	0	0	0	0	no
0	0	0	0	0	0	0	0	0	0	0	0	no
0	0	0	0	0	0	0	0	0	1	0	0	yes
0	0	0	0	0	0	0	0	0	0	0	0	no
0	0	0	0	0	0	0	0	0	0	0	0	no
0	0	0	0	0	0	0	0	0	0	0	0	no
0	0	0	0	0	0	0	0	0	0	0	0	no
0	0	0	0	0	0	0	0	0	0	0	0	no
0	0	0	0	0	0	0	0	0	0	0	0	no
0	0	0	0	0	0	0	0	0	0	0	0	no
0	0	0	0	0	0	0	0	0	0	0	0	no
0	0	0	0	0	0	0	0	0	0	0	0	no
0	0	0	0	0	0	0	0	0	0	0	0	no
0	0	0	0	0	0	0	0	0	0	0	0	no
0	0	0	0	0	0	0	0	0	0	0	0	no
0	0	0	0	0	0	0	0	0	0	0	0	no
0	0	0	0	0	0	0	0	0	0	0	0	no
0	0	0	0	0	0	0	0	0	0	0	0	no
0	0	0	0	0	0	0	0	0	0	0	0	no
0	0	0	0	0	0	0	0	0	0	0	0	no
0	0	0	0	0	0	1	0	0	0	0	0	yes
0	0	0	0	0	0	1	0	0	0	0	0	yes
0	0	0	0	0	0	0	0	0	0	0	0	no
0	0	0	0	0	0	0	0	0	0	0	0	no
0	0	0	0	0	0	0	0	0	0	0	0	no
0	0	0	0	0	0	0	0	0	0	0	0	no
0	0	0	0	0	0	0	0	0	0	0	0	no
0	0	0	0	0	0	0	0	0	0	0	0	no
0	0	0	0	0	0	0	0	0	0	0	0	no
0	0	0	0	0	0	0	0	0	0	0	0	no
0	0	0	0	0	0	0	0	0	0	0	0	no
0	0	0	0	0	0	0	0	0	0	0	0	no
0	0	0	0	0	0	0	0	0	0	0	0	no
0	0	0	0	0	0	0	0	0	0	0	0	no

0	0	0	0	0	0	0	0	0	0	0	0	no
0	0	0	0	0	0	0	0	0	0	0	0	no
0	0	0	0	0	0	0	0	0	0	0	0	no
0	0	0	0	0	0	0	0	0	0	0	0	no
0	0	0	0	1	0	1	0	0	0	0	0	yes
0	0	0	0	0	0	0	0	0	0	0	0	no
0	0	0	0	0	0	0	0	0	0	0	0	no
0	0	0	0	0	0	0	0	0	0	0	0	no
0	0	0	0	0	1	0	0	0	0	0	0	yes
0	0	0	0	0	0	0	0	0	0	0	0	no
0	0	0	0	0	0	0	0	0	0	0	0	no
0	0	0	0	0	0	0	0	0	0	0	0	no
0	0	0	0	0	0	0	0	0	0	0	0	no
0	0	0	0	0	0	0	0	0	0	0	0	no
0	0	0	0	0	0	0	0	0	0	0	0	no
0	0	0	0	0	0	0	0	0	0	0	0	no
0	0	0	0	0	0	0	0	0	0	0	0	no
0	0	0	0	0	0	0	0	0	0	0	0	no
0	0	0	0	0	0	0	0	0	0	0	0	no
0	0	0	0	0	0	0	0	0	0	0	0	no
0	0	0	0	0	0	0	0	0	0	0	0	no
0	0	0	0	0	0	0	0	0	0	0	0	no
0	0	0	0	0	0	0	0	0	0	0	0	no
0	0	0	0	0	0	0	0	0	0	0	0	no
0	0	0	0	0	0	0	0	0	0	0	0	no
0	0	0	0	0	0	0	0	0	0	0	0	no
0	0	0	0	0	0	1	0	0	0	1	1	yes
0	0	0	0	0	0	0	0	0	0	0	0	no
0	0	0	0	0	1	0	0	0	0	0	0	yes
0	0	0	0	0	0	0	0	0	0	0	0	no
0	0	0	0	0	0	0	0	0	0	0	0	no
0	0	0	0	0	0	0	0	0	0	0	0	no
0	0	0	0	0	0	0	0	0	0	0	0	no
0	0	0	0	0	0	0	0	0	0	0	0	no
0	0	0	0	0	0	0	0	0	0	0	0	no
0	0	0	0	0	0	0	0	0	0	0	0	no
0	0	0	0	0	0	0	0	0	0	0	0	no
0	0	0	0	0	1	0	0	0	0	0	0	yes
0	0	0	0	0	0	0	0	0	0	0	0	no
0	0	0	0	0	0	0	0	0	0	0	0	no

0	0	0	0	0	0	0	0	0	0	0	0	no
0	0	0	0	0	0	0	0	0	0	0	0	no
0	0	0	0	0	0	0	0	0	0	0	0	no
0	0	0	0	0	0	0	0	0	0	0	0	no
0	0	0	0	0	0	0	0	0	0	0	0	no
0	0	0	0	0	0	0	0	0	0	0	0	no
0	0	0	0	0	0	0	0	0	0	0	0	no
0	0	0	0	0	0	0	0	0	0	0	0	no
0	0	0	0	0	0	0	0	0	0	0	0	no
0	0	0	0	0	0	0	0	0	0	0	0	no
0	0	0	0	0	0	0	0	0	0	0	0	no
0	0	0	0	0	0	0	0	0	0	0	0	no
0	0	0	0	0	0	0	0	0	0	0	0	no
0	0	0	0	0	0	0	0	0	0	0	0	no
0	0	0	0	0	0	0	0	0	0	0	0	no
0	0	0	0	0	0	0	0	0	0	0	0	no
0	0	0	0	0	0	0	0	0	0	0	0	no
0	0	0	0	0	0	0	0	0	0	0	0	no
0	0	0	0	0	0	0	0	0	0	0	0	no
0	0	0	0	0	0	0	0	0	0	0	0	no
0	0	0	0	0	0	0	0	0	0	0	0	no
0	0	0	0	0	0	0	0	0	0	0	0	no
0	0	0	0	0	0	0	0	0	0	0	0	no
0	0	0	0	0	0	0	0	0	0	0	0	no
0	0	0	0	0	0	0	0	0	0	0	0	no
0	0	0	0	0	0	0	0	0	0	0	0	no
0	0	0	0	0	0	0	0	0	0	0	0	no
0	0	0	0	0	0	0	0	0	0	0	0	no
0	0	0	0	0	0	0	0	0	0	0	0	no
0	0	0	0	0	0	0	0	0	0	0	0	no
0	0	0	0	0	0	0	1	0	0	0	0	yes
0	0	0	0	0	0	0	0	0	0	0	0	no
0	0	0	0	0	0	0	0	0	0	0	0	no
0	0	0	0	0	0	0	0	0	0	0	0	no
0	0	0	0	0	0	0	0	0	0	0	0	no
0	0	0	0	0	0	0	0	0	0	0	0	no
0	0	0	0	0	0	0	0	0	0	0	0	no
0	0	0	0	0	0	0	0	0	0	0	0	no
0	0	0	0	0	0	0	0	0	0	0	0	no
0	0	0	0	0	0	0	0	0	0	0	0	no

0	0	0	0	0	0	0	0	0	0	0	0	no
0	0	0	0	0	0	0	0	0	0	0	0	no
0	0	0	0	0	0	0	0	0	0	0	0	no
0	0	0	0	0	0	0	0	0	0	0	0	no
0	0	0	0	0	0	0	0	0	0	0	0	no
0	0	0	0	1	0	0	0	0	0	0	0	yes
0	0	0	0	0	0	0	0	0	0	0	0	no
0	0	0	0	0	0	0	0	0	0	0	0	no
0	0	0	0	0	0	0	0	0	0	0	0	no
0	0	0	0	0	0	0	0	0	0	0	0	no
0	0	0	0	0	0	0	0	0	0	0	0	no
0	0	0	1	0	0	0	0	0	0	0	0	yes
0	0	0	0	0	0	0	0	0	0	0	0	no
0	0	0	0	0	0	0	0	0	0	0	0	no
0	0	0	0	0	0	0	0	0	0	0	0	no
0	0	0	0	0	0	0	0	0	0	0	0	no
0	0	0	0	0	0	0	0	0	0	0	0	no
0	0	0	0	0	0	0	0	0	0	0	0	no
0	0	0	0	0	0	0	0	0	0	0	0	no
0	0	0	0	0	0	0	0	0	0	0	0	no
0	0	0	0	0	0	0	0	0	0	0	0	no
0	0	0	0	0	0	0	0	0	0	0	0	no
0	0	0	0	0	0	0	0	0	0	0	0	no
0	0	0	0	0	0	0	0	0	0	0	0	no
0	0	0	0	0	0	0	0	0	0	0	0	no
0	0	0	0	0	0	0	0	0	0	0	0	no
0	0	0	0	0	0	0	0	0	0	0	0	no
0	0	0	0	0	0	0	0	0	0	0	0	no
0	0	0	0	0	0	0	0	0	0	0	0	no
0	0	0	0	0	0	0	0	0	0	0	0	no
0	0	0	0	0	0	0	0	0	0	0	0	no
0	0	0	0	0	0	0	0	0	0	0	0	no
0	0	0	0	0	0	0	0	0	0	0	0	no
0	0	0	0	0	0	0	0	0	0	0	0	no
0	0	0	0	0	0	0	0	0	0	0	0	no
0	0	0	0	0	0	0	0	0	0	0	0	no
0	0	0	0	0	0	0	0	0	0	0	0	no
0	0	0	0	0	0	0	0	0	0	0	0	no
0	0	0	0	0	0	0	0	0	0	0	0	no
0	0	0	0	0	0	0	0	0	0	0	0	no

0	0	0	0	0	0	0	0	0	0	0	0	no
0	0	0	0	0	0	0	0	0	0	0	0	no
0	0	0	1	0	1	0	0	0	0	0	0	yes
